# Dual nickel and Lewis acid catalysis for cross-electrophile coupling: the allylation of aryl halides with allylic alcohols†
†Electronic supplementary information (ESI) available. CCDC 1515176. For ESI and crystallographic data in CIF or other electronic format see DOI: 10.1039/c7sc03140h


**DOI:** 10.1039/c7sc03140h

**Published:** 2017-11-06

**Authors:** Xue-Gong Jia, Peng Guo, Jicheng Duan, Xing-Zhong Shu

**Affiliations:** a State Key Laboratory of Applied Organic Chemistry (SKLAOC) , College of Chemistry and Chemical Engineering , Lanzhou University , 222 South Tianshui Road , Lanzhou , 730000 , China . Email: shuxingzh@lzu.edu.cn

## Abstract

Controlling the selectivity in cross-electrophile coupling reactions is a significant challenge, particularly when one electrophile is much more reactive. We report a general and practical strategy to address this problem in the reaction between reactive and unreactive electrophiles by a combination of nickel and Lewis acid catalysis. This strategy is used for the coupling of aryl halides with allylic alcohols to form linear allylarenes selectively. The reaction tolerates a wide range of functional groups (*e.g.* silanes, boronates, anilines, esters, alcohols, and various heterocycles) and works with various allylic alcohols. Complementary to most current routes for the C3 allylation of an unprotected indole, this method provides access to C2 and C4–C7 allylated indoles. Preliminary mechanistic experiments reveal that the reaction might start with an aryl nickel intermediate, which then reacts with Lewis acid activated allylic alcohols in the presence of Mn.

## Abstract 2

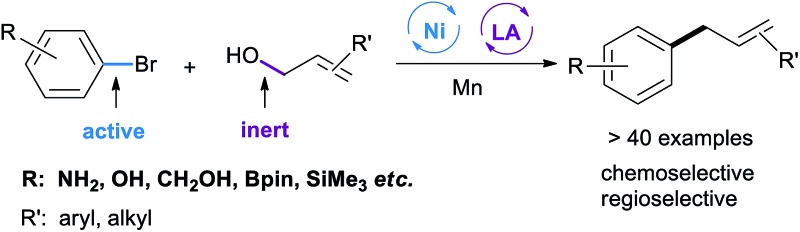
Dual nickel and Lewis acid catalysis has been developed for the coupling reaction between reactive and unreactive electrophiles.

## Introduction

Selective cross-electrophile coupling has recently emerged as an increasingly popular approach for constructing C–C bonds.^1^ The reaction achieves the union of two different bench-stable electrophiles (aryl/alkyl halides, *etc.*) and avoids using air and/or moisture sensitive organometallic reagents (RMgX, RZnX, 
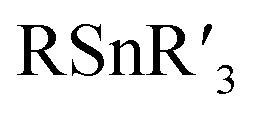
, RB(OH)_2_, *etc.*).^2^ Unlike in conventional cross-coupling, where the selectivity is controlled by the oxidative addition of an electrophile and transmetallation of a nucleophile, generally both electrophiles will compete for oxidative addition at the catalyst in cross-electrophile coupling. Thus, the control of the selectivity for the cross-product over the symmetric dimer is a particular challenge, especially when one electrophile is much more reactive than the other. Indeed, such a reaction has been realized very recently^3^ and significant progress is achieved in nickel catalysis with a metal reductant.^4^ The substrate, however, remains to be confined to reactive electrophiles (R–I, Br, Cl, OTf, *etc.*). Given that inert O-electrophiles (alcohol, phenol, ether, *etc.*) are more readily available and their coupling reactions typically require organometallic reagents,^5^ the development of a strategy to couple these compounds with electrophiles will be highly attractive but remains extremely challenging.^6^

**Scheme 1:**
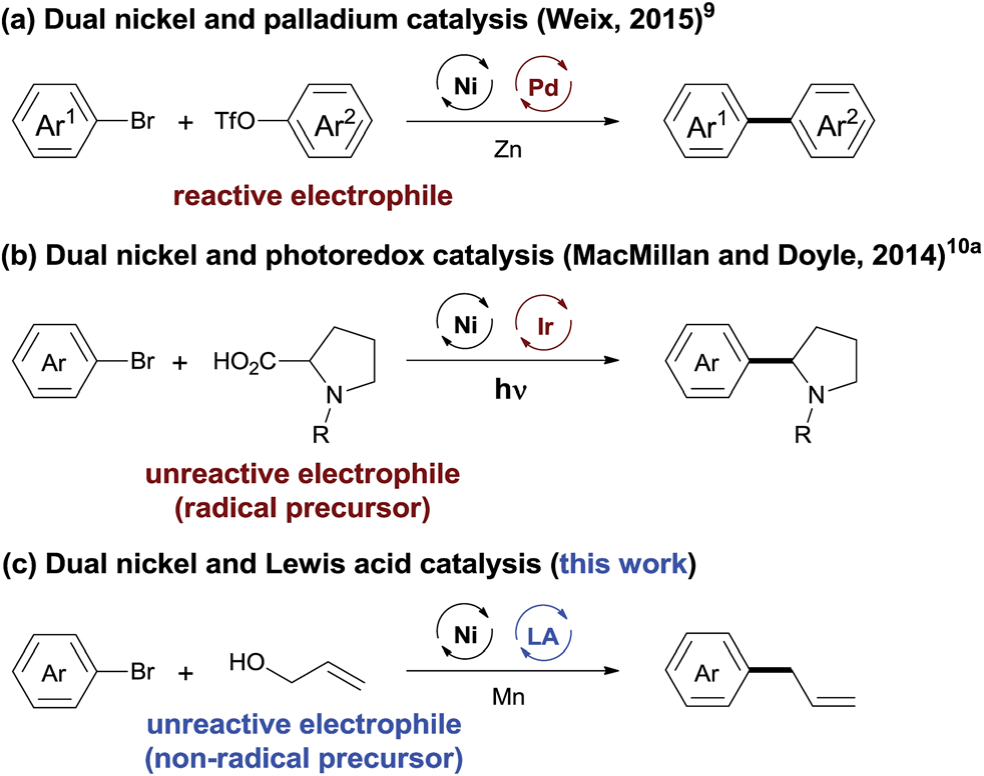
Dual catalysis to address the selectivity challenge in cross-electrophile coupling.

The integration of multiple catalytic systems to enhance reactivity and/or selectivity is a concept often employed by biological catalysts^7^ and is an emerging strategy in molecular catalysis.^8^ The cooperative effects offer an opportunity to overcome the selectivity challenge in cross-electrophile coupling reactions (Scheme 1). Recently, Weix has reported dual nickel and palladium catalysis, achieving the cross-coupling of two reactive electrophiles (Ar–Br + Ar–OTf).^9^ MacMillan and Doyle demonstrated that cooperative nickel and photoredox catalysis enabled the coupling between reactive and unreactive electrophiles (aryl/alkyl halides + carboxylic acids).^10^ This strategy, while powerful, is less effective when a non-radical precursor is used. We considered that the use of a LA (Lewis acid) will lower the activation energy of the inert C–O bond,^11^ offering an opportunity for the cross-electrophile coupling of unreactive O-electrophiles. We report here a general and practical strategy for cross-electrophile coupling between reactive and unreactive electrophiles by a combination of nickel and LA catalysis (Scheme 1c). The strategy was applied for the allylation of aryl halides with allylic alcohols to afford allylarenes.

Allylarenes are ubiquitous motifs in various biologically active natural products.^12^ The precise synthesis of these compounds has been achieved by the coupling reaction of aryl halides with allyl metals,^13^ the allylation reaction of aryl metals with allylic substrates,^14^ and the reductive allylation reaction of Ar–Br with allylic acetate.^15^ These reactions, while powerful, require the pre-activation of at least one reactant. In terms of the atom- and step-economy, the synthesis of allylarenes directly from non-activated precursors will be much more attractive, but remains a particular challenge due to: (1) the selectivity for the allylation of electrophiles (Ar–Br) over nucleophiles (C–OH)^16^ and (2) the selectivity for the reaction of Ar–Br with C–OH over C=C bonds.^17^ In this article, we demonstrated such a convenient synthetic route for the selective synthesis of allylarenes from Ar–Br and allylic alcohols.

## Results and discussion

We began our investigation by exploring the reaction of 4-bromotoluene **1aa** with cinnamyl alcohol **2a**.^18^ The Mizoroki–Heck reaction product was not observed under reductive conditions. In the absence of a LA, a significant amount of biaryl dimer was obtained, but no cross-product or trace of cross-product **3** were observed (Table S1†). The addition of a catalytic amount of LA significantly improved the selectivity for **3** (Table S2†). Further optimization of the reaction conditions revealed that the use of Ni(dppp)Cl_2_ (10 mol%), bpy (20 mol%), ZrCl_4_ (10 mol%), and Mn (3.0 equiv.) in DMA afforded **3** in 85% yield.^19^

**Scheme 2:**
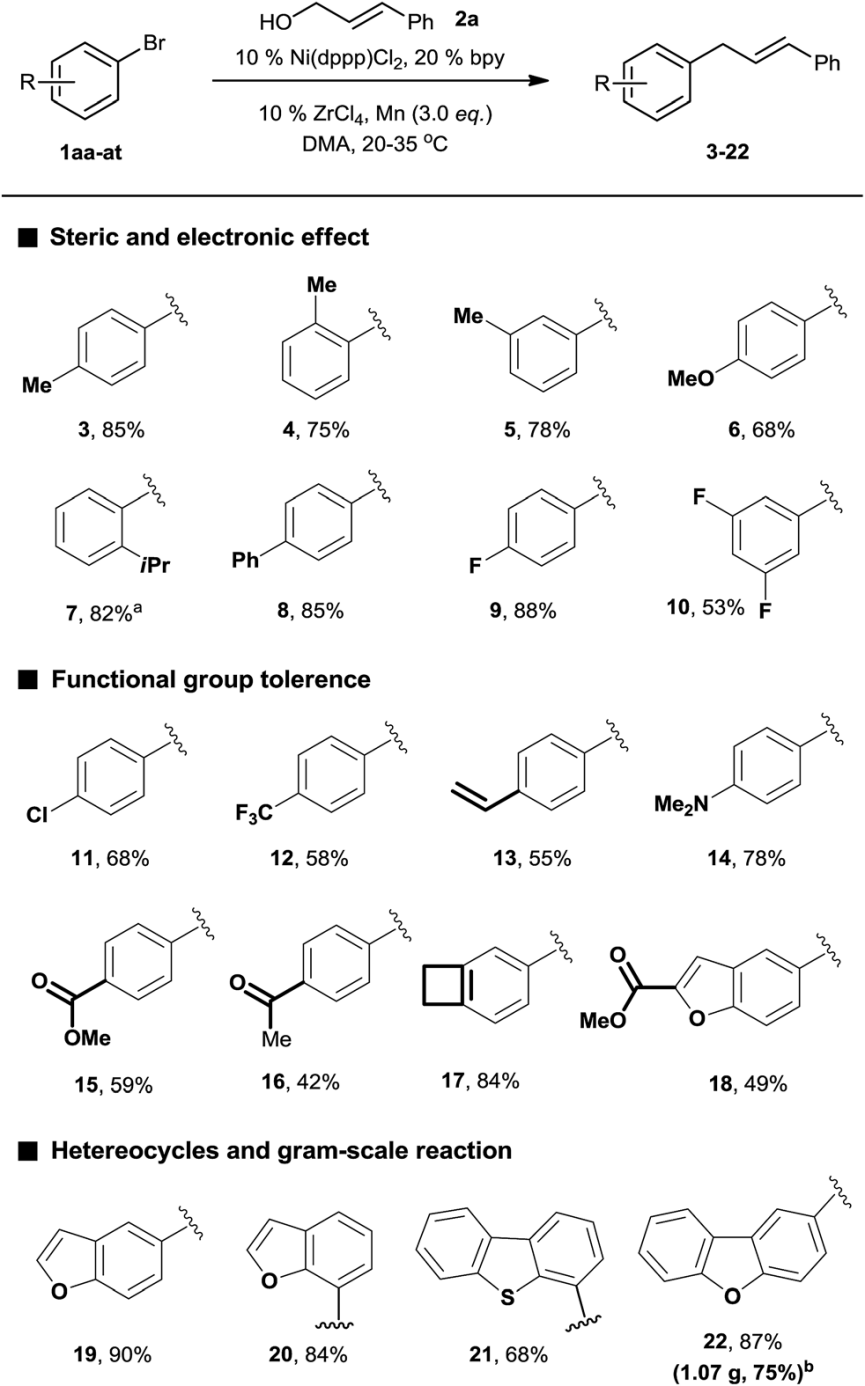
The scope of aryl bromides. All the data are the averages of the two experiments. **1aa-at** (1.5 equiv.) and **2a** (1 equiv.) were used. The reactions were for 32–48 h. The yields are the isolated yields. ^a^Catalyst: 10% Ni(diglyme)Br_2_. ^b^95 h.

With the optimized conditions in hand, we then investigated the reaction scope of aryl bromides. Both the electron-rich and electron-poor aryl bromides gave cross-coupling products in moderate to good yields (Scheme 2, **3–10**). Substitution around the aromatic ring was tolerated (**3–5**). Sterically hindered substitution was tolerated when Ni(diglyme)Br_2_ was used as a catalyst (**7**). The reaction was selective for the functionalization of the C–Br bond over the C–Cl and C–F bonds and styrenyl groups, thus enabling later availability for additional transformation (**9–13**). The functional groups such as tertiary amines, esters, and ketones, as well as a strained ring, were accommodated and remained intact (**14–18**). Heteroarenes and polyarenes, which are prevalent in pharmaceuticals, can be precisely allylated (**18–22**). The reaction could be scaled up to gram scale and gave **22** in a 75% yield.

**Scheme 3:**
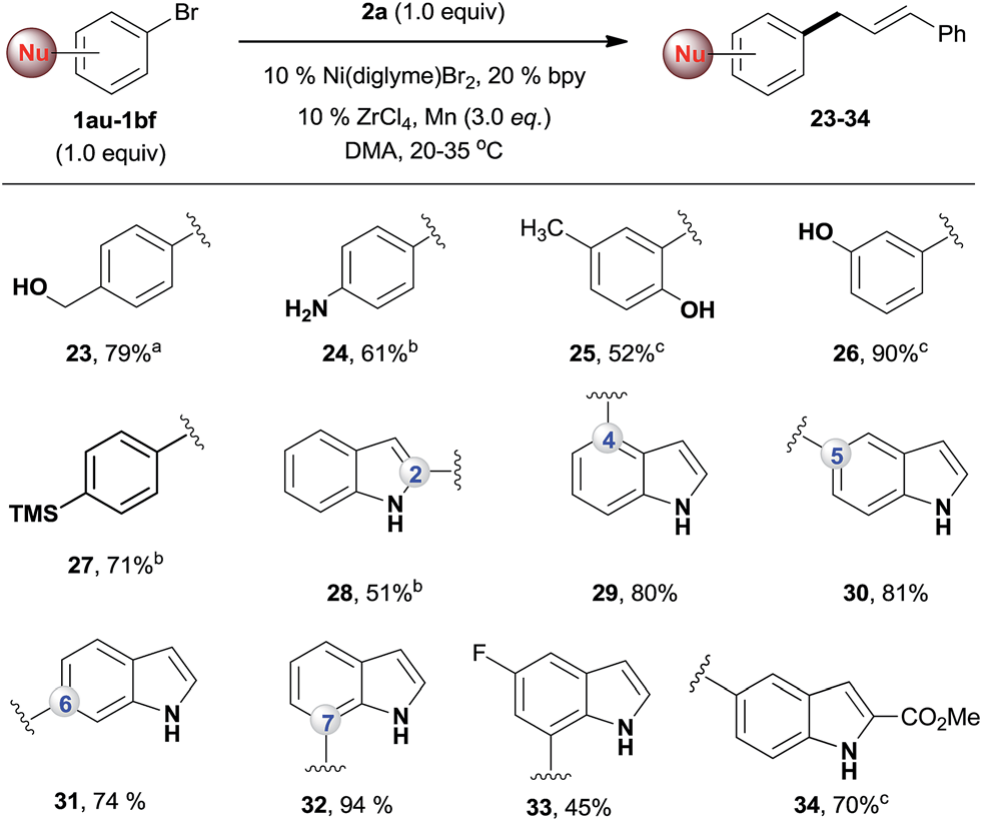
The reactions of **2a** with nucleophilic aryl bromides. All the data are the averages of the two experiments. The yields are the isolated yields. The reactions were for 32–48 h. ^a^The conditions in Scheme 2, reaction for 60 h. ^b^The conditions in Scheme 2, amount of ArBr: 1.0 equiv. ^c^Amount of ArBr: 1.5 equiv.

While the existing allylation reactions of the allylic alcohols are efficient for allylating nucleophiles,^14^ this reaction is highly selective for electrophiles. A broad range of nucleophilic aryl bromides were then selectively allylated, leaving the alcohol,^20^ amine,^21^ phenol,^22^ indole^23^ and silane groups intact (Scheme 3). The utility of this method was illustrated by a regiodivergent synthesis of allylated indoles, which are important structural motifs in many bioactive natural products and drugs.^24^ Being complementary to conventional methods for the allylation of indoles at the C3 position,^23^ our approach provides direct access to C2-, C4-, C5-, C6-, and C7-allylated indoles (**28–34**). Of note, the desired products were obtained in high yields even when an equal amount of the two electrophiles was used.

The reaction proceeded efficiently with a variety of allylic alcohols and in most cases gave the linear (*E*)-products selectively (Table 1). In addition to the cinnamyl alcohols, unsubstituted and alkyl-substituted allylic alcohols also coupled with a functionalized aryl group (entries 1–5). Although the reaction with α-ethyl substituted alcohol is less selective, the substrate with phenyl substitution gave only the linear (*E*)-product (entries 6 and 7).

**Table 1:** The scope of allylic alcohols^*a*^

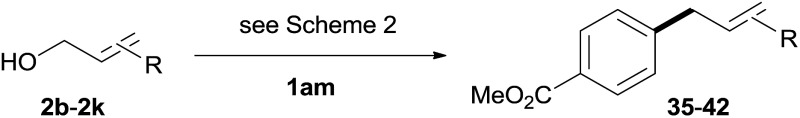			
Entry	Allylic alcohol	Product	Yield
1	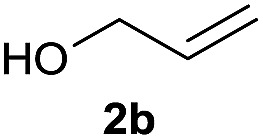	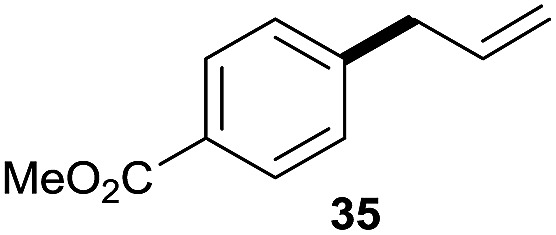	80%
2	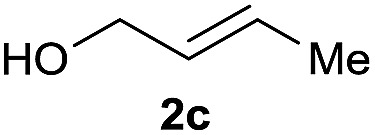	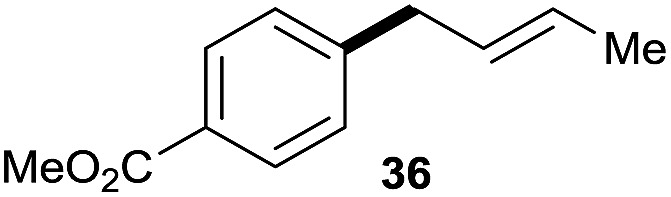	62%^*b*^ ^,^^*c*^
3	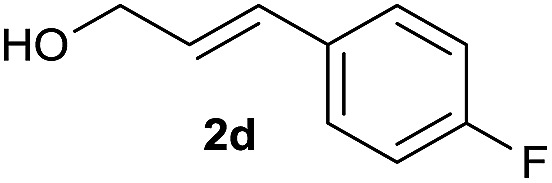	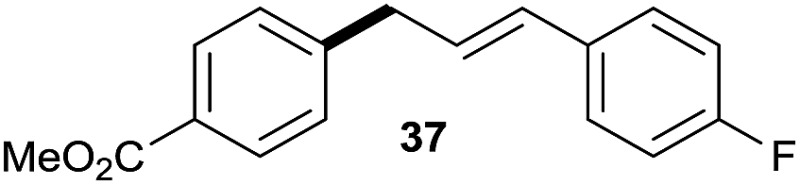	75%
4	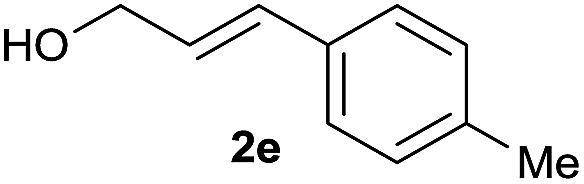	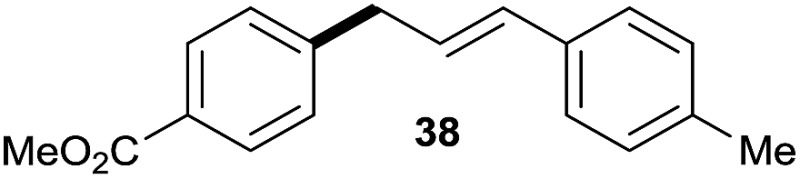	73%
5	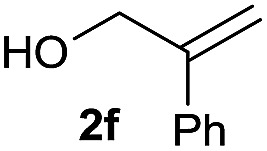	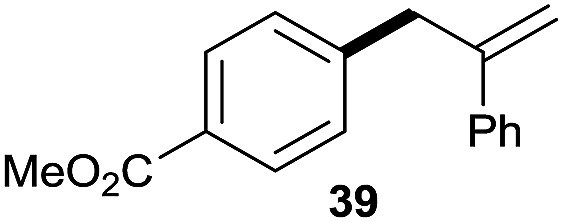	58%
6	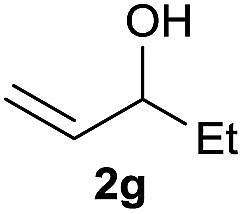	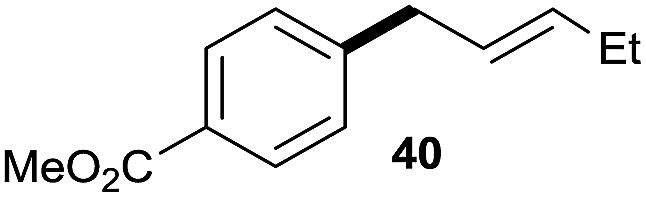	63%^*b*^ ^,^^*d*^
7	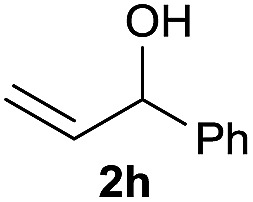	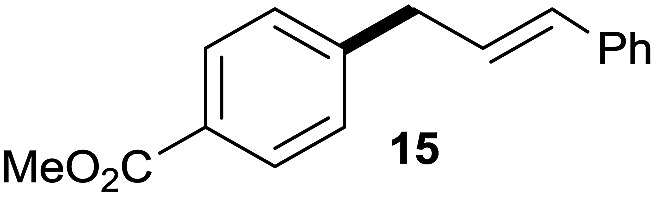	76%
8	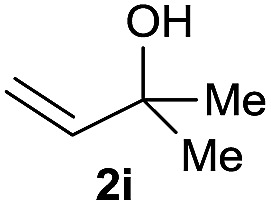	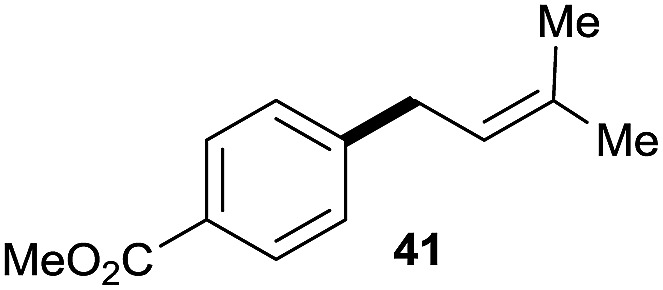	66%^*b*^ ^,^^*e*^
9	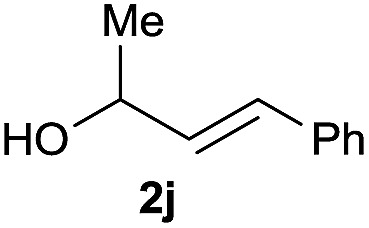	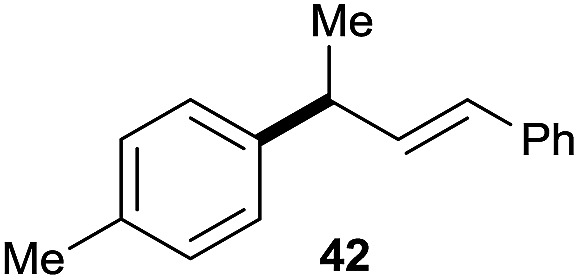	56%^*f*^
10	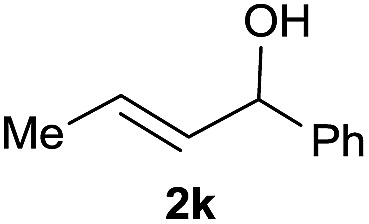	42	60%

^*a*^All the data are the averages of the two experiments. The yields are the isolated yields. The reactions were for 48–50 h. Amount of **1am**: 2.0 equiv. **1aa** were used for **2j** and **2k**.

^*b*^Catalyst: 10% Ni(dppf)Cl_2_, 20% 3,4,7,8-tetramethyl-1,10-phenanthroline, 20% AlCl_3_, **1am** (1.5 equiv.).

^*c*^[l/b] (linear/branched ratio) = 4 : 1, linear product is a 3 : 1 *E*/*Z* isomer.

^*d*^[l/b] = 11 : 1, linear product is a 5 : 1 *E*/*Z* isomer.

^*e*^[l/b] = 180 : 1.

^*f*^15% catalysts were used.



1

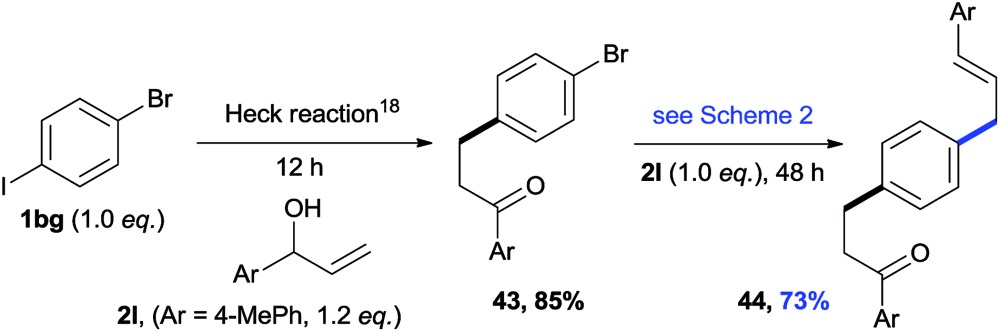



2

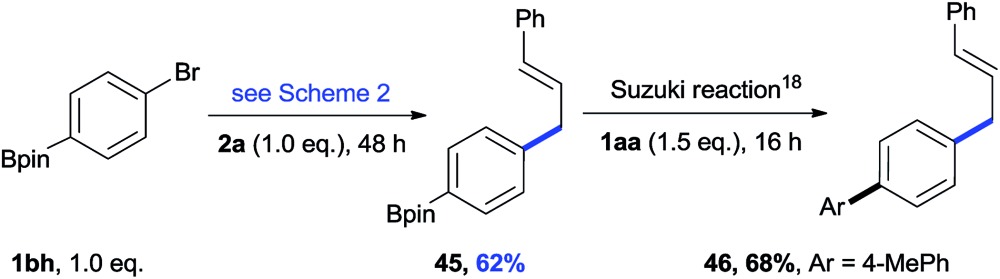

Prenylated arenes have been found in many bioactive compounds, and their syntheses have been extensively studied but require prenyl-metal species (allylfluorosilanes, allylstannanes, allylboron derivatives, *etc.*).^13^ Such a structure, however, could be efficiently constructed here from isoprenyl alcohol with a linear/branched ratio of 180 : 1 (entry 8). Both nonsymmetrical substrates **2j** and **2k** gave the same product **42** in useful yields, suggesting that the reaction goes through a π-allyl nickel intermediate.

The cross-electrophile coupling reaction complements the Pd-catalyzed reactions well, enabling 1-bromo-4-iodobenzene to be sequentially functionalized with allylic alcohol **2l** to the ketone **43** and allylated product **44** (eqn (1)). Our reaction conditions were also compatible with aryl borates and a sequence of C–Br bond allylation and C–B bond coupling is possible for the conversion of substrate **1bh** to product **46** (eqn (2)).

The LA was expected to lower the activation energy of the C–OH bond. To confirm this assumption and understand the mechanistic details of this process, we monitored the reaction of **1aa** with **2j** by GC analysis. In the absence of a LA, no cross-coupling product **42** and side products from **2j** were observed in 40 min, but the aryl dimer **51** was steadily increasing (Fig. S1a†). This result indicates that aryl bromide is reactive towards the Ni catalyst, while the allylic alcohol is inert. The use of 15 mol% of ZrCl_4_ significantly promoted the cross-coupling process, indicating the crucial role of the LA in the activation of allylic alcohols (Fig. S1b†).

**Scheme 4:**
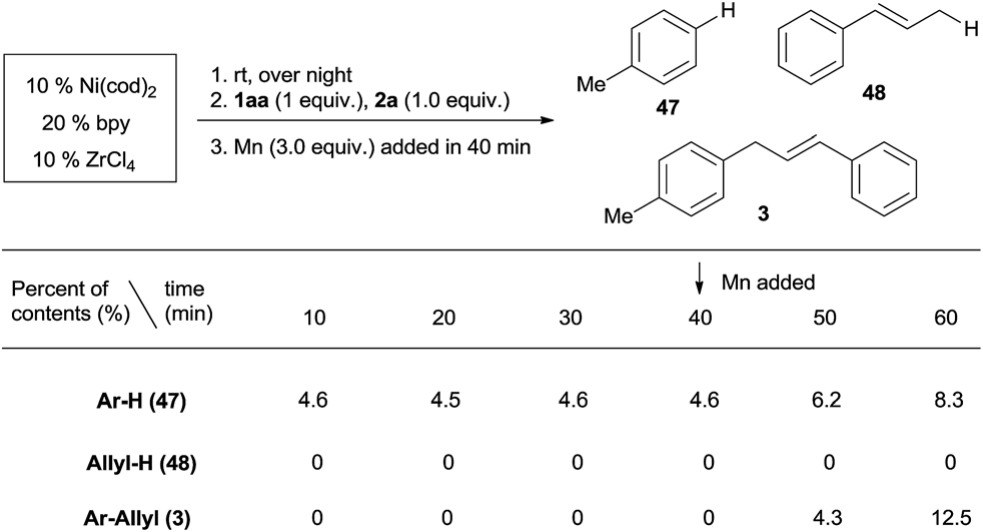
The selectivity of Ar–Br and the allylic alcohol in the initial oxidative addition to Ni(0). A mixture of **1aa**/**2a** (1 : 1) was added to an *in situ* generated Ni(0) catalytic system. Samples were collected every 10 min and analysed by GC. Mn was added at 40 min.

Both aryl bromide and allylic alcohol will undergo oxidative addition to Ni(0), generating Ar–Ni^II^(L)Br and allyl-Ni^II^(L)X.16a,b,21b In order to determine which intermediate is formed first, we studied the relative reactivity of **1aa** and **2a** with *in situ* generated (bpy)Ni^0^(cod).^25^ We would expect, by quenching the reaction, a Ni(ii) intermediate would afford Ar–H (**47**) or allyl-H (**48**).^26^Scheme 4 shows that, in every 10 min before the addition of Mn, 4.6% of Ar–H is obtained in each sample but no allyl-H is observed, consistent with Ar–Ni^II^(L)Br forming first. While both cross-product **3** and the Ar–Ar dimer were steadily increasing after the addition of Mn (3 equiv.), allyl-H or allyl–allyl were still not observed (Scheme S1†), suggesting a mechanism in which Ar–Ni^II^(L)Br serves as an intermediate.

**Fig. 1:**
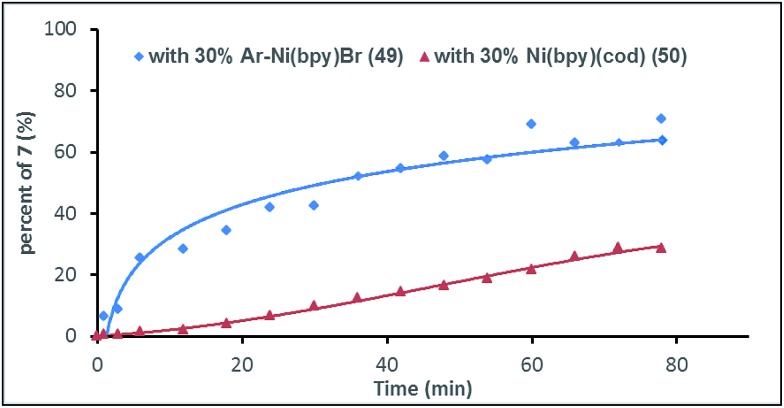
The relative reactivity of Ar–Ni^II^(bpy)Br (**49**) and (bpy)Ni^0^(cod) (**50**) capable of catalysing the reaction of **1ae** with **2a**. Complex **49** or **50** (30 mol%) was added to a solution of **1ae** (1.2 or 1.5 equiv.), **2a** (1.0 equiv.), bpy (30 mol%), ZrCl_4_ (10 mol%) and Mn (3.0 equiv.) in DMA. Samples were collected, quenched with H_2_O, and analysed by GC.

To gain more insight into the mechanism of this process, complex Ar–Ni^II^(bpy)Br (**49**)^27^ was synthesized from the reaction of (bpy)Ni^0^(cod) (**50**) with aryl bromide **1ae**. We would expect that, if Ar–Ni^II^(bpy)Br (**49**) is an intermediate, the initial rate of reaction with complex **49** would be faster than that of the reaction with pre-catalyst **50**. Fig. 1 shows that, in 6 min, the use of complex **49** (30 mol%) gave product **7** in almost 30% yield, while only a trace of **7** is formed when pre-catalyst **50** (30 mol%) is used (Fig. 1). This result further indicates that Ar–Ni^II^(L)Br is likely the key intermediate in this reaction.

The reduction of Ni(ii) complex to Ni(i) by Mn has been extensively reported.4a,b The stoichiometric reactions of **49** with alcohol **2a** gave product **7** in 0% yield (without Mn) and 90% yield (with Mn), suggesting that Ni(ii) complex **49** might be reduced to the more nucleophilic ArNi(i) first, then react with the allylic alcohols (eqn (3)). The use of electron-poor aryl bromides (*e.g.***18***vs.***19** and **32***vs.***33**) generally gave inferior results, which is consistent with this process. While the use of a radical scavenger, such as butylated hydroxytoluene (BHT), hydroquinone, and 1,1-diphenylethylene, did not inhibit the reaction of **1aa** and **2a**, no product **3** was obtained when 2,2,6,6-tetramethyl-1-piperidinyloxy (TEMPO, 1.5 equiv.) was employed (Table S8†), consistent with the presence of a single-electron process capable of reducing oxidized Ni catalyst.
3

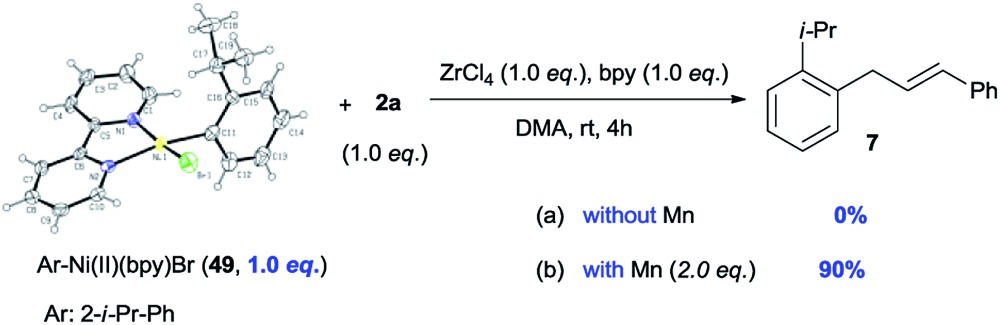




**Scheme 5:**
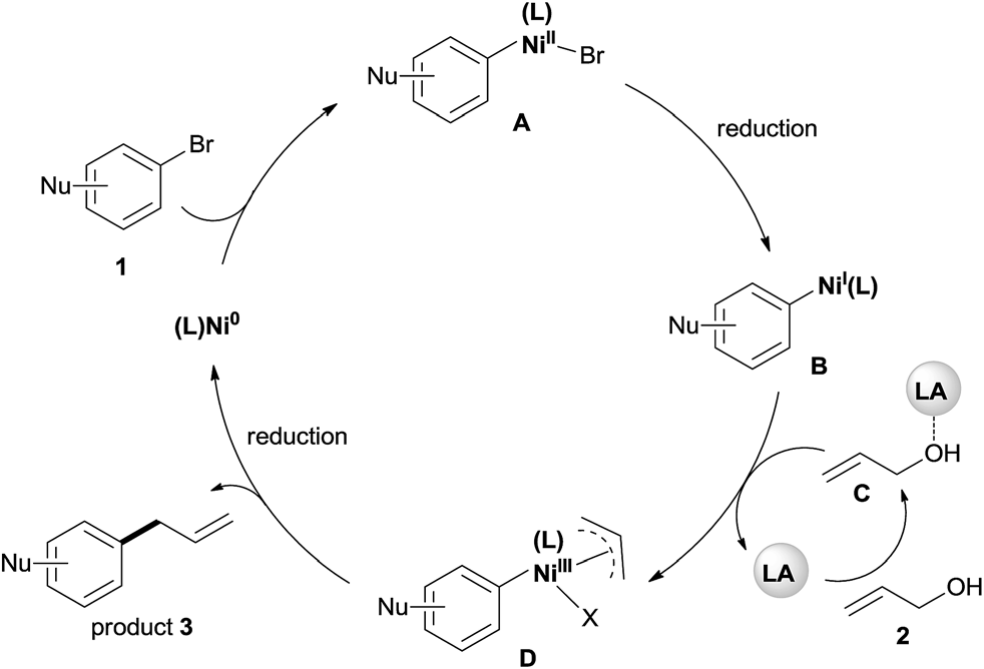
The proposed mechanism for the allylation of aryl bromides with allylic alcohols by dual catalysis.

Although a detailed mechanistic picture for this reaction requires further investigation, based on the above results, we tentatively proposed a catalytic cycle, as shown in Scheme 5.^28^ The oxidative addition of Ar–Br to Ni(0) gives the arylnickel(ii) intermediate **A**. The reduction of **A** to arylnickel(i) **B**4a,b followed by the oxidative addition of LA-activated allylic alcohol will give the π-allylnickel(iii) intermediate **D**.^29^ The reductive elimination of this complex will afford the desired product and recycle the catalyst with Mn. At present, we cannot rule out a radical mechanism as shown in the reaction of aryl halides with alkyl halides.^30^

## Conclusions

In summary, we have reported a dual nickel and Lewis acid-catalyzed allylation of aryl halides with allylic alcohols. This approach represents a new strategy for cross-electrophile coupling between reactive and unreactive electrophiles. The reaction tolerated a wide range of functional groups including alcohols, phenols, anilines, silanes, and even borates. In most cases, the reaction gave linear (*E*)-allylarenes highly selectively. The utility of this method is clearly illustrated by the facile access to C2 and C4–C7 allylated indoles. Preliminary mechanistic studies revealed that the reaction might start with an aryl nickel intermediate, which then reacted with allylic alcohols in the presence of a Lewis acid and reductant. The application of this strategy for the cross-electrophile coupling of other unreactive electrophiles is ongoing in our laboratory.

## Conflicts of interest

There are no conflicts to declare.

## Supplementary Material

Supplementary information

Crystal structure data
