# Supplementary material for: Dual nickel and Lewis acid catalysis for cross-electrophile coupling: the allylation of aryl halides with allylic alcohols
Source: Chem Sci. 2017 Nov 6;9(3):640–5. doi: 10.1039/c7sc03140h (PMC5868389; doi:10.1039/c7sc03140h)
Supplement: Supplementary file 1 [file SC-009-C7SC03140H-s001.pdf]

Electronic Supplementary Material (ESI) for Chemical Science.
This journal is © The Royal Society of Chemistry 2017

Supporting Information

Dual Nickel and Lewis Acid Catalysis for Cross-Electrophile Coupling: Allylation of Aryl Halides with Allylic Alcohols

Xue-Gong Jia, Peng Guo, Jicheng Duan, Xing-Zhong Shu*

State Key Laboratory of Applied Organic Chemistry (SKLAOC), College of Chemistry and Chemical Engineering, Lanzhou University, 222 South Tianshui Road, Lanzhou, 730000, China.

Table of contents:

1. General Information.. S2

2. Optimization of Reaction Conditions S3-S9

3. Mechanistic Investigation S10-S102

4. Preparation of Allylic Alcohols……………………………...S13-S16

5. Allylation Reactions of Aryl Bromides with Allylic Alcohols................S17-S39

6. Sequence Reactions in Eq.1 and 2................................S40-S42

7. Monitoring of the Reaction of 1aa and 2j by GC Analysis…………………….S43

8. Procedure for experiments in Scheme 4 .... S43

9. Synthesis and the Reactions of Ar-Ni^{II}(bpy)Br 49 and (bpy)Ni^{0}(cod) 50........................S44-S50

10. Reference S51

11. Copies of ^{1}H and ^{13}C NMR Spectra for New Compounds…….……………………..S52-S111

1. General Information

Reagents and solvents:

Ni(dppp)Cl_2 (Energy chemical), Ni(diglyme)Br_2 (Aldrich), Ni(dppf)Cl_2 (9dingchem), $ZrCl_4$ (Energy chemical), $AlCl_3$ (Alfa), manganese powder (-140+350 mesh, Alfa), 2,2'-bipyridine (Energy chemical), 3,4,7,8-tetramethyl-1,10-phenanthroline (Ark) were used as received. Other nickel catalysts, reductant, Lewis acids, ligands tested were from commercial suppliers and used as received.

All aryl bromides, allylic alcohols **2a-c**, **2g**, **2i** were purchased (Admas, Energy chemical, Ark, TCI) and used as received. Other known starting materials (**2d**, **2e**, **2f**, **2h**, **2j**, **2k**, **2l**) were prepared according to the literature procedures and were referenced.

Anhydrous N,N-dimethylformamide (DMF), N,N-dimethylacetamide (DMA), toluene, Et_2O, THF were purified using a solvent-purification system that contained activated alumina and molecular sieves. Other solvents were dried and purified according to the procedure from "Purification of Laboratory Chemicals book".[1]

Analytical methods:

^{1}H and ^{13}C NMR spectra were collected with Bruker AVANCE III 400 MHz and Agilent-NMR-inova 600 MHz spectrometers at ambient temperature and were referenced to the signal of tetramethylsilane (TMS, 0 ppm) for ^{1}H NMR spectra and the signal of chloroform ($CHCl_3$, center line: $\delta = 77.2$ ppm) for ^{13}C NMR spectra. **^{19}F NMR** spectra was collected with Bruker AVANCE III 400 MHz spectrometers at ambient temperature. **^{11}B NMR** spectra was collected with Ailent-NMR-inova 600 MHz spectrometers at ambient temperature.

HRMS was performed on Bruker Apex II FT-ICR mass instrument (ESI) and waters GCT Premier TOFMS (EI).

GC-MS data was collected on Thermo Scientific TRACE DSQ GC-MS.

GC analysis was performed on Thermo Scientific TRACE 1300.

IR spectra were collected with Bruker-TENSOR27 spectrometer and only major peaks were reported in cm^{-1}.

X-Ray analysis was performed on a Bruker SuperNova, Dual, Cu at zero, Eos diffractometer.

TLC analysis of the reaction was performed on XINNUO SGF254 TLC plates using UV light, potassium permanganate stain, and I_2 to visualize the reaction components.

Flash chromatography was carried out on XINNUO silica gel (particle size 200-300 mesh) according to standard procedures.

2. Optimization of Reaction Conditions

2.1 Controlled Experiments

We began the investigation by exploring the reaction of aryl bromide **1aa** with allylic alcohol **2a** under reductive conditions. In the absence of a Lewis acid, aryl bromide was dimerized to **51,** however only trace amount of allylated product **3** was formed in 4 h (Table S1, entry 1). The formation of product **3** is proposed to be catalyzed by in situ generated $MnBr_2$ as a Lewis acid. Indeed, addition of 10 mol % of $MnBr_2$ gave **3** in 18% yield (Table S2, entry 12). Further, the allylated product **42** was not formed until the reaction of **1aa** and **2j** proceeded for 40 min in the absence of Lewis acid (Figure S1). No reaction was observed in the absence of Ni catalyst, Mn reductant or bpy ligand (Table S1, entries 2-4). The addition of catalytic amount of $BF_3 \cdot Et_2O$ as a Lewis acid significantly improved the selectivity for the formation of product **3** (Table S1, entry 5).

Table S1. Controlled experiments.[a]

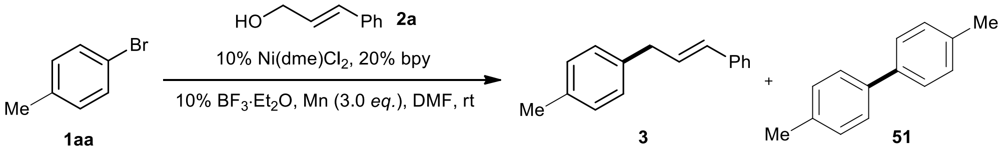

entry	reaction conditions	yield of **3** (%)	yield of **51** (%)
1	No $BF_3 \cdot Et_2O$	4	40
2	No Ni catalyst	0	0
3	No Mn as reductant	0	0
4	No bpy as ligand	0	0
**5**	**as shown**	**43**	**20**

[a] Reaction conditions: 4-bromotoluene **1aa** (0.2 mmol, 1.0 *eq.*), cinnamyl alcohol **2a** (0.2 mmol, 1.0 *eq.*), $Ni(dme)Cl_2$ (10 mol %), bpy (20 mol %), $BF_3 \cdot Et_2O$ (10 mol %) and Mn (3 *eq.*) in DMF (2.0 mL) was stirred at room temperature for 4 h. Yields were determined by GC analysis with dodecane as internal standard.

2.2 Effect of Lewis acids

The allylation reaction of **1aa** with **2a** was promoted with a broad range of Lewis acids, as is shown in Table S2. Among which, $ZrCl_4$ gave the best result, affording **3** in 55% yield (Table S2, entry 9).

Table S2. Effect of various Lewis acids.[a]

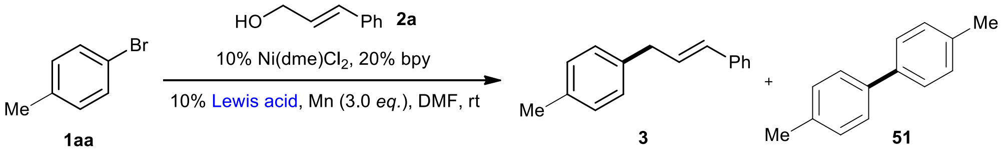

entry	Lewis acids	yield of **3** (%)	yield of **51** (%)
1	BPh_3	37	17
2	$B(C_6F_5)_3$	24	37
3	BEt_3	18	25
4	LiOTf	10	28
5	$Bi(OTf)_3$	0	0
6	$BiCl_3$	5	3
7	$Mg(OTf)_2$	13	26
8	$Al(OTf)_3$	16	27
**9**	**$ZrCl_4$**	**55**	**22**
10	$Sc(OTf)_3$	47	20
11	$Hf(OTf)_4$	34	30
12	$MnBr_2$	18	37
13	$AlCl_3$	17	30

[a] Reaction conditions: 4-bromotoluene **1aa** (0.2 mmol, 1.0 *eq.*), cinnamyl alcohol **2a** (0.2 mmol, 1.0 *eq.*), $Ni(dme)Cl_2$ (10 mol %), bpy (20 mol %), Lewis acid (10 mol %) and Mn (3 *eq.*) in DMF (2.0 mL) was stirred at room temperature for 16 h. Yields were determined by GC analysis with dodecane as internal standard.

2.3 Effect of solvents

The reaction proceeded in highly polar aprotic solvents and the best result was obtained when DMA (Dimethylacetamide) was used (Table S3, entries 1-5). The dilution of DMA either with THF or toluene gave poor results and this indicates that the strong LA may not act as the “buffering” effect of the Lewis basic solvent DMA (entries 6-11). No reaction was observed in CH_3CN, toluene, dioxane, DCE and THF (entries 12-16).

Table S3. Effect of solvents.[a]

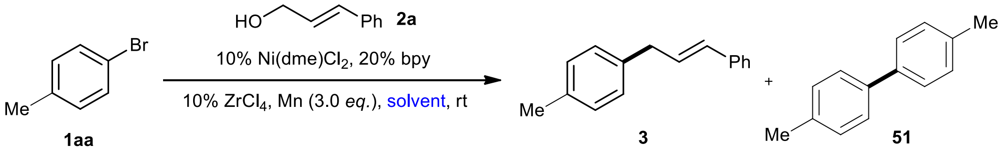

entry	solvent	yield of **3** (%)	yield of **51** (%)
1	DMSO	16	23
2	DMPU	3	4
3	DMI	4	10
4	DMF	55	22
**5**	**DMA**	**59**	**14**
6	DMA/THF = 3:1	21	38
7	DMA/THF = 1:1	9	44
8	DMA/THF = 1:3	15	29
9	DMA/toluene =3:1	11	30
10	DMA/toluene = 1:1	21	28
11	DMA/toluene = 1:3	5	4
12	CH_3CN	0	0
13	toluene	0	0
14	dioxane	0	0
15	DCE	0	0
16	THF	0	0

[a] Reaction conditions: 4-bromotoluene **1aa** (0.2 mmol, 1.0 *eq.*), cinnamyl alcohol **2a** (0.2 mmol, 1.0 *eq.*), $Ni(dme)Cl_2$ (10 mol %), bpy (20 mol %), $ZrCl_4$ (10 mol %) and Mn (3 *eq.*) in solvent (2.0 mL) was stirred at room temperature for 16 h. Yields were determined by GC analysis with dodecane as internal standard.

2.4 Effect of nickel catalysts

The investigation of nickel sources revealed that the use of Ni(dppp)Cl_2 as a catalyst gave the desired product **3** in 85% yield with 2% yield of dimer **51** (Table S4, entry 9). Comparable results were obtained when Ni(diglyme)Br_2 and Ni(dppf)Cl_2 were used (entries 8 and 10).

Table S4. Effect of various Ni catalysts.[a]

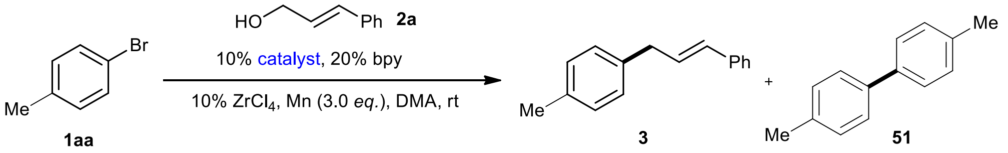

entry	Ni catalyst	yield of **3** (%)	yield of **51** (%)
1	NiF_2	0	0
2	$NiCl_2$	44	20
3	$NiBr_2$	50	13
4	NiI_2	68	8
5	$NiCO_3$	0	0
6	Ni(acac)$_2$	56	2
7	Ni(PPh_3)$_2$$Cl_2$	67	6
**8**	**Ni(diglyme)Br_2**	**80**	**6**
**9**[b]	**Ni(dppp)Cl_2**	**85**	**2**
**10**[b]	**Ni(dppf)Cl_2**	**80**	**5**
11[b]	Ni(dppe)Cl_2	46	3
12	Ni(cod)$_2$	73	6

[a] Reaction conditions: 4-bromotoluene **1aa** (0.2 mmol, 1.0 *eq.*), cinnamyl alcohol **2a** (0.2 mmol, 1.0 *eq.*), Ni catalyst (10 mol %), bpy (20 mol %), $ZrCl_4$ (10 mol %) and Mn (3 *eq.*) in DMA (2.0 mL) was stirred at room temperature for 16 h. Yields were determined by GC analysis with dodecane as internal standard. [b] Reactions for 32 h.

2.5 Effect of ligands

Table S5. Effect of ligands.[a]

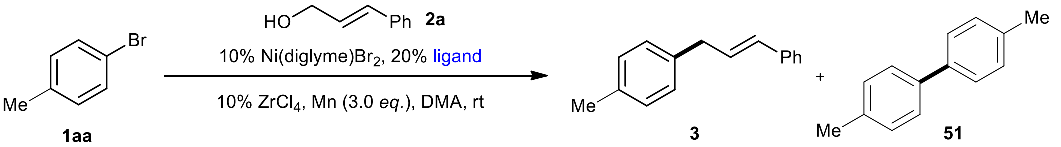

1aa 3 51

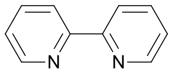

L1, 80%, 6%

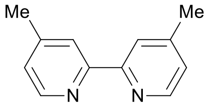

L2, 30%, 8%

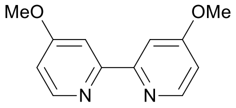

L3, 21%, 25%

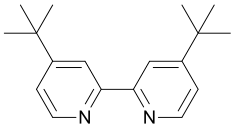

L4, 44%, 24%

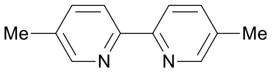

L5, 29%, 22%

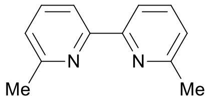

L6, 6%, 0%

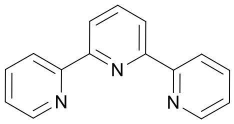

L7, 0%, 0%

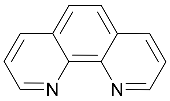

L8, 22%, 8%

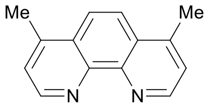

L9, 13%, 0%

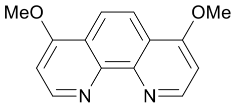

L10, 18%, 14%

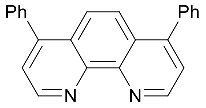

L11, 65%, 8%

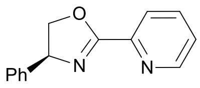

L12, 29%, 28%

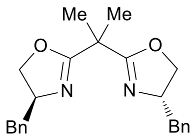

L13, 9%, 4%

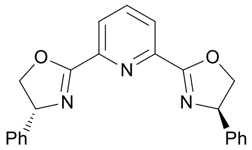

L14, 11%, 1%

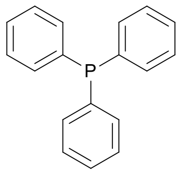

L15, 4%, 0%

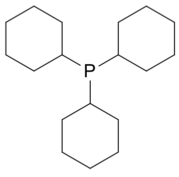

L16, 0%, 0%

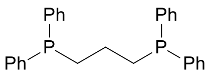

L17, 0%, 0%

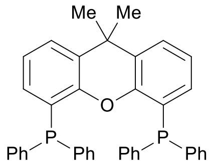

L18, 0%, 0%

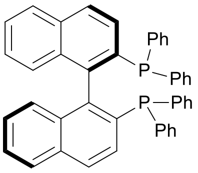

L19, 6%, 0%

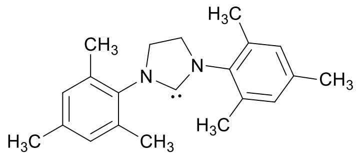

L20, 0%, 0%

[a] Reaction conditions: 4-bromotoluene **1aa** (0.2 mmol, 1.0 *eq.*), cinnamyl alcohol **2a** (0.2 mmol, 1.0 *eq.*), Ni(diglyme)Br_2 (10 mol %), ligand (20 mol %), $ZrCl_4$ (10 mol %) and Mn (3 *eq.*) in DMA (2.0 mL) was stirred at room temperature for 16 h. Yields were determined by GC analysis with dodecane as internal standard. Yields of **3** and **51** were given.

2.6 Effect of reaction temperature and the ratio of 1aa to 2a

With the increase of reaction temperature, the formation of product **3** was decreased, while the formation of dimer **51** was steadily increased (Table S6, entries 1-5). Product **3** was isolated in 80% yield when 1.5 equiv. of aryl bromide **1aa** was used (entry 6). A comparable result was also obtained when Ni(dppp)Cl_2 was used as a catalyst (entry 7). As the applicability of this catalyst is more general than Ni(diglyme)Br_2 for reactions in Scheme 2, conditions in entry 7 were then used as standard conditions.

Table S6. Effect of reaction temperature and aryl bromide loading.[a]

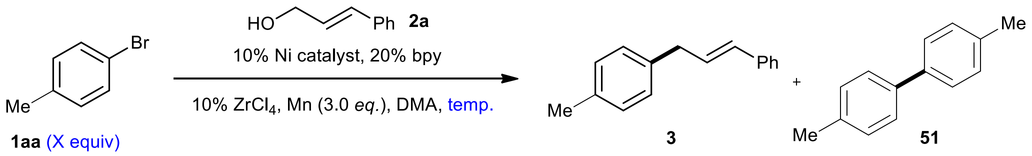

entry	catalyst	X	temp. (°C)	yield of **3** (%)	yield of **51** (%)
1	Ni(diglyme)Br_2	1.0	30	80	6
2	Ni(diglyme)Br_2	1.0	40	60	15
3	Ni(diglyme)Br_2	1.0	60	47	27
4	Ni(diglyme)Br_2	1.0	80	37	30
5	Ni(diglyme)Br_2	1.0	100	32	31
**6**	**Ni(diglyme)Br_2**	**1.5**	**30**	**88 (80)**[b]	**21**
**7**[c]	**Ni(dppp)Cl_2**	**1.5**	**30**	**92 (85)**[b]	**11**
**8**[c]	**Ni(dppf)Cl_2**	**1.5**	**30**	**83**	**25**
9	Ni(diglyme)Br_2	1.8	30	87	22
10	Ni(diglyme)Br_2	2.0	30	68	35

[a] Reaction conditions: 4-bromotoluene **1aa** (X *eq.*), cinnamyl alcohol **2a** (0.2 mmol, 1.0 *eq.*), Ni catalyst (10 mol %), bpy (20 mol %), $ZrCl_4$ (10 mol %) and Mn (3 *eq.*) in DMA (2.0 mL) was stirred at listed temperature for 16 h. Yields were determined by GC analysis with dodecane as internal standard. [b] Isolated yield. [c] Reactions for 32 h.

2.7 Investigation of other aryl electrophiles and reductants

The reaction is less effective when either aryl chloride or iodide was used (Table S7, entries 1-2). Reaction with O-electrophile Ar-OMs did not give any desired product (Table S7, entry 3). The use of Zn or Mg as a reductant gave product **3** in less than 20% yield (entries 4-5).

Table S7. Effect of other aryl electrophiles and reductants

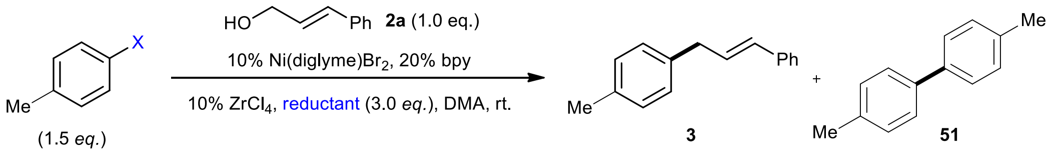

entry	X	reductant	yield of **3** (%)	yield of **51** (%)
1	Cl	Mn	5	0
2	I	Mn	13	53
3	OMs	Mn	0	0
4	Br	Zn	19	5
5	Br	Mg	13	6

[a] Reaction conditions: Ar-X (1.5 *eq.*), cinnamyl alcohol **2a** (0.2 mmol, 1.0 *eq.*), Ni(diglyme)Br_2 (10 mol %), bpy (20 mol %), $ZrCl_4$ (10 mol %) and reductant (3 *eq.*) in DMA (2.0 mL) was stirred at room temperature for 16 h. Yields were determined by GC analysis with dodecane as internal standard.

3. Mechanistic Investigation

3.1 Monitoring of the reaction of 1aa with 2j

Reactions of **1aa** with **2j** were monitored by GC analysis in the absence (Figure S1a) or presence (Figure S1b) of a Lewis acid. Without Lewis acid, the formation of dimer **51** was steadily increased after 20 min, while only trace amount of allylated product **42** was formed after 40 min. No dehydroxylation product allyl-H **52** was observed in 150 min. This result indicates that aryl bromide is reactive towards Ni catalyst, while allylic alcohol is inert. The formation of trace amount of product **42** is ascribed to in situ formed $MnBr_2$, which acted as a Lewis acid (Table S2, entry 12).

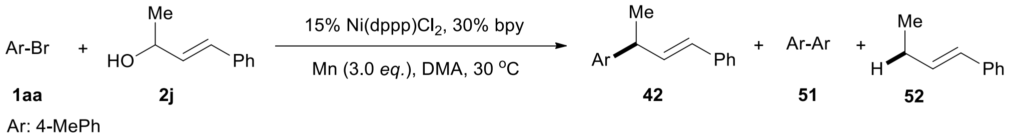

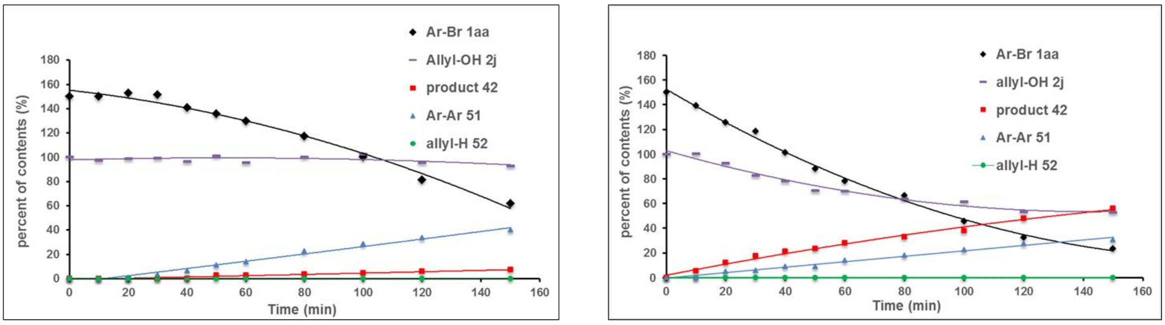

(a) Reaction in the absence of Lewis acid (b) Reaction in the presence of Lewis acid

Figure S1. Monitoring of the reaction of 1aa and 2j

3.2 Selectivity of Ar-Br and allylic alcohol in initial oxidative addition to Ni(0).

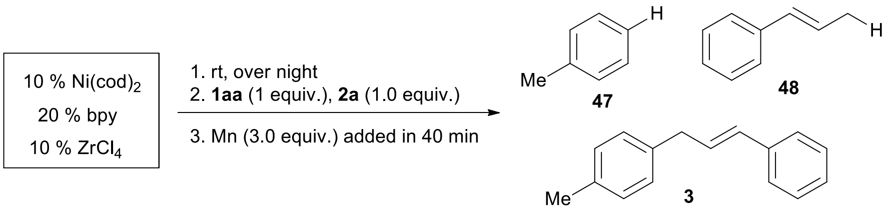

Percent of contents (%) \ time (min)	10	20	30	40 (↓ Mn added)	50	60
**Ar-Br (1aa)**	96	94	95	95	91	78
**Allyl-OH (2a)**	97	99	98	100	95	85
**Ar-H (47)**	4.6	4.5	4.6	4.6	6.2	8.3
**Allyl-H (48)**	0	0	0	0	0	0
**Ar-Allyl (3)**	0	0	0	0	4.3	12.5
**Ar-Ar**	0	0	0	0	1.5	2.4
**Allyl-Allyl**	0	0	0	0	0	0

Scheme S1 Selectivity of Ar-Br and allylic alcohol in initial oxidative addition to Ni(0). A mixture of 1aa/2a (1:1) was added to an in situ generated (bpy)Ni^0(cod). Samples were collected in each 10 min and analysed by GC. Mn was added in 40 min.

3.3 Effect of radical inhibitors

Table S8. Effect of radical inhibitors on the reaction of 1aa with 2a

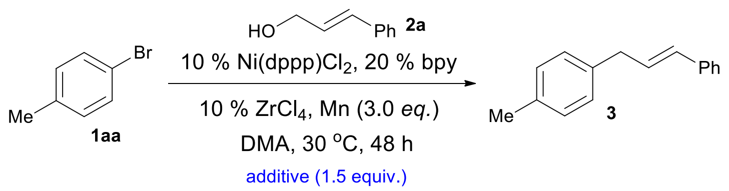

entry	additive	yield of **3** (%)[a]
1	none	92
2	BHT[b]	94
3	hydroquinone	97
4	1,1-diphenylethylene	92
5	TEMPO[c]	0

[a] Yields were GC yields. [b] BHT: butylated hydroxytoluene. [c] TEMPO: 2,2,6,6-Tetramethyl-1-piperidinyloxy.

4. Preparation of Allylic Alcohols

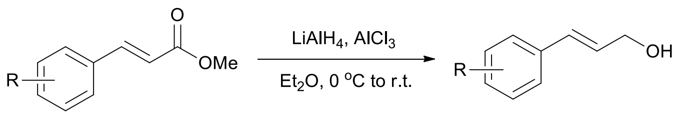

General Procedure A

To a solution of $LiAlH_4$ (532 mg, 14 mmol) in anhydrous Et_2O (40 mL) was slowly added a mixed solution of $AlCl_3$ (528 mg, 4 mol) in Et_2O (8 mL) at 0 ^oC under an argon atmosphere. After stirring at the same temperature for 5 min, a solution of ester (4.0 mmol) in anhydrous Et_2O (20 mL) was added slowly. The reaction mixture was allowed to warm up to room temperature after 30 min and stirred for 1 h. The solution was carefully quenched with 1 M HCl and then neutralized with saturated *aq.* $NaHCO_3$ (50 mL). The mixture was extracted with ethyl acetate (3 × 50 mL). The combined organic layers was washed with saturated brine (40 mL), dried over anhydrous Na_2SO_4 and concentrated under reduced pressure. The crude product was purified by flash chromatography on silica gel.

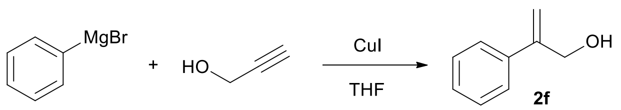

General Procedure B[2]

To a flamed-dried 100 mL round-bottom flask was added prop-2-yn-1-ol (224 mg, 4 mmol, 1.0 *eq.*), CuI (380 mg, 2 mmol, 0.5 *eq.*) and THF (50 mL). The mixture was cooled to -78 ^oC and stirred for 5 min. Phenylmagnesium bromide (1.4 mL, 2.9 M in THF, 3.0 *eq.*) was slowly added and the reaction mixture was stirred at room temperature for 16 h. After quenched with saturated NH_4Cl, the reaction mixture was extracted with EtOAc (3 × 30 mL). The combined organic layers was washed with saturated brine (50 mL), dried over anhydrous Na_2SO_4 and concentrated under reduced pressure. The crude product was purified by flash chromatography on silica gel.

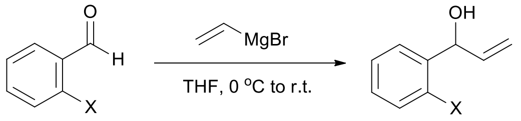

General Procedure C

To a solution of aldehyde (4 mmol, 1.0 *eq.*) in THF (50 mL) was added vinylmagnesium bromide (12 mL, 1M in THF, 3.0 *eq.*) at 0 ^oC. After stirring at room temperature for 6 h, the reaction mixture

was quenched with saturated NH_4Cl and extracted with EtOAc (3 × 30 mL). The combined organic layers was washed with saturated brine (50 mL), dried over anhydrous Na_2SO_4 and concentrated under reduced pressure. The crude product was purified by flash chromatography on silica gel.

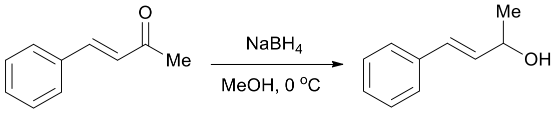

General Procedure D[3]

To a solution of (*E*)-4-phenylbut-3-en-2-one (583 mg, 4 mmol, 1.0 *eq.*) in MeOH (50 mL) was slowly added $NaBH_4$ (181 mg, 4.8 mmol, 1.2 *eq.*) at 0 °C. After stirring at the same temperature for 1h, the reaction mixture was quenched with 0.1 M HCl until no further hydrogen evolution was observed. Most of MeOH was removed under reduced pressure. The mixture was extracted with EtOAc (3 × 30 mL). The combined organic layers was washed with saturated brine (50 mL), dried over anhydrous Na_2SO_4 and concentrated under reduced pressure. The crude product was purified by flash chromatography on silica gel.

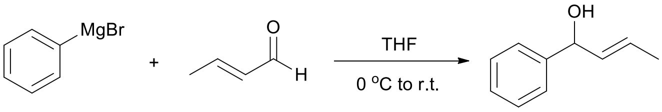

General Procedure E[3]

To a solution of crotonaldehyde (280 mg, 4 mmol, 1.0 *eq.*) in THF (50 mL) was slowly added phenylmagnesium bromide (2.1 mL, 2.9 M in THF, 1.5 *eq.*) at 0 °C. After stirring at room temperature for 1h, the reaction mixture was quenched with saturated NH_4Cl and extracted with EtOAc (3 × 30 mL). The combined organic layers was washed with saturated brine (50 mL), dried over anhydrous Na_2SO_4 and concentrated under reduced pressure. The crude product was purified by flash chromatography on silica gel.

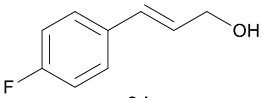

2d

(*E*)-3-(4-fluorophenyl)prop-2-en-1-ol (known compound). The title compound (492 mg, 81% yield) was synthesized from (*E*)-methyl 3-(4-fluorophenyl)acrylate according to general procedure A. The title compound was isolated as a colorless oil by silica gel column (PE/EA = 3/1). **^{1}H NMR, ^{13}C NMR** and **^{19}F NMR** data are compatible with those reported in ref. 4.

^{1}H NMR (400 MHz, $CDCl_3$, TMS): δ 7.32 (dd, *J* = 8.8 Hz, 5.2 Hz, 2H), 6.99 (dd, *J* = 8.8, 8.8

Hz, 2H), 6.55 (d, J = 16.0 Hz, 1H), 6.26 (dt, J = 16.0, 6.0 Hz, 1H), 4.29 (d, J = 5.2 Hz, 2H), 2.09 (brs, 1H).

^{13}C NMR (100 MHz, CDCl$_3$): δ 162.5 (d, $^1J_{CF}$ = 245 Hz), 132.9 (d, $^4J_{CF}$= 3 Hz), 130.0, 128.4 (d, $^5J_{CF}$ = 2 Hz), 128.1 (d, $^3J_{CF}$ = 8 Hz), 115.7 (d, $^2J_{CF}$ = 21 Hz), 63.6.

^{19}F NMR (376 MHz, CDCl$_3$): δ -114.35.

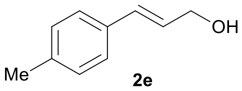

2e

(*E*)-3-(p-tolyl)prop-2-en-1-ol (known compound). The title compound (533 mg, 90% yield) was synthesized from (*E*)-methyl 3-(4-methylphenyl)acrylate according to general procedure A. The title compound was isolated as an oil by silica gel column (PE/EA = 3/1). **^{1}H NMR** and **^{13}C NMR** data are compatible with those reported in ref. 5.

^{1}H NMR (400 MHz, CDCl$_3$, TMS): δ 7.27 (d, J = 8.0 Hz, 2H), 7.11 (d, J = 8.0 Hz, 2H), 6.56 (d, J = 15.6 Hz, 1H), 6.29 (dt, J = 16.0, 6.0 Hz, 1H), 4.28 (dd, J = 5.6 Hz, 1.2 Hz, 2H), 2.33 (s, 3H), 1.85 (brs, 1H).

^{13}C NMR (100 MHz, CDCl$_3$): δ 137.6, 134.0, 131.2, 129.4, 127.6, 126.5, 63.8, 21.3.

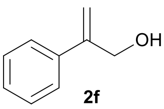

2f

2-Phenylprop-2-en-1-ol (known compound). The title compound (402 mg, 75% yield) was synthesized from prop-2-yn-1-ol according to general procedure B. The title compound was isolated as an oil by silica gel column (PE/EA = 4/1). **^{1}H NMR** and **^{13}C NMR** data are compatible with those reported in ref. 2.

^{1}H NMR (400 MHz, CDCl$_3$, TMS): δ 7.45-7.42 (m, 2H), 7.37-7.30 (m, 3H), 5.46 (s, 1H), 5.34 (d, J = 1.2 Hz, 1H), 4.52 (s, 2H), 2.02 (brs, 1H).

^{13}C NMR (100 MHz, CDCl$_3$): δ 147.4, 138.7, 128.7, 128.1, 126.2, 112.7, 65.0.

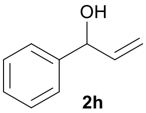

2h

1-Phenylprop-2-en-1-ol (known compound). The title compound (369 mg, 69% yield) was synthesized from benzaldehyde according to general procedure C. The title compound was isolated as an oil by silica gel column (PE/EA = 6/1). **^{1}H NMR** and **^{13}C NMR** data are compatible with those

reported in ref. 6.

^{1}H NMR (400 MHz, $CDCl_3$, TMS): δ 7.34-7.25 (m, 5H), 6.05-5.97 (m, 1H), 5.30 (dt, *J* = 17.2 Hz, 1.2 Hz, 1H), 5.18-5.13 (m, 2H), 2.34 (brs, 1H).

^{13}C NMR (100 MHz, $CDCl_3$): δ 142.7, 140.4, 128.7, 127.8, 126.5, 115.2, 75.4.

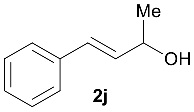

2j

(*E*)-4-phenylbut-3-en-2-ol (known compound). The title compound (562 mg, 95% yield) was synthesized from (*E*)-4-phenylbut-3-en-2-one according to general procedure D. The title compound was isolated as an oil by silica gel column (PE/EA = 3/1). **^{1}H NMR** and **^{13}C NMR** data are compatible with those reported in ref. 5.

^{1}H NMR (400 MHz, $CDCl_3$, TMS): δ 7.39-7.36 (m, 2H), 7.31 (t, *J* = 7.2 Hz, 2H), 7.55-7.22 (m, 1H), 6.55 (d, *J* = 16.0 Hz, 1H), 6.26 (dd, *J* = 16.0 Hz, 6.4 Hz, 1H), 4.51- 4.44 (m, 1H), 2.18 (brs, 1H), 1.36 (d, *J* = 6.4 Hz, 3H).

^{13}C NMR (100 MHz, $CDCl_3$): δ 136.8, 133.7, 129.4, 128.7, 127.7, 126.6, 69.0, 23.5.

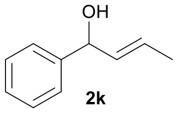

2k

1-Phenylprop-2-en-1-ol (known compound). The title compound (426 mg, 72% yield) was synthesized from crotonaldehyde according to general procedure E. The title compound was isolated as a colorless oil by silica gel column (PE/EA = 2/1). **^{1}H NMR** and **^{13}C NMR** data are compatible with those reported in ref. 7.

^{1}H NMR (400 MHz, $CDCl_3$, TMS): δ 7.36-7.31 (m, 4H), 7.29-7.23 (m, 1H), 5.78-5.64 (m, 2H), 5.13 (d, *J* = 6.4 Hz, 1H), 2.07 (brs, 1H), 1.70 (d, *J* = 5.6 Hz, 3H).

^{13}C NMR (100 MHz, $CDCl_3$): δ 143.5, 133.7, 128.5, 127.54, 127.45, 126.2, 75.2, 17.8.

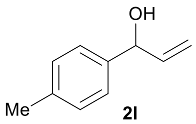

2l

1-(p-tolyl)prop-2-en-1-ol (known compound). The title compound (402 mg, 68% yield) was synthesized from 4-methylbenzaldehyde according to general procedure C. The title compound was isolated as an oil by silica gel column (PE/EA = 6/1). **^{1}H NMR** and **^{13}C NMR** data are compatible

with those reported in ref. 6.

^{1}H NMR (400 MHz, CDCl$_3$, TMS): δ 7.22 (d, *J* = 8.0 Hz, 2H), 7.14 (d, *J* = 8.0 Hz, 2H), 6.05-5.96 (m, 1H), 5.30 (dt, *J* = 17.2 Hz, 1.2 Hz, 1H), 5.16-5.10 (m, 2H), 2.32 (s, 3H), 2.28 (brs, 1H).

^{13}C NMR (100 MHz, CDCl$_3$): δ 140.5, 139.9, 137.5, 129.3, 126.4, 114.9, 75.2, 21.2.

5. Allylation Reactions of Aryl Bromides with Allylic Alcohols

5.1 General procedure for the allylation of aryl bromides with allylic alcohols

To a reaction tube charged with Ni(dppp)Cl$_2$ (10.8 mg, 0.02 mmol), bpy (6.2 mg, 0.04 mmol), ZrCl$_4$ (4.7 mg, 0.02 mmol) and Mn (32.9 mg, 0.6 mmol) was added a solution of allylic alcohol (0.2 mmol) and aryl bromide (0.3 mmol) in DMA (2 mL, 0.1 M). The reaction mixture was frozen by submersion in a liquid nitrogen bath. The reaction tube was vacuumed and then filled with argon for three times. The reaction mixture was stirred at listed temperature for listed time. The reaction was quenched with water (30 mL) and extracted with EtOAc (3 × 20 mL). The combined organic layers was washed with saturated brine (20 mL), dried over anhydrous Na$_2$SO$_4$ and concentrated under reduced pressure. The residue was purified by silica gel column to give the desired cross-coupling product.

5.2 Characterization data of allylated product

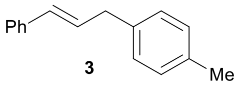

3

1-Cinnamyl-4-methylbenzene (known compound). The title compound was prepared according to the general procedure, using 4-bromotoluene (**1aa**, 51 mg, 0.3 mmol) and (*E*)-3-phenylprop-2-en-1-ol (**2a**, 27 mg, 0.2 mmol). The reaction was conducted at 30 ℃ for 48 h. The crude product was purified by flash chromatography (100:0 petroleum ether/ethyl acetate) to afford a colorless oil. **^{1}H NMR** and **^{13}C NMR** data are compatible with those reported in ref. 8.

First run: 36 mg (86%). **Second run**: 35 mg (84%).

^{1}H NMR (400 MHz, CDCl$_3$, TMS): δ 7.35 (d, *J* = 8.0 Hz, 2H), 7.28 (t, *J* = 8.0 Hz, 2H), 7.19 (t, *J* = 7.2 Hz, 1H), 7.14-7.10 (m, 4H), 6.44 (d, *J* = 15.6 Hz, 1H), 6.34 (dt, *J* = 15.6 Hz, 6.4 Hz, 1H),

3.50 (d, J = 6.4Hz, 2H), 2.33 (s, 3H).

^{13}C NMR (100 MHz, $CDCl_3$): δ 137.7, 137.2, 135.8, 131.0, 129.7, 129.4, 128.73, 128.65, 127.2, 126.3, 39.1, 21.2.

IR (cm^{-1}): 3024, 2921, 1598, 1514, 1496, 1448, 965, 809, 755, 691.

GC-MS (EI) m/z (rel intensity, ion): 208.11 (100.00, M^+).

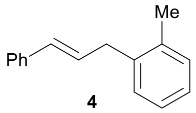

4

1-Cinnamyl-2-methylbenzene (known compound). The title compound was prepared according to the general procedure, using 2-bromotoluene (**1ab**, 51 mg, 0.3 mmol) and (*E*)-3-phenylprop-2-en-1-ol (**2a**, 27 mg, 0.2 mmol). The reaction was conducted at 30 ℃ for 48 h. The crude product was purified by flash chromatography (100:0 petroleum ether/ethyl acetate) to afford a colorless oil. **^{1}H NMR** and **^{13}C NMR** data are compatible with those reported in ref. 9.

First run: 30 mg (72%). **Second run**: 32 mg (78%).

^{1}H NMR (400 MHz, $CDCl_3$, TMS): δ 7.40 (d, J = 7.6 Hz, 2H), 7.34 (t, J = 7.6 Hz, 2H), 7.28-7.22 (m, 5H), 6.42-6.40 (m, 2H), 3.58 (d, J = 4.8 Hz, 2H), 2.40 (s, 3H).

^{13}C NMR (100 MHz, $CDCl_3$): δ 138.4, 137.7, 136.6, 131.1, 130.4, 129.4, 128.71, 128.66, 127.2, 126.6, 126.3, 126.2, 37.0, 19.6.

IR (cm^{-1}): 3024, 2923, 1494, 1448, 966, 744, 692.

GC-MS (EI) m/z (rel intensity, ion): 208.07 (100.00, M^+).

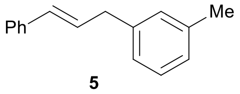

5

1-Cinnamyl-3-methylbenzene (known compound). The title compound was prepared according to the general procedure, using 3-bromotoluene (**1ac**, 51 mg, 0.3 mmol) and (*E*)-3-phenylprop-2-en-1-ol (**2a**, 27 mg, 0.2 mmol). The reaction was conducted at 30 ℃ for 48 h. The crude product was purified by flash chromatography (100:0 petroleum ether/ethyl acetate) to afford a colorless oil. **^{1}H NMR** and **^{13}C NMR** data are compatible with those reported in ref. 9.

First run: 32 mg (78%). **Second run**: 33 mg (79%).

^{1}H NMR (400 MHz, $CDCl_3$, TMS): δ 7.35 (dd, J = 7.6 Hz, 1.2Hz, 2H), 7.28 (t, J = 7.6 Hz, 2H), 7.19 (t, J = 7.6 Hz, 2H), 7.05-7.02 (m, 3H), 6.45 (d, J = 16.0 Hz, 1H), 6.34 (dt, J = 16.0 Hz, 6.4 Hz, 1H), 3.51 (d, J = 6.8 Hz, 2H), 2.33 (s, 3H).

^{13}C NMR (100 MHz, CDCl$_3$): δ 140.3, 138.3, 137.7, 131.1, 129.62, 129.55, 128.7, 128.6, 127.3, 127.1, 126.3, 125.9, 39.5, 21.6.

IR (cm^{-1}): 3025, 2920, 1607, 1494, 965, 750, 692.

GC-MS (EI) m/z (rel intensity, ion): 208.06 (100.00, M$^+$).

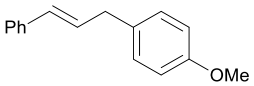

6

1-Cinnamyl-4-methoxybenzene (known compound[8]). The title compound was prepared according to the general procedure, using 1-bromo-4-methoxybenzene (**1ad**, 56 mg, 0.3 mmol) and (*E*)-3-phenylprop-2-en-1-ol (**2a**, 27 mg, 0.2 mmol). The reaction was conducted at 30 ℃ for 48 h. The crude product was purified by flash chromatography (20:1 petroleum ether/ethyl acetate) to afford a light yellow oil. **^{1}H NMR** and **^{13}C NMR** data are compatible with those reported in ref. 8.

First run: 31 mg (69%). **Second run**: 30 mg (67%).

^{1}H NMR (400 MHz, CDCl$_3$, TMS): δ 7.31 (d, *J* = 8.0 Hz, 2H), 7.26 (d, *J* = 8.0 Hz, 2H), 7.19-7.12 (m, 3H), 6.84 (d, *J* = 7.2 Hz, 2H), 6.41 (d, *J* = 16.0 Hz, 1H), 6.36-6.28 (m, 1H), 3.76 (s, 3H), 3.7 (d, *J* = 6.8 Hz, 2H).

^{13}C NMR (100 MHz, CDCl$_3$): δ 158.3, 137.7, 132.3, 130.9, 129.84, 129.76, 129.7, 127.2, 126.3, 114.1, 55.4, 38.6.

IR (cm^{-1}): 3026, 2834, 1510, 1245, 1036, 966, 828, 729, 692.

GC-MS (EI) m/z (rel intensity, ion): 224.04 (100.00, M$^+$).

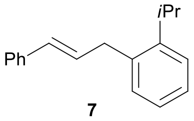

7

1-Cinnamyl-2-isopropylbenzene. The title compound was prepared according to the general procedure, using 1-bromo-2-isopropylbenzene (**1ae**, 60 mg, 0.3 mmol) and (*E*)-3-phenylprop-2-en-1-ol (**2a**, 27 mg, 0.2 mmol). Catalyst: Ni(diglyme)Br$_2$ (7.0 mg, 0.02 mmol) was used. The reaction was conducted at 20 ℃ for 32 h. The crude product was purified by flash chromatography (100:0 petroleum ether/ethyl acetate) to afford a colorless oil.

First run: 40 mg (84%). **Second run**: 38 mg (80%).

^{1}H NMR (400 MHz, CDCl$_3$, TMS): δ 7.33-7.13 (m, 9H), 6.36-6.34 (m, 2H), 3.59 (d, *J* = 4.8 Hz, 2H), 3.25-3.18 (m, 1H), 1.23 (d, *J* = 6.8 Hz, 6H).

^{13}C NMR (100 MHz, CDCl$_3$)**: δ 147.1, 137.8, 136.9, 130.9, 130.0, 129.8, 128.7, 127.2, 127.0, 126.2, 126.0, 125.6, 36.4, 29.0, 24.1.

IR (cm^{-1}): 3025, 2963, 1600, 1489, 1448, 1033, 758, 738, 692.

HRMS (EI): [M$^+$] calcd for $C_{18}H_{20}$ 236.1565, found 236.1566.

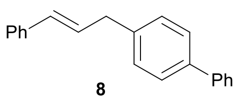

8

4-Cinnamyl-1,1'-biphenyl (known compound). The title compound was prepared according to the general procedure, using 4-bromo-1,1'-biphenyl (**1af**, 70 mg, 0.3 mmol) and (*E*)-3-phenylprop-2-en-1-ol (**2a**, 27 mg, 0.2 mmol). The reaction was conducted at 30 °C for 48 h. The crude product was purified by flash chromatography (100:0 petroleum ether/ethyl acetate) to afford a pale yellow solid. M.P. = 41 – 42 ^{o}C. **^{1}H NMR** and **^{13}C NMR** data are compatible with those reported in ref. 10.

First run: 45 mg (83%). **Second run**: 47 mg (87%).

^{1}H NMR (400 MHz, CDCl$_3$, TMS): δ 7.58 (d, *J* = 7.2 Hz, 2H), 7.54 (d, *J* = 8.0 Hz, 2H), 7.44-7.40 (m, 2H), 7.38-7.27 (m, 7H), 7.22-7.18 (m, 1H), 6.49 (d, *J* = 16.0 Hz, 1H), 6.38 (dt, *J* = 16.0 Hz, 6.8 Hz, 1H), 3.59 (d, *J* = 6.8 Hz, 2H).

^{13}C NMR (100 MHz, CDCl$_3$): δ 141.2, 139.5, 139.4, 137.7, 131.4, 129.3, 128.9, 128.7, 127.4, 127.34, 127.28, 127.2, 126.4, 39.2.

IR (cm^{-1}): 3026, 1486, 965, 832, 761, 747, 693.

GC-MS (EI) m/z (rel intensity, ion): 270.08 (100.00, M$^+$).

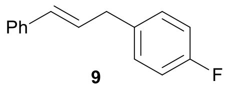

9

1-Cinnamyl-4-fluorobenzene (known compound). The title compound was prepared according to the general procedure, using 1-bromo-4-fluorobenzene (**1ag**, 52 mg, 0.3 mmol) and (*E*)-3-phenylprop-2-en-1-ol (**2a**, 27 mg, 0.2 mmol). The reaction was conducted at 30 °C for 48 h. The crude product was purified by flash chromatography (100:0 petroleum ether/ethyl acetate) to afford a pale yellow oil. **^{1}H NMR** and **^{13}C NMR** data are compatible with those reported in ref. 8.

First run: 39 mg (91%). **Second run**: 36 mg (85%).

^{1}H NMR (400 MHz, CDCl$_3$, TMS): δ 7.35 (d, *J* = 7.2 Hz, 2H), 7.29 (t, *J* = 7.2 Hz, 2H), 7.23-7.16 (m, 3H), 6.98 (d, *J* = 8.8 Hz, 2H), 6.43 (d, *J* = 16.0 Hz, 1H), 6.31 (dt, *J* = 16.0 Hz, 6.4 Hz,

1H), 3.50 (d, J = 6.8 Hz, 2H).

^{13}C NMR (100 MHz, CDCl$_3$): δ 161.7 (d, $^1J_{CF}$ = 242 Hz), 137.5, 135.9 (d, $^4J_{CF}$= 3 Hz), 131.4, 130.2 (d, $^3J_{CF}$= 8 Hz), 129.2, 128.7, 127.4, 126.3, 115.4 (d, $^2J_{CF}$ = 21 Hz), 38.7.

^{19}F NMR (376 MHz, CDCl$_3$): δ -117.26.

IR (cm^{-1}): 3027, 1601, 1509, 1221, 966, 829, 730, 692.

GC-MS (EI) m/z (rel intensity, ion): 212.07 (100.00, M$^+$).

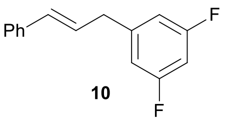

10

1-Cinnamyl-3,5-difluorobenzene. The title compound was prepared according to the General Procedure, using 1-bromo-3,5-difluorobenzene (**1ah**, 58 mg, 0.3 mmol) and (*E*)-3-phenylprop-2-en-1-ol (**2a**, 27 mg, 0.2 mmol). The reaction was conducted at 30 ℃ for 48 h. The crude product was purified by flash chromatography (100:0 petroleum ether/ethyl acetate) to afford a colorless oil.

First run: 23 mg (50%). **Second run**: 26 mg (56%).

^{1}H NMR (400 MHz, CDCl$_3$, TMS): δ 7.37-7.28 (m, 4H), 7.24-7.20 (m, 1H), 6.76 (dd, J = 8.0 Hz, 2 Hz, 2H), 6.68-6.63 (m, 1H), 6.47 (d, J = 15.6 Hz, 1H), 6.27 (dt, J = 15.6 Hz, 6.8 Hz, 1H), 3.51 (d, J = 6.8 Hz, 2H).

^{13}C NMR (100 MHz, CDCl$_3$): δ 163.2 (dd, $^1J_{CF}$ = 247 Hz, $^3J_{CF}$ = 13 Hz), 144.4, 137.2, 132.5, 128.8, 127.7, 127.4, 126.4, 111.5 (dd, $^2J_{CF}$ = 18 Hz, $^3J_{CF}$ = 7 Hz), 101.9 (t, $^2J_{CF}$ = 26 Hz), 39.1.

^{19}F NMR (376 MHz, CDCl$_3$): δ -110.35.

IR (cm^{-1}): 3028, 1625, 1597, 1117, 966, 851, 748, 684.

HRMS (EI): [M$^+$] calcd for $C_{15}H_{12}F_2$ 230.0907, found 230.0899.

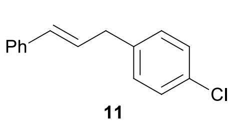

11

1-Chloro-4-cinnamylbenzene (known compound). The title compound was prepared according to the general procedure, using 1-bromo-4-chlorobenzene (**1ai**, 57 mg, 0.3 mmol) and (*E*)-3-phenylprop-2-en-1-ol (**2a**, 27 mg, 0.2 mmol). The reaction was conducted at 30 ℃ for 48 h. The crude product was purified by flash chromatography (100:0 petroleum ether/ethyl acetate) to afford a pale yellow oil. **^{1}H NMR** and **^{13}C NMR** data are compatible with those reported in ref. 8.

First run: 30 mg (66%). **Second run**: 32 mg (70%).

^{1}H NMR (400 MHz, $CDCl_3$, TMS): δ 7.36-7.17 (m, 9H), 6.44 (d, *J* = 15.6 Hz, 1H), 6.30 (dt, *J* = 15.6 Hz, 6.8 Hz, 1H), 3.51 (d, *J* = 6.8 Hz, 2H).

^{13}C NMR (100 MHz, $CDCl_3$): δ 138.7, 137.4, 132.1, 131.6, 130.2, 128.74, 128.72, 127.4, 126.3, 38.8.

IR (cm^{-1}): 3026, 1491, 1091, 965, 744, 692.

GC-MS (EI) m/z (rel intensity, ion): 228.02 (65.15, M^+).

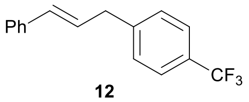

12

1-Cinnamyl-4-(trifluoromethyl)benzene (known compound). The title compound was prepared according to the General Procedure, using 1-bromo-4-(trifluoromethyl)benzene (**1aj**, 67 mg, 0.3 mmol) and (*E*)-3-phenylprop-2-en-1-ol (**2a**, 27 mg, 0.2 mmol). The reaction was conducted at 30 ℃ for 48 h. The crude product was purified by flash chromatography (100:0 petroleum ether/ethyl acetate) to afford a colorless oil. **^{1}H NMR** and **^{13}C NMR** data are compatible with those reported in ref. 9.

First run: 32 mg (61%). **Second run**: 29 mg (56%).

^{1}H NMR (400 MHz, $CDCl_3$, TMS): δ 7.56 (d, *J* = 8.0 Hz, 2H), 7.36-7.28 (m, 6H), 7.24-7.19 (m, 1H), 6.47 (d, *J* = 16.0 Hz, 1H), 6.32 (dt, *J* = 15.6 Hz, 6.8 Hz, 1H), 3.59 (d, *J* = 6.8 Hz, 2H).

^{13}C NMR (100 MHz, $CDCl_3$): δ 144.4, 137.2, 132.0, 129.1, 128.70, 128.69 (q, *J* = 32 Hz), 128.0, 127.5, 126.3,125.5 (q, *J* = 4 Hz), 124.5(q, *J* = 270 Hz), 39.3.

^{19}F NMR (376 MHz, $CDCl_3$): δ -62.32.

IR (cm^{-1}): 3028, 1618, 1326, 1163, 1123, 1067, 1018, 965, 832, 692.

GC-MS (EI) m/z (rel intensity, ion): 262.04 (100.00, M^+).

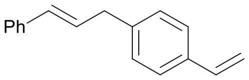

13

1-Cinnamyl-4-vinylbenzene (known compound). The title compound was prepared according to the general procedure, using 1-bromo-4-vinylbenzene (**1ak**, 55 mg, 0.3 mmol) and (*E*)-3-phenylprop-2-en-1-ol (**2a**, 27 mg, 0.2 mmol). The reaction was conducted at 30 ℃ for 48 h. The crude product was purified by flash chromatography (100:0 petroleum ether/ethyl acetate) to afford a pale yellow oil. **^{1}H NMR** and **^{13}C NMR** data are compatible with those reported in ref. 9.

First run: 23 mg (53%). **Second run**: 24 mg (55%).

^{1}H NMR (400 MHz, $CDCl_3$, TMS): δ 7.37-7.35 (m, 4H), 7.29 (t, *J* = 7.2 Hz, 2H), 7.21-7.19 (m, 3H), 6.70 (dd, *J* = 17.6 Hz, 10.8 Hz, 1H), 6.45 (d, *J* = 16.0 Hz, 1H), 6.33 (dt, *J* = 15.6 Hz, 6.8 Hz, 1H), 5.72 (d, *J* = 17.6 Hz, 1H), 5.20 (d, *J* = 10.8 Hz, 1H), 3.53 (d, *J* = 6.4 Hz, 2H).

^{13}C NMR (100 MHz, $CDCl_3$): δ 140.0, 137.7, 136.8, 135.9, 131.3, 129.2, 129.0, 128.7, 127.3, 126.6, 126.3, 113.4, 39.2.

IR (cm^{-1}): 3025, 1629, 1510, 1496, 1448, 1405, 965, 907, 738, 692.

GC-MS (EI) m/z (rel intensity, ion): 220.06 (100, M^+).

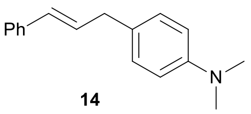

14

4-Cinnamyl-*N*,*N*-dimethylaniline (known compound). The title compound was prepared according to the general procedure, using 4-bromo-N,N-dimethylaniline (**1al**, 60 mg, 0.3 mmol) and (*E*)-3-phenylprop-2-en-1-ol (**2a**, 27 mg, 0.2 mmol). The reaction was conducted at 30 ℃ for 48 h. The crude product was purified by flash chromatography (50:1 petroleum ether/ethyl acetate) to afford a light yellow oil. **^{1}H NMR** and **^{13}C NMR** data are compatible with those reported in ref. 9.

First run: 37 mg (79%). **Second run**: 36 mg (77%).

^{1}H NMR (400 MHz, $CDCl_3$, TMS): δ 7.34 (d, *J* = 7.2 Hz, 2H), 7.27 (t, *J* = 7.6 Hz, 2H), 7.18 (t, *J* = 7.2 Hz, 1H), 7.11 (d, *J* = 7.6 Hz, 2H), 6.71 (d, *J* = 8.8 Hz, 2H), 6.43 (d, *J* = 16.0 Hz, 1H), 6.34 (dt, *J* = 15.6 Hz, 6.4Hz, 1H), 3.45 (d, *J* = 6.4 Hz, 2H), 2.91 (s, 6H).

^{13}C NMR (100 MHz, $CDCl_3$): δ 149.5, 137.9, 130.5, 130.4, 129.5, 128.6, 128.4, 127.1, 126.3, 113.3, 41.1, 38.6.

IR (cm^{-1}): 3024, 2888, 2800, 1615, 1520, 1344, 1163, 965, 818, 748, 696.

HRMS (ESI): $[M+H]^+$ calcd for $C_{17}H_{19}N$ 238.1590, found 238.1594.

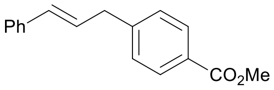

15

1-(4-Cinnamylphenyl)ethan-1-one (known compound).

Reaction in scheme 2: The title compound was prepared according to the general procedure, using methyl 4-bromobenzoate (**1am**, 64 mg, 0.3 mmol) and (*E*)-3-phenylprop-2-en-1-ol (**2a**, 27 mg, 0.2 mmol). The reaction was conducted at 30 ℃ for 48 h. The crude product was purified by flash chromatography (30:1 petroleum ether/ethyl acetate) to afford a light yellow oil. **^{1}H NMR** and **^{13}C NMR** data are compatible with those reported in ref. 11.

First run: 28 mg (56%). **Second run**: 31 mg (62%).

Reaction in table 1, entry 7: The title compound was prepared according to the General Procedure, using methyl 4-bromobenzoate (**1am**, 85 mg, 0.4 mmol) and 1-phenylprop-2-en-1-ol (**2h**, 27 mg, 0.2 mmol). The reaction was conducted at 30 ℃ for 48 h. The crude product was purified by flash chromatography (30:1 petroleum ether/ethyl acetate) to afford a light yellow oil.

First run: 39 mg (77%). **Second run**: 38 mg (75%).

^{1}H NMR (400 MHz, $CDCl_3$, TMS): δ 7.98 (d, *J* = 8.0 Hz, 2H), 7.36-7.27 (m, 6H), 7.21 (t, *J* = 7.2 Hz, 1H), 6.47 (d, *J* = 16.0 Hz, 1H), 6.32 (dt, *J* = 15.6 Hz, 6.8 Hz, 1H), 3.90 (s, 3H) 3.59 (d, *J* = 6.8 Hz, 2H).

^{13}C NMR (100 MHz, $CDCl_3$): δ 167.2, 145.8, 137.4, 132.0, 130.0, 128.9, 128.7, 128.4, 128.2, 127.5, 126.3, 52.2, 39.4.

IR (cm^{-1}): 3026, 2950, 1720, 1610, 1435, 1280, 1110, 966, 743, 693.

HRMS (ESI): $[M+H]^+$ calcd for $C_{17}H_{16}O_2$ 253.1223, found 253.1222.

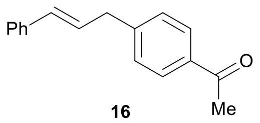

16

1-(4-Cinnamylphenyl)ethan-1-one (known compound). The title compound was prepared according to the general procedure, using 1-(4-bromophenyl)ethanone (**1an**, 59 mg, 0.3 mmol) and (*E*)-3-phenylprop-2-en-1-ol (**2a**, 27 mg, 0.2 mmol). The reaction was conducted at 30 ℃ for 52 h. The crude product was purified by flash chromatography (20:1 petroleum ether/ethyl acetate) to afford a light yellow oil. **^{1}H NMR** and **^{13}C NMR** data are compatible with those reported in ref. 11.

First run: 21 mg (44%). **Second run**: 19 mg (40%).

^{1}H NMR (400 MHz, $CDCl_3$, TMS): δ 7.90 (d, *J* = 8.0 Hz, 2H), 7.36-7.27 (m, 6H), 7.21 (t, *J* = 7.2 Hz, 1H), 6.47 (d, *J* = 16.0 Hz, 1H), 6.33 (d, *J* = 16.0 Hz, 6.8Hz, 1H), 3.60 (d, *J* = 6.4 Hz, 2H), 2.59 (s, 3H).

^{13}C NMR (100 MHz, $CDCl_3$): δ 198.0, 146.1, 137.4, 135.6, 132.1, 129.1, 128.9, 128.7, 128.1, 127.5, 126.4, 39.4, 26.8.

IR (cm^{-1}): 3026, 1681, 1606, 1357, 1268, 965, 749, 694.

GC-MS (EI) m/z (rel intensity, ion): 236.08 (100, M^+).

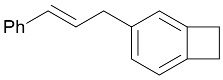

17

3-Cinnamylbicyclo[4.2.0]octa-1,3,5-triene. The title compound was prepared according to the general procedure, using 3-bromobicyclo[4.2.0]octa-1(6),2,4-triene (**1ao**, 54 mg, 0.3 mmol) and (*E*)-3-phenylprop-2-en-1-ol (**2a**, 27 mg, 0.2 mmol). The reaction was conducted at 30 ℃ for 48 h. The crude product was purified by flash chromatography (100:0 petroleum ether/ethyl acetate) to afford a light yellow oil.

First run: 36 mg (82%). **Second run**: 37 mg (85%).

^{1}H NMR (400 MHz, $CDCl_3$, TMS): δ 7.34 (d, *J* = 7.2 Hz, 2H), 7.27 (t, *J* = 7.6 Hz, 2H), 7.21-7.18 (m, 1H), 7.06 (d, *J* = 7.6 Hz, 1H), 6.99-6.95 (m, 2 H), 6.43 (d, *J* = 16.0 Hz, 1H), 6.33 (dt, *J* = 16.0 Hz, 6.4 Hz, 1H), 3.50 (d, *J* = 6.8 Hz, 2H), 3.14 (s, 4H).

^{13}C NMR (100 MHz, $CDCl_3$): δ 146.2, 143.7, 138.9, 137.8, 130.9, 130.1, 128.7, 127.4, 127.2, 126.3, 123.1, 122.7, 40.2, 29.6, 29.4.

IR (cm^{-1}): 3024, 2918, 1474, 1448, 1195, 964, 764, 691.

HRMS (EI): [M^+] calcd for $C_{17}H_{16}$ 220.1252, found 220.1260.

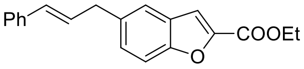

18

Ethyl 5-cinnamylbenzofuran-2-carboxylate. The title compound was prepared according to the general procedure, using methyl 6-bromo-1H-indole-2-carboxylate (**1ap**, 77 mg, 0.3 mmol) and (*E*)-3-phenylprop-2-en-1-ol (**2a**, 27 mg, 0.2 mmol). Catalyst: Ni(dppp)Cl_2 (10.8 mg, 0.02 mmol), bpy (6.2 mg, 0.04 mmol), $ZrCl_4$ (4.7 mg, 0.02 mmol). The reaction was conducted at 30 ℃ for 48 h. The crude product was purified by flash chromatography (4:1 petroleum ether/ethyl acetate) to afford a white solid.

First run: 29 mg (48 %). **Second run**: 30 mg (49 %), white solid, M.P. = 159 – 160 °C

^{1}H NMR (400 MHz, $CDCl_3$, TMS): δ 7.53-7.47 (m, 2H), 7.47 (d, *J* = 0.8 Hz, 1H), 7.37-7.27 (m, 5H), 7.22-7.19 (m, 1H), 6.47 (d, *J* = 16.0 Hz, 1H), 6.38 (dt, *J*= 16.0 Hz, 6.4 Hz, 1H), 4.44 (q, *J* = 7.2 Hz, 2H), 3.64 (d, *J* = 8.0 Hz, 2H), 1.42 (t, *J* = 7.2 Hz, 3H).

^{13}C NMR (100 MHz, $CDCl_3$): δ 159.7, 154.7, 146.1, 137.4, 135.9, 131.4, 129.2, 128.8, 128.6, 127.3, 126.2, 122.3, 113.8, 112.3, 61.6, 39.2, 14.4.

IR (cm^{-1}): 3081, 3024, 3059, 3026, 2982, 1729, 1575, 1466, 1446, 1369, 1294, 1193, 1137, 1095, 1018, 967, 764, 740, 693.

HRMS (ESI): $[M+H]^+$ calcd. for $C_{20}H_{19}O_3$ 307.1329 found 307.1324.

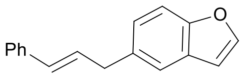

19

5-Cinnamylbenzofuran. The title compound was prepared according to the general procedure, using 5-bromobenzofuran (**1aq**, 59 mg, 0.3 mmol) and (*E*)-3-phenylprop-2-en-1-ol (**2a**, 27 mg, 0.2 mmol). The reaction was conducted at 30 ℃ for 48 h. The crude product was purified by flash chromatography (20:1 petroleum ether/ethyl acetate) to afford a light yellow oil.

First run: 41 mg (88%). **Second run**: 43 mg (91%).

^{1}H NMR (400 MHz, $CDCl_3$, TMS): δ 7.58 (d, J = 2.4 Hz, 1H), 7.44-7.42 (m, 2H), 7.35 (d, J = 7.6 Hz, 2H), 7.28 (t, J = 7.2 Hz, 2H), 7.21-7.14 (m, 2H), 6.70-6.69 (m, 1H), 6.48-6.35 (m, 2H), 3.62 (d, J = 6.0 Hz, 2H).

^{13}C NMR (100 MHz, $CDCl_3$): δ 154.0, 145.4, 137.7, 134.8, 131.0, 130.0, 128.7, 127.9, 127.3, 126.3, 125.3, 121.0, 111.4, 106.6, 39.4.

IR (cm^{-1}): 3025, 1495, 1467, 1262, 1030, 966, 758, 735, 693.

HRMS (ESI): $[M+H]^+$ calcd for $C_{17}H_{14}O_2$ 235.1117, found 235.1116.

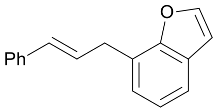

20

7-Cinnamylbenzofuran. The title compound was prepared according to the general procedure, using 7-bromobenzofuran (**1ar**, 59 mg, 0.3 mmol) and (*E*)-3-phenylprop-2-en-1-ol (**2a**, 27 mg, 0.2 mmol). The reaction was conducted at 30 ℃ for 48 h. The crude product was purified by flash chromatography (20:1 petroleum ether/ethyl acetate) to afford a pale yellow solid. M.P. = 63 – 64 ^{o}C.

First run: 38 mg (81%). **Second run**: 40 mg (86%).

^{1}H NMR (400 MHz, $CDCl_3$, TMS): δ 7.60 (d, J = 2.0 Hz, 1H), 7.45 (d, J = 7.2 Hz, 1H), 7.34 (d, J = 7.6 Hz, 2H), 7.26 (t, J = 7.6 Hz, 2H), 7.19-7.14 (m, 3H), 6.75 (d, J = 2.0 Hz, 1H), 6.54-6.41 (m, 2H), 3.83 (d, J = 6.0 Hz, 2H).

^{13}C NMR (100 MHz, $CDCl_3$): δ 153.7, 144.8, 137.6, 131.4, 128.6, 127.9, 127.3, 127.2, 126.2, 124.4, 124.0, 123.1, 119.4, 107.0, 33.2.

IR (cm^{-1}): 3027, 2958, 1597, 1495, 1427, 1175, 1126, 965, 793, 738, 696.

HRMS (ESI): $[M+H]^+$ calcd for $C_{17}H_{14}O_2$ 235.1117, found 235.1115.

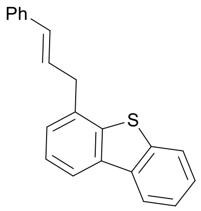

21

4-Cinnamyldibenzo[*b,d*]thiophene. The title compound was prepared according to the general procedure, using 4-bromodibenzo[b,d]thiophene (**1as**, 79 mg, 0.3 mmol) and (*E*)-3-phenylprop-2-en-1-ol (**2a**, 27 mg, 0.2 mmol). The reaction was conducted at 30 ℃ for 48 h. The crude product was purified by flash chromatography (20:1 petroleum ether/ethyl acetate) to afford a pale yellow solid. M.P. = 91 – 92 ^oC.

First run: 43 mg (72%). **Second run**: 38 mg (64%).

^{1}H NMR (400 MHz, CDCl$_3$, TMS): δ 8.16-8.14 (m, 1H), 8.05 (d, *J* = 7.6 Hz, 1H), 7.87-7.85 (m, 1H), 7.47-7.42 (m, 3H), 7.38-7.34 (m, 3H), 7.29 (t, *J* = 7.2 Hz, 2H), 7.22-7.18 (m, 1H), 6.62 (d, *J* = 16.0 Hz, 1H), 6.45 (dt, *J* = 16.0 Hz, 6.8 Hz, 1H), 3.82 (d, *J* = 6.4 Hz, 2H).

^{13}C NMR (100 MHz, CDCl$_3$): δ 139.5, 139.4, 137.5, 136.2, 136.1, 134.7, 132.5, 128.7, 127.5, 126.9, 126.7, 126.4, 125.1, 124.6, 123.0, 121.9, 119.9, 38.7.

IR (cm^{-1}): 3026, 2916, 2849, 1442, 1400, 1264, 966, 738.

HRMS (EI): $[M^+]$ calcd for $C_{21}H_{16}S$ 300.0973, found 300.0989.

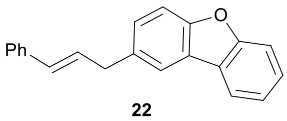

22

(*E*)-2-(4-phenylbut-2-en-1-yl)dibenzo[*b,d*]furan. The title compound was prepared according to the general procedure, using 2-bromodibenzo[b,d]furan (**1at**, 74 mg, 0.3 mmol) and (*E*)-3-phenylprop-2-en-1-ol (**2a**, 27 mg, 0.2 mmol). The reaction was conducted at 30 ℃ for 48 h. The crude product was purified by flash chromatography (20:1 petroleum ether/ethyl acetate) to afford a light yellow oil.

First run: 48 mg (84%). **Second run**: 51 mg (90%).

Gram scale reaction: To a 100 mL round-bottomed flask charged with Ni(dppp)Cl_2 (270 mg, 0.5 mmol), bpy (156 mg, 1 mmol), $ZrCl_4$ (118 mg, 0.5 mmol) and Mn (825 mg, 15 mmol) was added a solution of 2-bromodibenzo[b,d]furan (**1at**, 1.85 g, 7.5 mmol) and (*E*)-3-phenylprop-2-en-1-ol (**2a**,

0.67 g, 5.0 mmol) in DMA (50 mL). The reaction mixture was frozen by submersion in a liquid nitrogen bath. After being vacuumed and filled with argon for three times, the reaction mixture was allowed to warm to 30 ℃ and stirred for 95 h. The reaction was quenched with water (100 mL) and extracted with EtOAc (3 × 50 mL). The combined organic layers was washed with brine (100 mL), dried over anhydrous Na_2SO_4 and concentrated under reduced pressure. The crude product was purified by flash chromatography (20:1 petroleum ether/ethyl acetate) to afford 1.07 g (75%) of desired product **22** as a light yellow oil.

^{1}H NMR (400 MHz, $CDCl_3$, TMS): δ 7.92 (d, *J* = 7.6 Hz, 1H), 8.00 (d, *J* = 0.4 Hz, 1H), 7.54 (d, *J* = 8.4, 1H), 7.49 (d, *J* = 8.4, 1H), 7.43 (t, *J* = 8.0, 1H), 7.38 (d, *J* = 7.6 Hz, 2H), 7.33-7.27 (m, 4H), 7.23-7.18 (m, 1H), 6.51-6.39 (m, 2H), 3.69 (d, *J* = 6.4 Hz, 2H).

^{13}C NMR (100 MHz, $CDCl_3$): δ 156.7, 155.2, 137.6, 134.8, 131.3, 129.7, 128.7, 128.1, 127.4, 127.2, 126.4, 124.6, 124.4, 122.8, 120.8, 120.6, 111.8, 111.6, 39.4.

IR (cm^{-1}): 3025, 1479, 1448, 1195, 965, 841, 749, 692.

HRMS (EI): [M^+] calcd for $C_{21}H_{16}O$ 284.1201, found 284.1216.

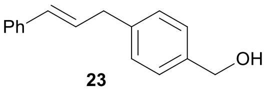

23

(4-Cinnamylphenyl)methanol. The title compound was prepared according to the general procedure, using (4-bromophenyl)methanol (**1au**, 56 mg, 0.3 mmol) and (*E*)-3-phenylprop-2-en-1-ol (**2a**, 27 mg, 0.2 mmol). The reaction was conducted at 30 ℃ for 60 h. The crude product was purified by flash chromatography (4:1 petroleum ether/ethyl acetate) to afford a colorless oil.

First run: 33 mg (74%). **Second run**: 38 mg (84%).

^{1}H NMR (400 MHz, $CDCl_3$, TMS): δ 7.36-7.18 (m, 9H), 6.45 (d, *J* = 15.6 Hz, 1H), 6.34 (dd, *J* = 15.6 Hz, 6.8 Hz, 1H), 4.67 (s, 2H), 3.54 (d, *J* = 6.8 Hz, 2H), 1.64 (brs, 1H).

^{13}C NMR (100 MHz, $CDCl_3$): δ 139.9, 139.0, 137.6, 131.3, 129.3, 129.1, 128.7, 127.5, 127.3, 126.3, 65.4, 39.2.

IR (cm^{-1}): 3333, 3025, 2918, 1541, 1419, 965, 756, 734, 692.

HRMS (ESI): $[M+Na]^+$ calcd for $C_{16}H_{16}O$ 247.1093, found 247.1095.

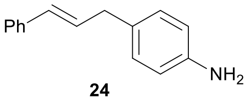

24

4-Cinnamylaniline (known compound). The title compound was prepared according to the

general procedure, using 4-bromoaniline (**1av**, 34 mg, 0.2 mmol) and (*E*)-3-phenylprop-2-en-1-ol (**2a**, 27 mg, 0.2 mmol). The reaction was conducted at 35 ℃ for 52 h. The crude product was purified by flash chromatography (4:1 petroleum ether/ethyl acetate) to afford a pale yellow solid. M.P. = 49 – 50 °C. **^{1}H NMR** and **^{13}C NMR** data are compatible with those reported in ref. 12.

First run: 24 mg (58%). **Second run**: 26 mg (63%).

^{1}H NMR (400 MHz, $CDCl_3$, TMS): δ 7.35-7.26 (m, 4H), 7.18 (t, *J* = 7.2 Hz, 1H), 7.02 (d, *J* = 8.0 Hz, 2H), 6.54 (d, *J* = 8.4 Hz, 2H), 6.41 (d, *J* = 16.0 Hz, 1H), 6.32 (d, *J* = 16.0 Hz, 6.8 Hz, 1H), 3.57 (brs, 2H), 3.43 (d, *J* = 6.4 Hz, 2H).

^{13}C NMR (100 MHz, $CDCl_3$): δ 144.8, 137.9, 130.7, 130.3, 130.2, 129.7, 128.7, 127.1, 126.3, 115.5, 38.7.

IR (cm^{-1}): 3361, 3024, 2918, 1621, 1515, 1276, 966, 825, 733, 692.

HRMS (ESI): $[M+H]^+$ calcd for $C_{15}H_{15}N$ 210.1277, found 210.1279.

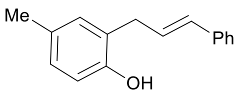

25

2-cinnamyl-4-methylphenol. The title compound was prepared according to the general procedure, using 2-bromo-4-methylphenol (**1aw**, 56 mg, 0.3 mmol) and (*E*)-3-phenylprop-2-en-1-ol (**2a**, 27 mg, 0.2mmol). Catalyst: Ni(diglyme)Br_2 (7.0 mg, 0.02 mmol). The reaction was conducted at 25 ℃ for 32 h. The crude product was purified by flash chromatography (5:1 petroleum ether/ethyl acetate) to afford a colorless oil.

First run: 23 mg (51%). **Second run**: 24 mg (53%)

^{1}H NMR (400 MHz, $CDCl_3$, TMS): δ 7.34 (d, *J* = 7.2 Hz, 2H), 7.27 (t, *J* = 8.0 Hz, 2H), 7.21-7.17 (m, 1H), 6.96-6.91 (m, 2H), 6.69 (d, *J* = 8.0 Hz, 1H), 6.48 (d, *J* = 15.6 Hz, 1H), 6.36 (dt, *J* = 16.0 Hz, 6.4 Hz, 1H), 4.18 (s, 1H), 3.51 (d, *J* = 6.4 Hz, 2H), 2.26 (s, 3H).

^{13}C NMR (100 MHz, $CDCl_3$): δ 151.9, 137.3, 131.5, 131.2, 130.0, 128.7, 128.4, 128.3, 127.4, 126.4, 125.6, 115.8, 34.2, 20.7.

IR (cm^{-1}): 3526, 3026, 1508, 1448, 1259, 1228, 1105, 967, 810, 747, 692.

HRMS (ESI): $[M+H]^+$ calcd. for $C_{16}H_{17}O$ 225.1274, found 225.1275.

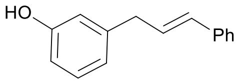

26

3-cinnamylphenol. The title compound was prepared according to the general procedure, using

3-bromophenol (**1ax**, 52 mg, 0.3 mmol) and (*E*)-3-phenylprop-2-en-1-ol (**2a**, 27 mg, 0.2mmol). Catalyst: Ni(diglyme)Br_2 (7.0 mg, 0.02 mmol). The reaction was conducted at 25 ℃ for 32 h. The crude product was purified by flash chromatography (5:1 petroleum ether/ethyl acetate) to afford a colorless oil.

First run: 37 mg (89%). **Second run**: 38 mg (91%)

^{1}H NMR (400 MHz, $CDCl_3$, TMS): δ 7.35 (d, *J* = 7.6 Hz, 2H), 7.29 (t, *J* =7.6 Hz, 2H), 7.23-7.15 (m, 2H), 6.81 (d, *J* = 7.6 Hz, 1H), 6.70-6.66 (m, 2H), 6.45 (d, *J* = 16.0 Hz, 1H), 6.32 (dt, *J* = 16.0 Hz, 6.8 Hz, 1H), 4.80 (s, 1H), 3.49 (d, *J* = 6.8 Hz, 2H).

^{13}C NMR (100 MHz, $CDCl_3$): δ 155.8, 142.3, 137.6, 131.5, 130.0, 129.0, 128.7, 127.3, 126.3, 121.4, 115.7, 113.3, 39.3.

IR (cm^{-1}): 3363, 3026, 1590, 1488, 1455, 1262, 1151, 966, 781, 754, 731,692.

HRMS (ESI): $[M+H]^+$ calcd. for $C_{15}H_{15}O$ 211.1117, found 211.1117.

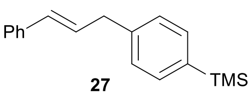

27

(4-Cinnamylphenyl)trimethylsilane. The title compound was prepared according to the general procedure, using (4-bromophenyl)trimethylsilane (**1ay**, 68 mg, 0.3 mmol) and (*E*)-3-phenylprop-2-en-1-ol (**2a**, 27 mg, 0.2 mmol). The reaction was conducted at 30 ℃ for 48 h. The crude product was purified by flash chromatography (100:0 petroleum ether/ethyl acetate) to afford a pale yellow oil.

First run: 36 mg (68%). **Second run**: 39 mg (74%).

^{1}H NMR (400 MHz, $CDCl_3$, TMS): δ 7.47 (d, *J* = 7.6 Hz, 2H), 7.35 (d, *J* = 7.2 Hz, 2H), 7.30-7.17 (m, 5H), 6.47 (d, *J* = 16.0 Hz, 1H), 6.35 (dt, *J* = 16.0 Hz, 6.8 Hz, 1H), 3.54 (d, *J* = 6.4 Hz, 2H), 0.26 (s, 9H).

^{13}C NMR (100 MHz, $CDCl_3$): δ 141.0, 138.1, 137.7, 133.8, 131.3, 129.3, 128.7, 128.3, 127.3, 126.3, 39.5, -0.9.

IR (cm^{-1}): 3027, 2955, 1600, 1396, 1248, 1108, 965, 839, 754, 692.

HRMS (EI): $[M^+]$ calcd for $C_{18}H_{22}Si$ 266.1491, found 266.1490.

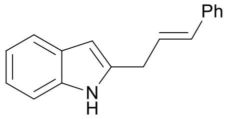

28

2-Cinnamyl-1*H*-indole (known compound). The title compound was prepared according to the general procedure, using 2-bromo-1H-indole (**1az**, 39 mg, 0.2 mmol) and (*E*)-3-phenylprop-2-en-1-ol (**2a**, 27 mg, 0.2 mmol). The reaction was conducted at 30 ℃ for 32 h. The crude product was purified by flash chromatography (10:1 petroleum ether/ethyl acetate) to afford a light yellow solid. M.P. = 88 – 89 ^{o}C. **^{1}H NMR** and **^{13}C NMR** data are compatible with those reported in ref. 13.

First run: 23 mg (49%). **Second run**: 25 mg (53%).

^{1}H NMR (400 MHz, $CDCl_3$, TMS): δ 7.96 (brs, 1H), 7.55 (d, *J* = 7.6 Hz, 1H), 7.40-7.29 (m, 5H), 7.22-7.24 (m, 1H), 7.13-7.06 (m, 2H), 6.57 (d, *J* = 16.0 Hz, 1H), 6.42-6.33 (m, 2H), 3.71(d, *J* = 6.8 Hz, 2H).

^{13}C NMR (100 MHz, $CDCl_3$): δ 137.1, 136.3, 132.5, 129.0, 128.8, 127.7, 126.5, 126.4, 121.5, 120.2, 119.9, 110.6, 100.6, 32.2.

IR (cm^{-1}): 3405, 3026, 2920, 1455, 1411, 966, 748, 700.

HRMS (ESI): $[M+H]^+$ calcd for $C_{17}H_{15}N$ 234.1277, found 234.1279.

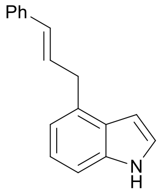

29

4-Cinnamyl-1*H*-indole. The title compound was prepared according to the general procedure, using 4-bromo-1H-indole (**1ba**, 39 mg, 0.2 mmol) and (*E*)-3-phenylprop-2-en-1-ol (**2a**, 27 mg, 0.2 mmol). Catalyst: Ni(diglyme)Br_2 (7.0 mg, 0.02 mmol). The reaction was conducted at 25 ℃ for 48 h. The crude product was purified by flash chromatography (10:1 petroleum ether/ethyl acetate) to afford a light yellow solid. M.P. = 81 – 82 ^{o}C.

First run: 37 mg (80%). **Second run**: 36 mg (78%).

^{1}H NMR (400 MHz, $CDCl_3$, TMS): δ 8.04 (brs, 1H), 7.33 (d, *J* = 7.6 Hz, 2H), 7.27-7.23 (m, 3H), 7.20-7.12 (m, 3H), 6.99 (dd, *J* = 7.2 Hz, 0.4 Hz, 1H), 6.63-6.61 (m, 1H), 6.55-6.43 (m, 2H), 3.82 (d, *J* = 5.6 Hz, 2H).

^{13}C NMR (100 MHz, $CDCl_3$): δ 137.9, 136.0, 132.5, 131.0, 129.4, 128.6, 127.5, 127.1, 126.3,

123.9, 122.4, 119.6, 109.5, 101.3, 37.2.

IR (cm^{-1}): 3417, 3026, 1497, 1435, 1341, 966, 752, 696.

HRMS (EI): $[M+H]^+$ calcd for $C_{17}H_{15}N$ 234.1277, found 234.1279.

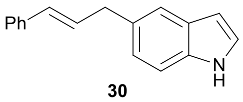

30

5-Cinnamyl-1*H*-indole. The title compound was prepared according to the general procedure, using 5-bromo-1H-indole (**1bb**, 39 mg, 0.2 mmol) and (*E*)-3-phenylprop-2-en-1-ol (**2a**, 27 mg, 0.2 mmol). Catalyst: Ni(diglyme)Br_2 (7.0 mg, 0.02 mmol). The reaction was conducted at 20 ℃ for 32 h. The crude product was purified by flash chromatography (10:1 petroleum ether/ethyl acetate) to afford a light yellow solid. M.P. = 60 – 61 ^oC.

First run: 39 mg (83%). **Second run**: 36 mg (78%).

^{1}H NMR (400 MHz, $CDCl_3$, TMS): δ 7.96 (brs, 1H), 7.49 (s, 1H), 7.35 (d, *J* = 7.2 Hz, 2H), 7.29-7.25 (m, 3H), 7.20-7.06 (m, 3H), 6.49-6.38 (m, 3H), 3.63 (d, *J* = 5.6 Hz, 2H).

^{13}C NMR (100 MHz, $CDCl_3$): δ 137.9, 134.7, 131.6, 130.7, 130.5, 128.6, 128.3, 127.1, 126.3, 124.6, 123.4, 120.4, 111.1, 102.5, 39.6.

IR (cm^{-1}): 3417, 3025, 1623, 1494, 1452, 1343, 1090, 967, 754, 725, 693.

HRMS (ESI): $[M+H]^+$ calcd for $C_{17}H_{15}N$ 234.1277, found 234.1279.

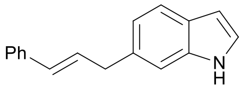

31

6-Cinnamyl-1*H*-indole. The title compound was prepared according to the general procedure, using 6-bromo-1H-indole (**1bc**, 39 mg, 0.2 mmol) and (*E*)-3-phenylprop-2-en-1-ol (**2a**, 27 mg, 0.2 mmol). Catalyst: Ni(diglyme)Br_2 (7.0 mg, 0.02 mmol). The reaction was conducted at 25 ℃ for 48 h. The crude product was purified by flash chromatography (10:1 petroleum ether/ethyl acetate) to afford a light yellow solid. M.P. = 89 – 90 ^oC.

First run: 34 mg (73%). **Second run**: 35 mg (75%).

^{1}H NMR (400 MHz, $CDCl_3$, TMS): δ 7.97 (brs, 1H), 7.57 (d, *J* = 8.0 Hz, 1H), 7.36 (d, *J* = 7.2 Hz, 2H), 7.28 (t, *J* = 8.0 Hz, 2H), 7.20-7.16 (m, 2H), 7.12 (t, *J* = 2.8 Hz, 1H), 7.01 (dd, *J* = 8.0 Hz, 1.2 Hz, 1H), 6.52-6.38 (m, 3H), 3.65 (d, *J* = 5.6 Hz, 2H).

^{13}C NMR (100 MHz, $CDCl_3$): δ 137.8, 136.4, 134.3, 130.8, 130.2, 128.7, 127.2, 126.5, 126.3, 124.1, 121.4, 120.8, 110.9, 102.6, 39.7.

IR (cm^{-1}): 3417, 3025, 1623, 1494, 1452, 1343, 1091, 967, 725, 692.

HRMS (ESI): $[M+H]^+$ calcd for $C_{17}H_{15}N$ 234.1277, found 234.1274.

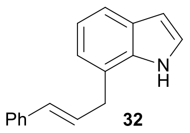

32

7-Cinnamyl-1*H*-indole (known compound). The title compound was prepared according to the general procedure, using 7-bromo-1H-indole (**1bd**, 39 mg, 0.2 mmol) and (*E*)-3-phenylprop-2-en-1-ol (**2a**, 27 mg, 0.2 mmol). Catalyst: Ni(diglyme)Br_2 (7.0 mg, 0.02 mmol). The reaction was conducted at 20 ℃ for 32 h. The crude product was purified by flash chromatography (10:1 petroleum ether/ethyl acetate) to afford a light yellow solid. M.P. = 54 – 55 ^oC. **^{1}H NMR** and **^{13}C NMR** data are compatible with those reported in ref. 14.

First run: 43 mg (93%). **Second run**: 44 mg (95%).

^{1}H NMR (400 MHz, $CDCl_3$, TMS): δ 8.18 (brs, 1H), 7.56 (d, J = 7.2 Hz, 1H), 7.35-7.33 (m, 2H), 7.30-7.27 (m, 2H), 7.23-7.19 (m, 1H), 7.14-7.05 (m, 3H), 6.60-6.54 (m, 2H), 6.45(dt, J = 16.0 Hz, 6.4 Hz, 1H), 3.78 (d, J = 6.4 Hz, 2H).

^{13}C NMR (100 MHz, $CDCl_3$): δ 137.2, 135.4, 131.5, 128.8, 128.6, 128.2, 127.6, 126.4, 124.3, 122.4, 122.3, 120.2, 119.5, 103.1, 36.1.

IR (cm^{-1}): 3426, 3055, 3026, 1492, 1433, 1410, 1345, 1105, 1067, 968, 730.

HRMS (ESI): $[M+H]^+$ calcd for $C_{17}H_{15}N$ 234.1277, found 234.1278.

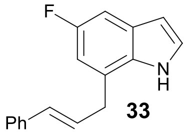

33

7-cinnamyl-5-fluoro-1H-indole. The title compound was prepared according to the general procedure, using 7-bromo-5-fluoro-1H-indole (**1be**, 43 mg, 0.2 mmol) and (*E*)-3-phenylprop-2-en-1-ol (**2a**, 27 mg, 0.2 mmol). Catalyst: Ni(diglyme)Br_2 (7.0 mg, 0.02 mmol), bpy (6.2 mg, 0.04 mmol), $ZrCl_4$ (4.7 mg, 0.02 mmol). The reaction was conducted at 23 ℃ for 48 h. The crude product was purified by flash chromatography (4:1 petroleum ether/ethyl acetate) to afford a colorless oil.

First run: 22 mg (44%). **Second run**: 23 mg (46%).

^{1}H NMR (400 MHz, $CDCl_3$, TMS): δ 8.13 (brs, 1H), 7.34-7.26 (m, 4H), 7.23-7.14 (m, 3H),

6.88 (d, J = 8.0 Hz, 1H), 6.56 (d, J = 16.0 Hz, 1H), 6.50-6.49 (m, 1H), 6.38 (dt, J = 16.0 Hz, 6.8 Hz, 1H), 3.71 (d, J = 8.0 Hz, 2H).

^{13}C NMR (100 MHz, CDCl$_3$): δ 158.1(d, $^{1}J_{CF}$ = 233 Hz), 137.0, 132.1, 131.8, 128.8, 128.3 (d, $^{3}J_{CF}$ = 11 Hz), 127.7, 127.4, 126.4, 125.8, 123.5 (d, $^{3}J_{CF}$ = 9 Hz), 110.7 (d, $^{2}J_{CF}$ = 27 Hz), 103.9 (d, $^{2}J_{CF}$ = 23 Hz), 103.2 (d, $^{4}J_{CF}$ = 5 Hz), 35.6.

^{19}F NMR (376 MHz, CDCl$_3$): δ -124.72.

IR (cm^{-1}): 3430, 3026, 2917, 2849, 1593, 1485, 1428, 1305, 1121, 967, 847, 791, 727, 694.

HRMS (ESI): $[M+H]^+$ calcd. for $C_{17}H_{15}FN$ 252.1183, found 252.1180.

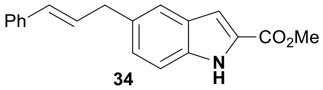

34

Methyl 5-cinnamyl-1H-indole-2-carboxylate. The title compound was prepared according to the general procedure, using methyl 5-bromo-1H-indole-2-carboxylate (**1bf**, 76 mg, 0.3 mmol) and (*E*)-3-phenylprop-2-en-1-ol (**2a**, 27 mg, 0.2 mmol). Catalyst: Ni(diglyme)Br$_2$ (7.0 mg, 0.02 mmol), bpy (6.2 mg, 0.04 mmol), ZrCl$_4$ (4.7 mg, 0.02 mmol). The reaction was conducted at 23 ℃ for 48 h. The crude product was purified by flash chromatography (4:1 petroleum ether/ethyl acetate) to afford a white solid.

First run: 40 mg (69 %). **Second run**: 41 mg (70 %), M.P. = 140 – 141 ^{o}C

^{1}H NMR (400 MHz, CDCl$_3$, TMS): δ 8.97 (brs, 1H), 7.54 (s, 1H), 7.38-7.36 (m, 3H), 7.29 (t, J = 7.6 Hz, 2H), 7.25-7.17 (m, 3H), 6.48 (d, J = 16.0 Hz, 1H), 6.40 (dt, J = 16.0 Hz, 6.4 Hz, 1H), 3.95 (s, 3H), 3.64 (d, J = 6.0 Hz, 2H).

^{13}C NMR (100 MHz, CDCl$_3$): δ 162.6, 137.6, 135.8, 132.7, 130.9, 129.9, 128.6, 127.9, 127.4, 127.2, 127.0, 126.2, 121.9, 112.0, 108.6, 52.1, 39.4.

IR (cm^{-1}): 3331, 3024, 2917, 2849, 1694, 1529, 1436, 1253, 1207, 767, 737, 691.

HRMS (ESI): $[M+H]^+$ calcd. for $C_{19}H_{18}NO_2$ 292.1332, found 292.1327.

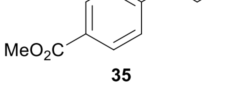

35

Methyl 4-allylbenzoate (known compound). The title compound was prepared according to the general procedure, using methyl 4-bromobenzoate (**1am**, 85 mg, 0.4 mmol) and prop-2-en-1-ol (**2b**, 12 mg, 0.2 mmol). The reaction was conducted at 35 ℃ for 50 h. The crude product was purified by flash chromatography (30:1 petroleum ether/ethyl acetate) to afford a colorless oil. **^{1}H NMR** and **^{13}C**

NMR data are compatible with those reported in ref. 15.

First run: 27 mg (78%). **Second run**: 29 mg (81%).

^{1}H NMR (400 MHz, $CDCl_3$, TMS): δ 7.97 (d, *J* = 8.4 Hz, 2H), 7.26 (d, *J* = 8.4 Hz, 2H), 6.01-5.91 (m, 1H), 5.12-5.07 (m, 2H), 3.90 (s, 3H), 3.44 (d, *J* = 6.8 Hz, 2H).

^{13}C NMR (100 MHz, $CDCl_3$): δ 167.3, 145.7, 136.6, 130.0, 128.8, 128.3, 116.8, 52.2, 40.3.

IR (cm^{-1}): 3057, 2953, 1720, 1612, 1436, 1282, 1179, 1111, 919, 739, 706.

HRMS (ESI): $[M+H]^+$ calcd for $C_{11}H_{12}O_2$ 177.0910, found 177.0908 .

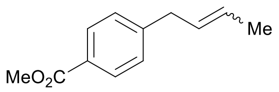

36

Methyl 4-(but-2-en-1-yl)benzoate. The title compound was prepared according to the general procedure, using methyl 4-bromobenzoate (**1am**, 64 mg, 0.3 mmol) and (*E*)-but-2-en-1-ol (**2c**, 14 mg, 0.2 mmol). Catalytic conditions: Ni(dppf)Cl_2 (13 mg, 0.02 mmol), 3,4,7,8-tetramethyl-1,10-phenanthroline (9 mg, 0.04 mmol), $AlCl_3$ (5 mg, 0.04 mmol). The reaction was conducted at 35 ℃ for 50 h. The crude product was purified by flash chromatography (30:1 petroleum ether/ethyl acetate) to afford a light yellow oil.

Total yields of linear/branched (4:1) isomers: **First run**: 24 mg (64%). **Second run**: 22 mg (59%).

Linear products (*E*/*Z* = 3.2/1 isomers) [15]

^{1}H NMR (400 MHz, $CDCl_3$, TMS): δ 7.96 (d, *J* = 8.4 Hz, 2H), 7.24 (d, *J* = 8.4 Hz, 2H), 5.66-5.49 (m, 2H), 3.90 (s, 3H), [3.45 (*Z*), d, *J* = 6.8 Hz; 3.36 (*E*), d, *J* = 5.2 Hz; 2 H)], [1.73 (*Z*), d, *J* = 6.4 Hz; 1.69 (*E*), d, *J* = 4.8 Hz, 3 H)].

Branched isomer.

^{1}H NMR (400 MHz, $CDCl_3$, TMS): δ 7.96 (d, *J* = 8.4 Hz, 2H), 7.24 (d, *J* = 8.4 Hz, 2H), 6.03-5.94 (m, 1H), 5.08-5.04 (m, 2H), 3.90 (s, 3H), 3.54-3.51 (m, 1H), 1.37 (d, *J* = 6.8 Hz, 3H).

Linear and branched isomers, ^{13}C NMR (100 MHz, $CDCl_3$): δ 167.3, 146.7, 142.5, 130.0, 129.93, 129.88, 129.2, 128.7, 128.5, 128.0, 127.5, 127.4, 125.9, 114.1, 52.2, 43.4, 39.2, 33.3, 20.7, 18.1, 13.1.

IR (cm^{-1}): 3024, 2952, 1722, 1610, 1435, 1280, 1178, 1109, 1019, 967, 762.

HRMS (ESI): $[M+H]^+$ calcd for $C_{12}H_{15}O_2$ 191.1067, found 191.1064.

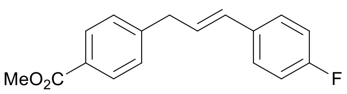

37

Methyl (*E*)-4-(3-(4-fluorophenyl)allyl)benzoate. The title compound was prepared according to the general procedure, using methyl 4-bromobenzoate (**1am**, 85 mg, 0.4 mmol) and (*E*)-3-(4-fluorophenyl)prop-2-en-1-ol (**2d**, 30 mg, 0.2 mmol). The reaction was conducted at 30 ℃ for 48 h. The crude product was purified by flash chromatography (30:1 petroleum ether/ethyl acetate) to afford a colorless oil.

First run: 42 mg (77%). **Second run**: 40 mg (73%).

^{1}H NMR (400 MHz, $CDCl_3$, TMS): δ 7.98 (d, J = 8.0 Hz, 2H), 7.33-7.29 (m, 4H), 6.98 (t, J = 8.8 Hz, 2H), 6.42 (d, J = 16.0 Hz, 1H), 6.24 (dt, J = 15.6 Hz, 6.8 Hz, 1H), 3.91 (s, 3H), 3.58 (d, J = 6.8 Hz, 2H).

^{13}C NMR (100 MHz, $CDCl_3$): δ 167.2, 162.4 (d, $^{1}J_{CF}$ = 245 Hz), 145.7, 133.5 (d, $^{4}J_{CF}$ = 3 Hz), 130.8, 130.0, 128.8, 128.4, 128.0 (d, $^{5}J_{CF}$ = 2 Hz), 127.8 (d, $^{3}J_{CF}$ = 8 Hz), 115.7 (d, $^{2}J_{CF}$ = 22 Hz), 52.2, 39.4.

^{19}F NMR (376 MHz, $CDCl_3$): δ -114.93.

IR (cm^{-1}): 3033, 2952, 1720, 1609, 1509, 1435, 1281, 1228, 1178, 1110, 968, 840, 761.

HRMS (ESI): $[M+H]^+$ calcd for $C_{17}H_{15}F_1O_2$ 271.1129, found 271.1125 .

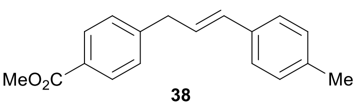

38

(*E*)-3-(p-tolyl)prop-2-en-1-ol. The title compound was prepared according to the general procedure, using methyl 4-bromobenzoate (**1am**, 85 mg, 0.4 mmol) and (*E*)-3-(p-tolyl)prop-2-en-1-ol (**2e**, 30 mg, 0.2 mmol). The reaction was conducted at 35 ℃ for 48 h. The crude product was purified by flash chromatography (30:1 petroleum ether/ethyl acetate) to afford a light yellow oil.

First run: 37 mg (70%). **Second run**: 40 mg (75%).

^{1}H NMR (400 MHz, $CDCl_3$, TMS): δ 7.97 (dd, J = 8.0 Hz, 0.8 Hz, 2H), 7.29 (d, J = 8.4 Hz, 2H), 7.24 (d, J = 8.0 Hz, 2H), 7.09 (d, J = 8.0 Hz, 2H), 6.42 (d, J = 15.6 Hz, 1H), 6.30-6.22 (dt, J = 15.6 Hz, 6.8 Hz, 1H), 3.89 (s, 3H), 3.56 (d, J = 6.8 Hz, 2H), 2.31(s, 3H).

^{13}C NMR (100 MHz, $CDCl_3$): δ 167.2, 146.0, 137.2, 134.6, 131.8, 130.0, 129.4, 128.8, 128.3, 127.1, 126.2, 52.1, 39.4, 21.3.

IR (cm^{-1}): 3024, 2951, 1720, 1610, 1511, 1435, 1280, 1178, 1110, 1020, 968, 809, 766.

HRMS (ESI): $[M+H]^+$ calcd for $C_{18}H_{19}O_2$ 267.1380, found 267.1378.

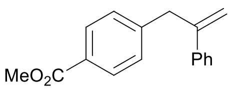

39

2-Phenylprop-2-en-1-ol (known compound). The title compound was prepared according to the general procedure, using methyl 4-bromobenzoate (**1am**, 85 mg, 0.4 mmol) and 2-phenylprop-2-en-1-ol (**2f**, 27 mg, 0.2 mmol). The reaction was conducted at 35 ℃ for 48 h. The crude product was purified by flash chromatography (30:1 petroleum ether/ethyl acetate) to afford a light yellow oil. **^{1}H NMR** and **^{13}C NMR** data are compatible with those reported in ref. 16.

First run: 30 mg (60%). **Second run**: 29 mg (57%).

^{1}H NMR (400 MHz, $CDCl_3$, TMS): δ 7.93 (d, J = 8.4 Hz, 2H), 7.38 (d, J = 7.6 Hz, 2H), 7.29-7.21 (m, 5H), 5.50 (s, 1H), 5.03 (s, 1H), 3.86 (s, 5H).

^{13}C NMR (100 MHz, $CDCl_3$): δ 167.2, 146.3, 145.2, 140.5, 129.8, 129.1, 128.5, 128.3, 127.8, 126.2, 115.2, 52.1, 41.8.

IR (cm^{-1}): 3030, 2951, 1721, 1611, 1435, 1281, 1178, 1110, 1021, 903.

HRMS (ESI): $[M+H]^+$ calcd for $C_{17}H_{16}O_2$ 253.1223, found 253.1221 .

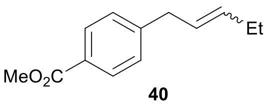

40

Methyl 4-(pent-2-en-1-yl)benzoate. The title compound was prepared according to the general procedure, using methyl 4-bromobenzoate (**1am**, 64 mg, 0.3 mmol) and pent-1-en-3-ol (**2g**, 17 mg, 0.2 mmol). Catalytic conditions: Ni(dppf)Cl_2 (13 mg, 0.02 mmol), 3,4,7,8-tetramethyl-1,10-phenanthroline (9 mg, 0.04 mmol), $AlCl_3$ (5 mg, 0.04 mmol). The reaction was conducted at 35 ℃ for 50 h. The crude product was purified by flash chromatography (30:1 petroleum ether/ethyl acetate) to afford a light yellow oil.

Total yields of linear/branched (11:1) isomers: **First run**: 25 mg (61%). **Second run**: 26 mg (64%).

^{1}H NMR [400 MHz, $CDCl_3$, TMS, Linear isomers (*E*/*Z* = 5/1)]: δ 7.96 (d, J = 8.4 Hz, 2H), 7.25 (d, J = 8.4 Hz, 2H), 5.61-5.50 (m, 2H), 3.90 (s, 3H), [3.44 (*Z*), d, J = 6.8 Hz; 3.37 (*E*), d, J = 4.8 Hz; 2 H)], [2.20-2.12 (*Z*), m; 2.06-2.03 (*E*), m, 2 H)], 0.99 (t, J = 7.2 Hz, 3H).

^{1}H NMR (400 MHz, $CDCl_3$, TMS, branched isomer): δ 7.96 (d, J = 8.4 Hz, 2H), 7.25 (d, J = 8.4 Hz, 2H), 5.96-5.89 (m, 1H), 5.07-5.02 (m, 2H), 3.90 (s, 3H), 3.23-3.17 (m, 1H), 1.77-1.72 (m,

2H), 0.86 (t, *J* = 7.6 Hz, 3H).

^{13}C NMR (100 MHz, $CDCl_3$, linear and branched isomers): δ 167.3, 146.8, 141.5, 134.6, 133.6, 129.9, 128.7, 128.5, 128.0, 127.9, 126.9, 126.5, 52.2, 39.2, 33.6, 28.4, 25.7, 14.4, 13.9, 12.2.

IR (cm^{-1}): 3030, 2962, 1723, 1611, 1435, 1280, 1178, 1111, 1020, 968, 761, 708.

HRMS (ESI): $[M+H]^+$ calcd for $C_{13}H_{16}O_2$ 205.1223, found 205.1220.

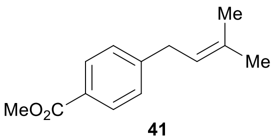

41

Methyl 4-(3-methylbut-2-en-1-yl)benzoate. The title compound was prepared according to the general procedure, using methyl 4-bromobenzoate (**1am**, 64 mg, 0.3 mmol) and 2-methylbut-3-en-2-ol (**2h**, 17 mg, 0.2 mmol). Catalytic conditions: Ni(dppf)Cl_2 (13 mg, 0.02 mmol), 3,4,7,8-tetramethyl-1,10-phenanthroline (9 mg, 0.04 mmol), $AlCl_3$ (5 mg, 0.04 mmol). The reaction was conducted at 35 ℃ for 50 h. The crude product was purified by flash chromatography (30:1 petroleum ether/ethyl acetate) to afford a light yellow oil. linear/branched (180:1).

First run: 27 mg (67%). **Second run**: 26 mg (65%).

^{1}H NMR (400 MHz, $CDCl_3$, TMS): ^{1}H NMR (400 MHz, $CDCl_3$, TMS): δ 7.95 (d, *J* = 8.0 Hz, 2H), 7.24 (d, *J* = 8.0 Hz, 2H), 5.32-5.28 (m, 1H), 3.90 (s, 3H), 3.39 (d, *J* = 7.2 Hz, 2H), 1.76 (s, 3H), 1.72 (s, 3H).

^{13}C NMR (100 MHz, $CDCl_3$): δ 167.4, 147.6, 133.7, 129.9, 128.5, 127.9, 122.3, 52.1, 34.6, 25.9, 18.0.

IR (cm^{-1}): 2915, 1722, 1611, 1435, 1280, 1177, 1110, 1020, 758.

HRMS (ESI): $[M+H]^+$ calcd for $C_{13}H_{16}O_2$ 205.1223, found 205.1220.

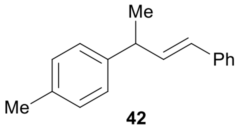

42

(*E*)-1-methyl-4-(4-phenylbut-3-en-2-yl)benzene (known compound).

Reaction of **1aa** with **2j**: The title compound was prepared according to the general procedure, using 1-bromo-4-methylbenzene (**1aa**, 51 mg, 0.3 mmol) and (*E*)-4-phenylbut-3-en-2-ol (**2j**, 30 mg, 0.2 mmol). Catalytic conditions: Ni(dppp)Cl_2 (16.2 mg, 0.03 mmol), bpy (9.3 mg, 0.06 mmol), $ZrCl_4$ (7.1 mg, 0.03 mmol). The reaction was conducted at 30 ℃ for 48 h. The crude product was purified

by flash chromatography (100:0 petroleum ether/ethyl acetate) to afford a light yellow oil.

First run: 24 mg (53%). **Second run**: 26 mg (58%).

Reaction of **1aa** with **2k**: The title compound was prepared according to the general procedure, using 1-bromo-4-methylbenzene (**1aa**, 51 mg, 0.3 mmol) and (*E*)-1-phenylbut-2-en-1-ol (**2k**, 30 mg, 0.2 mmol). The reaction was conducted at 30 ℃ for 48 h. The crude product was purified by flash chromatography (100:0 petroleum ether/ethyl acetate) to afford a light yellow oil.

^{1}H NMR and **^{13}C NMR** data are compatible with those reported in ref. 17.

First run: 27 mg (60%). **Second run**: 26 mg (59%).

^{1}H NMR (400 MHz, $CDCl_3$, TMS): δ 7.34 (d, *J* = 7.2 Hz, 2H), 7.28 (d, *J* = 7.2 Hz, 2H), 7.23-7.11 (m, 5H), 6.43-6.33 (m, 2H), 3.63-3.57 (m, 1H), 2.32 (s, 3H), 1.44 (d, *J* = 6.8 Hz, 3H).

^{13}C NMR (100 MHz, $CDCl_3$): δ 142.8, 137.8, 135.9, 135.7, 129.4, 128.7, 128.5, 127.4, 127.2, 126.3, 42.3, 21.5, 21.2.

IR (cm^{-1}): 3024, 2965, 1598, 1513, 1494, 1448, 1371, 1014, 965, 815, 751, 692.

GC-MS (EI) m/z (rel intensity, ion): 222.11 (51.92, M^+).

6. Sequence Reactions in *Eq.*1 and 2

6.1 Synthesis of compound 44

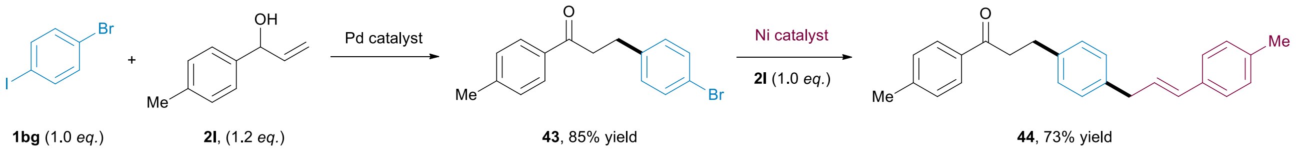

A reaction tube charged with $Pd(OAc)_2$ (44.8 mg, 0.2 mmol), tetrabutylammonium chloride (1.11 g, 4.0 mmol), sodium bicarbonate (0.84 g, 8.0 mmol) and DMF (20mL) was vacuumed and back-filled with Ar for 3 times. The reaction mixture was stirred at room temperature for 5 minutes. 1-Bromo-4-iodobenzene (1.13 g, 4.0 mmol) and 1-(p-tolyl)prop-2-en-1-ol (0.71 g, 4.8 mmol) were then added. After stirring at 40 ^{o}C for 12 hours, the resulting dark reaction mixture was cooled to room temperature, diluted with EtOAc (50 mL) and water (50 mL), and separated. The aqueous layer was extracted with diethyl ether (3 × 50 mL). The combined organic solution was washed with saturated brine (50 mL), dried with anhydrous Na_2SO_4 and concentrated under reduced pressure. The residue is purified by silica gel column (PE/EA = 25/1) to give compound **43** (1.02 g, 85% yield) as a white solid. [18]

Known compound, M.P. = 80 – 81 ^{o}C. **^{1}H NMR** and **^{13}C NMR** data are compatible with those reported in ref. 19.

^{1}H NMR (400 MHz, $CDCl_3$, TMS): δ 7.87 (d, *J* = 8.0 Hz, 2H), 7.43 (d, *J* = 8.0 Hz, 2H), 7.27 (d, *J* = 8.0 Hz, 2H), 7.15 (d, *J* = 8.4 Hz, 2H), 3.27 (t, *J* = 7.2 Hz, 2H), 3.04 (t, *J* = 7.6 Hz, 2H), 2.43(s, 3H).

^{13}C NMR (100 MHz, $CDCl_3$): δ 198.6, 144.2, 140.6, 134.5, 131.7, 130.4, 129.5, 128.3, 120.0, 40.1, 29.7, 21.8.

IR (cm^{-1}): 2918, 1671, 1606, 1485, 1425, 807.

GC-MS (EI) m/z (rel intensity, ion): 301.98 (100, M^+).

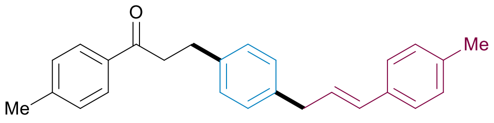

44

(*E*)-1-(p-tolyl)-3-(4-(3-(p-tolyl)allyl)phenyl)propan-1-one. The title compound was prepared according to the General Procedure, using compound **43** (60 mg, 0.2 mmol) and 1-(p-tolyl)prop-2-en-1-ol (**2l**, 30 mg, 0.2 mmol). The reaction was conducted at 30 ℃ for 48 h. The

crude product was purified by flash chromatography (20:1 petroleum ether/ethyl acetate) to afford a light yellow solid. M.P. = 69 – 70 °C. 52 mg, 73% yield.

^{1}H NMR (400 MHz, $CDCl_3$, TMS): δ 7.85 (d, *J* = 8.4 Hz, 2H), 7.25-7.22 (m, 4H), 7.20-7.15 (m, 4H), 7.08 (d, *J* = 8.0 Hz, 2H), 6.41 (d, *J* = 15.6 Hz, 1H), 6.27 (dt, *J* = 15.6 Hz, 6.8 Hz, 1H), 3.49 (d, *J* = 6.8 Hz, 2H), 3.24 (t, *J* = 8.4 Hz, 2H), 3.02 (d, *J* = 8.0 Hz, 2H), 2.39 (s, 3H), 2.31 (s, 3H).

^{13}C NMR (100 MHz, $CDCl_3$): δ 199.1, 143.9, 139.4, 138.3, 137.0, 134.9, 134.6, 131.1, 129.43, 129.35, 129.0, 128.7, 128.5, 128.3, 126.2, 40.6, 39.1, 30.0, 21.8, 21.3.

IR (cm^{-1}): 3024, 2920, 1682, 1607, 1512, 1409, 1180, 971, 811.

HRMS (EI): $[M+H]^+$ calcd for $C_{26}H_{26}O$ 355.2066, found 355.2061.

6.2 Synthesis of compound 46

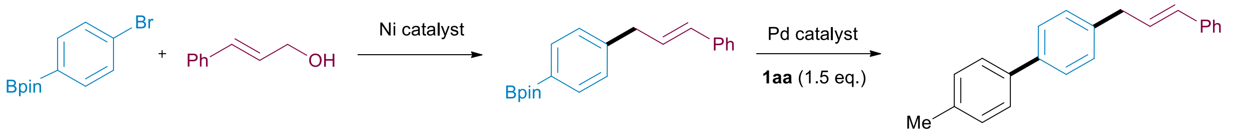

1bh, 1.0 eq. **2a** (1.0 eq.) **45**, 62% yield **46**, 68% yield

(*E*)-2-(4-cinnamylphenyl)-4,4,5,5-tetramethyl-1,3,2-dioxaborolane (45). The title compound was prepared according to the General Procedure, using 2-(4-bromophenyl)-4,4,5,5-tetramethyl-1,3,2-dioxaborolane (**1bh**, 141 mg, 0.5 mmol) and (*E*)-3-phenylprop-2-en-1-ol (**2a**, 67 mg, 0.5 mmol). The reaction was conducted at 30 ℃ for 48 h. The crude product was purified by flash chromatography (20:1 petroleum ether/ethyl acetate) to afford a light yellow solid. M.P. = 92 – 93 °C. 99 mg, 62% yield.

^{1}H NMR (400 MHz, $CDCl_3$, TMS): δ 7.76 (d, *J* = 8.0 Hz, 2H), 7.35 (d, *J* = 7.2 Hz, 2H), 7.30-7.25 (m, 4H), 7.20 (t, *J* = 7.2 Hz, 1H), 6.48 (d, *J* = 15.6 Hz, 1H), 6.34 (dt, *J* = 15.6 Hz, 6.8Hz, 1H), 3.57 (d, *J* = 6.8 Hz, 2H), 1.34 (s, 12H).

^{13}C NMR (100 MHz, $CDCl_3$): δ 143.7, 137.7, 135.2, 131.4, 129.1, 128.7, 128.3, 127.3, 126.3, 83.9, 39.7, 25.1.

^{11}B NMR (192 MHz, $CDCl_3$): 30.95

IR (cm^{-1}): 3026, 2978, 1611, 1399, 1360, 1273, 1144, 1090, 963, 859, 739, 658.

HRMS (EI): $[M+H]^+$ calcd for $C_{21}H_{25}BO_2$ 321.2020, found 321.2022.

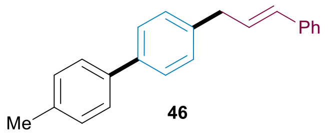

46

4-cinnamyl-4'-methyl-1,1'-biphenyl (46)

A tube charged with $Pd(PPh_3)_4$ (17 mg, 0.015 mmol), potassium carbonate (82 mg, 0.59 mmol), **45** (96 mg, 0.3 mmol), 1-bromo-4-methylbenzene **1aa** (76 mg, 0.45 mmol), DMF (5 mL) and H_2O (0.25 mL) was vacuumed and back-filled with Ar for 3 times. After stirring at 80 ^oC for 16 hours, the reaction mixture was quenched with water (20 mL) and extracted with EtOAc (3 × 20 mL). The combined organic mixture was washed with saturated brine (30 mL), dried over anhydrous Na_2SO_4 and concentrated under reduced pressure. The residue was purified by silica gel column (PE/EA=100/1) to give compound **46** (58 mg, 68% yield) as a white solid.[20] M.P. = 56 – 57 ^oC.

^{1}H NMR (400 MHz, $CDCl_3$, TMS): δ 7.53-7.47 (m, 4H), 7.37 (d, *J* = 7.2 Hz, 2H), 7.30 (d, *J* = 7.2 Hz, 4H), 7.24-7.20 (m, 3H), 6.49 (d, *J* = 16.0 Hz, 1H), 6.38 (dt, *J* = 15.6 Hz, 6.8 Hz, 1H), 3.58 (d, *J* = 6.8 Hz, 2H), 2.38 (s, 3H).

^{13}C NMR (100 MHz, $CDCl_3$): δ 139.3, 139.2, 138.4, 137.7, 137.0, 131.4, 129.7, 129.3, 129.2, 128.7, 127.31, 127.25, 127.1, 126.3, 39.2, 21.3.

IR (cm^{-1}): 3024, 1498, 966, 809, 745, 692.

GC-MS (EI) m/z (rel intensity, ion): 284.07 (100, M^+).

7. Monitoring of the Reaction of 1aa and 2j by GC Analysis

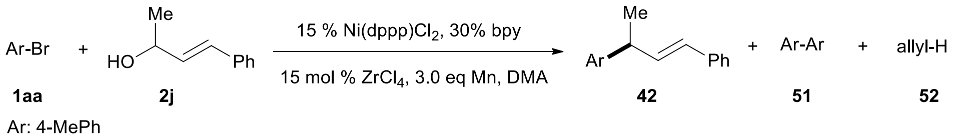

The reaction worked in an argon-filled glove box.

Reaction in the presence of $ZrCl_4$: To a reaction tube charged with $Ni(dppp)Cl_2$ (12.2 mg, 0.03 mmol), bpy (9.3 mg, 0.06 mmol), $ZrCl_4$ (7.1 mg, 0.03 mmol) and Mn (32.9 mg, 0.6 mmol) was added a solution of 1-bromo-4-methylbenzene (**1aa**, 51 mg, 0.3 mmol) and (*E*)-4-phenylbut-3-en-2-ol (**2j**, 30 mg, 0.2 mmol) in DMA (2 mL, 0.1 M). The reaction mixture was stirred at room temperature. A 50 μL of the reaction mixture was removed with a pipette in every 10 to 30 min. It was quenched with 50 μL of H_2O, diluted with diethyl ether (1 mL), and filtered through a syringe filter. The filtrate was analyzed by GC and the yield was calculated versus the internal standard (dodecane).

Reaction in the absence of $ZrCl_4$: above procedure, but no $ZrCl_4$ was used.

8. Procedure for experiments in Scheme 4.

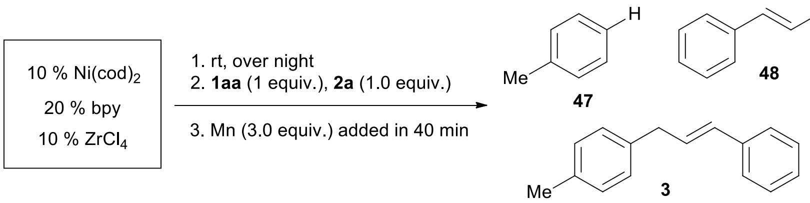

The reaction worked in an argon-filled glove box. To a reaction tube charged with bpy (6.2 mg, 0.04 mmol), $Ni(cod)_2$ (5.5 mg, 0.02 mmol), $ZrCl_4$ (4.7 mg, 0.02 mmol) was added DMA (1 mL). The reaction mixture was stirred at room temperature overnight. A solution of (*E*)-3-phenylprop-2-en-1-ol (**2a**, 27 mg, 0.2mmol) and 4-bromotoluene (**1aa**, 34 mg, 0.2 mmol) in DMA (1 mL) was then added. A 100 μL of the reaction mixture was removed with a pipette in every 10 min. Mn powder (33mg, 3.0 eq.) was added in 40 min. The removed reaction mixture was quenched with water, diluted with diethyl ether (1 mL), and filtered through a syringe filter. The filtrate was analyzed by GC and the yield was calculated versus the internal standard (dodecane).

9. Synthesis and Reactions of Ar-Ni^{II}(bpy)Br 49 and (bpy)Ni^{0}(cod) 50

9.1 Synthesis of complex 49

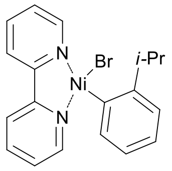

49

The reaction worked in an argon-filled glove box. To a flame-dried round-bottomed flask was added bpy (156 mg, 1.0 mmol), Ni(cod)$_2$ (275 mg, 1.0 mmol) and THF (40 mL). After the reaction mixture was stirred at room temperature for overnight, 1-bromo-2-isopropylbenzene (239 mg, 1.2 mmol) was added and the color changed from dark purple to red. After stirring at room temperature for 4h, the mixture solution was concentrated under reduced pressure. The solid was washed with dry *n*-pentane for several times and then dried under vacuum for 2 h to give complex **49** (330 mg, 80% yield) as a red solid.[21] The X-Ray quality crystals were obtained by slow diffusion of pentane into a toluene solution of **49** at -10 ^{o}C for 3 days.

^{1}H NMR (400 MHz, acetone-*d*6, TMS): δ 9.46 (d, *J* = 5.2 Hz, 1H), 8.38 (t, *J* = 8.0 Hz, 2H), 8.23-8.16 (m, 2H), 7.73 (t, *J* = 6.4 Hz, 1H), 7.54 (d, *J* = 7.2 Hz, 1H), 7.39 (t, *J* = 6.8 Hz, 1H), 7.19 (d, *J* = 5.6 Hz, 1H), 6.87 (d, *J* = 6.8 Hz, 1H), 6.81-6.74 (m, 2H), 5.27-5.20 (m, 1H), 1.36 (d, *J* = 6.8 Hz, 3H), 1.16 (d, *J* = 6.8 Hz, 3H).

^{13}C NMR (150 MHz, acetone-*d*6): δ 156.9, 153.9, 153.7, 151.9, 151.6, 148.6, 140.2, 139.6, 137.1, 127.3, 124.3, 123.9, 123.5, 122.7, 122.0, 37.9, 24.8, 24.2.

IR (cm^{-1}): 3104, 3046, 2957, 2925, 2863, 1602, 1443, 1260, 1024, 763, 736.

Anal. Cald for $C_{19}H_{19}N_2NiBr$: 55.13% C, 4.63% H, 6.77% N; found 54.98% C, 5.05% H, 5.74% N.

X-Ray crystallographic data

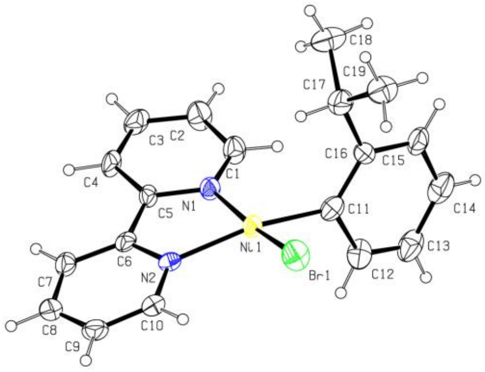

Table 1 Crystal data and structure refinement for **49** (CCDC 1515176).

Identification code	**49**
Empirical formula	$C_{19}H_{19}BrN_2Ni$
Formula weight	413.98
Temperature/K	293(2)
Crystal system	monoclinic
Space group	$P2_1/c$
a/Å	8.4028(11)
b/Å	11.9498(9)
c/Å	17.1489(17)
α/°	90.00
β/°	94.163(10)
γ/°	90.00
Volume/Å^3	1717.4(3)
Z	4
ρ_{calc}g/cm^3	1.601
μ/mm^{-1}	3.456
F(000)	840.0
Crystal size/mm^3	0.2300 × 0.1400 × 0.0500
Radiation	MoKα (λ = 0.71073)
2Θ range for data collection/°	7.06 to 52.04
Index ranges	$-9 \leq h \leq 10$, $-14 \leq k \leq 12$, $-20 \leq l \leq 21$
Reflections collected	6745
Independent reflections	3378 [R_{int} = 0.0475, R_{sigma} = 0.0849]
Data/restraints/parameters	3378/0/210
Goodness-of-fit on F^2	1.052
Final R indexes [I>=2σ (I)]	R_1 = 0.0781, wR_2 = 0.1586
Final R indexes [all data]	R_1 = 0.1312, wR_2 = 0.1843

Largest diff. peak/hole / e Å^{-3} 1.48/-1.27

Table 4 Bond Lengths for **49**.

**Atom**	**Atom**	**Length/Å**	**Atom**	**Atom**	**Length/Å**
Br1	Ni1	2.2855(13)	C6	C7	1.384(11)
Ni1	N1	1.928(6)	C7	C8	1.373(12)
Ni1	N2	1.973(6)	C8	C9	1.369(13)
Ni1	C11	1.895(9)	C9	C10	1.367(12)
N1	C1	1.344(10)	C11	C12	1.404(12)
N1	C5	1.343(9)	C11	C16	1.389(11)
N2	C6	1.358(9)	C12	C13	1.330(13)
N2	C10	1.348(10)	C13	C14	1.411(15)
C1	C2	1.377(12)	C14	C15	1.444(14)
C2	C3	1.363(13)	C15	C16	1.364(11)
C3	C4	1.375(12)	C16	C17	1.536(12)
C4	C5	1.380(11)	C17	C18	1.566(13)
C5	C6	1.459(11)	C17	C19	1.487(12)

Table 5 Bond Angles for **49**.

**Atom**	**Atom**	**Atom**	**Angle/°**	**Atom**	**Atom**	**Atom**	**Angle/°**
N1	Ni1	Br1	168.8(2)	N2	C6	C7	121.4(8)
N1	Ni1	N2	81.9(3)	C7	C6	C5	124.9(7)
N2	Ni1	Br1	97.07(19)	C8	C7	C6	119.6(8)
C11	Ni1	Br1	90.0(2)	C9	C8	C7	119.0(8)
C11	Ni1	N1	93.8(3)	C10	C9	C8	119.4(8)
C11	Ni1	N2	164.4(3)	N2	C10	C9	122.8(8)
C1	N1	Ni1	126.8(6)	C12	C11	Ni1	116.5(7)
C5	N1	Ni1	115.6(5)	C16	C11	Ni1	127.3(7)
C5	N1	C1	117.1(7)	C16	C11	C12	116.0(8)

C6	N2	Ni1	114.0(5)	C13	C12	C11	124.1(10)
C10	N2	Ni1	128.1(6)	C12	C13	C14	119.2(10)
C10	N2	C6	117.7(7)	C13	C14	C15	118.9(10)
N1	C1	C2	123.5(8)	C16	C15	C14	117.8(10)
C3	C2	C1	119.4(9)	C11	C16	C17	119.5(8)
C2	C3	C4	117.5(8)	C15	C16	C11	123.6(9)
C3	C4	C5	121.0(8)	C15	C16	C17	116.9(8)
N1	C5	C4	121.4(8)	C16	C17	C18	112.7(8)
N1	C5	C6	113.9(7)	C19	C17	C16	113.4(8)
C4	C5	C6	124.7(7)	C19	C17	C18	108.2(8)
N2	C6	C5	113.7(7)				

Table 6 Torsion Angles for **49**.

**A**	**B**	**C**	**D**	**Angle/°**	**A**	**B**	**C**	**D**	**Angle/°**
Br1	Ni1	N1	C1	94.5(12)	C2	C3	C4	C5	2.4(14)
Br1	Ni1	N1	C5	-77.2(12)	C3	C4	C5	N1	-0.4(13)
Br1	Ni1	N2	C6	166.0(5)	C3	C4	C5	C6	-179.0(8)
Br1	Ni1	N2	C10	-19.0(7)	C4	C5	C6	N2	-172.2(7)
Br1	Ni1	C11	C12	98.6(6)	C4	C5	C6	C7	8.5(12)
Br1	Ni1	C11	C16	-87.5(7)	C5	N1	C1	C2	4.1(14)
Ni1	N1	C1	C2	-167.5(8)	C5	C6	C7	C8	176.8(8)
Ni1	N1	C5	C4	169.7(6)	C6	N2	C10	C9	0.4(12)
Ni1	N1	C5	C6	-11.6(8)	C6	C7	C8	C9	2.0(13)
Ni1	N2	C6	C5	-2.6(8)	C7	C8	C9	C10	-0.4(14)
Ni1	N2	C6	C7	176.7(6)	C8	C9	C10	N2	-0.8(14)
Ni1	N2	C10	C9	-174.4(6)	C10	N2	C6	C5	-178.1(7)
Ni1	C11	C12	C13	169.3(8)	C10	N2	C6	C7	1.2(11)
Ni1	C11	C16	C15	-171.0(6)	C11	Ni1	N1	C1	-15.3(8)

Ni1	C11	C16	C17	10.5(11)	C11	Ni1	N1	C5	173.0(6)
N1	Ni1	N2	C6	-2.7(5)	C11	Ni1	N2	C6	-77.3(12)
N1	Ni1	N2	C10	172.2(7)	C11	Ni1	N2	C10	97.6(13)
N1	Ni1	C11	C12	-91.9(7)	C11	C12	C13	C14	1.8(16)
N1	Ni1	C11	C16	82.0(7)	C11	C16	C17	C18	-122.9(9)
N1	C1	C2	C3	-2.1(16)	C11	C16	C17	C19	113.7(9)
N1	C5	C6	N2	9.1(9)	C12	C11	C16	C15	2.9(12)
N1	C5	C6	C7	-170.1(7)	C12	C11	C16	C17	-175.5(7)
N2	Ni1	N1	C1	179.7(8)	C12	C13	C14	C15	4.1(15)
N2	Ni1	N1	C5	8.1(6)	C13	C14	C15	C16	-6.2(13)
N2	Ni1	C11	C12	-18.8(15)	C14	C15	C16	C11	2.6(13)
N2	Ni1	C11	C16	155.1(9)	C14	C15	C16	C17	-178.9(8)
N2	C6	C7	C8	-2.4(12)	C15	C16	C17	C18	58.5(10)
C1	N1	C5	C4	-2.8(12)	C15	C16	C17	C19	-64.9(11)
C1	N1	C5	C6	175.9(7)	C16	C11	C12	C13	-5.3(13)
C1	C2	C3	C4	-1.2(15)					

9.2 Synthesis of complex 50

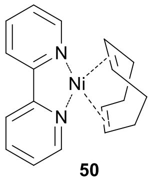

50

This compound was synthesized according to a modified literature procedure.[22] The reaction worked in an argon-filled glove box. To a flame-dried round-bottomed flask was added bpy (312 mg, 2.0 mmol), Ni(cod)$_2$ (550 mg, 2.0 mmol) and THF (50 mL). After stirring at room temperature overnight, the reaction mixture was removed from glove box. Much of THF was removed under reduced pressure and deoxygenated ethyl ether (10 mL) was added. A solid was precipitated when cooled with liquid nitrogen. The collected solid was washed with degassed cold Et2O (3 x 5 mL) and dried for 1 h under vacuum to give a black, shiny solid (270mg, 41% yield). **^{1}H NMR** and **^{13}C NMR** data are compatible with those reported in ref. 23. **^{1}H NMR (400 MHz, C_6D_6):** δ 10.15 (d, *J* = 5.6

Hz, 2H), 7.29-7.28 (m, 4H), 7.03-6.99 (m, 2H), 3.92 (s, 4H), 2.84-2.82 (m, 4H), 1.98-1.92 (m, 4H). **^{13}C NMR (100 MHz, C_6D_6):** δ 150.6, 145.4, 124.9, 122.3, 122.2, 82.1, 31.9.

9.3 The procedure for experiments in Figure 1.

These two reaction were conducted an argon-filled glove box at the same time.

The reaction of 1ae with 2a catalyzed by 30% of Ar-Ni^{II}(bpy)Br (49). To a mixture of 1-bromo-2-isopropylbenzene (**1ae**, 48 mg, 0.24 mmol), (*E*)-3-phenylprop-2-en-1-ol (**2a**, 27 mg, 0.2 mmol), $ZrCl_4$ (4.7 mg, 0.02 mmol) and bpy (9.3 mg, 0.06 mmol) in DMA (1 mL) was added a solution of Ar-Ni^{II}(bpy)Br (**49**, 25 mg, 0.06 mmol) in DMA (1 mL) and Mn (33 mg, 3.0 *eq.*). The reaction mixture was stirred at room temperature. A 100 μL of the reaction mixture was collected with a pipette each time. The collected mixture was quenched with H_2O (200 μL), diluted with diethyl ether (1 mL) and filtered through a syringe filter. The filtrate was analyzed by GC and the yield was calculated versus the internal standard (dodecane).

The reaction of 1ae with 2a catalyzed by 30% of (bpy)Ni^0(cod) (50). To a mixture of 1-bromo-2-isopropylbenzene (**1ae**, 60 mg, 0.3 mmol), (*E*)-3-phenylprop-2-en-1-ol (**2a**, 27 mg, 0.2 mmol), $ZrCl_4$ (4.7 mg, 0.02 mmol) and bpy (9.3 mg, 0.06 mmol) in DMA (1 mL) was added a solution of (bpy)Ni^0(cod) (**50**, 19.4 mg, 0.06 mmol) in DMA (1 mL) and Mn (33 mg, 3.0 *eq.*). The reaction mixture was stirred at room temperature. A 100 μL of the reaction mixture was collected with a pipette each time. The collected mixture was quenched with H_2O (200 μL), diluted with diethyl ether (1 mL) and filtered through a syringe filter. The filtrate was analyzed by GC and the yield was calculated versus the internal standard (dodecane).

9.4 Stoichiometric reaction of complex 49 with 2a

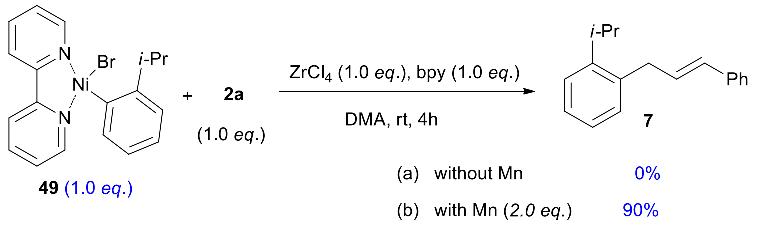

To a tube charged with **49** (82.6 mg, 0.2 mmol), bpy (31.0 mg, 0.2 mmol), $ZrCl_4$ (47 mg, 0.2 mmol) and Mn (32.9 mg, 0.6 mmol) was added a solution of Cinnamyl alcohol **2a** (26.8 mg, 0.2 mmol) in DMA (2 mL, 0.1 M) at -78 °C. The reaction tube was vacuumed and refilled with argon for

3 times. After stirring at room temperature for 4 h, the reaction mixture was quenched with H_2O (30 mL) and extracted with EtOAc (3 × 30 mL). The organic mixture was analyzed by GC-MS and GC to show 90% yield of **7** was obtained in the presence of Mn, but no desired product was observed without Mn.

10. Reference

1. W. L. F. Armarego, C. L. L. Chai, *Purification of laboratory chemicals*, 5th ed., Butterworth-Heinemann, **2003**.
2. M.V. Vita, P. Caramenti, J. Waser, *Org. Lett.* **2015**, *17*, 5832.
3. S. J. Chen, G. P. Lu, C. Cai, *Chem. Commun.* **2015**, *51*, 11512.
4. R. Tomita, K. Mantani, A. Hamasaki, T. Ishida, M. Tokunaga, *Chem. Eur. J.* **2014**, *20*, 9914.
5. C. B. Kelly, J. M. Ovian, R. M. Cywar, T. R. Gosselin, R. J. Wiles, N. E. Leadbeater, *Org. Biomol. Chem.* **2015**, *13*, 4255.
6. J. W. Kim, T. Koike, M. Kotani, K. Yamaguchi, N. Mizuno, *Chem. Eur. J.* **2008**, *14*, 4104.
7. S. Akai, R. Hanada, N. Fujiwara, Y. Kita, M. Egi, *Org. Lett.* **2010**, *12* 4900.
8. S. M. Sarkar, Y. Uozumi, Y. Yamada, *Angew. Chem. Int. Ed.* **2011**, *50*, 9437.
9. H. Tsukamoto, T. Uchiyama, T. Suzuki, Y. Kondo, *Org. Biomol. Chem.* **2008**, *6*, 3005.
10. J. L. Tao, B. Yang, Z. X. Wang, *J. Org. Chem.*, **2015**, *80*, 12627.
11. L. L. Anka-Lufford, M. R. Prinsell, D. J. Weix , *J. Org. Chem.* **2012**, *77*, 9989.
12. K. Motokura, N. Nakagiri, T. Mizugaki, K. Ebitani, K. Kaneda, *J. Org. Chem.* **2007**, *72*, 6006.
13. S. L. Sharada, T. Edmond, *J. Org. Chem.* **1994**, *59*, 4250.
14. D. W. Robbins, T. A. Boebel, J. F. Hartwig, *J. Am. Chem. Soc*. **2010**, *132*, 4068.
15. Y. Yamamoto, S. Takada, N. Miyaura, *Organometallics* **2009**, *28*, 152.
16. X. Z. Cui, S. Wang, Y. W. Zhang, Q. Qian, H. G. Gong, *Org. Biomol. Chem.* **2013**, *11*, 3094.
17. Y. Nishimoto, M. Kajioka, T. Saito, M. Yasudaa, A. Baba, *Chem. Commun.* **2008**, 6396.
18. C. H. Schuster, J. R. Coombs, Z. A. Kasun, J. P. Morken, *Org. Lett.* **2014**, *16*, 4420.
19. M. Vellakkaran, M. Andappanb, N. Kommu, *Green Chem.* **2014**, *16*, 2788.
20. Y. Wang, S. Zhang, L. Wang, W. Zhang, N. Zhou, Z. Zhang, X. Zhu, *Polym. Chem.* **2015**, *6*, 4669.
21. S. Biswas, D. J. Weix, *J. Am. Chem. Soc*. **2013**, *135*, 16192.
22. J. J. Eisch, A. M. Piotrowski, K. I. Han, C. Kruger, Y. H. Tsay *Organometallics*, **1985**, *4*, 224.
23. P. Binger, M. J. Doyle, J. Mcmeeking, C. Kruger, Y.-H. Tsay. *J. Organometal. Chem.*, **1977**, *135*, 405.

2d

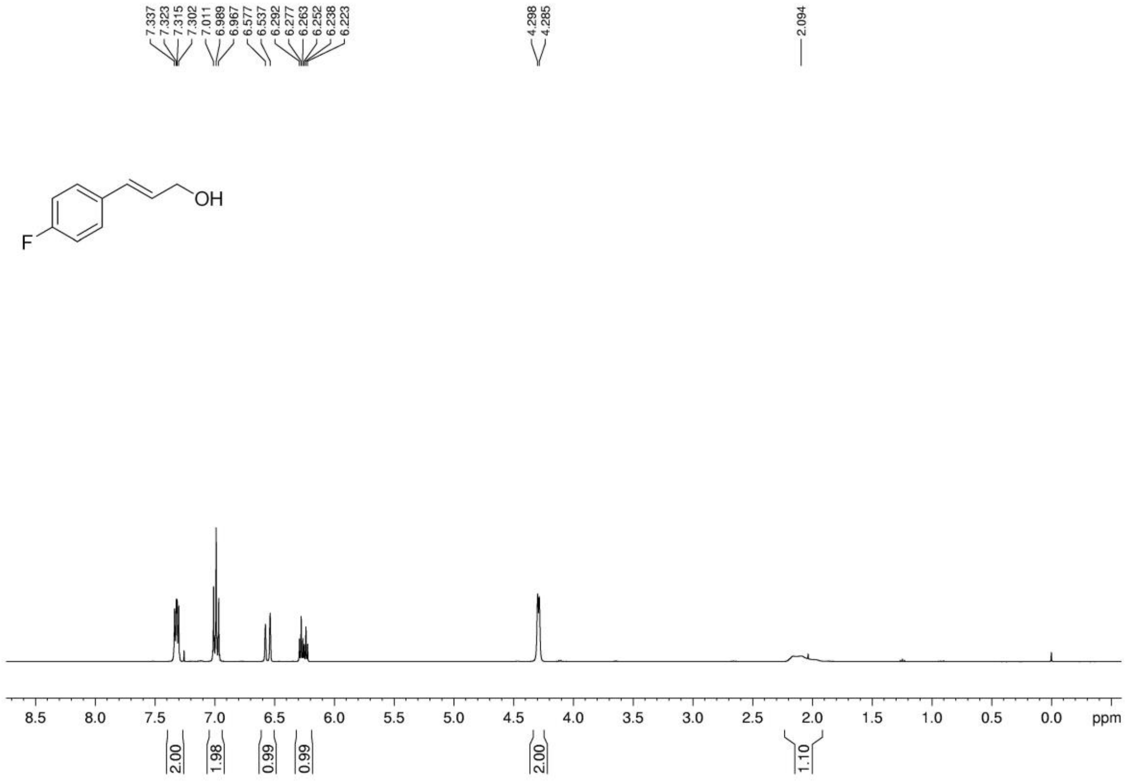
7.337
7.323
7.315
7.302
7.011
6.989
6.967
6.577
6.537
6.292
6.277
6.263
6.252
6.238
6.223
4.298
4.285
2.094
OH
F
2.00
1.98
0.99
0.99
2.00
1.10
ppm

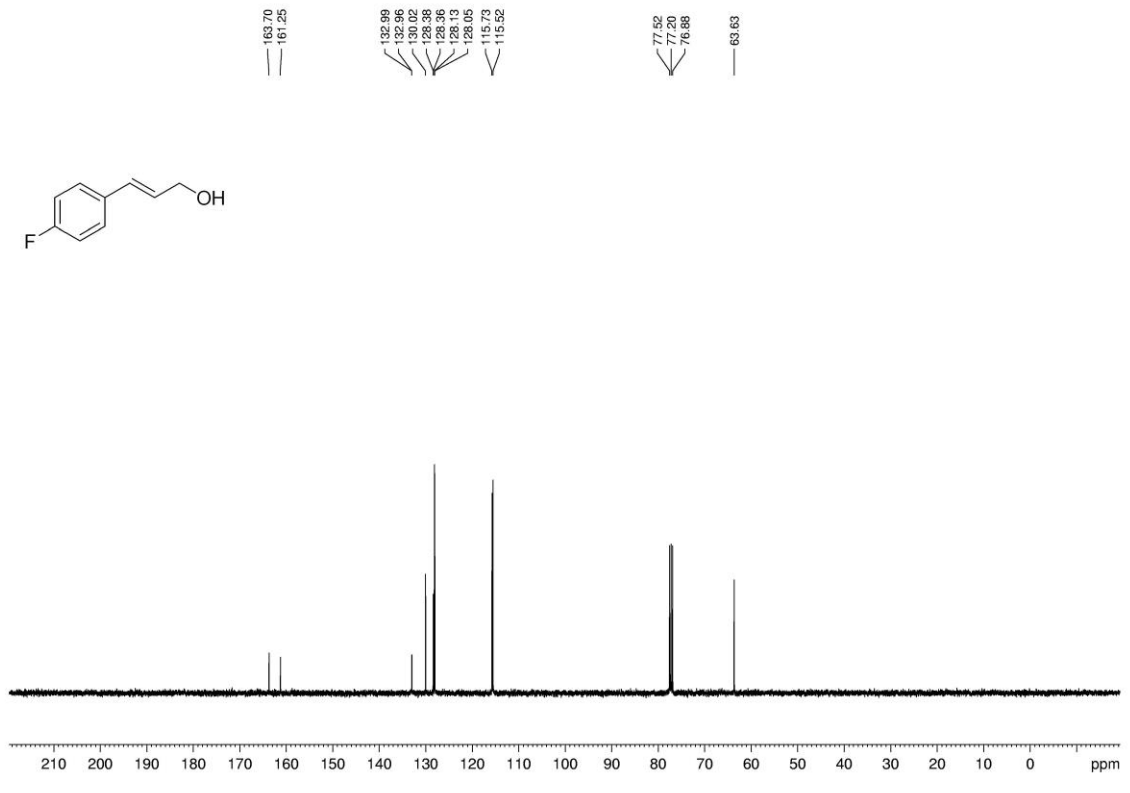
163.70
161.25
132.99
132.96
130.02
128.38
128.36
128.13
128.05
115.73
115.52
77.52
77.20
76.88
63.63
OH
F
ppm

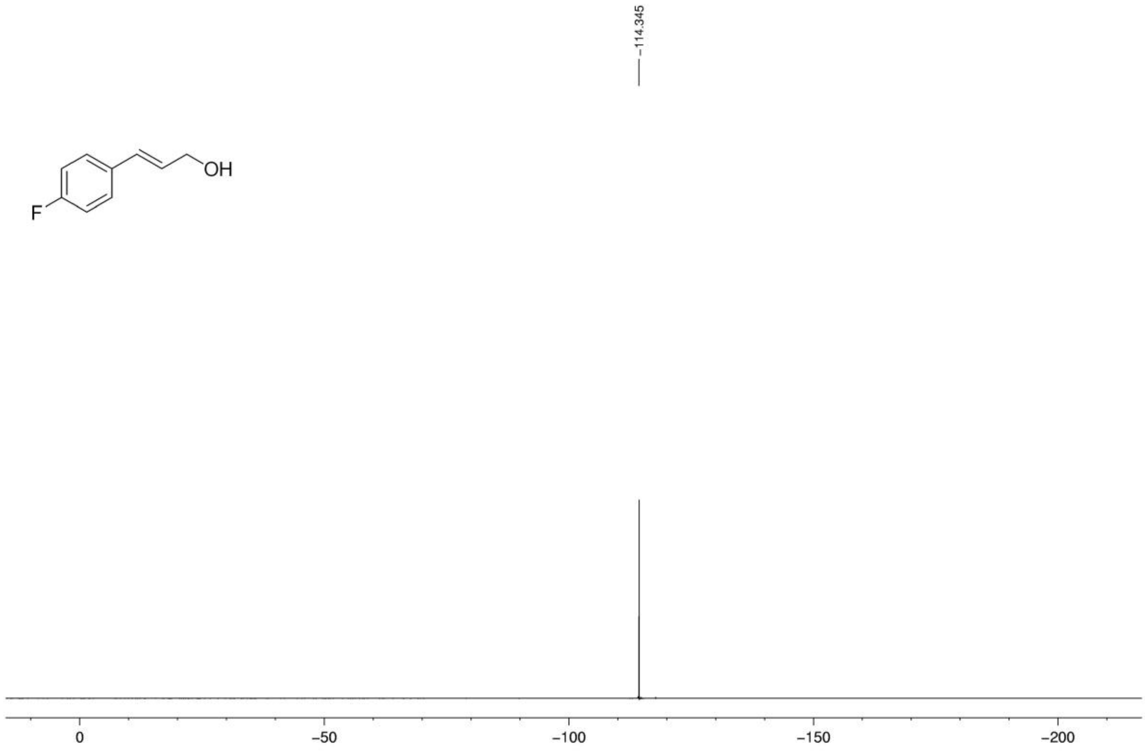

F
OH
-114.345
0
-50
-100
-150
-200

2e

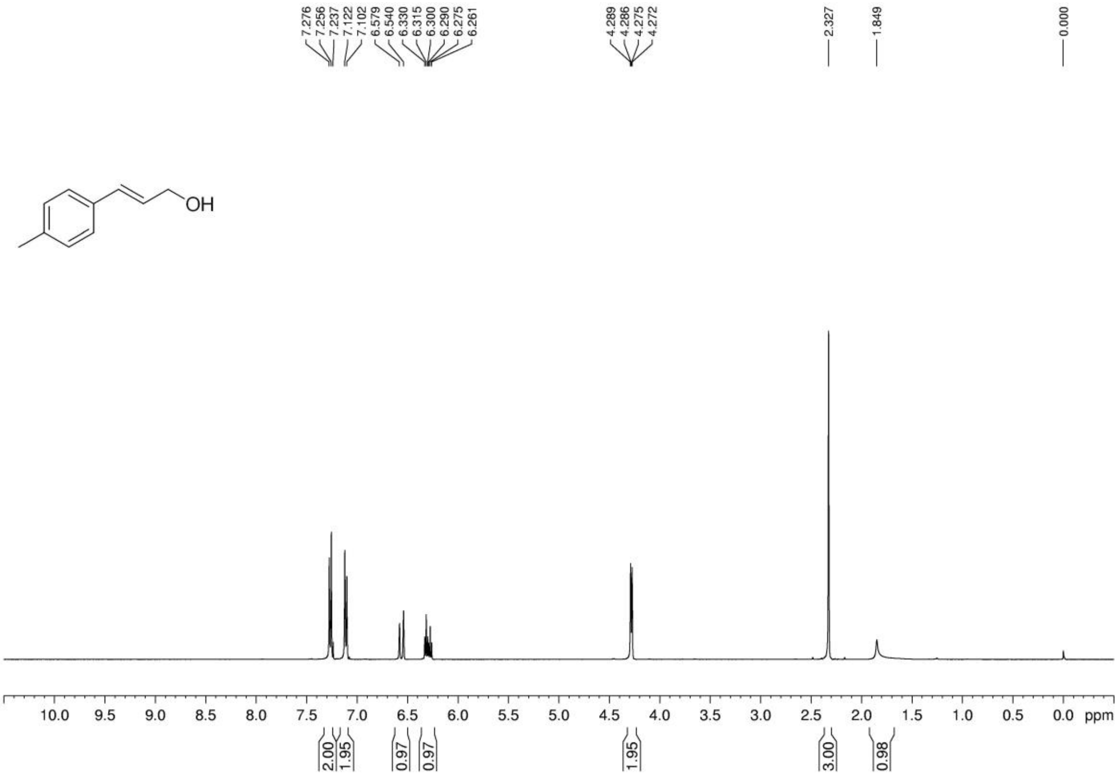

7.276
7.256
7.237
7.122
7.102
6.579
6.540
6.330
6.315
6.300
6.290
6.275
6.261
4.289
4.286
4.275
4.272
2.327
1.849
0.000
OH
10.0 9.5 9.0 8.5 8.0 7.5 7.0 6.5 6.0 5.5 5.0 4.5 4.0 3.5 3.0 2.5 2.0 1.5 1.0 0.5 0.0 ppm
2.00
1.95
0.97
0.97
1.95
3.00
0.98

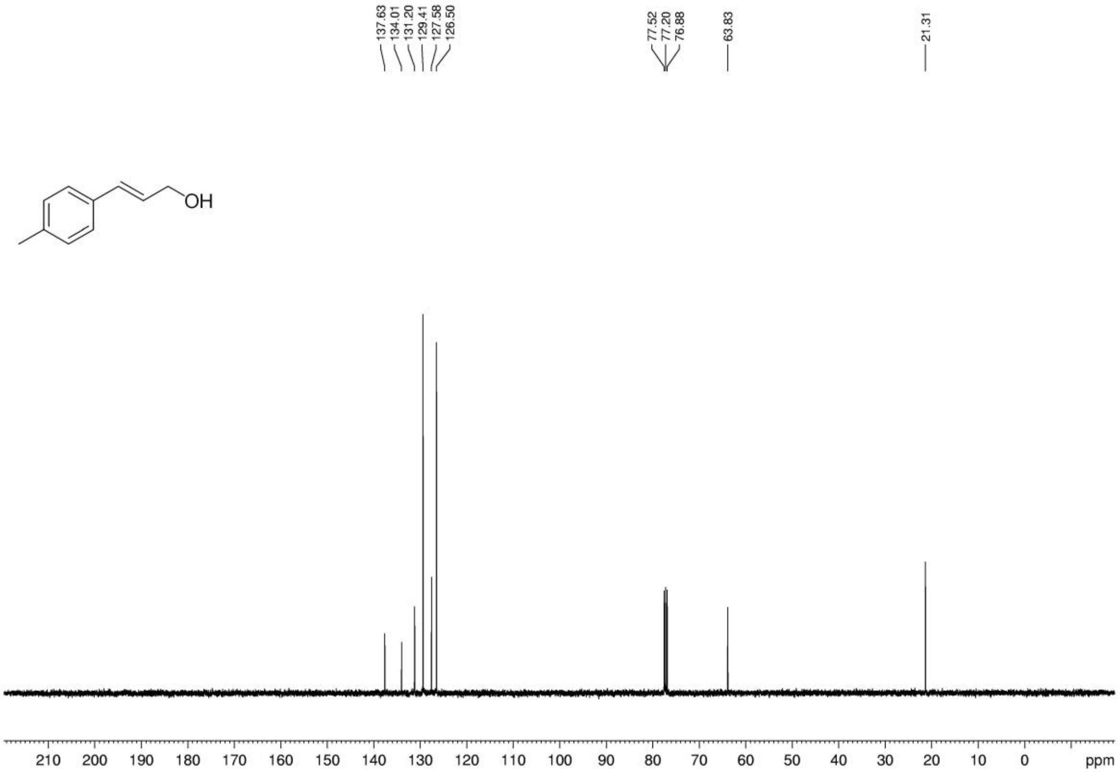

137.63
134.01
131.20
129.41
127.58
126.50
77.52
77.20
76.88
63.83
21.31
OH
210 200 190 180 170 160 150 140 130 120 110 100 90 80 70 60 50 40 30 20 10 0 ppm

2f

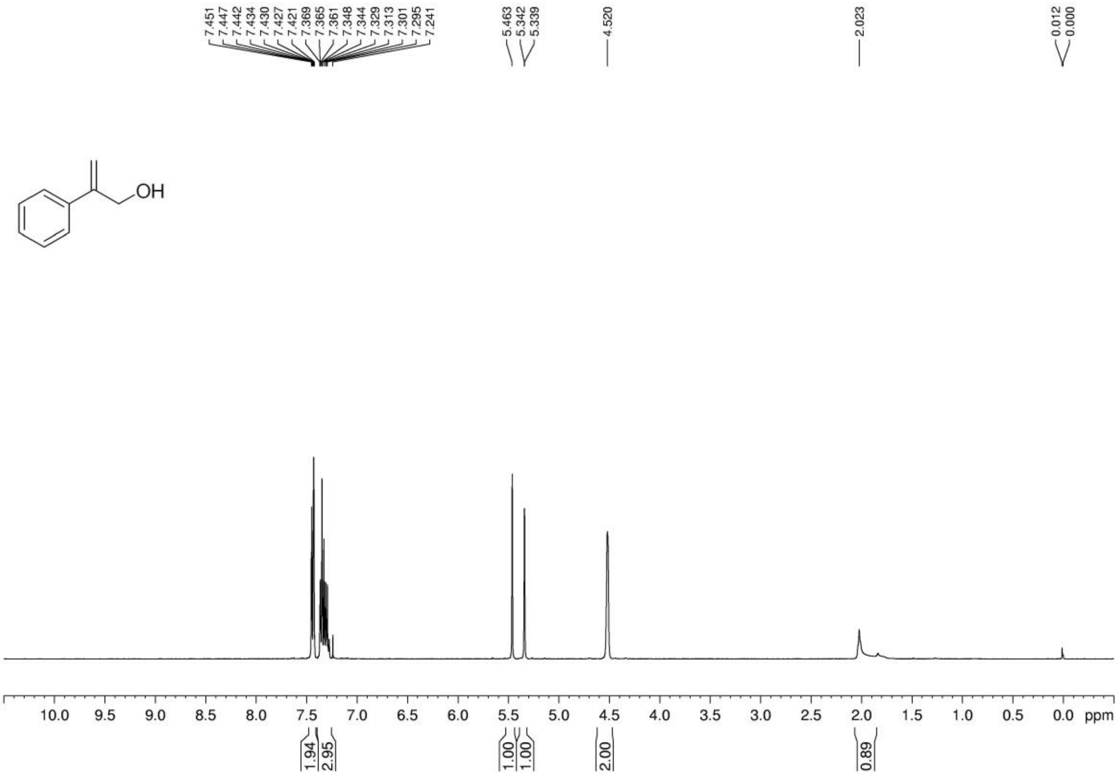
7.451
7.447
7.442
7.434
7.430
7.427
7.421
7.369
7.365
7.361
7.348
7.344
7.329
7.313
7.301
7.295
7.241
5.463
5.342
5.339
4.520
2.023
0.012
0.000
OH
ppm
1.94
2.95
1.00
1.00
2.00
0.89

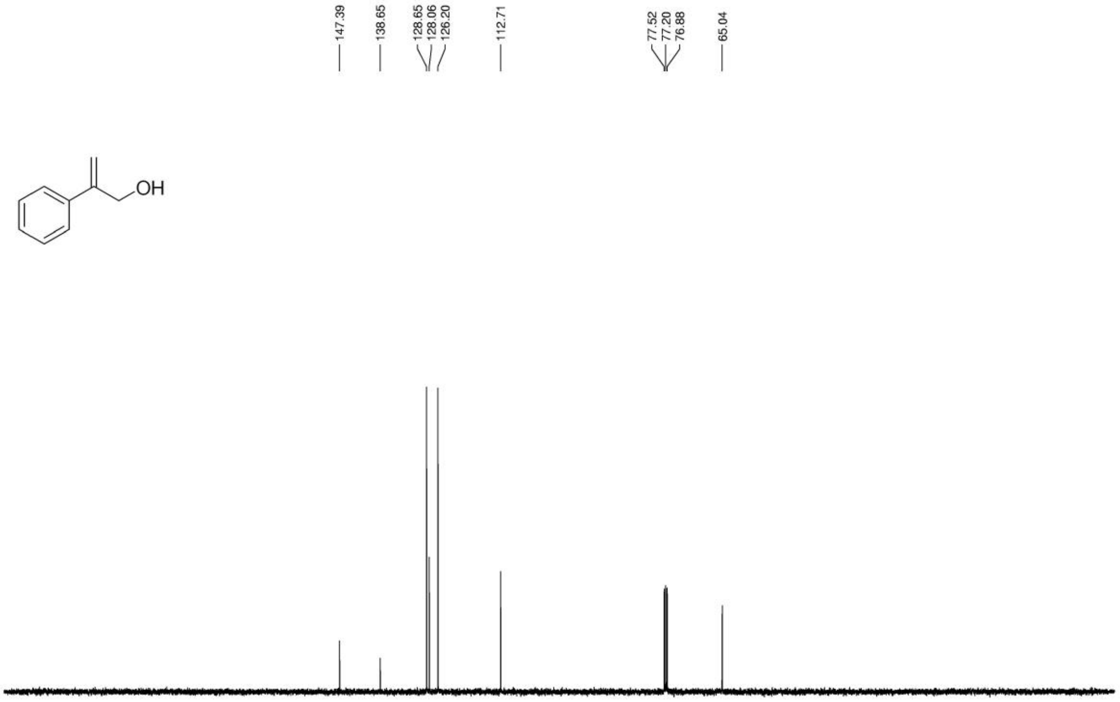
147.39
138.65
128.65
128.06
126.20
112.71
77.52
77.20
76.88
65.04
OH
ppm

2h

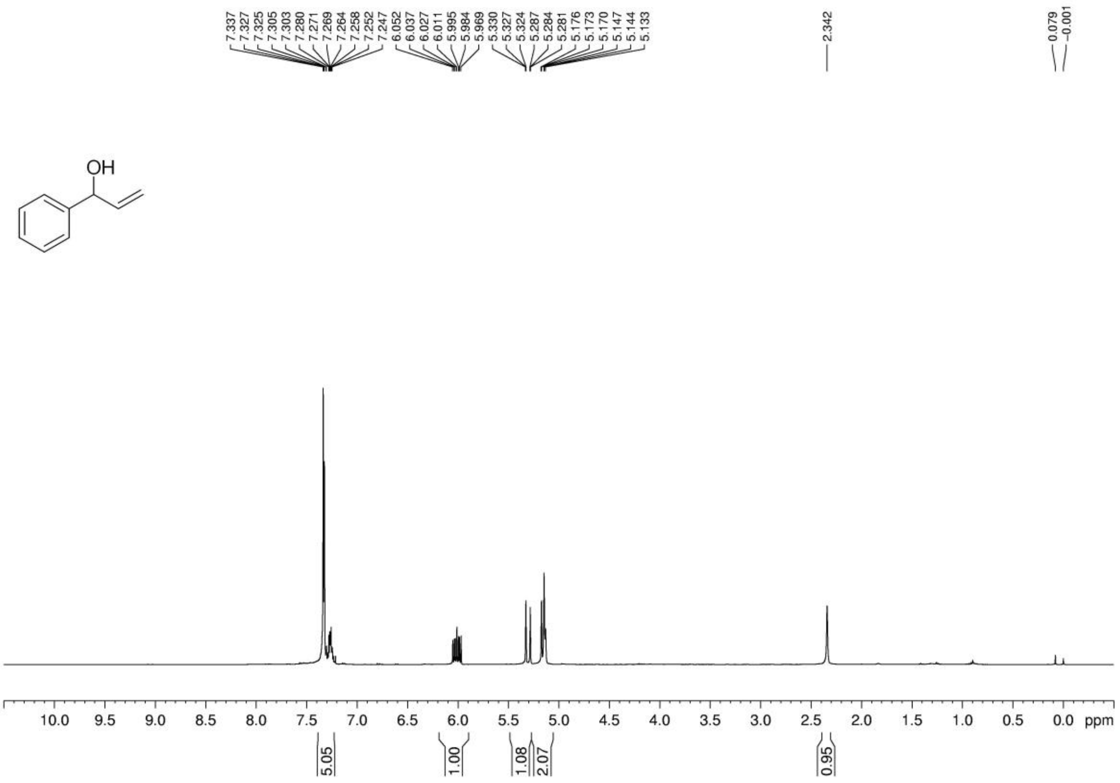
7.337
7.327
7.325
7.305
7.303
7.280
7.271
7.269
7.264
7.258
7.252
7.247
6.052
6.037
6.027
6.011
5.995
5.984
5.969
5.330
5.327
5.324
5.287
5.284
5.281
5.176
5.173
5.170
5.147
5.144
5.133
2.342
0.079
-0.001
OH
10.0
9.5
9.0
8.5
8.0
7.5
7.0
6.5
6.0
5.5
5.0
4.5
4.0
3.5
3.0
2.5
2.0
1.5
1.0
0.5
0.0
ppm
5.05
1.00
1.08
2.07
0.95

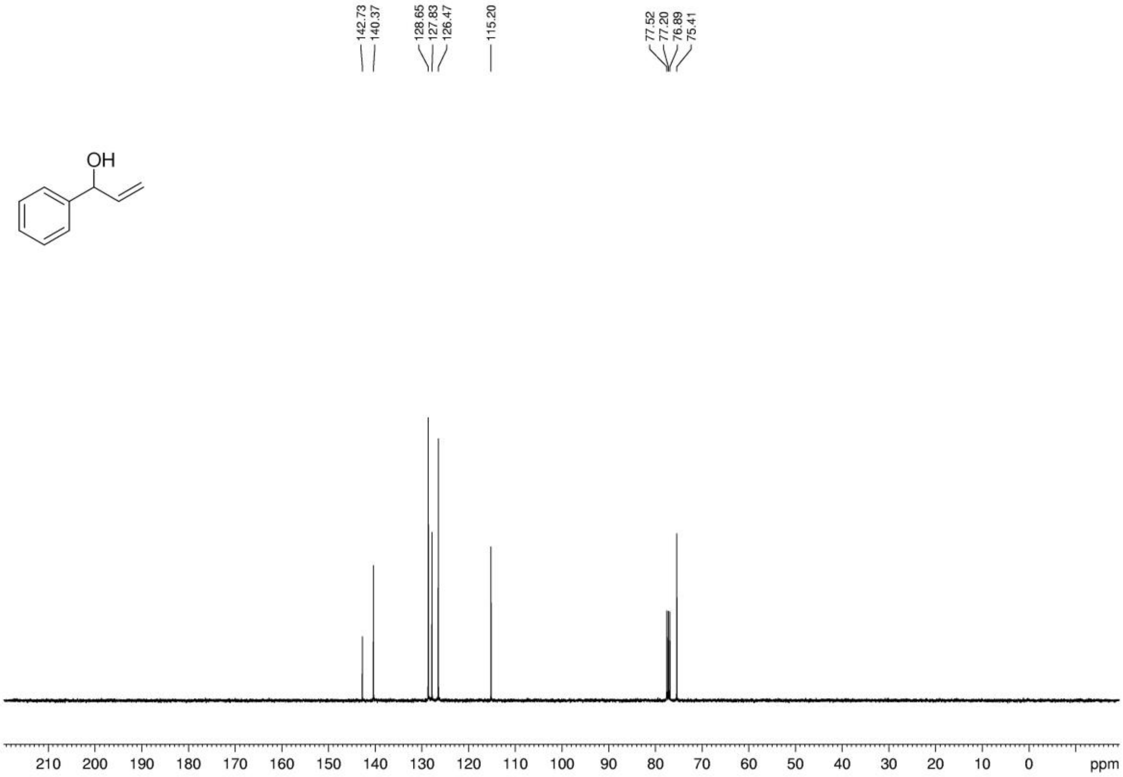
142.73
140.37
128.65
127.83
126.47
115.20
77.52
77.20
76.89
75.41
OH
210
200
190
180
170
160
150
140
130
120
110
100
90
80
70
60
50
40
30
20
10
0
ppm

2j

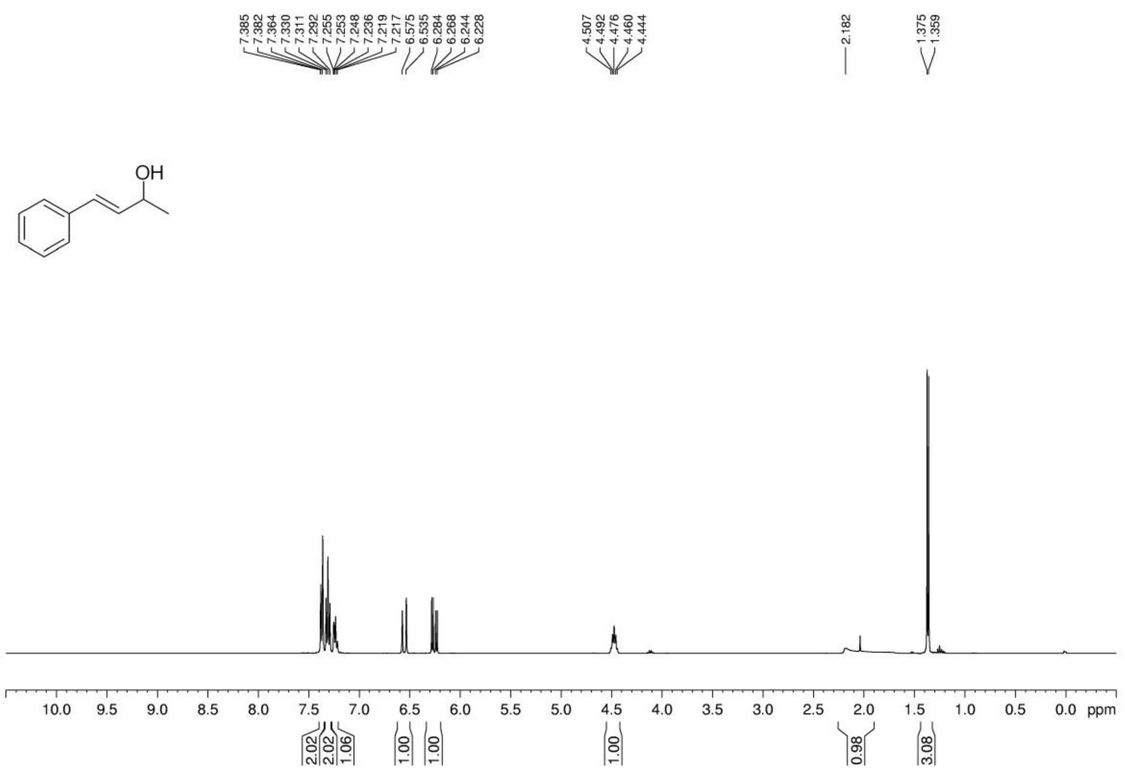
7.385 7.382 7.364 7.330 7.311 7.292 7.255 7.253 7.248 7.236 7.219 7.217 6.575 6.535 6.284 6.268 6.244 6.228
4.507 4.492 4.476 4.460 4.444
2.182
1.375 1.359
OH
10.0 9.5 9.0 8.5 8.0 7.5 7.0 6.5 6.0 5.5 5.0 4.5 4.0 3.5 3.0 2.5 2.0 1.5 1.0 0.5 0.0 ppm
2.02 2.02 1.06 1.00 1.00 1.00 0.98 3.08

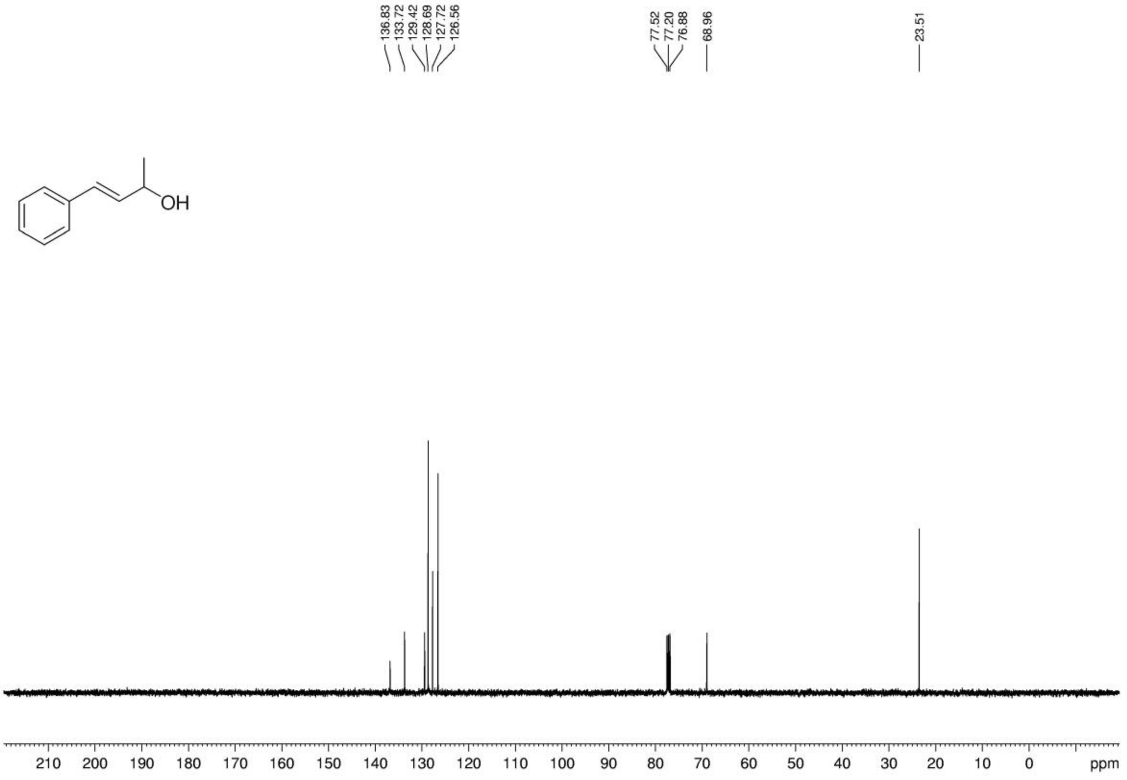
136.83 133.72 129.42 128.69 127.72 126.56
77.52 77.20 76.88 68.96
23.51
OH
210 200 190 180 170 160 150 140 130 120 110 100 90 80 70 60 50 40 30 20 10 0 ppm

2k

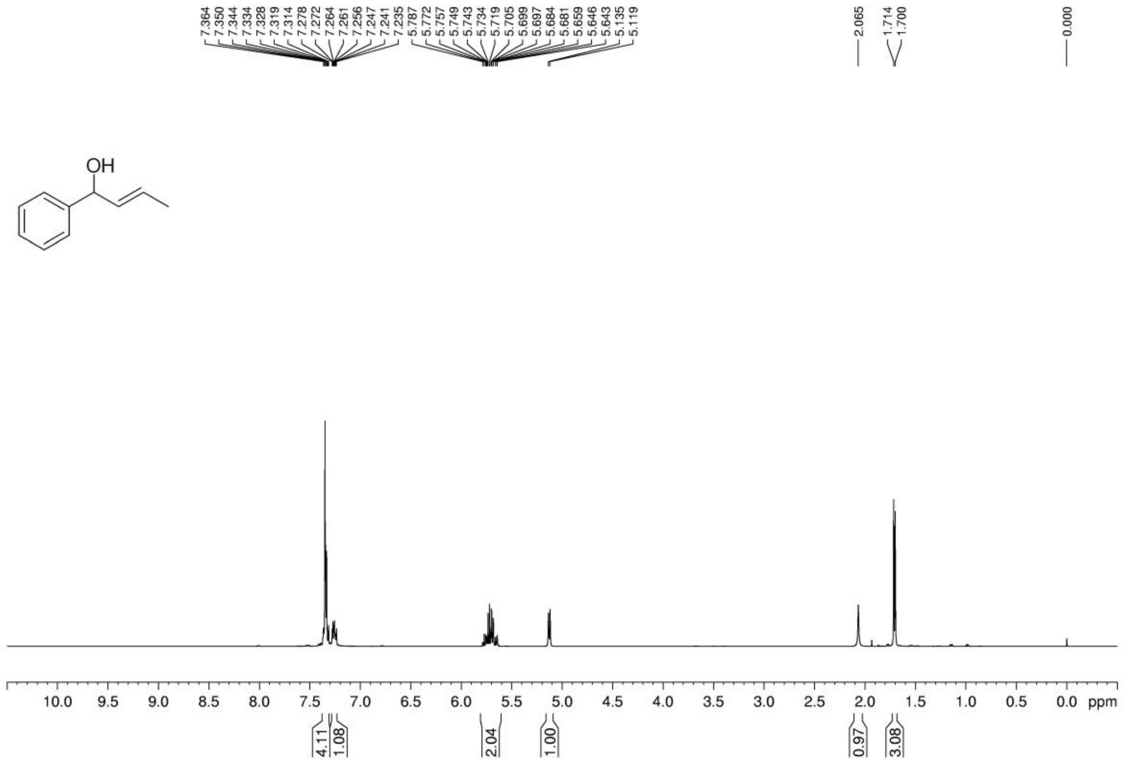
7.364 7.350 7.344 7.334 7.328 7.319 7.314 7.278 7.272 7.264 7.261 7.256 7.247 7.241 7.235 5.787 5.772 5.757 5.749 5.743 5.734 5.719 5.705 5.699 5.697 5.684 5.681 5.659 5.646 5.643 5.135 5.119
2.065
1.714 1.700
0.000
OH
10.0 9.5 9.0 8.5 8.0 7.5 7.0 6.5 6.0 5.5 5.0 4.5 4.0 3.5 3.0 2.5 2.0 1.5 1.0 0.5 0.0 ppm
4.11 1.08 2.04 1.00 0.97 3.08

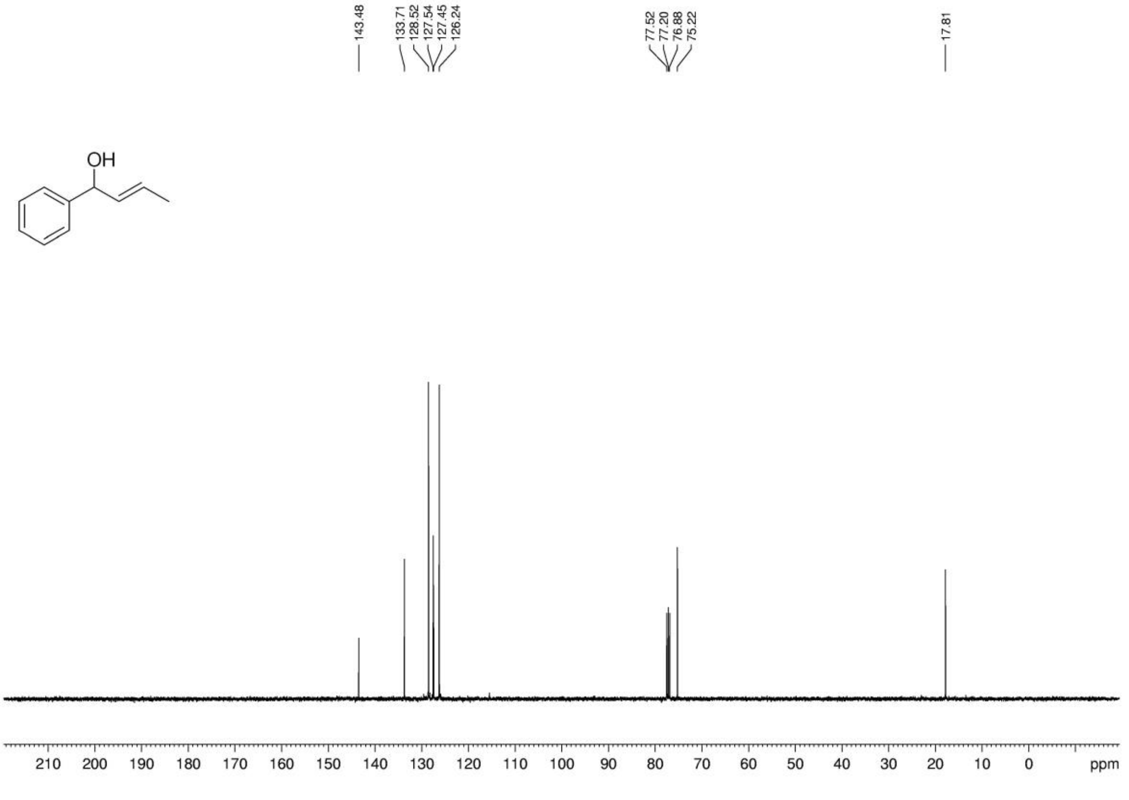
143.48
133.71 128.52 127.54 127.45 126.24
77.52 77.20 76.88 75.22
17.81
OH
210 200 190 180 170 160 150 140 130 120 110 100 90 80 70 60 50 40 30 20 10 0 ppm

2l

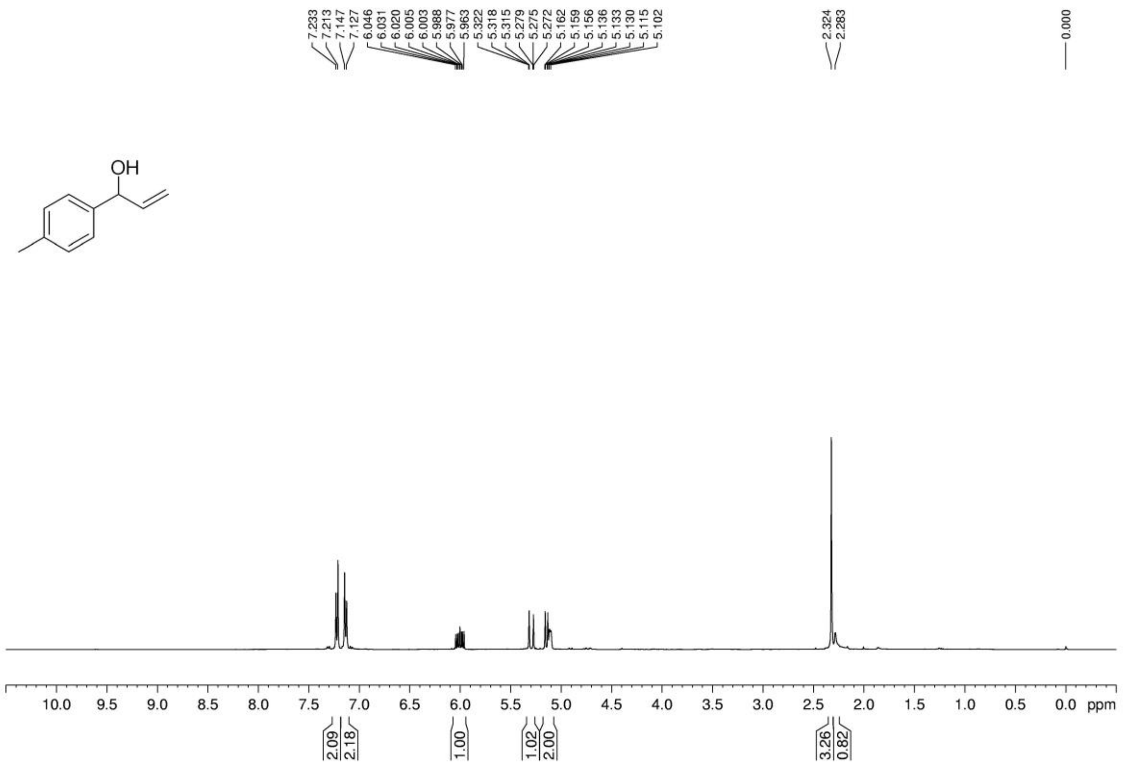
7.233
7.213
7.147
7.127
6.046
6.031
6.020
6.005
6.003
5.988
5.977
5.963
5.322
5.318
5.315
5.279
5.275
5.272
5.162
5.159
5.156
5.136
5.133
5.130
5.115
5.102
2.324
2.283
0.000
OH
10.0 9.5 9.0 8.5 8.0 7.5 7.0 6.5 6.0 5.5 5.0 4.5 4.0 3.5 3.0 2.5 2.0 1.5 1.0 0.5 0.0 ppm
2.09
2.18
1.00
1.02
2.00
3.26
0.82

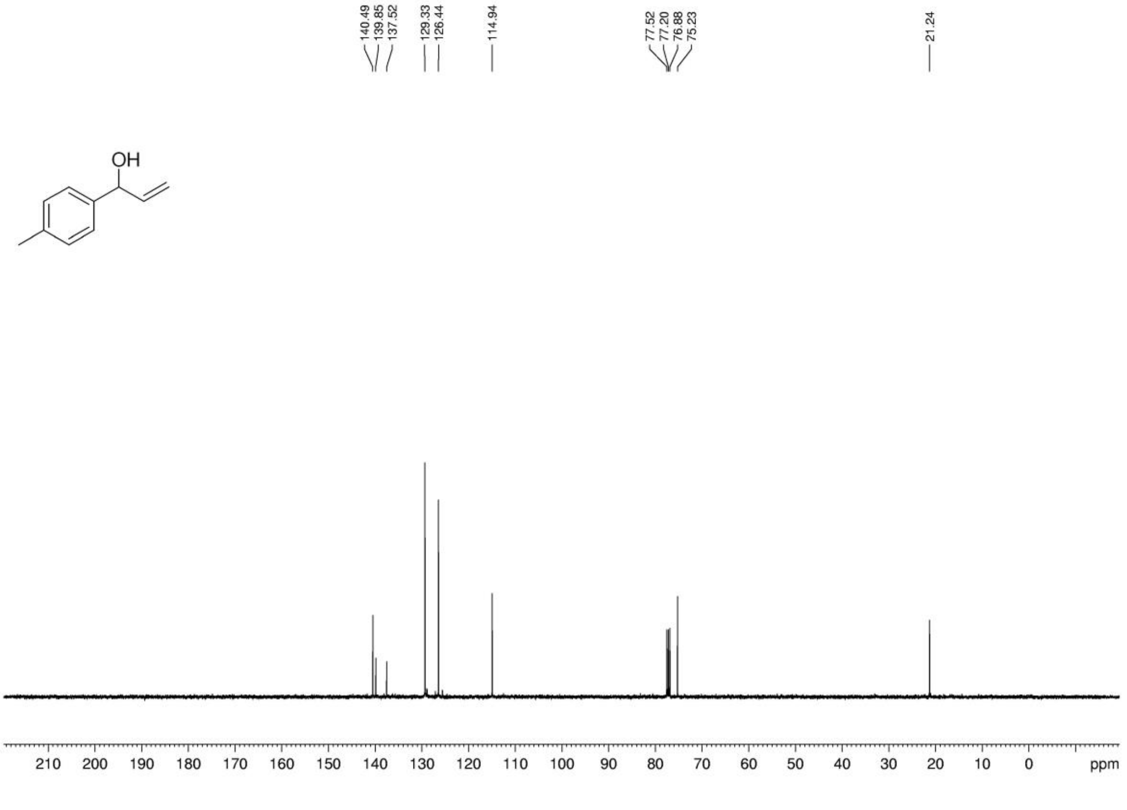
140.49
139.85
137.52
129.33
126.44
114.94
77.52
77.20
76.88
75.23
21.24
OH
210 200 190 180 170 160 150 140 130 120 110 100 90 80 70 60 50 40 30 20 10 0 ppm

3

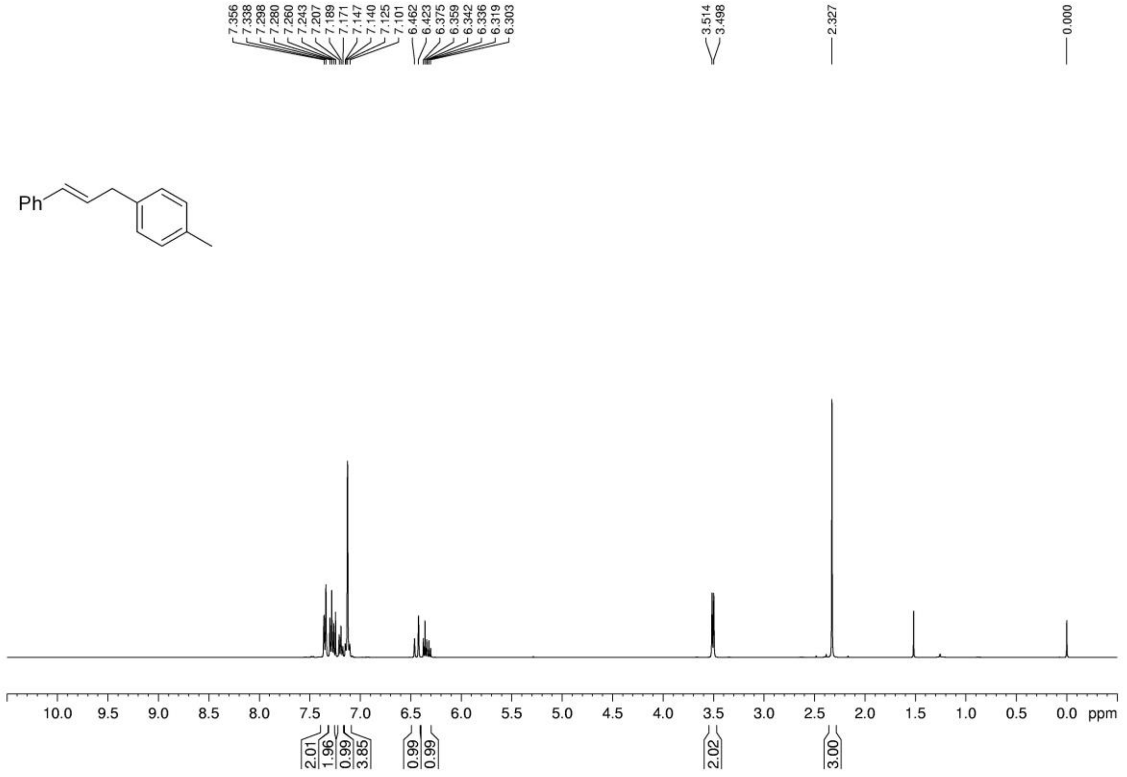
7.356
7.338
7.298
7.280
7.260
7.243
7.207
7.189
7.171
7.147
7.140
7.125
7.101
6.462
6.423
6.375
6.359
6.342
6.336
6.319
6.303
3.514
3.498
2.327
0.000
Ph
10.0 9.5 9.0 8.5 8.0 7.5 7.0 6.5 6.0 5.5 5.0 4.5 4.0 3.5 3.0 2.5 2.0 1.5 1.0 0.5 0.0 ppm
2.01
1.96
0.99
3.85
0.99
0.99
2.02
3.00

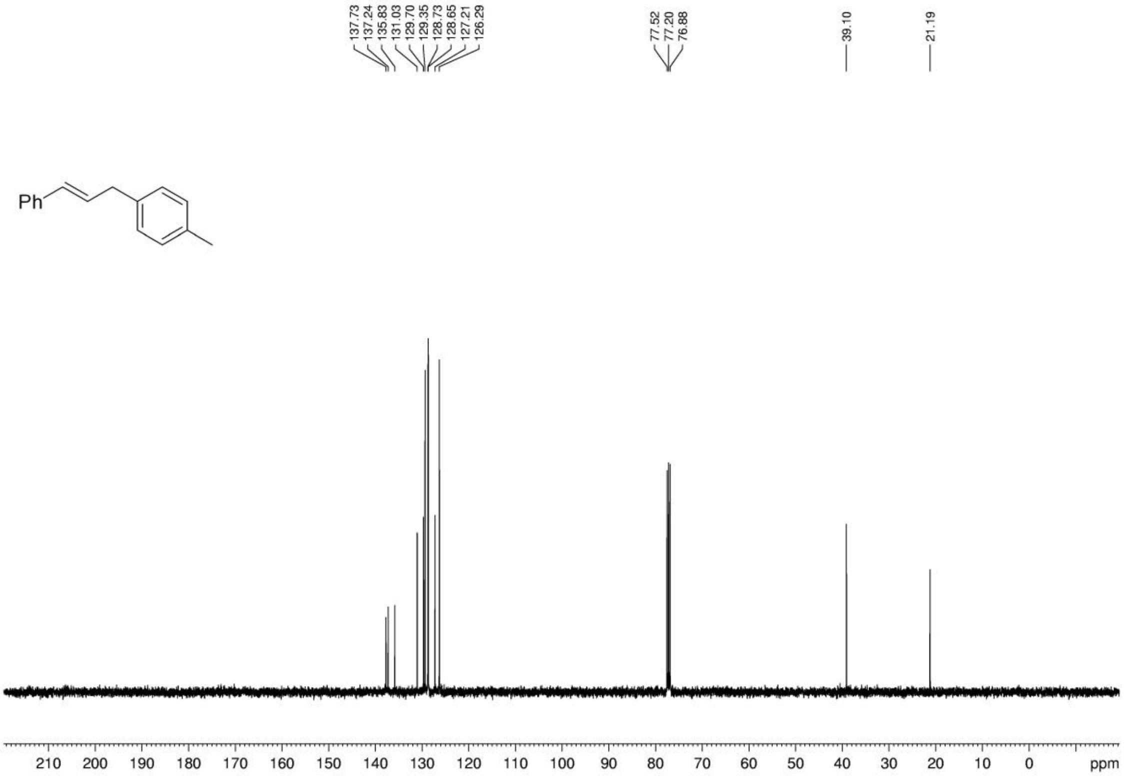
137.73
137.24
135.83
131.03
129.70
129.35
128.73
128.65
127.21
126.29
77.52
77.20
76.88
39.10
21.19
Ph
210 200 190 180 170 160 150 140 130 120 110 100 90 80 70 60 50 40 30 20 10 0 ppm

4

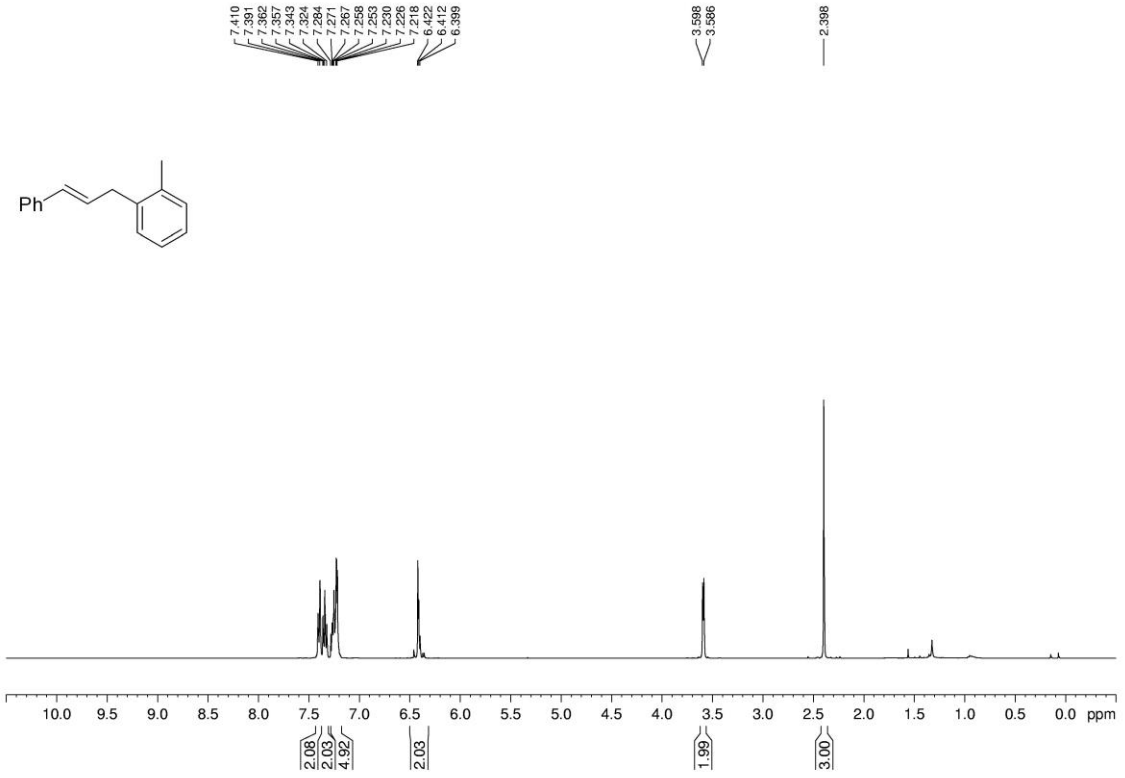

7.410
7.391
7.362
7.357
7.343
7.324
7.284
7.271
7.267
7.258
7.253
7.230
7.226
7.218
6.422
6.412
6.399
3.598
3.586
2.398
Ph
10.0 9.5 9.0 8.5 8.0 7.5 7.0 6.5 6.0 5.5 5.0 4.5 4.0 3.5 3.0 2.5 2.0 1.5 1.0 0.5 0.0 ppm
2.08
2.03
4.92
2.03
1.99
3.00

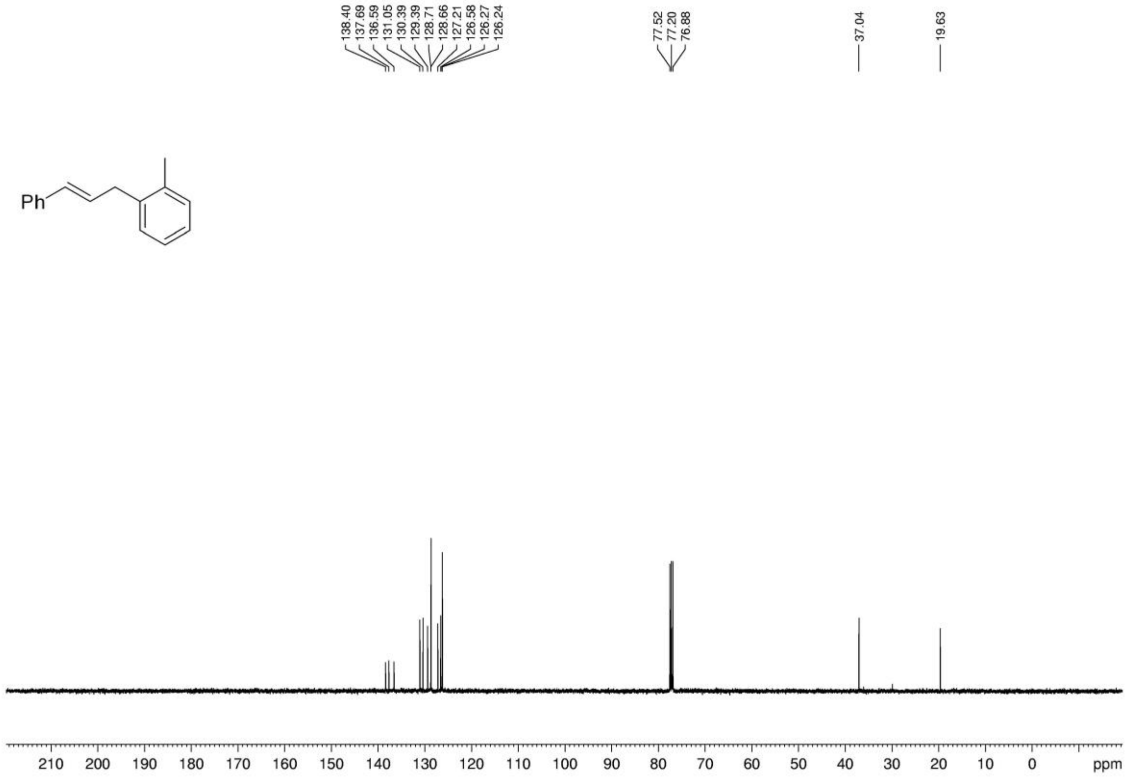

138.40
137.69
136.59
131.05
130.39
129.39
128.71
128.66
127.21
126.58
126.27
126.24
77.52
77.20
76.88
37.04
19.63
Ph
210 200 190 180 170 160 150 140 130 120 110 100 90 80 70 60 50 40 30 20 10 0 ppm

5

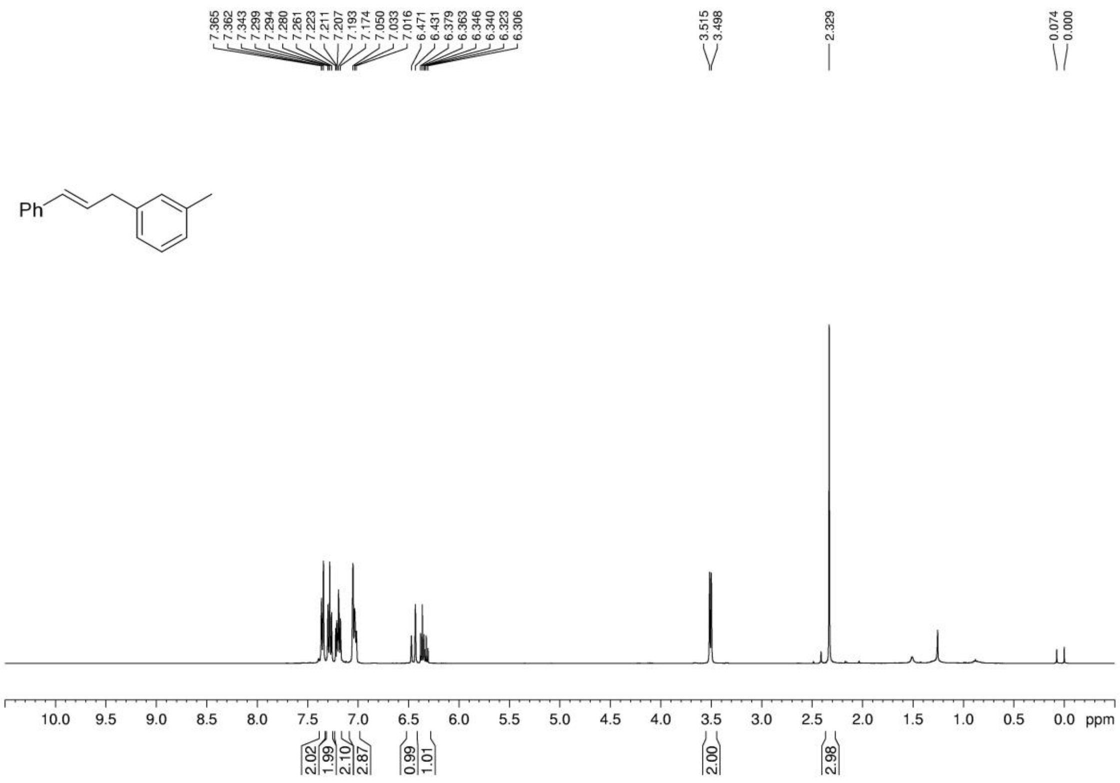

7.365
7.362
7.343
7.299
7.294
7.280
7.261
7.223
7.211
7.207
7.193
7.174
7.050
7.033
7.016
6.471
6.431
6.379
6.363
6.346
6.340
6.323
6.306
3.515
3.498
2.329
0.074
0.000
Ph
10.0 9.5 9.0 8.5 8.0 7.5 7.0 6.5 6.0 5.5 5.0 4.5 4.0 3.5 3.0 2.5 2.0 1.5 1.0 0.5 0.0 ppm
2.02
1.99
2.10
2.87
0.99
1.01
2.00
2.98

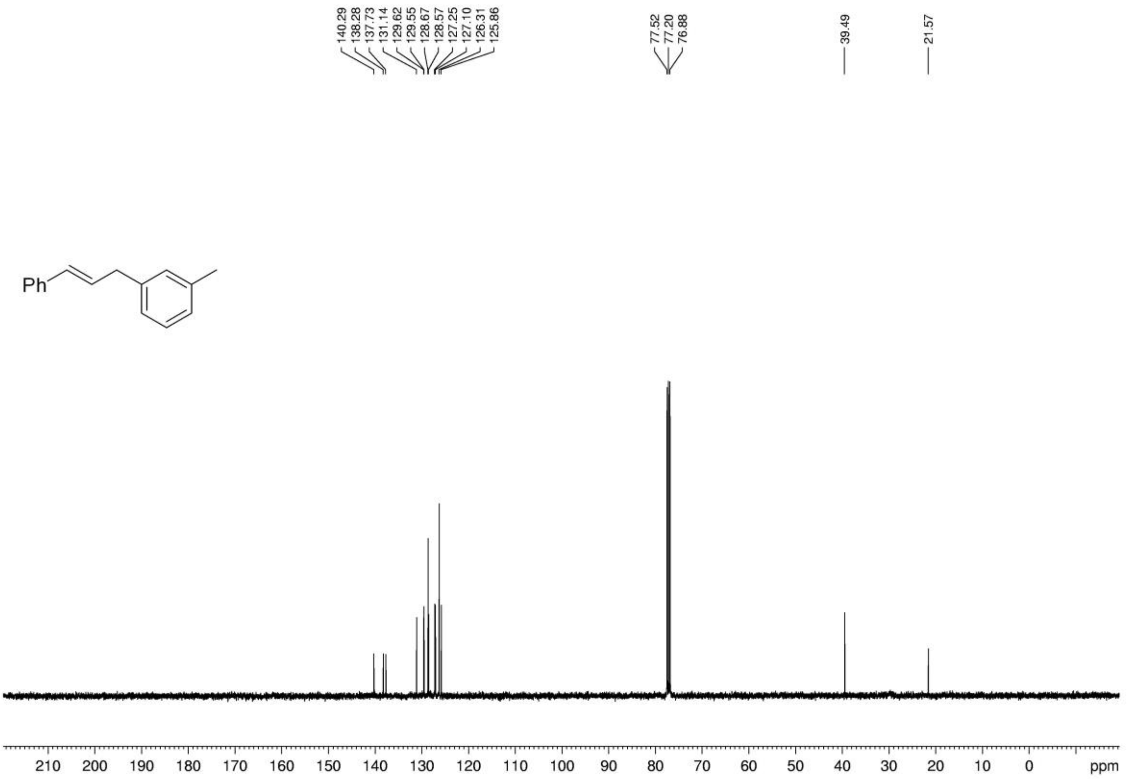

140.29
138.28
137.73
131.14
129.62
129.55
128.67
128.57
127.25
127.10
126.31
125.86
77.52
77.20
76.88
39.49
21.57
Ph
210 200 190 180 170 160 150 140 130 120 110 100 90 80 70 60 50 40 30 20 10 0 ppm

6

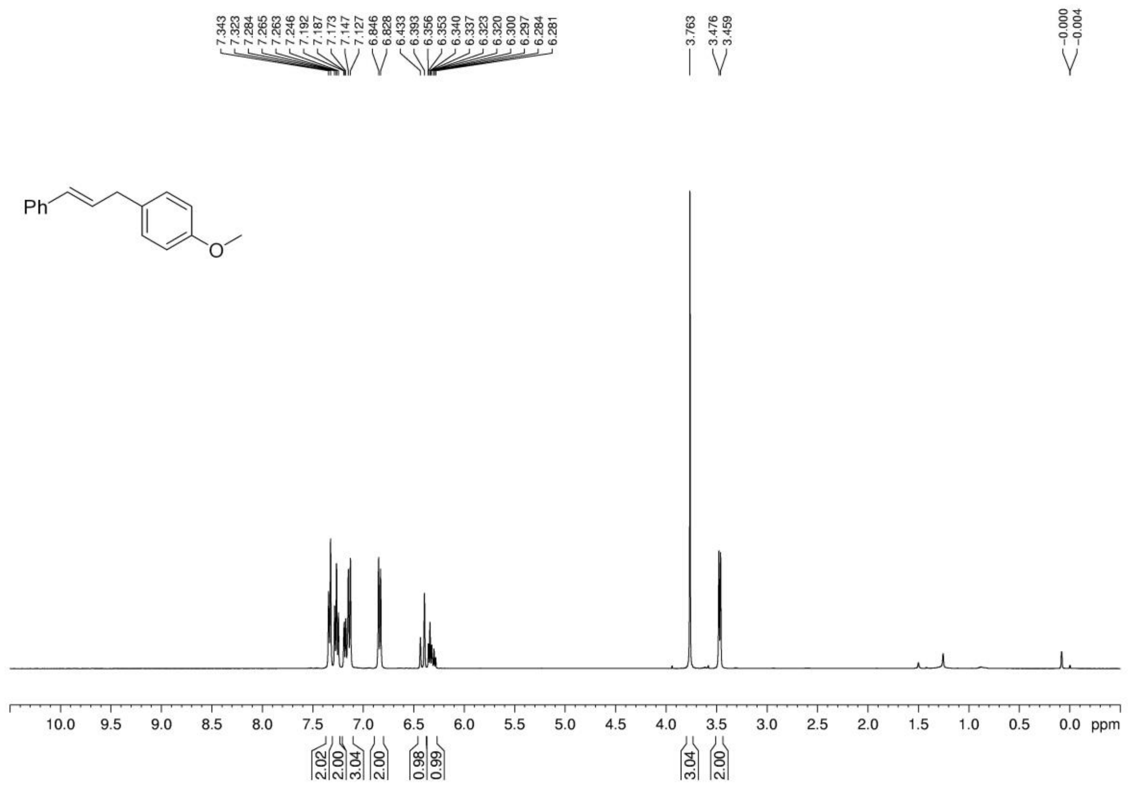
Ph
O
7.343
7.323
7.284
7.265
7.263
7.246
7.192
7.187
7.173
7.147
7.127
6.846
6.828
6.433
6.393
6.356
6.353
6.340
6.337
6.323
6.320
6.300
6.297
6.284
6.281
3.763
3.476
3.459
0.000
-0.004
10.0 9.5 9.0 8.5 8.0 7.5 7.0 6.5 6.0 5.5 5.0 4.5 4.0 3.5 3.0 2.5 2.0 1.5 1.0 0.5 0.0 ppm
2.02
2.00
3.04
2.00
0.98
0.99
3.04
2.00

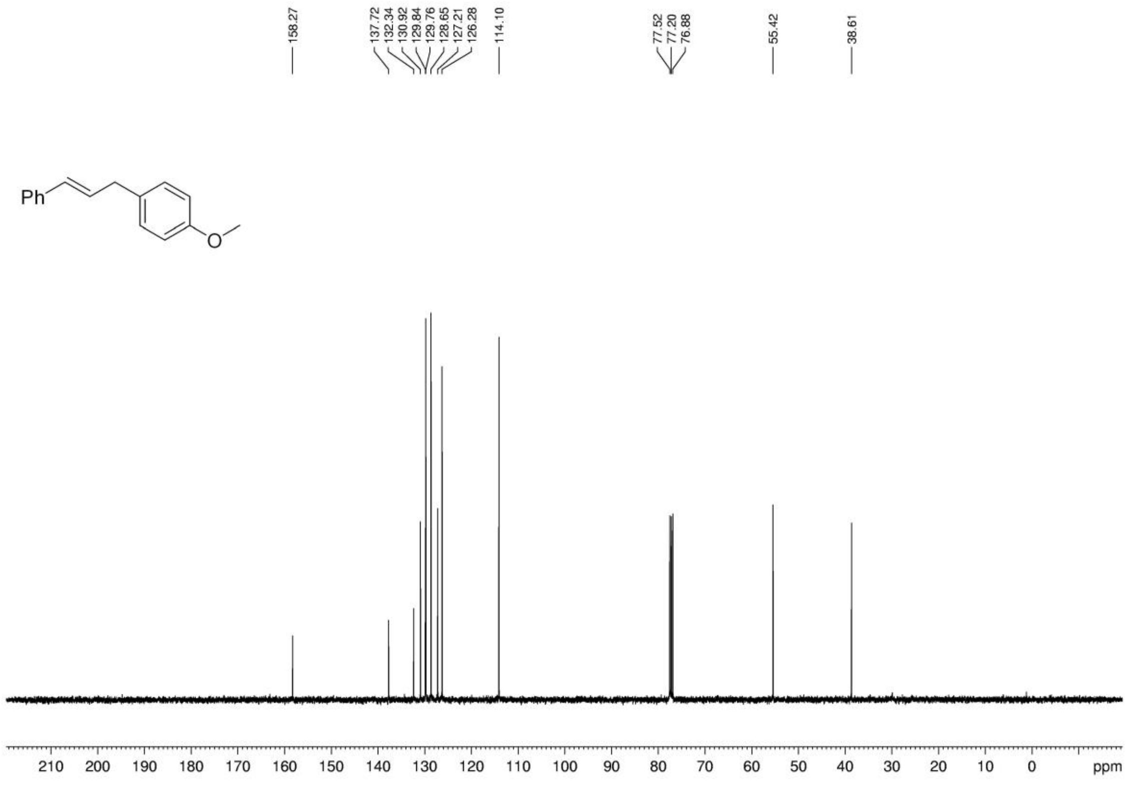
Ph
O
158.27
137.72
132.34
130.92
129.84
129.76
128.65
127.21
126.28
114.10
77.52
77.20
76.88
55.42
38.61
210 200 190 180 170 160 150 140 130 120 110 100 90 80 70 60 50 40 30 20 10 0 ppm

7

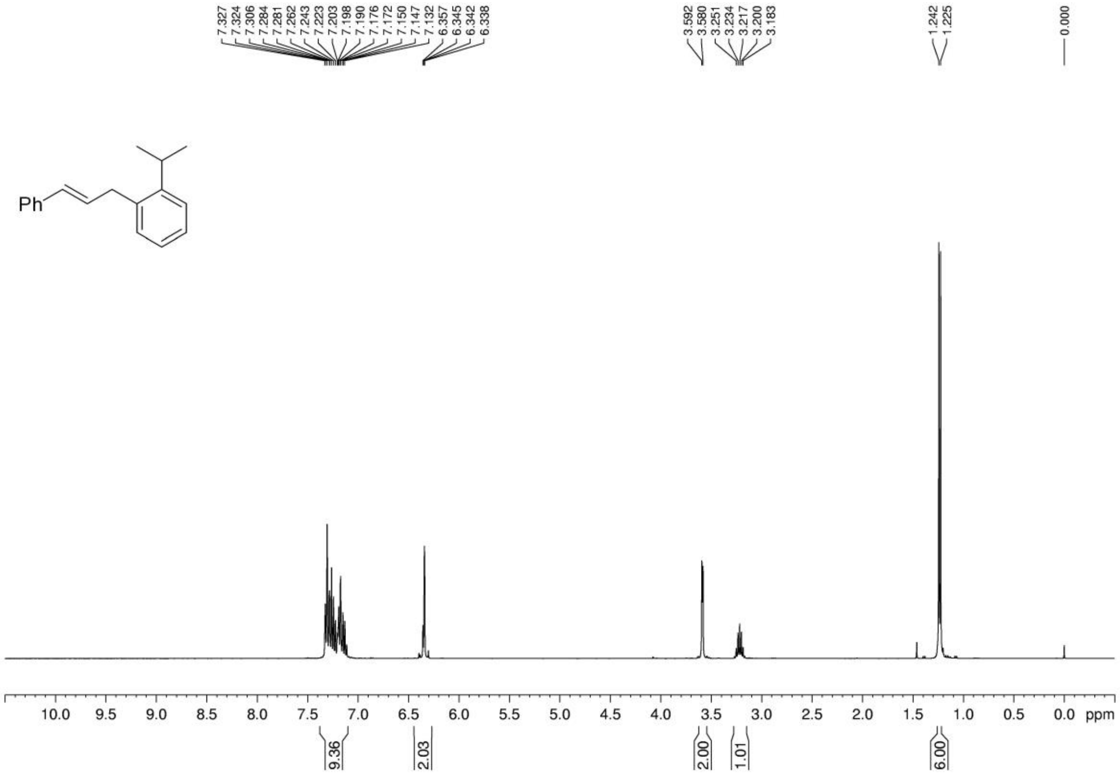
7.327
7.324
7.306
7.284
7.281
7.262
7.243
7.223
7.203
7.198
7.190
7.176
7.172
7.150
7.147
7.132
6.357
6.345
6.342
6.338
3.592
3.580
3.251
3.234
3.217
3.200
3.183
1.242
1.225
0.000
Ph
10.0 9.5 9.0 8.5 8.0 7.5 7.0 6.5 6.0 5.5 5.0 4.5 4.0 3.5 3.0 2.5 2.0 1.5 1.0 0.5 0.0 ppm
9.36
2.03
2.00
1.01
6.00

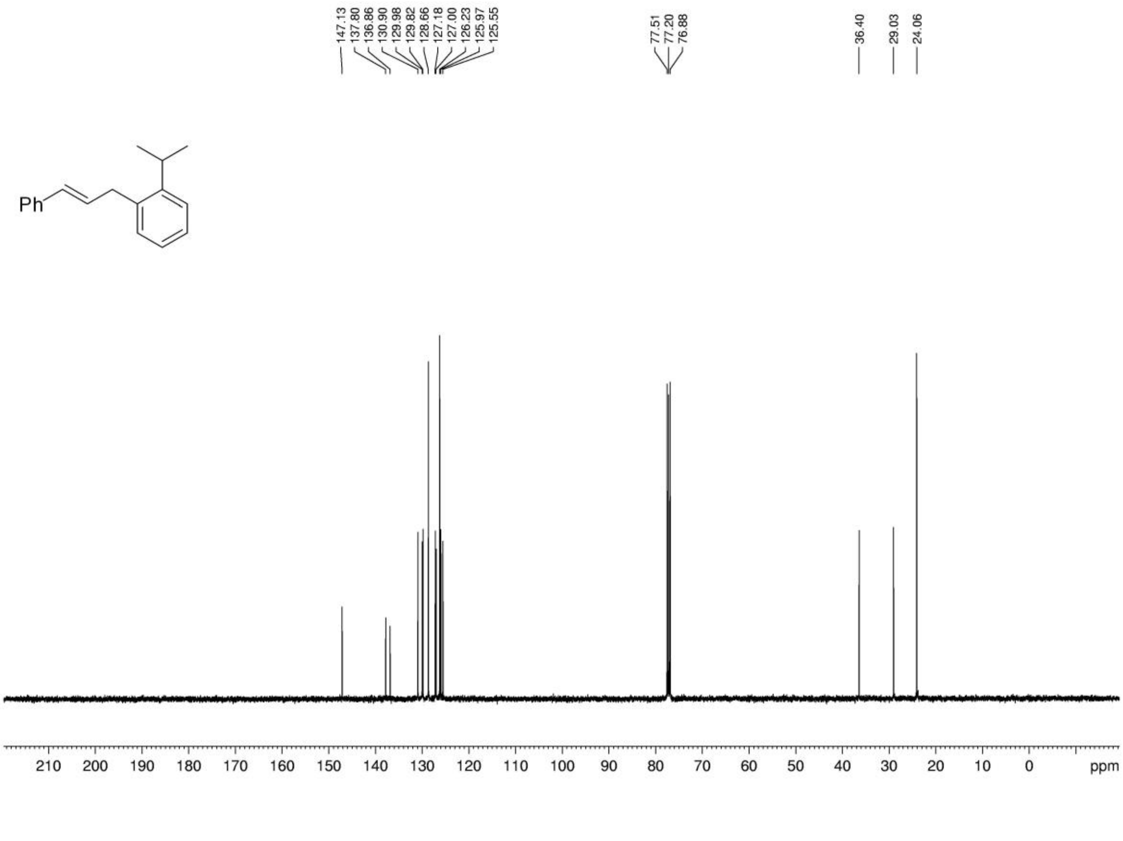
147.13
137.80
136.86
130.90
129.98
129.82
128.66
127.18
127.00
126.23
125.97
125.55
77.51
77.20
76.88
36.40
29.03
24.06
Ph
210 200 190 180 170 160 150 140 130 120 110 100 90 80 70 60 50 40 30 20 10 0 ppm

8

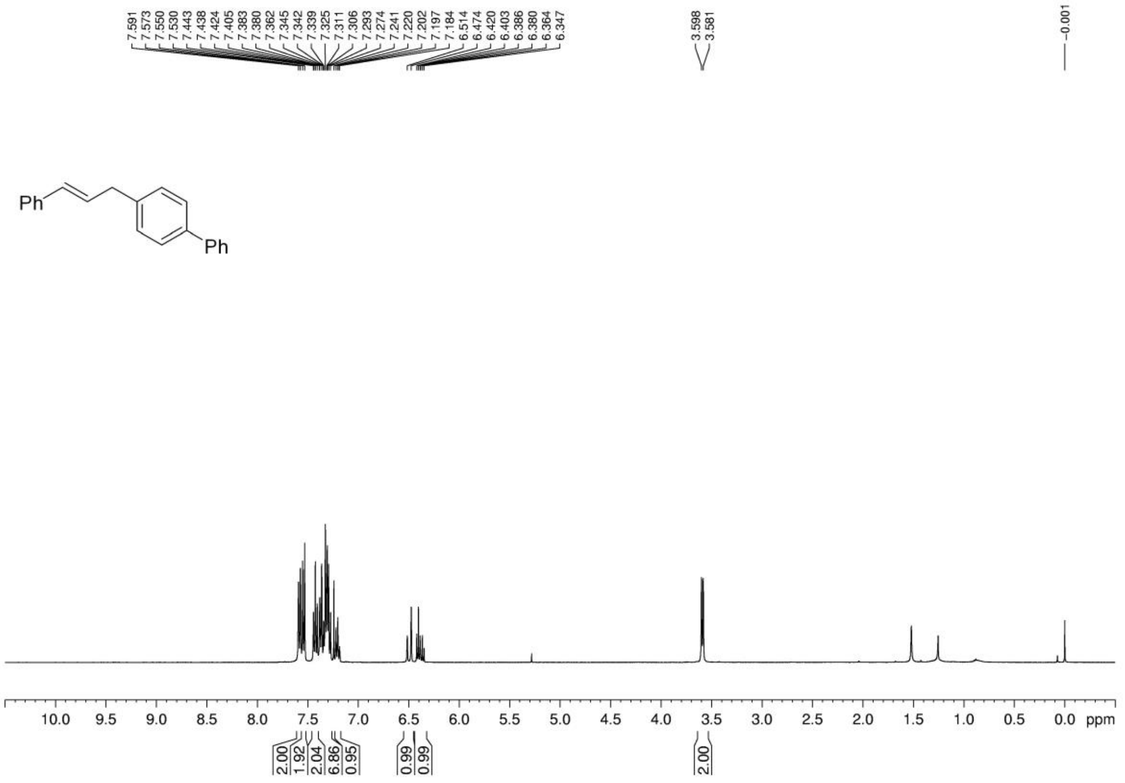
7.591
7.573
7.550
7.530
7.443
7.438
7.424
7.405
7.383
7.380
7.362
7.345
7.342
7.339
7.325
7.311
7.306
7.293
7.274
7.241
7.220
7.202
7.197
7.184
6.514
6.474
6.420
6.403
6.386
6.380
6.364
6.347
3.598
3.581
-0.001
Ph
Ph
10.0 9.5 9.0 8.5 8.0 7.5 7.0 6.5 6.0 5.5 5.0 4.5 4.0 3.5 3.0 2.5 2.0 1.5 1.0 0.5 0.0 ppm
2.00
1.92
2.04
6.86
0.95
0.99
0.99
2.00

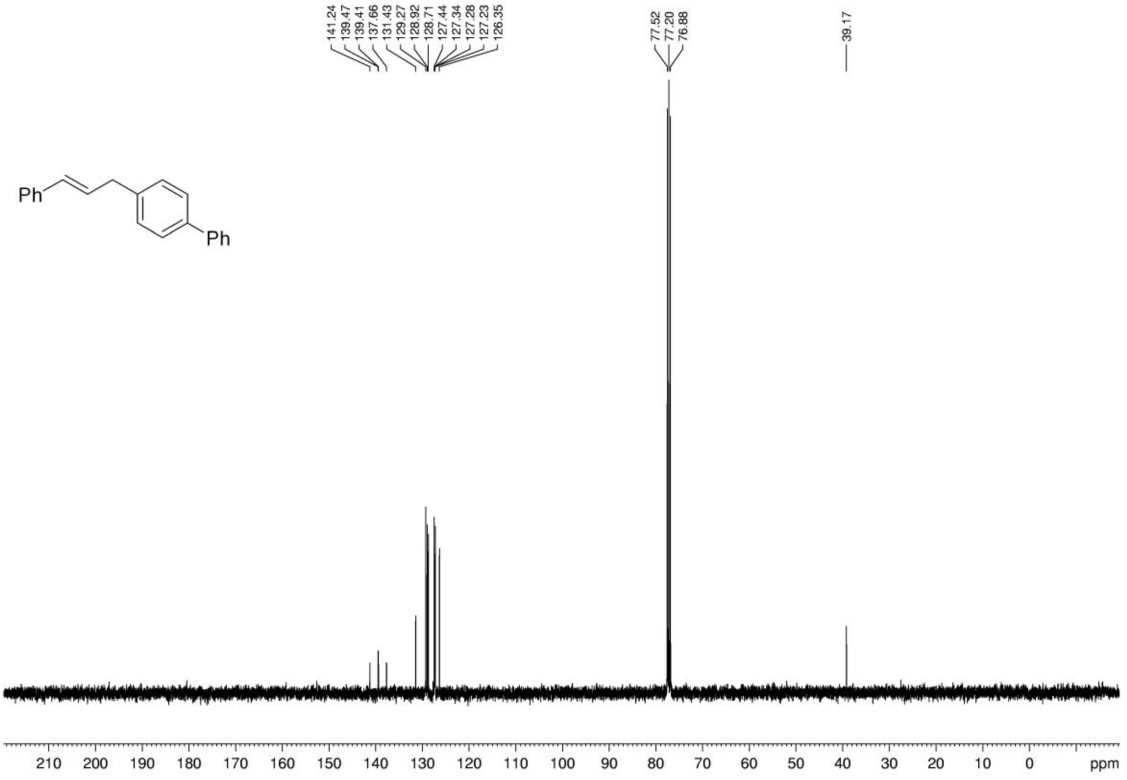
141.24
139.47
139.41
137.66
131.43
129.27
128.92
128.71
127.44
127.34
127.28
127.23
126.35
77.52
77.20
76.88
39.17
Ph
Ph
210 200 190 180 170 160 150 140 130 120 110 100 90 80 70 60 50 40 30 20 10 0 ppm

9

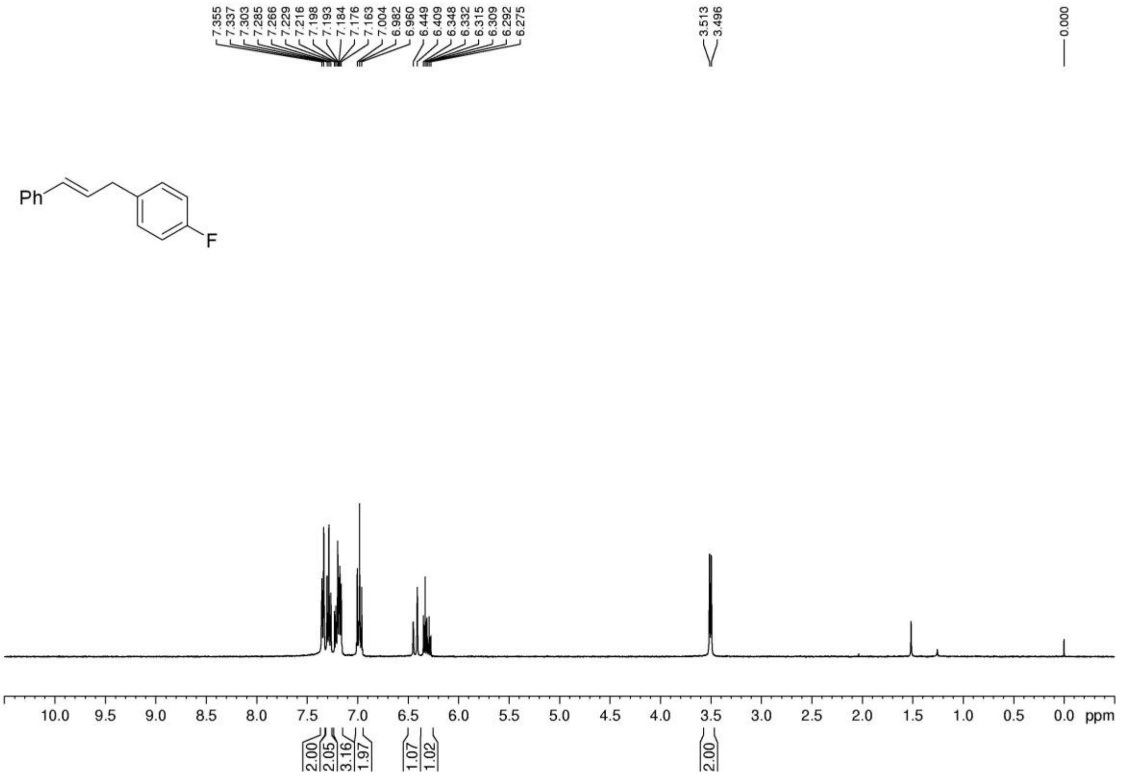
7.355
7.337
7.303
7.285
7.266
7.229
7.216
7.198
7.193
7.184
7.176
7.163
7.004
6.982
6.960
6.449
6.409
6.348
6.332
6.315
6.309
6.292
6.275
3.513
3.496
0.000
Ph
F
10.0 9.5 9.0 8.5 8.0 7.5 7.0 6.5 6.0 5.5 5.0 4.5 4.0 3.5 3.0 2.5 2.0 1.5 1.0 0.5 0.0 ppm
2.00
2.05
3.16
1.97
1.07
1.02
2.00

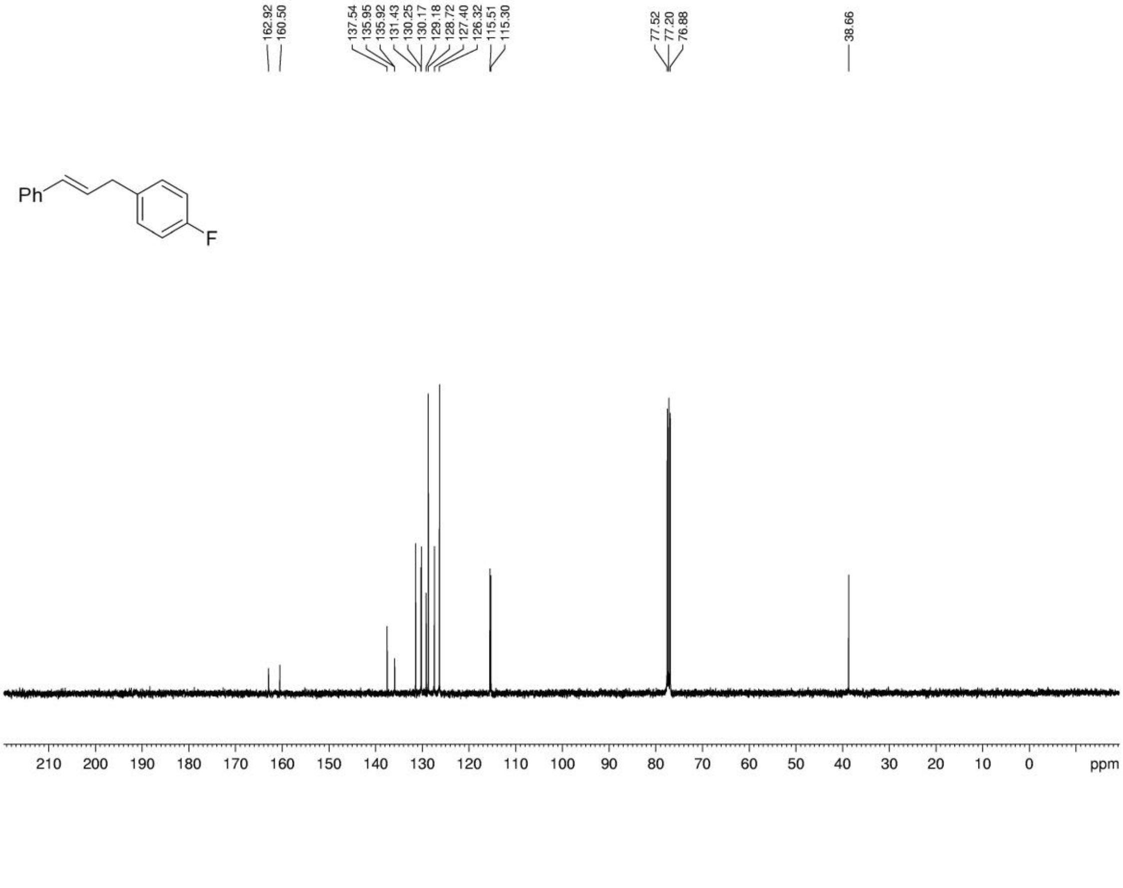
162.92
160.50
137.54
135.95
135.92
131.43
130.25
130.17
129.18
128.72
127.40
126.32
115.51
115.30
77.52
77.20
76.88
38.66
Ph
F
210 200 190 180 170 160 150 140 130 120 110 100 90 80 70 60 50 40 30 20 10 0 ppm

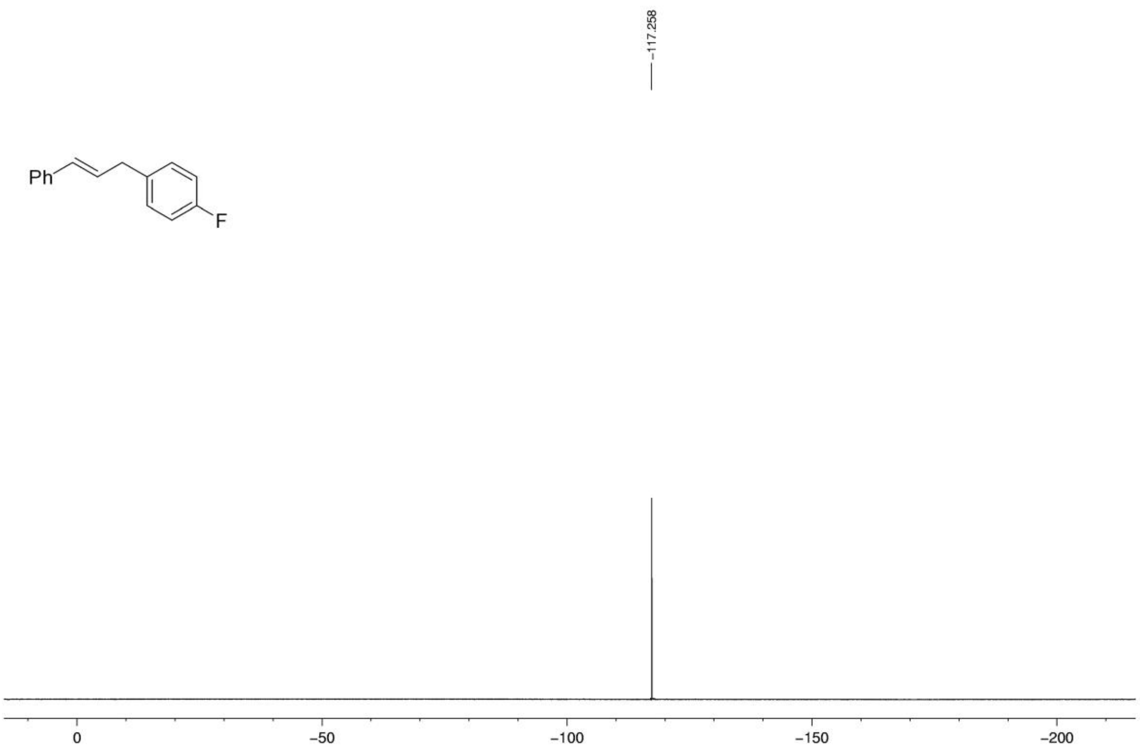

−117.258
Ph
F
0
−50
−100
−150
−200

10

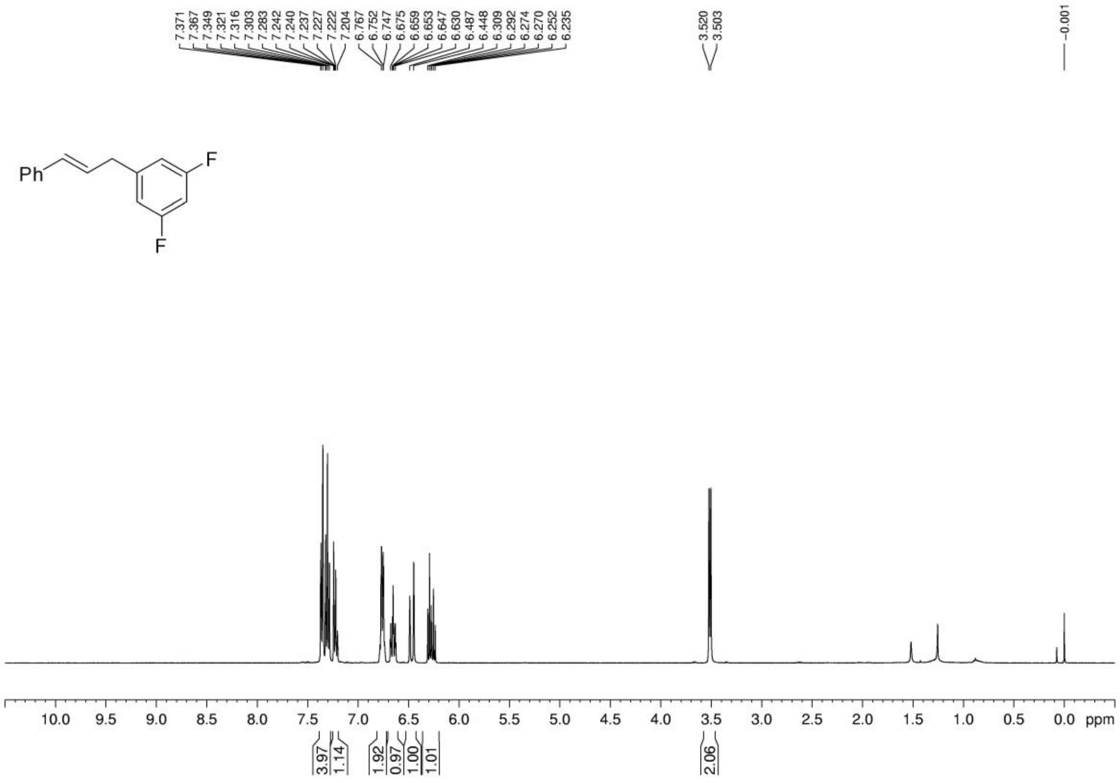

7.371 7.367 7.349 7.321 7.316 7.303 7.283 7.242 7.240 7.237 7.227 7.222 7.204 6.767 6.752 6.747 6.675 6.659 6.653 6.647 6.630 6.487 6.448 6.309 6.292 6.274 6.270 6.252 6.235
3.520 3.503
-0.001
Ph
F
F
10.0 9.5 9.0 8.5 8.0 7.5 7.0 6.5 6.0 5.5 5.0 4.5 4.0 3.5 3.0 2.5 2.0 1.5 1.0 0.5 0.0 ppm
3.97 1.14 1.92 0.97 1.00 1.01
2.06

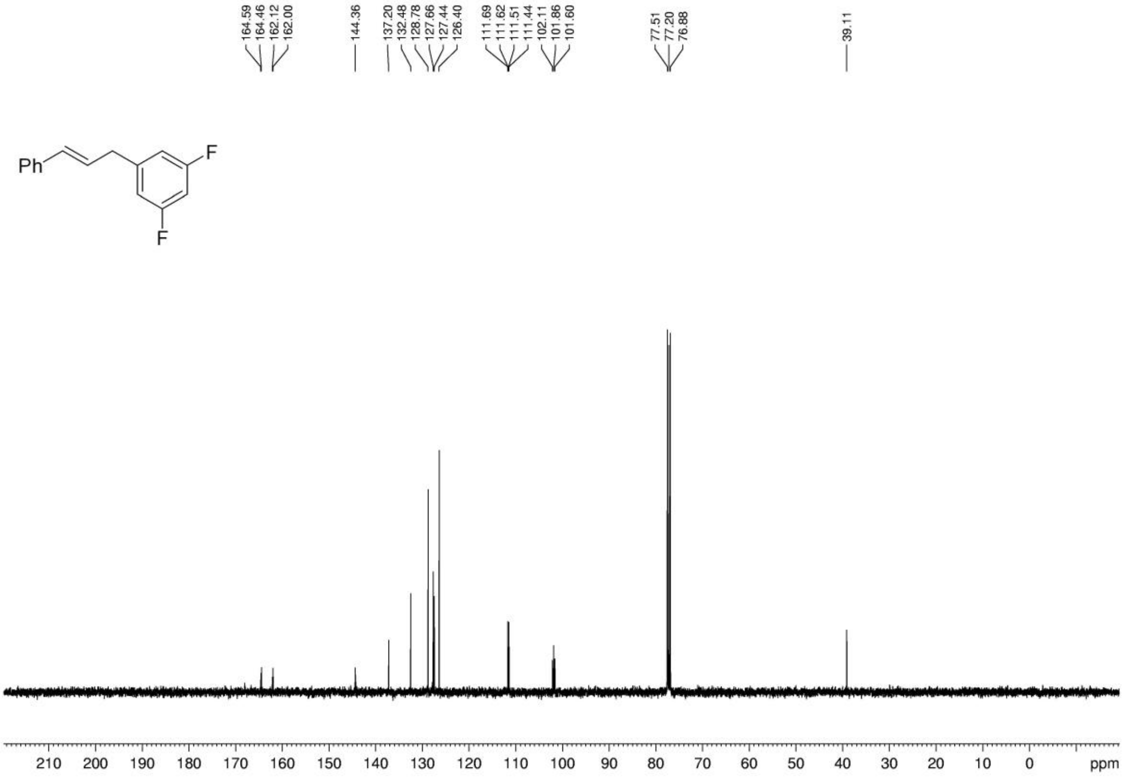

164.59 164.46 162.12 162.00
144.36
137.20 132.48 128.78 127.66 127.44 126.40
111.69 111.62 111.51 111.44 102.11 101.86 101.60
77.51 77.20 76.88
39.11
Ph
F
F
210 200 190 180 170 160 150 140 130 120 110 100 90 80 70 60 50 40 30 20 10 0 ppm

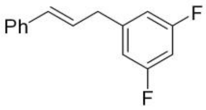
Ph
F
F

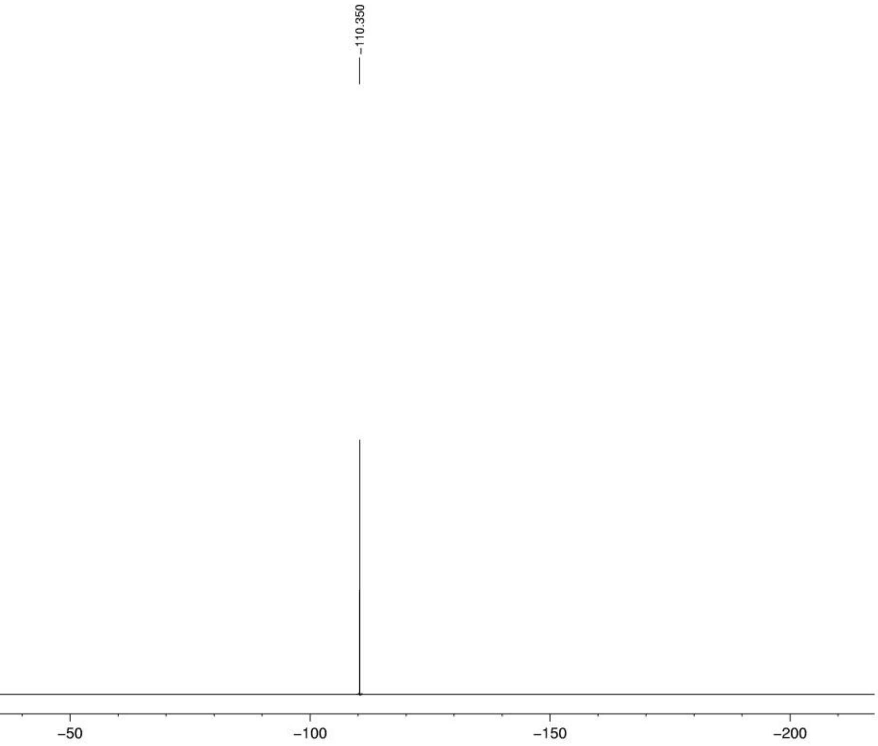
−110.350
0
−50
−100
−150
−200

11

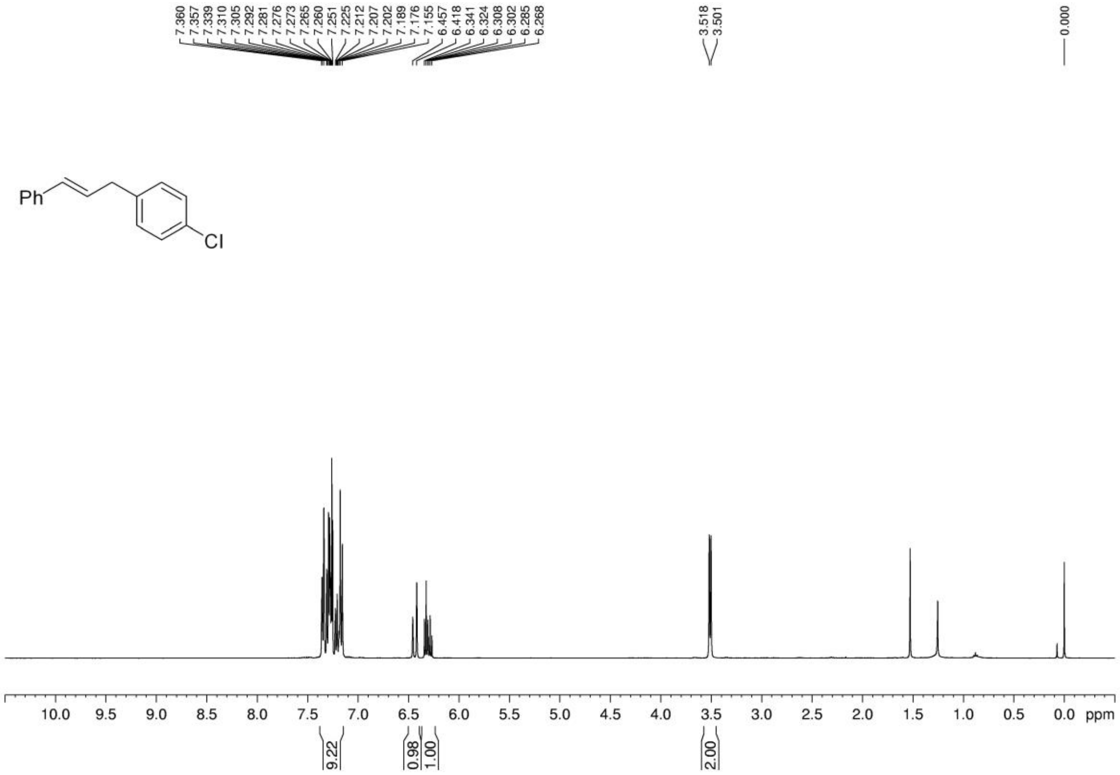
7.360
7.357
7.339
7.310
7.305
7.292
7.281
7.276
7.273
7.265
7.260
7.251
7.225
7.212
7.207
7.202
7.189
7.176
7.155
6.457
6.418
6.341
6.324
6.308
6.302
6.285
6.268
3.518
3.501
0.000
Ph
Cl
10.0 9.5 9.0 8.5 8.0 7.5 7.0 6.5 6.0 5.5 5.0 4.5 4.0 3.5 3.0 2.5 2.0 1.5 1.0 0.5 0.0 ppm
9.22
0.98
1.00
2.00

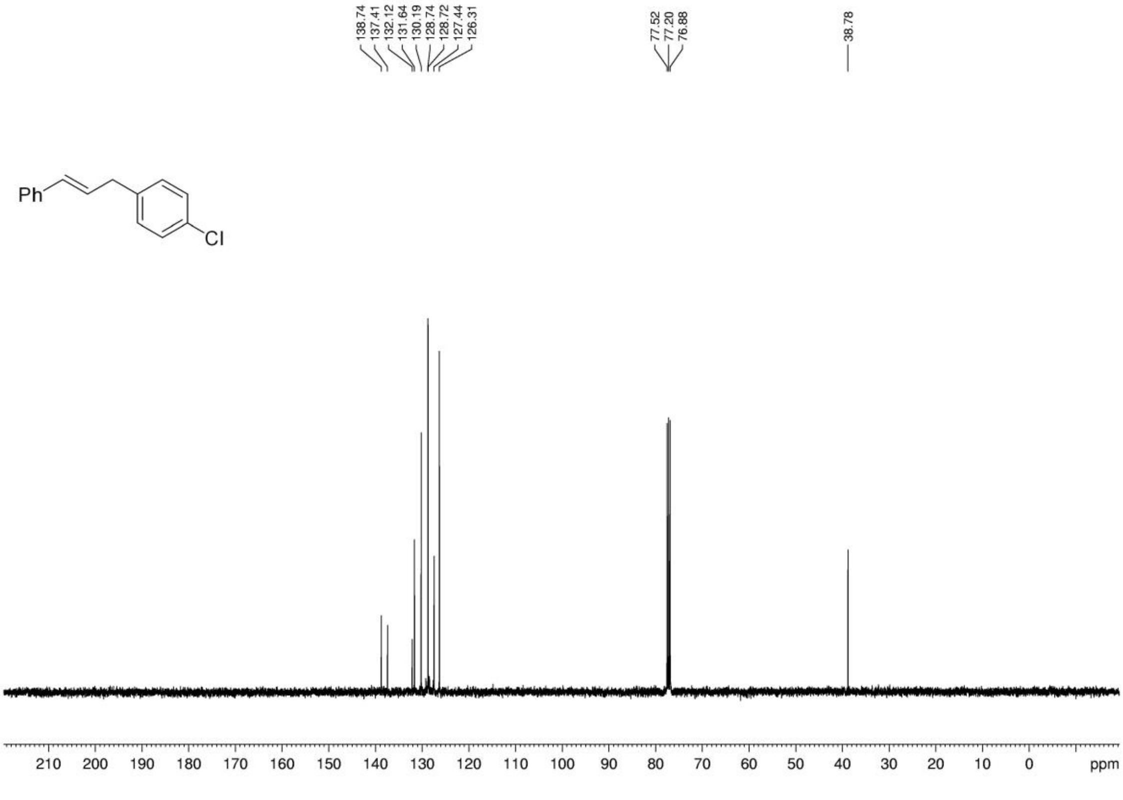
138.74
137.41
132.12
131.64
130.19
128.74
128.72
127.44
126.31
77.52
77.20
76.88
38.78
Ph
Cl
210 200 190 180 170 160 150 140 130 120 110 100 90 80 70 60 50 40 30 20 10 0 ppm

12

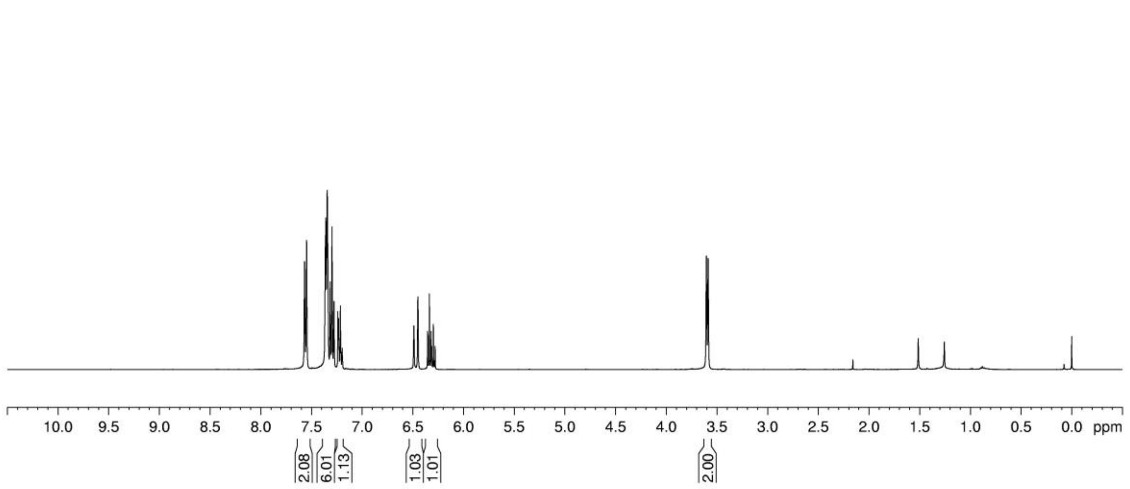

7.567
7.547
7.357
7.343
7.338
7.314
7.309
7.296
7.277
7.239
7.235
7.232
7.219
7.214
7.208
7.196
6.489
6.449
6.353
6.336
6.319
6.314
6.297
6.279
3.603
3.586
0.000
10.0 9.5 9.0 8.5 8.0 7.5 7.0 6.5 6.0 5.5 5.0 4.5 4.0 3.5 3.0 2.5 2.0 1.5 1.0 0.5 0.0 ppm
2.08
6.01
1.13
1.03
1.01
2.00

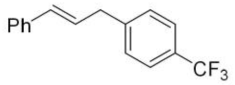

Ph
CF3

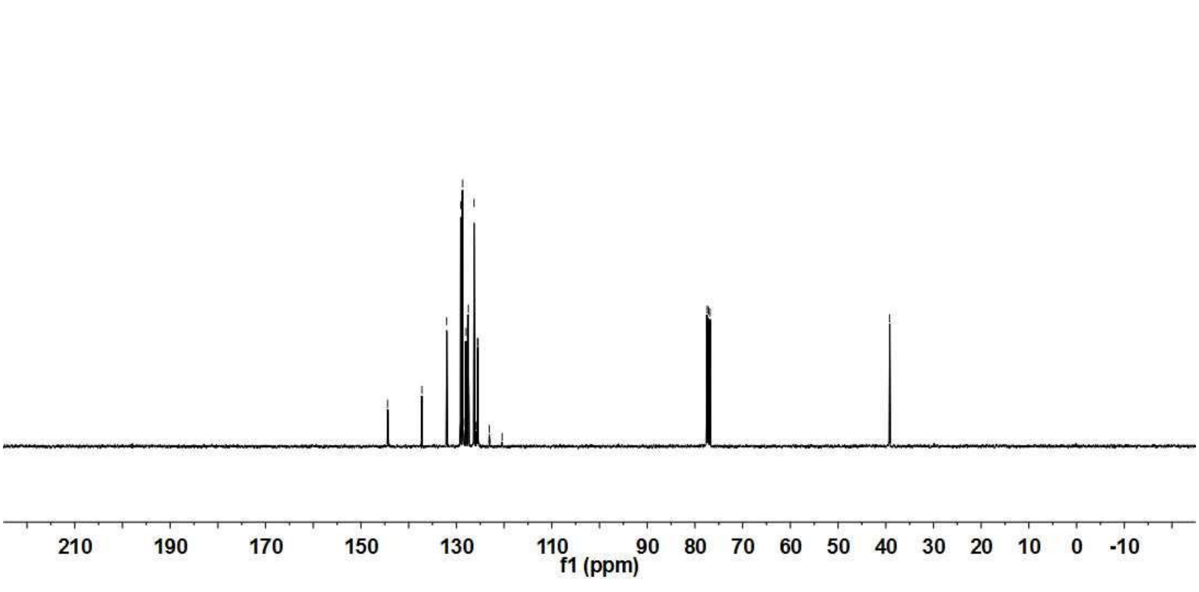

144.41
137.22
132.04
129.09
128.86
128.70
128.53
128.21
128.02
127.51
126.29
125.81
125.58
125.54
125.51
125.47
123.10
120.40
77.46
77.14
76.82
39.19
210 190 170 150 130 110 90 80 70 60 50 40 30 20 10 0 -10
f1 (ppm)

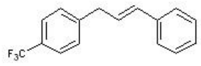

F3C

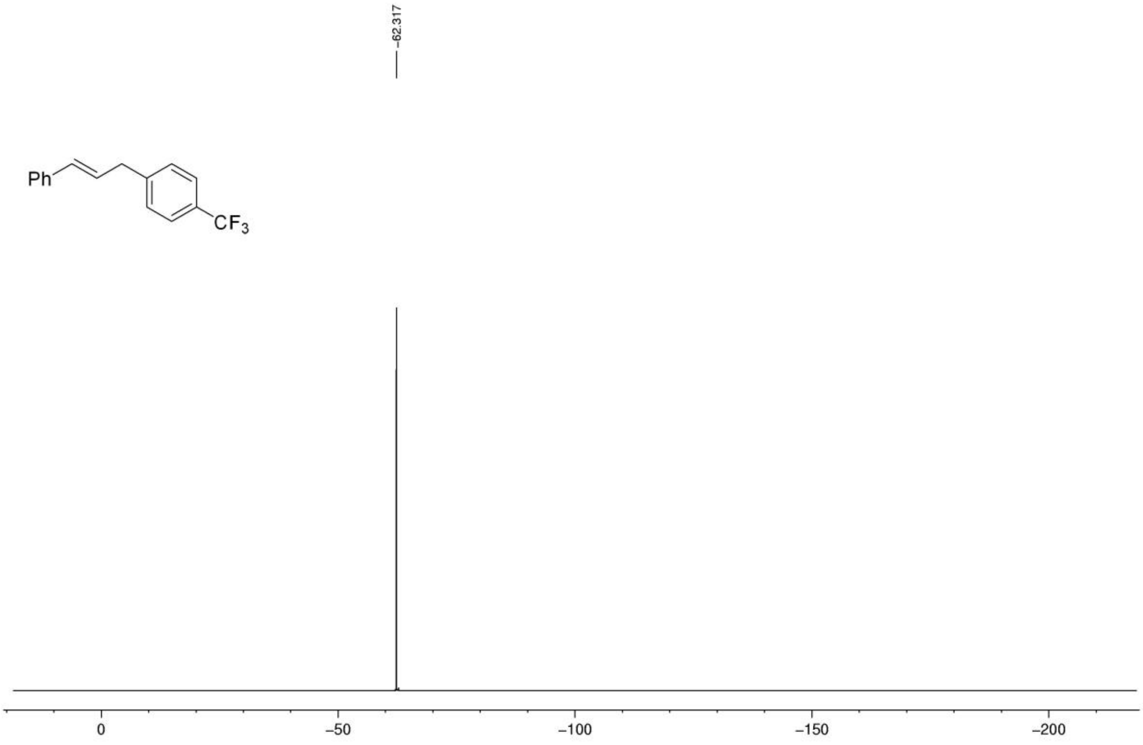

-62.317
Ph
CF3
0
−50
−100
−150
−200

13

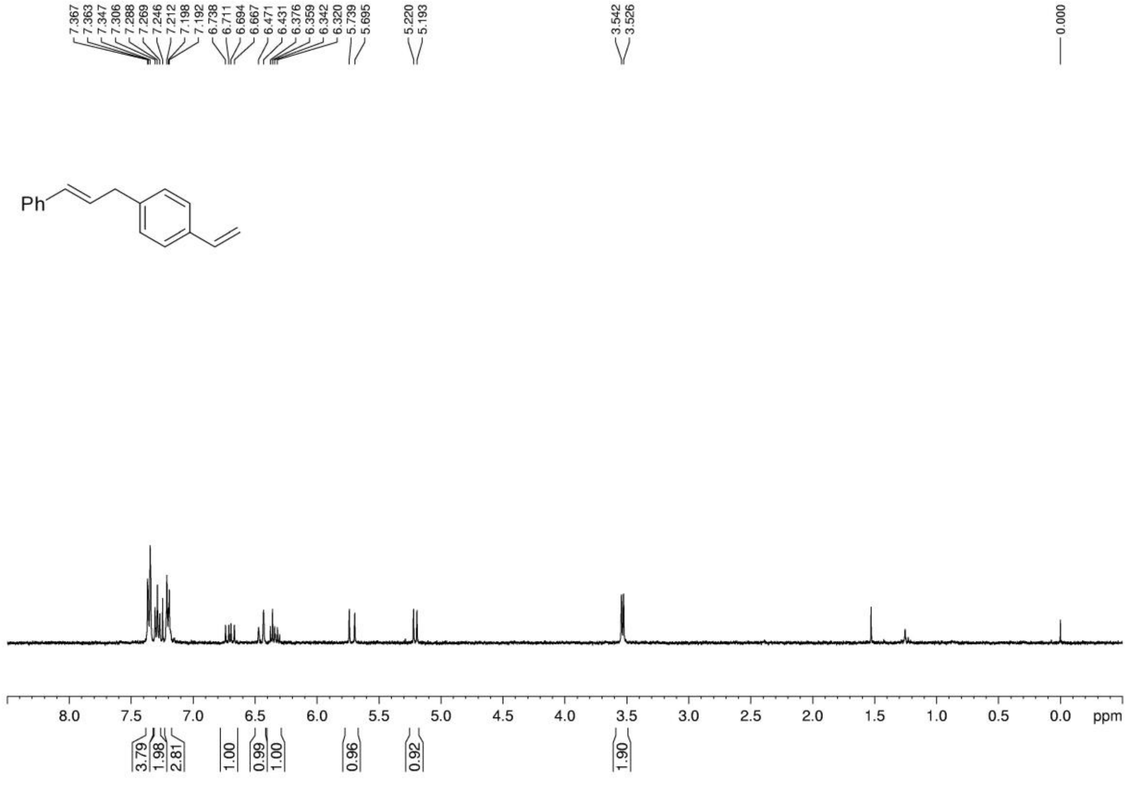
7.367
7.363
7.347
7.306
7.288
7.269
7.246
7.212
7.198
7.192
6.738
6.711
6.694
6.667
6.471
6.431
6.376
6.359
6.342
6.320
5.739
5.695
5.220
5.193
3.542
3.526
0.000
Ph
8.0 7.5 7.0 6.5 6.0 5.5 5.0 4.5 4.0 3.5 3.0 2.5 2.0 1.5 1.0 0.5 0.0 ppm
3.79
1.98
2.81
1.00
0.99
1.00
0.96
0.92
1.90

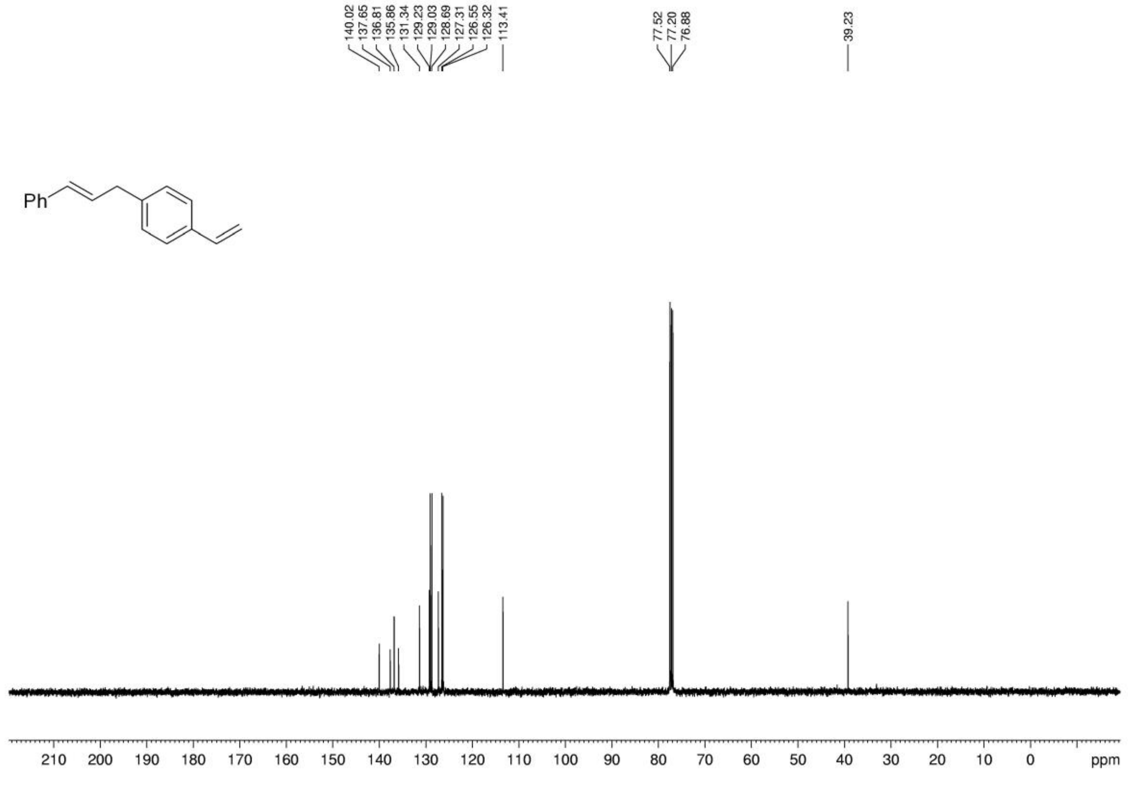
140.02
137.65
136.81
135.86
131.34
129.23
129.03
128.69
127.31
126.55
126.32
113.41
77.52
77.20
76.88
39.23
Ph
210 200 190 180 170 160 150 140 130 120 110 100 90 80 70 60 50 40 30 20 10 0 ppm

14

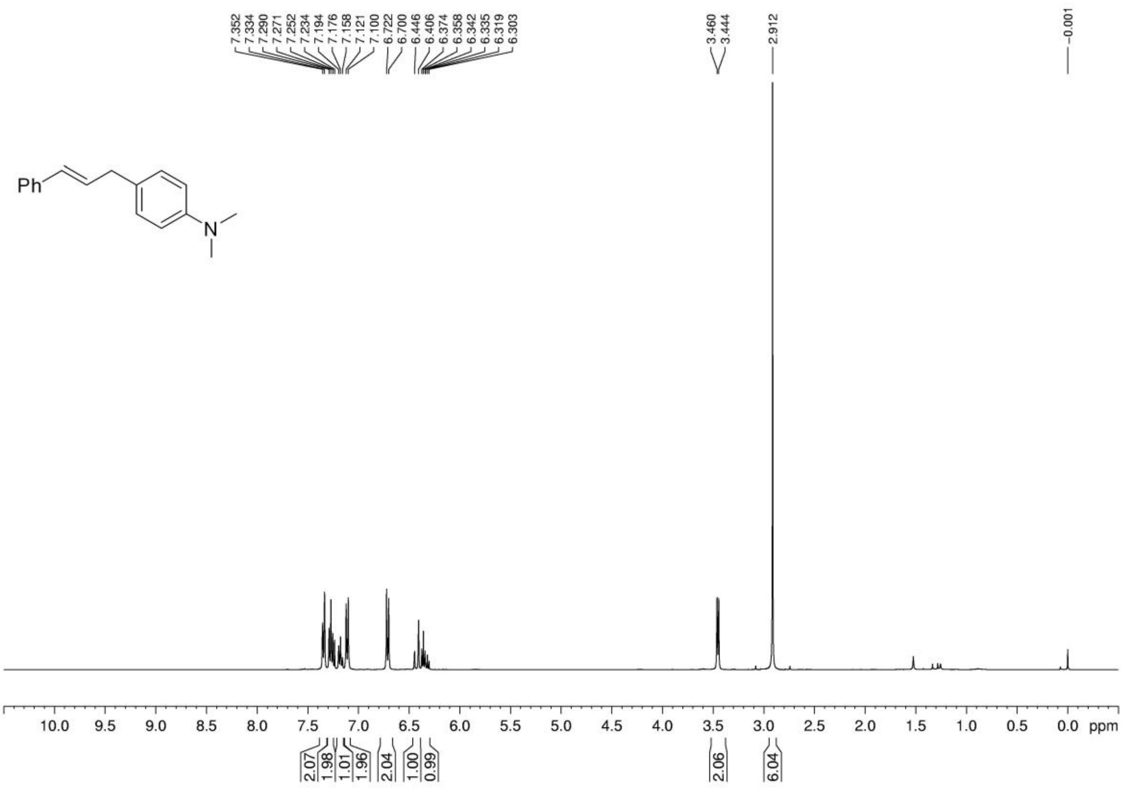
7.352
7.334
7.290
7.271
7.252
7.234
7.194
7.176
7.158
7.121
7.100
6.722
6.700
6.446
6.406
6.374
6.358
6.342
6.335
6.319
6.303
3.460
3.444
2.912
-0.001
Ph
N
10.0 9.5 9.0 8.5 8.0 7.5 7.0 6.5 6.0 5.5 5.0 4.5 4.0 3.5 3.0 2.5 2.0 1.5 1.0 0.5 0.0 ppm
2.07
1.98
1.01
1.96
2.04
1.00
0.99
2.06
6.04

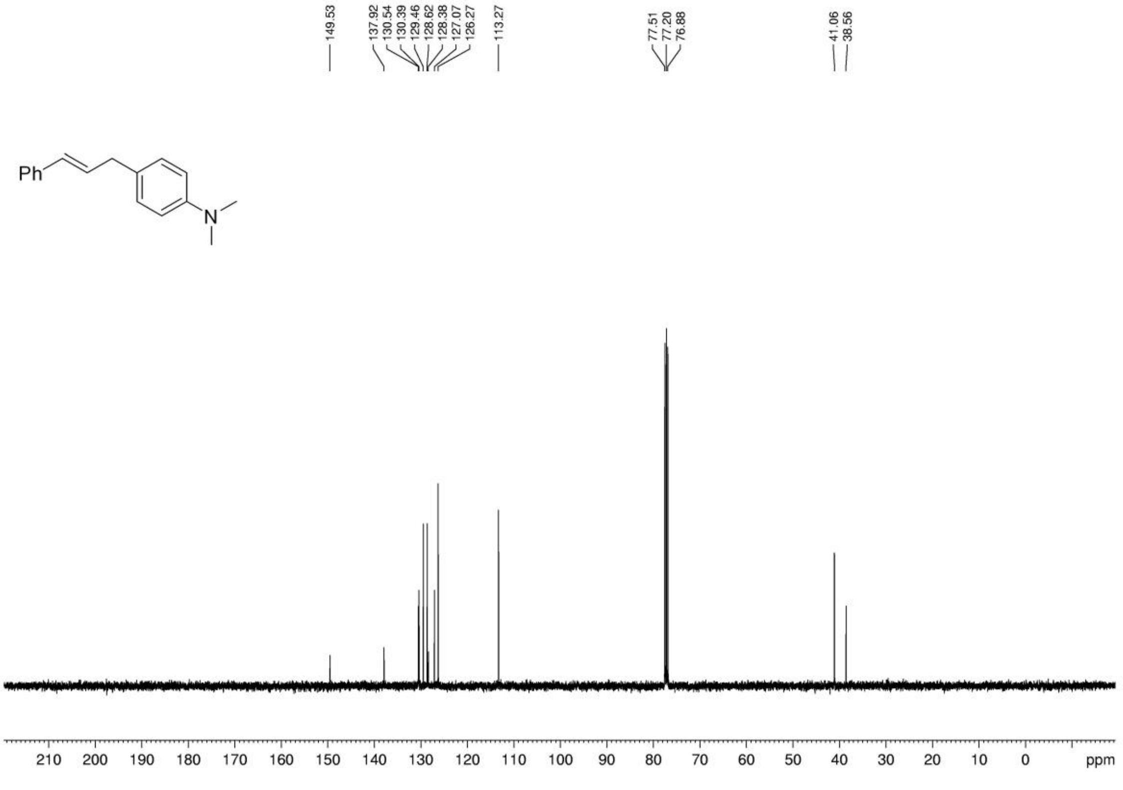
149.53
137.92
130.54
130.39
129.46
128.62
128.38
127.07
126.27
113.27
77.51
77.20
76.88
41.06
38.56
Ph
N
210 200 190 180 170 160 150 140 130 120 110 100 90 80 70 60 50 40 30 20 10 0 ppm

15

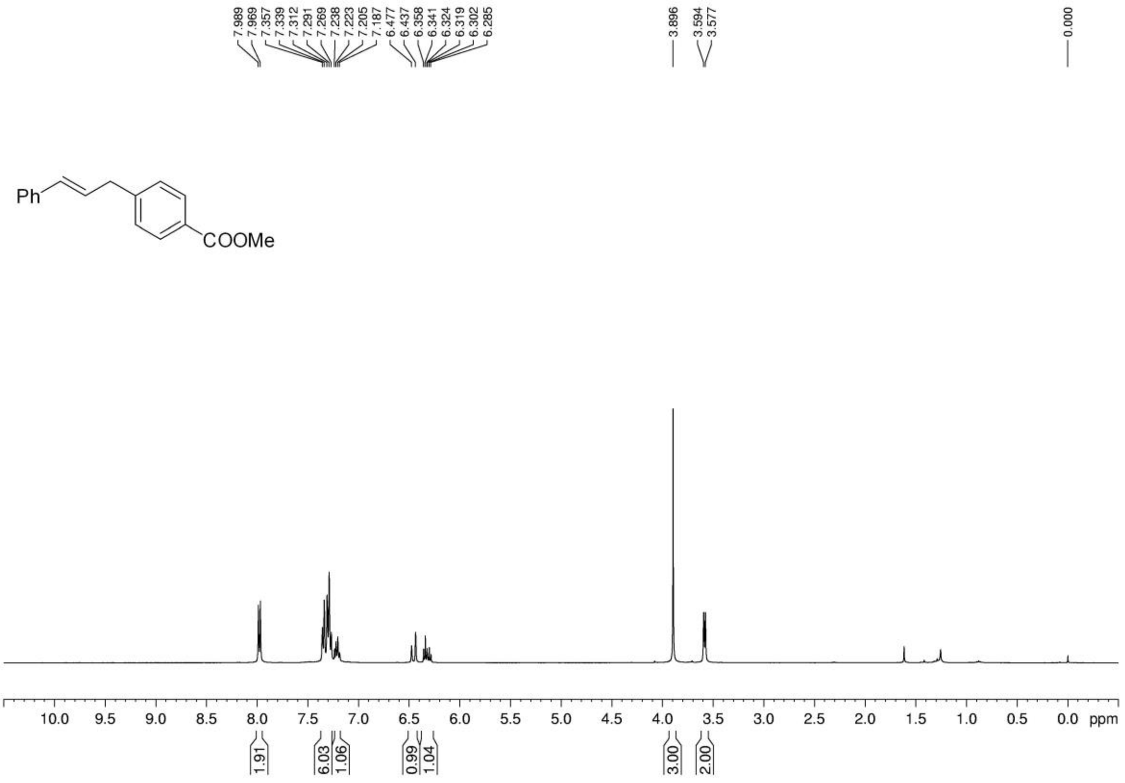
7.989 7.969 7.357 7.339 7.312 7.291 7.269 7.238 7.223 7.205 7.187 6.477 6.437 6.358 6.341 6.324 6.319 6.302 6.285
3.896 3.594 3.577
0.000
Ph
COOMe
10.0 9.5 9.0 8.5 8.0 7.5 7.0 6.5 6.0 5.5 5.0 4.5 4.0 3.5 3.0 2.5 2.0 1.5 1.0 0.5 0.0 ppm
1.91 6.03 1.06 0.99 1.04 3.00 2.00

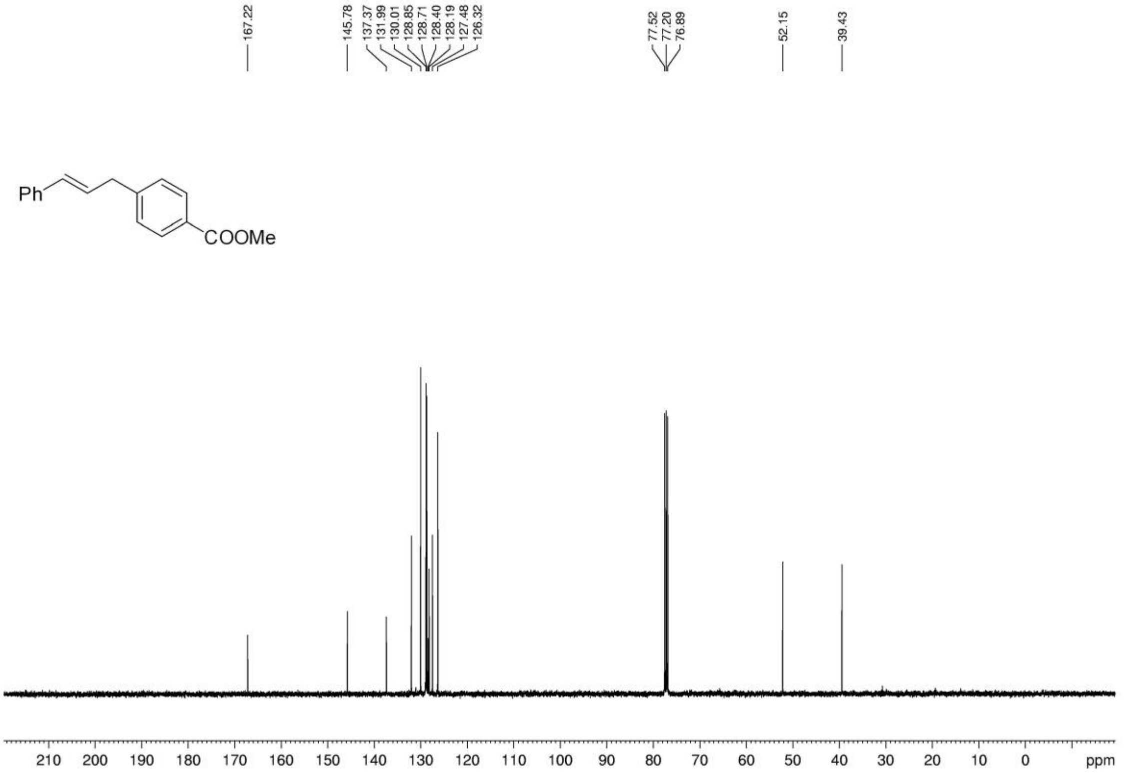
167.22
145.78 137.37 131.99 130.01 128.85 128.71 128.40 128.19 127.48 126.32
77.52 77.20 76.89
52.15
39.43
Ph
COOMe
210 200 190 180 170 160 150 140 130 120 110 100 90 80 70 60 50 40 30 20 10 0 ppm

16

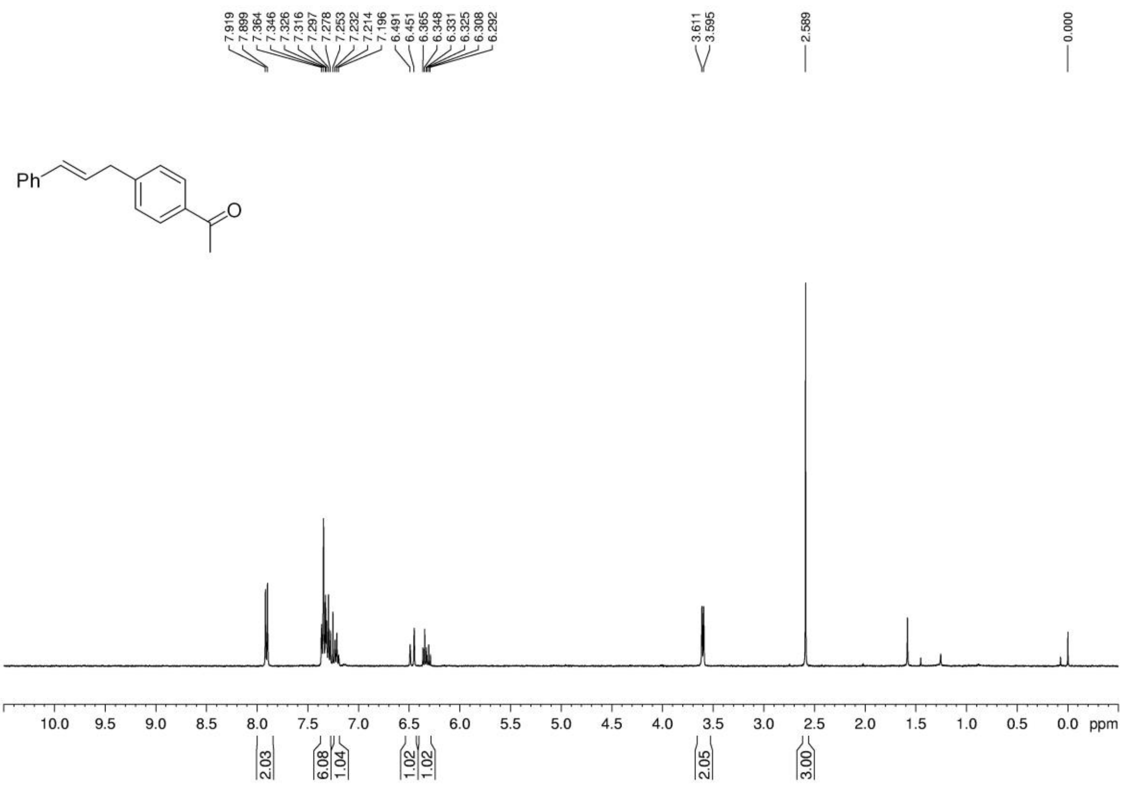

7.919
7.899
7.364
7.346
7.326
7.316
7.297
7.278
7.253
7.232
7.214
7.196
6.491
6.451
6.365
6.348
6.331
6.325
6.308
6.292
3.611
3.595
2.589
0.000
Ph
O
10.0 9.5 9.0 8.5 8.0 7.5 7.0 6.5 6.0 5.5 5.0 4.5 4.0 3.5 3.0 2.5 2.0 1.5 1.0 0.5 0.0 ppm
2.03
6.08
1.04
1.02
1.02
2.05
3.00

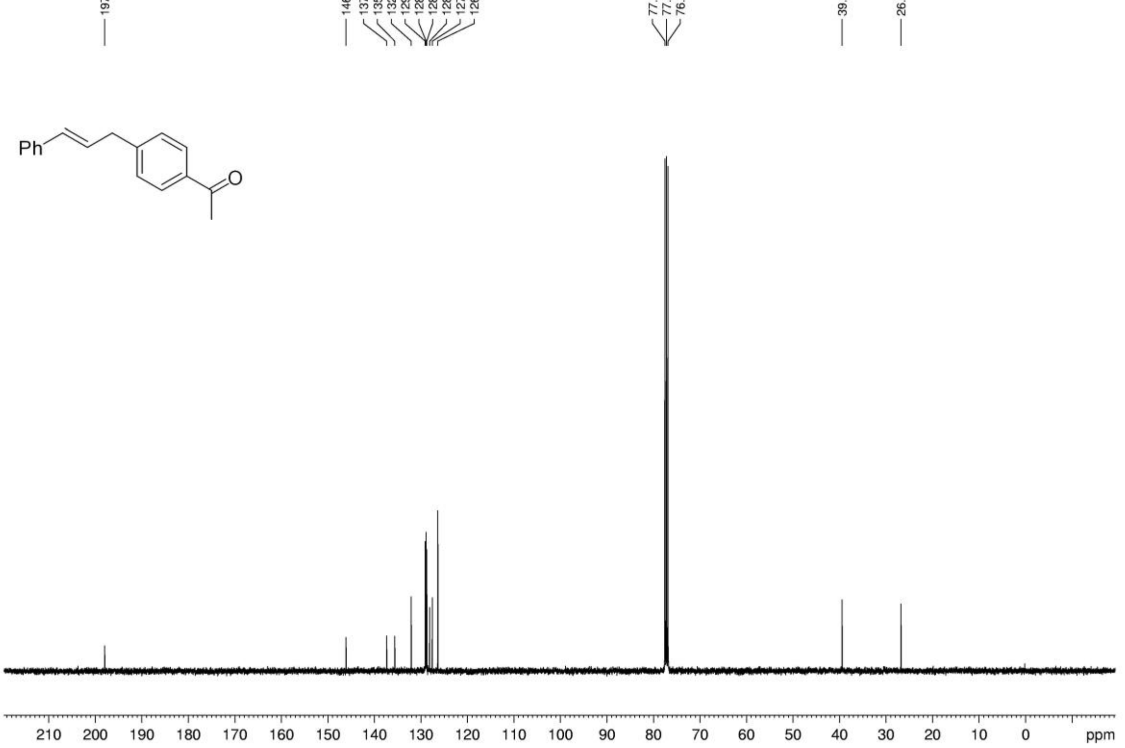

197.98
146.09
137.36
135.61
132.08
129.06
128.85
128.74
128.11
127.54
126.35
77.52
77.20
76.88
39.44
26.76
Ph
O
210 200 190 180 170 160 150 140 130 120 110 100 90 80 70 60 50 40 30 20 10 0 ppm

17

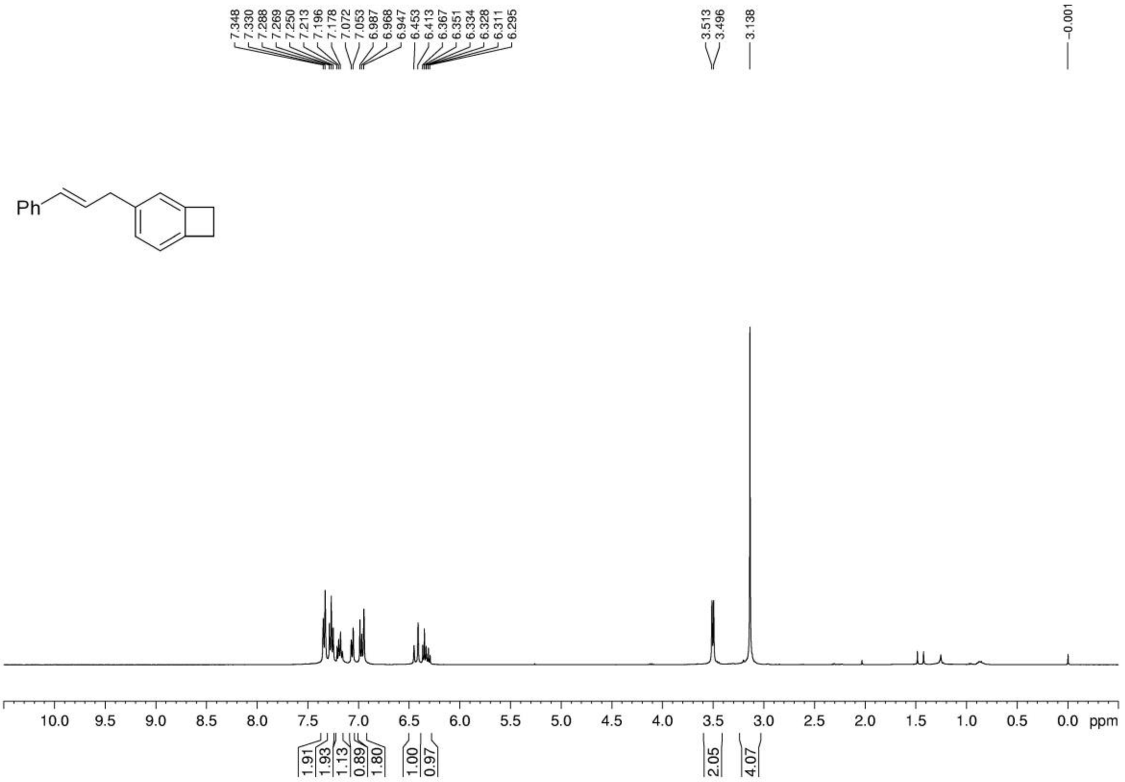
7.348
7.330
7.288
7.269
7.250
7.213
7.196
7.178
7.072
7.053
6.987
6.968
6.947
6.453
6.413
6.367
6.351
6.334
6.328
6.311
6.295
3.513
3.496
3.138
-0.001
Ph
10.0 9.5 9.0 8.5 8.0 7.5 7.0 6.5 6.0 5.5 5.0 4.5 4.0 3.5 3.0 2.5 2.0 1.5 1.0 0.5 0.0 ppm
1.91
1.93
1.13
0.89
1.80
1.00
0.97
2.05
4.07

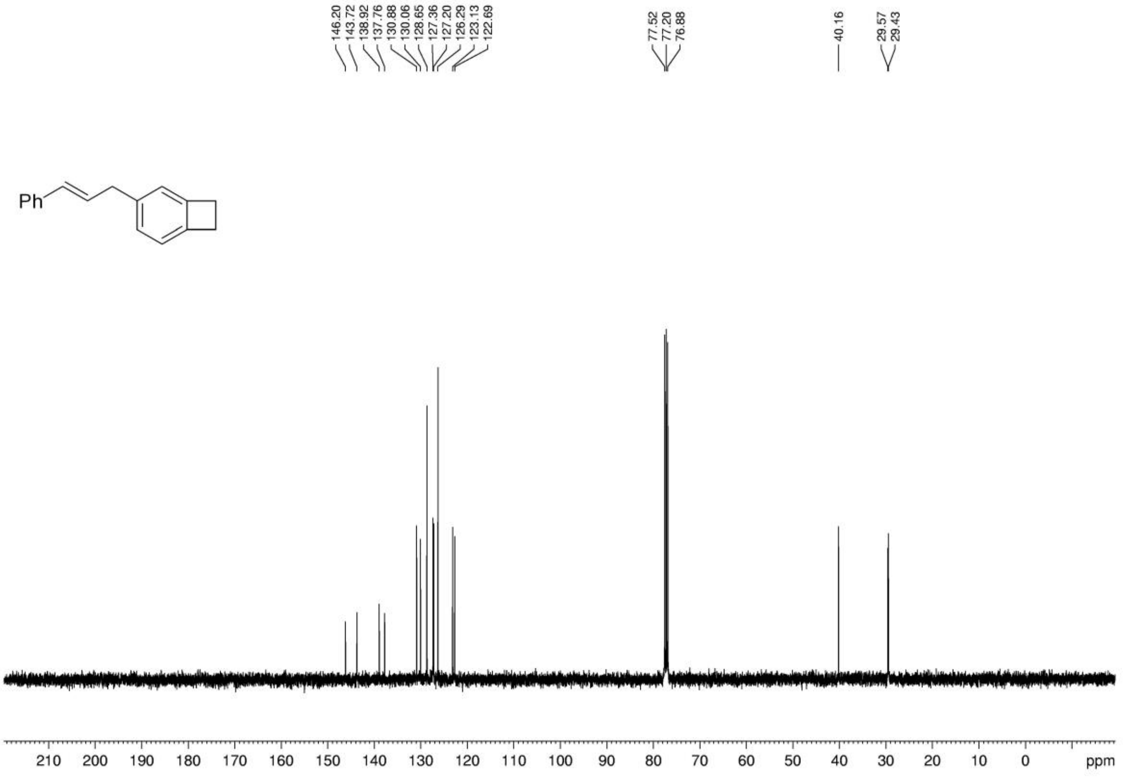
146.20
143.72
138.92
137.76
130.88
130.06
128.65
127.36
127.20
126.29
123.13
122.69
77.52
77.20
76.88
40.16
29.57
29.43
Ph
210 200 190 180 170 160 150 140 130 120 110 100 90 80 70 60 50 40 30 20 10 0 ppm

18

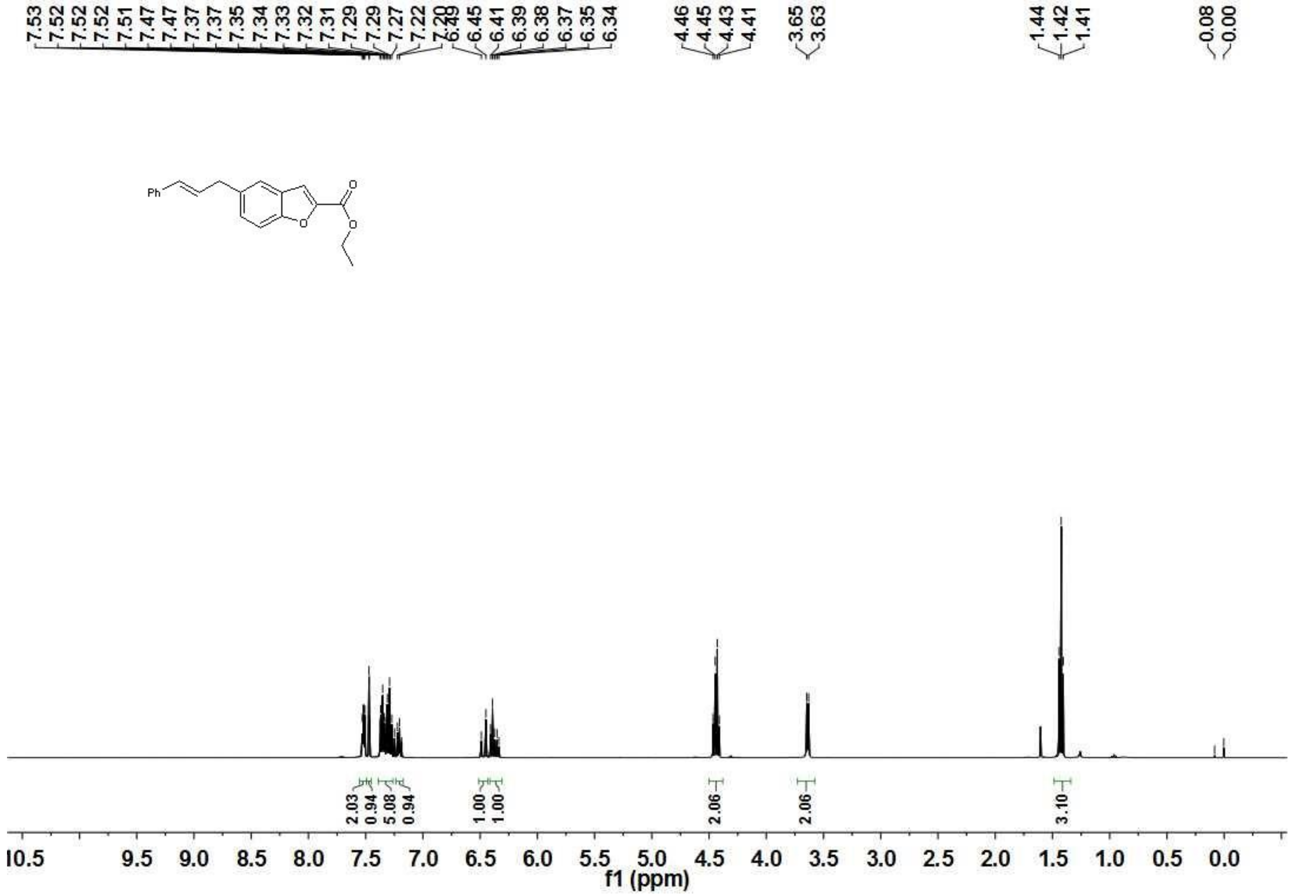

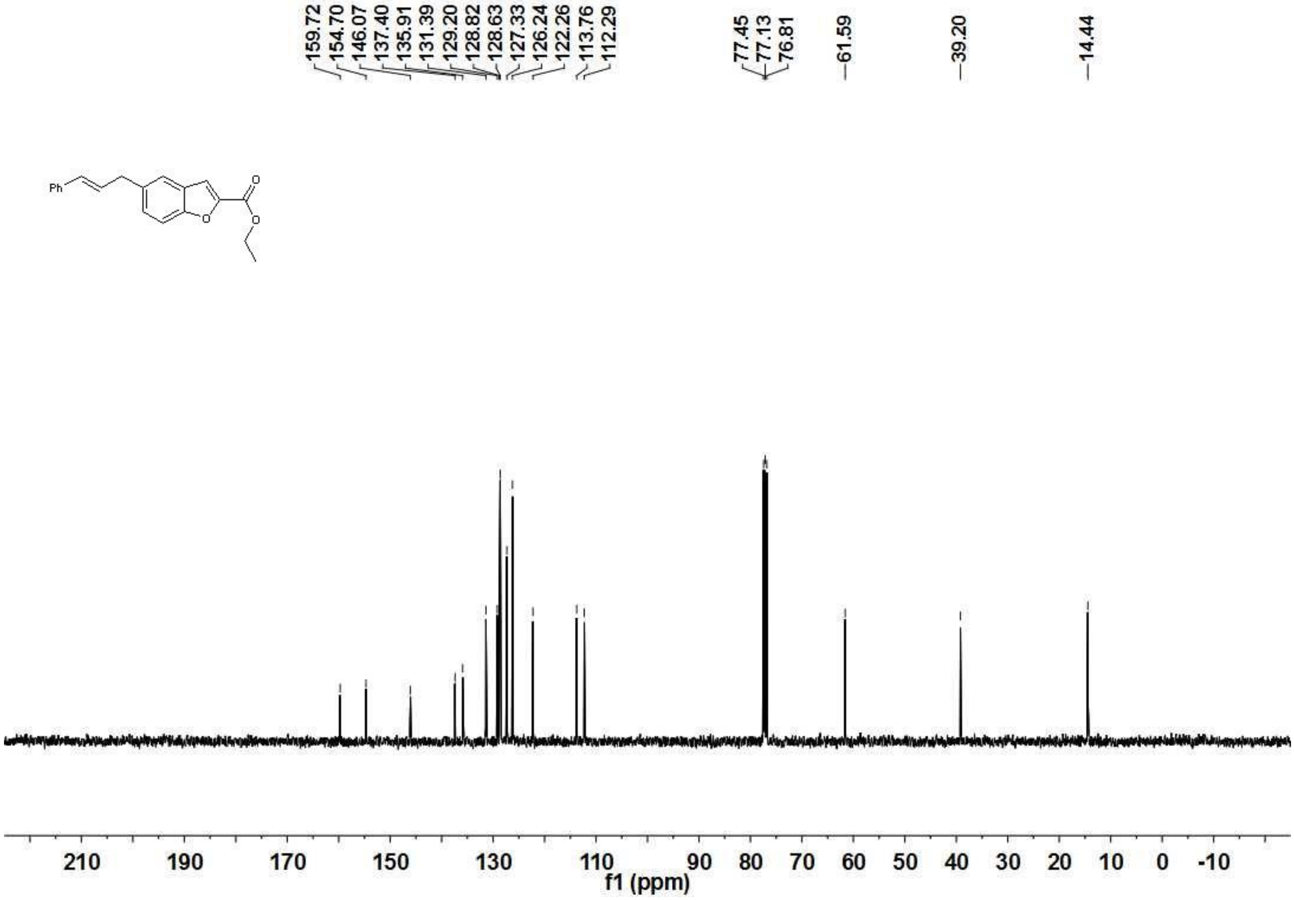

19

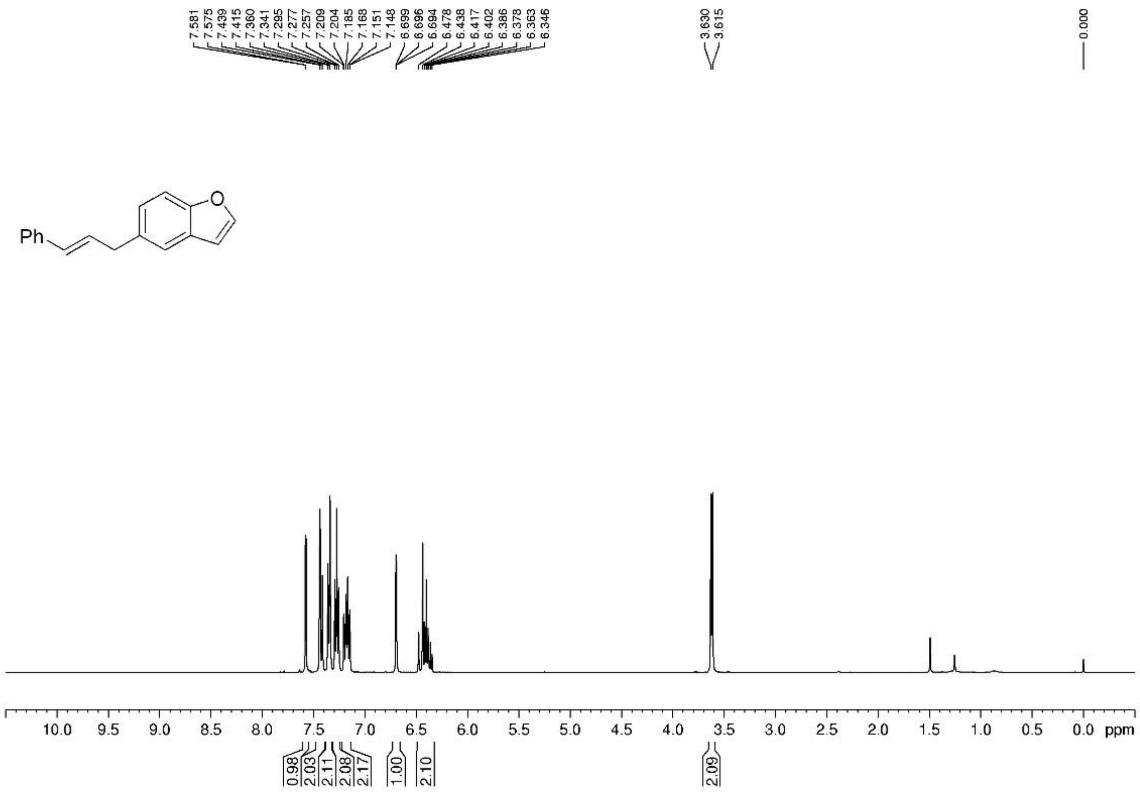

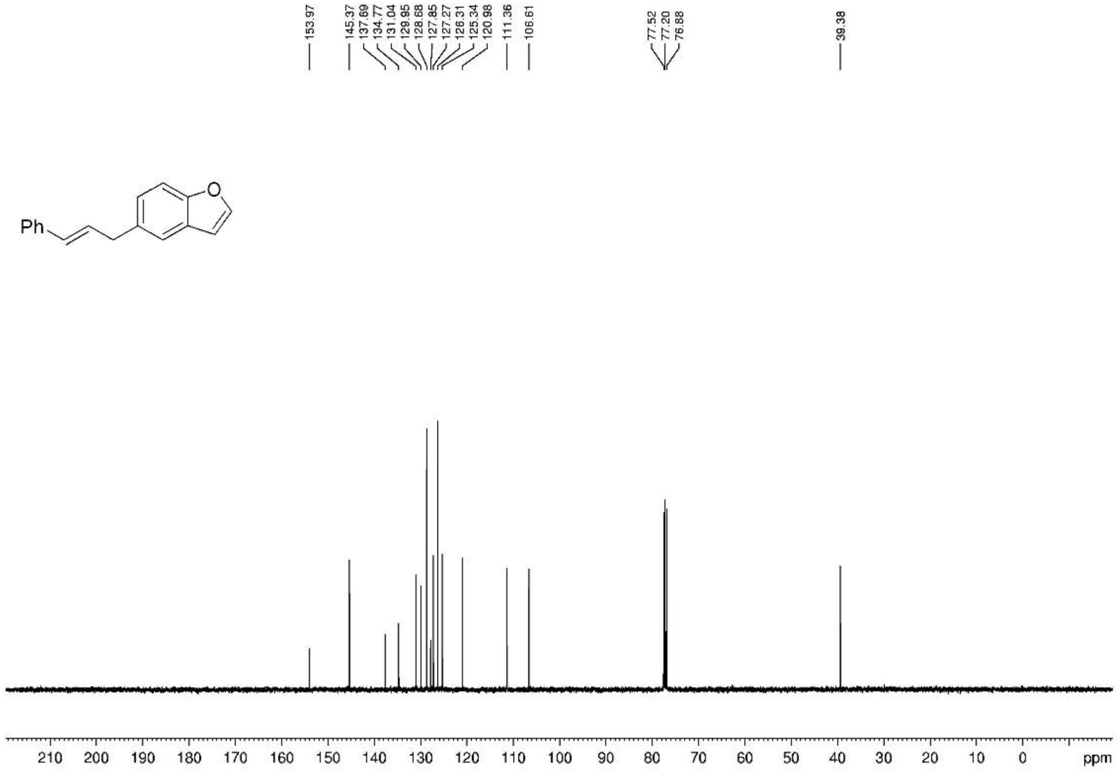

20

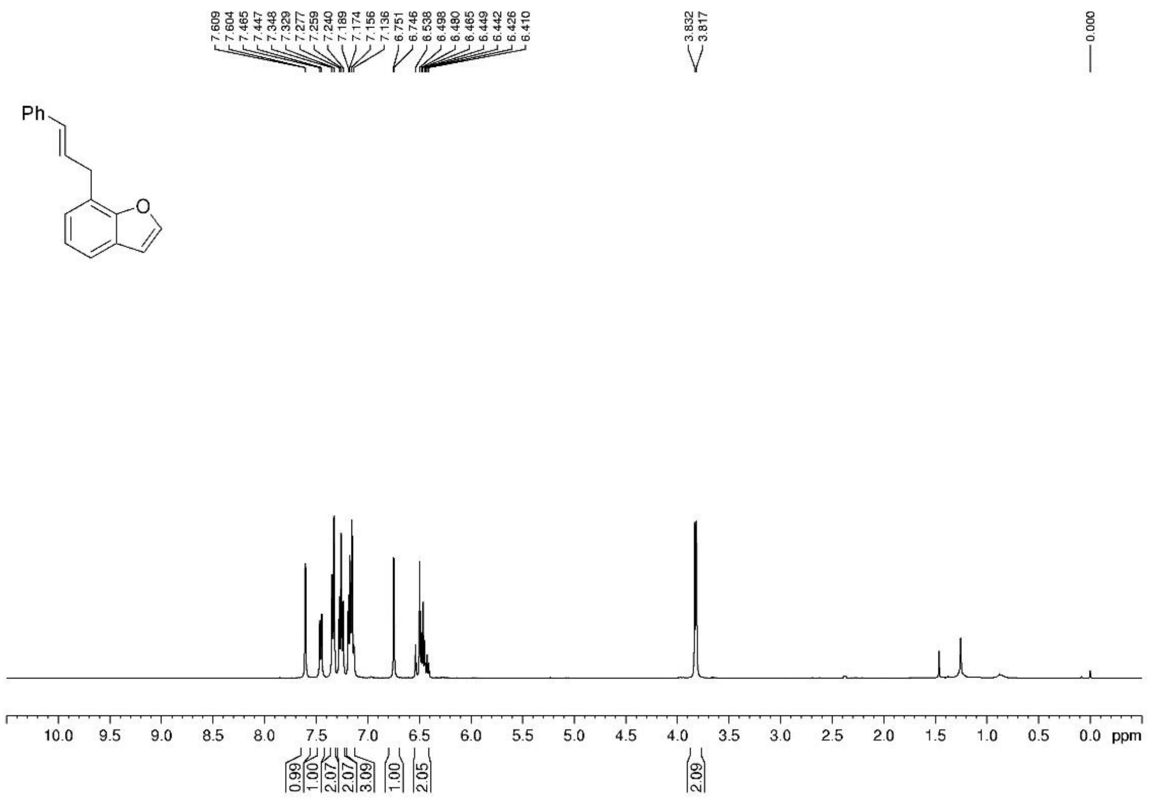

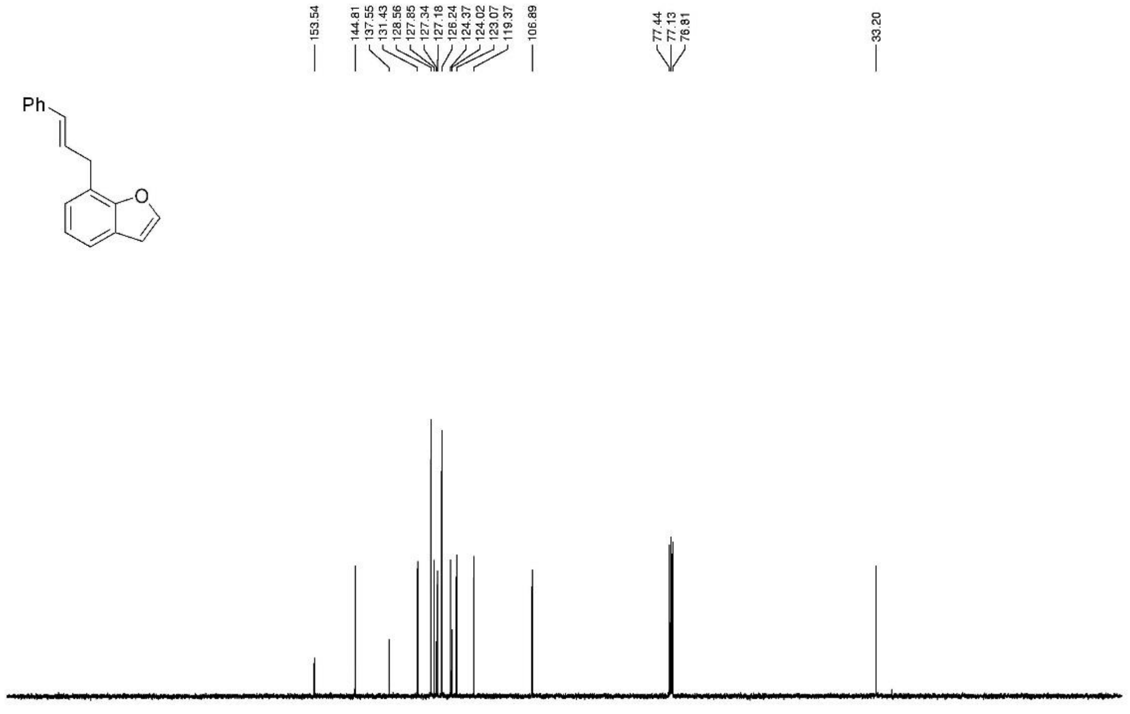

21

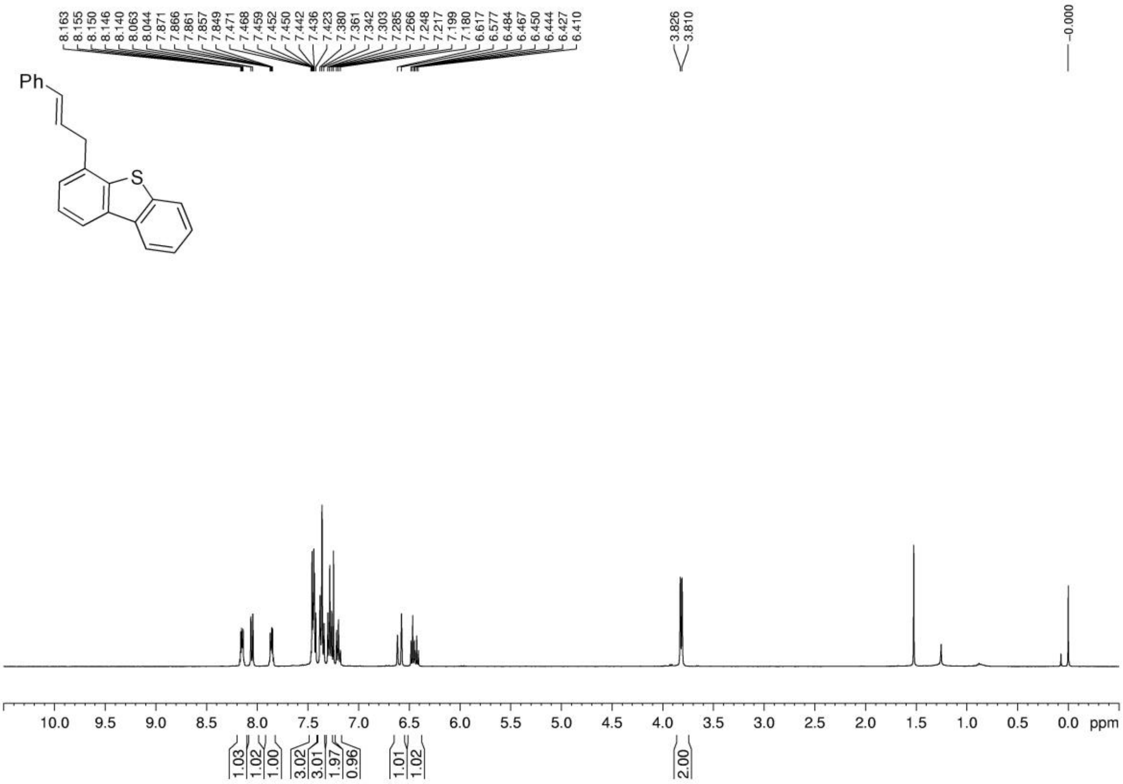

8.163
8.155
8.150
8.146
8.140
8.063
8.044
7.871
7.866
7.861
7.857
7.849
7.471
7.468
7.459
7.452
7.450
7.442
7.436
7.423
7.380
7.361
7.342
7.303
7.285
7.266
7.248
7.217
7.199
7.180
6.617
6.577
6.484
6.467
6.450
6.444
6.427
6.410
3.826
3.810
-0.000
Ph
S
10.0 9.5 9.0 8.5 8.0 7.5 7.0 6.5 6.0 5.5 5.0 4.5 4.0 3.5 3.0 2.5 2.0 1.5 1.0 0.5 0.0 ppm
1.03
1.02
1.00
3.02
3.01
1.97
0.96
1.01
1.02
2.00

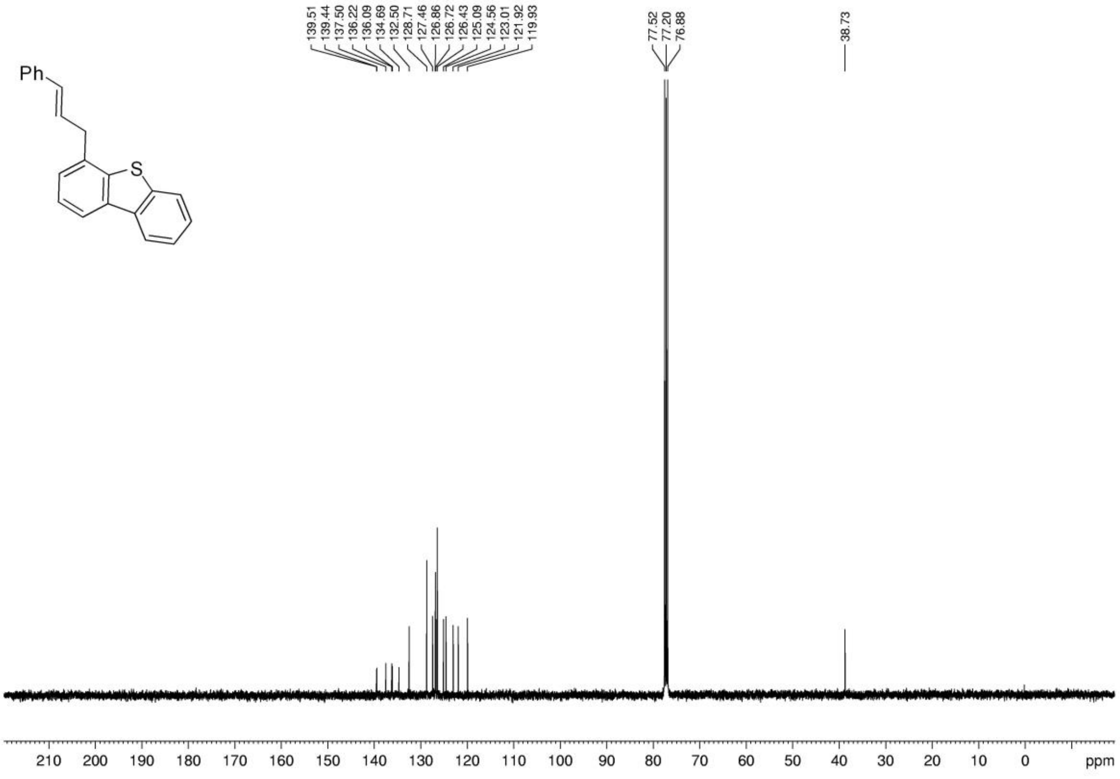

139.51
139.44
137.50
136.22
136.09
134.69
132.50
128.71
127.46
126.86
126.72
126.43
125.09
124.56
123.01
121.92
119.93
77.52
77.20
76.88
38.73
Ph
S
210 200 190 180 170 160 150 140 130 120 110 100 90 80 70 60 50 40 30 20 10 0 ppm

22

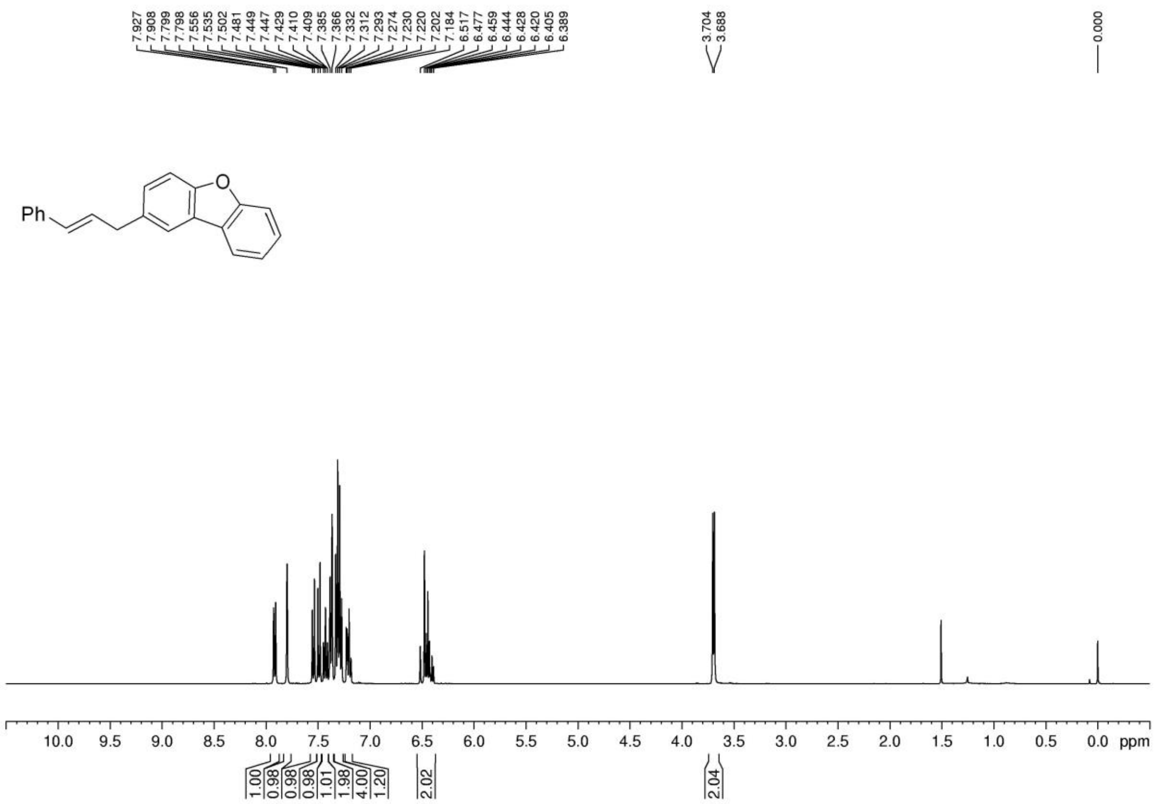

7.927
7.908
7.799
7.798
7.556
7.535
7.502
7.481
7.449
7.447
7.429
7.410
7.409
7.385
7.366
7.332
7.312
7.293
7.274
7.230
7.220
7.202
7.184
6.517
6.477
6.459
6.444
6.428
6.420
6.405
6.389
3.704
3.688
0.000
Ph
O
10.0 9.5 9.0 8.5 8.0 7.5 7.0 6.5 6.0 5.5 5.0 4.5 4.0 3.5 3.0 2.5 2.0 1.5 1.0 0.5 0.0 ppm
1.00
0.98
0.98
0.98
1.01
1.98
4.00
1.20
2.02
2.04

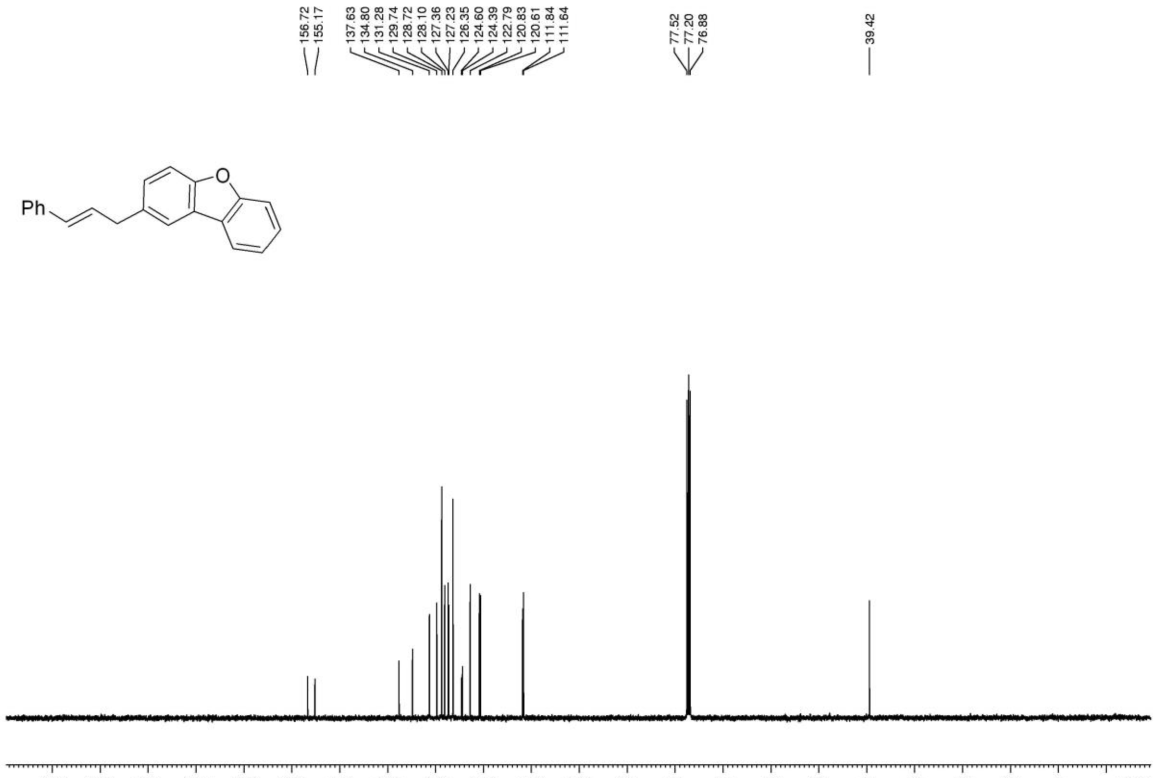

156.72
155.17
137.63
134.80
131.28
129.74
128.72
128.10
127.36
127.23
126.35
124.60
124.39
122.79
120.83
120.61
111.84
111.64
77.52
77.20
76.88
39.42
Ph
O
210 200 190 180 170 160 150 140 130 120 110 100 90 80 70 60 50 40 30 20 10 0 ppm

23

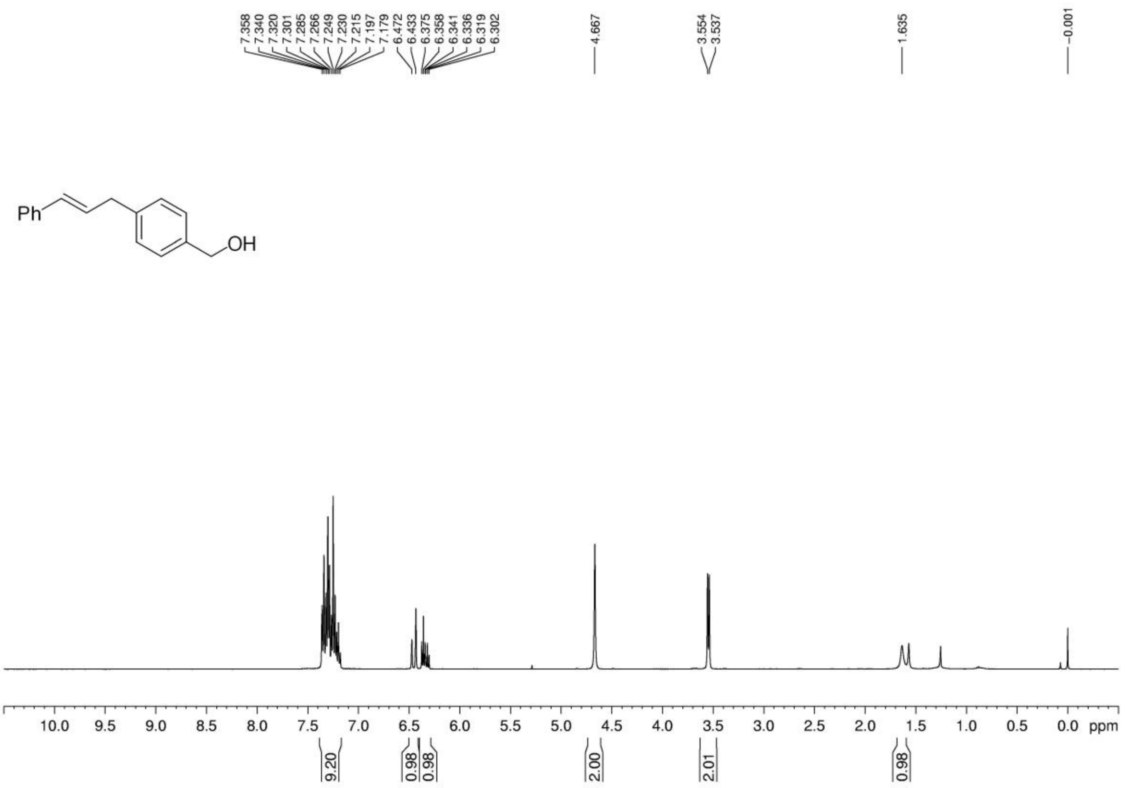

Ph
OH
7.358 7.340 7.320 7.301 7.285 7.266 7.249 7.230 7.215 7.197 7.179 6.472 6.433 6.375 6.358 6.341 6.336 6.319 6.302
4.667
3.554 3.537
1.635
-0.001
10.0 9.5 9.0 8.5 8.0 7.5 7.0 6.5 6.0 5.5 5.0 4.5 4.0 3.5 3.0 2.5 2.0 1.5 1.0 0.5 0.0 ppm
9.20 0.98 0.98 2.00 2.01 0.98

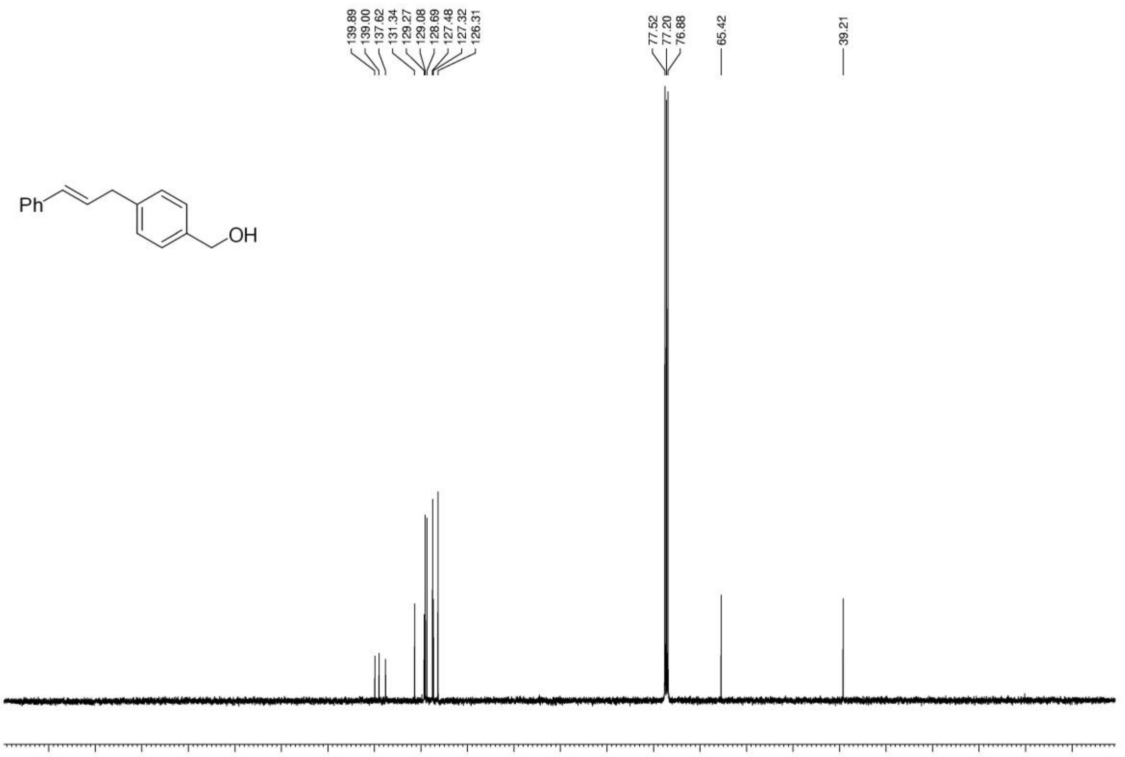

Ph
OH
139.89 139.00 137.62 131.34 129.27 129.08 128.69 127.48 127.32 126.31
77.52 77.20 76.88
65.42
39.21
210 200 190 180 170 160 150 140 130 120 110 100 90 80 70 60 50 40 30 20 10 0 ppm

24

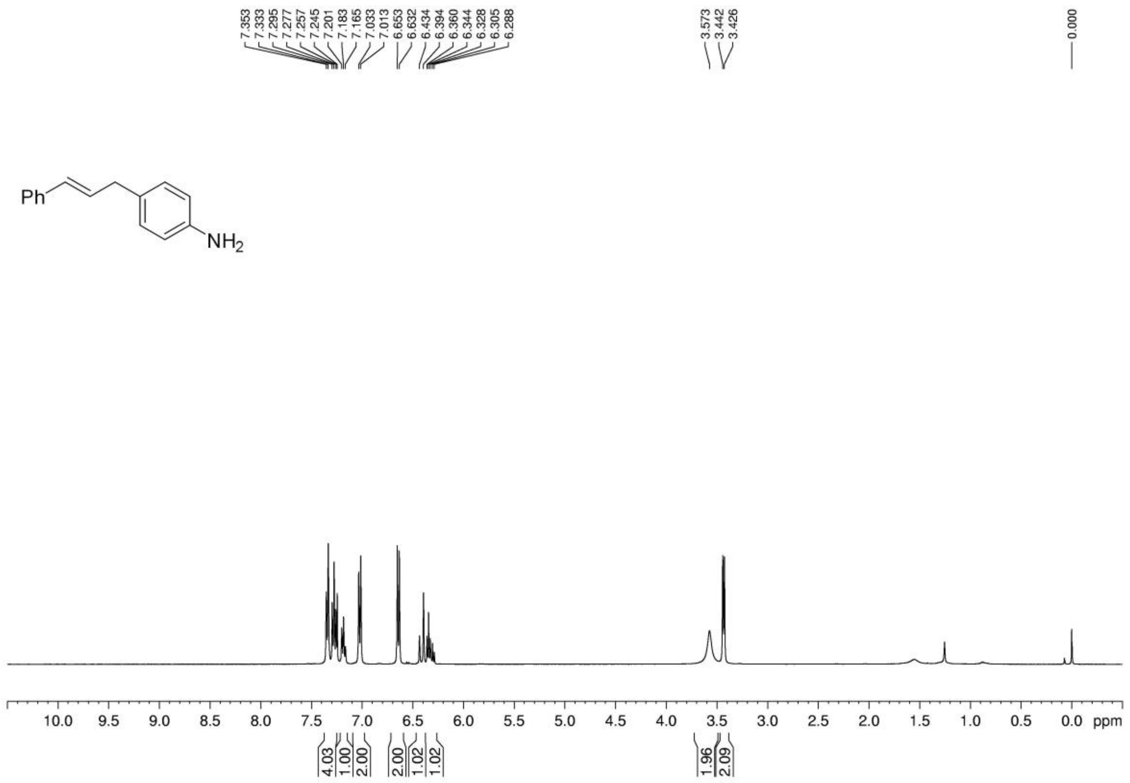
7.353 7.333 7.295 7.277 7.257 7.245 7.201 7.183 7.165 7.033 7.013 6.653 6.632 6.434 6.394 6.360 6.344 6.328 6.305 6.288
3.573 3.442 3.426
0.000
Ph
NH2
10.0 9.5 9.0 8.5 8.0 7.5 7.0 6.5 6.0 5.5 5.0 4.5 4.0 3.5 3.0 2.5 2.0 1.5 1.0 0.5 0.0 ppm
4.03 1.00 2.00 2.00 1.02 1.02
1.96 2.09

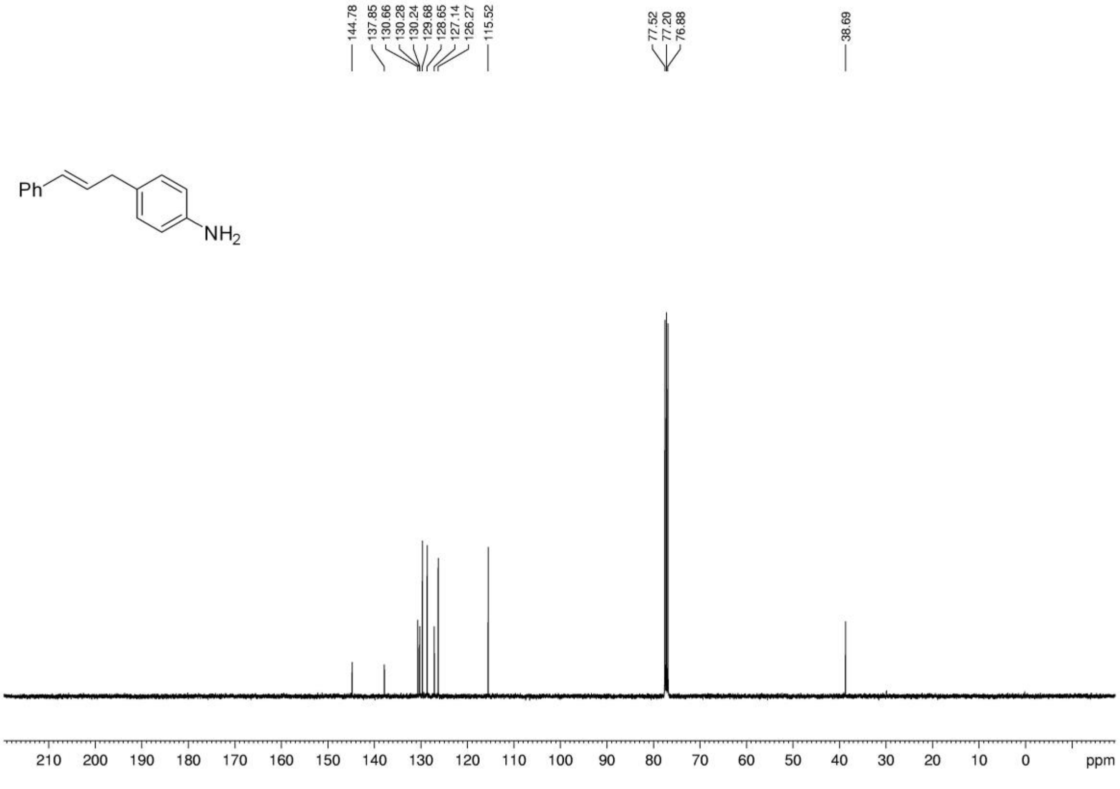
144.78 137.85 130.66 130.28 130.24 129.68 128.65 127.14 126.27 115.52
77.52 77.20 76.88
38.69
Ph
NH2
210 200 190 180 170 160 150 140 130 120 110 100 90 80 70 60 50 40 30 20 10 0 ppm

25

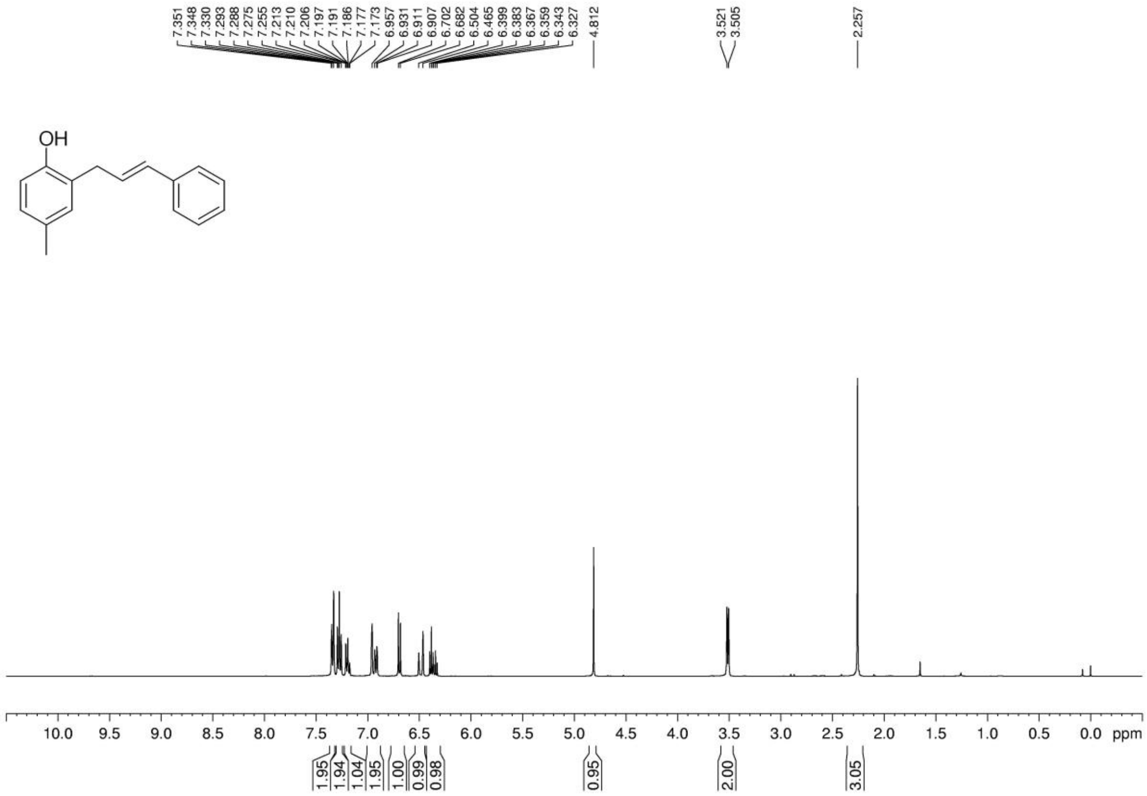
7.351 7.348 7.330 7.293 7.288 7.275 7.255 7.213 7.210 7.206 7.197 7.191 7.186 7.177 7.173 6.957 6.931 6.911 6.907 6.702 6.682 6.504 6.465 6.399 6.383 6.367 6.359 6.343 6.327
4.812
3.521 3.505
2.257
OH
10.0 9.5 9.0 8.5 8.0 7.5 7.0 6.5 6.0 5.5 5.0 4.5 4.0 3.5 3.0 2.5 2.0 1.5 1.0 0.5 0.0 ppm
1.95 1.94 1.04 1.95 1.00 0.99 0.98 0.95 2.00 3.05

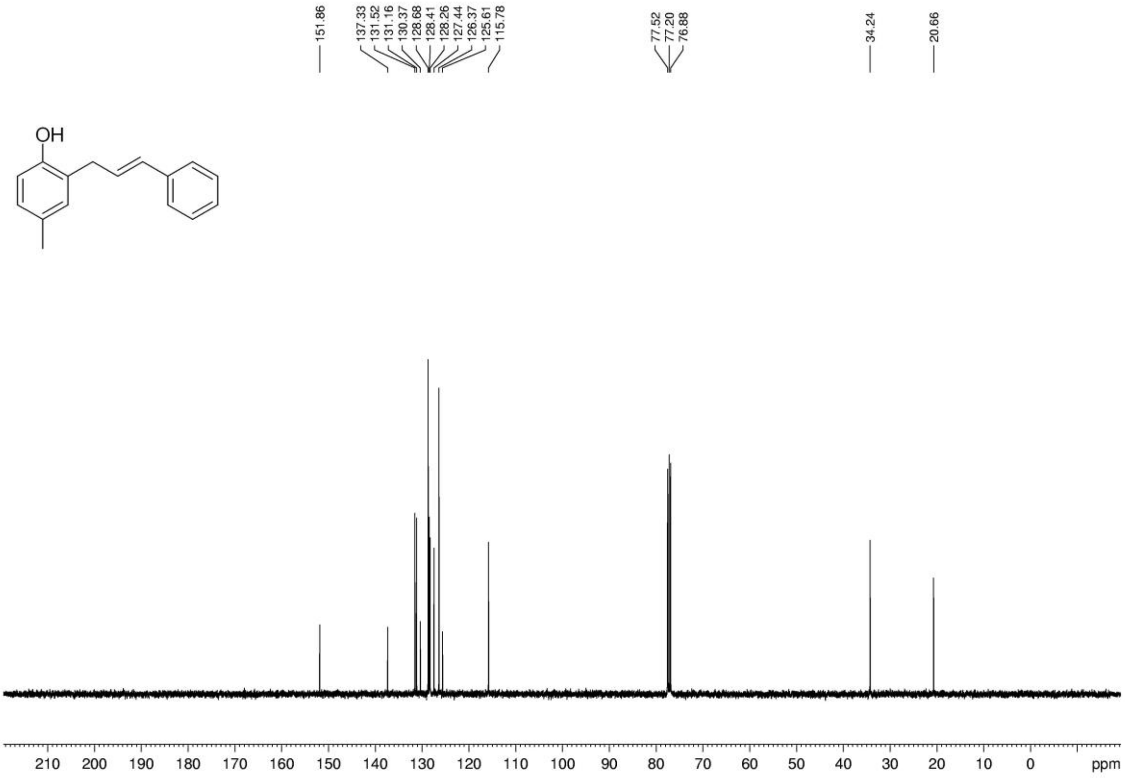
151.86
137.33 131.52 131.16 130.37 128.68 128.41 128.26 127.44 126.37 125.61 115.78
77.52 77.20 76.88
34.24
20.66
OH
210 200 190 180 170 160 150 140 130 120 110 100 90 80 70 60 50 40 30 20 10 0 ppm

26

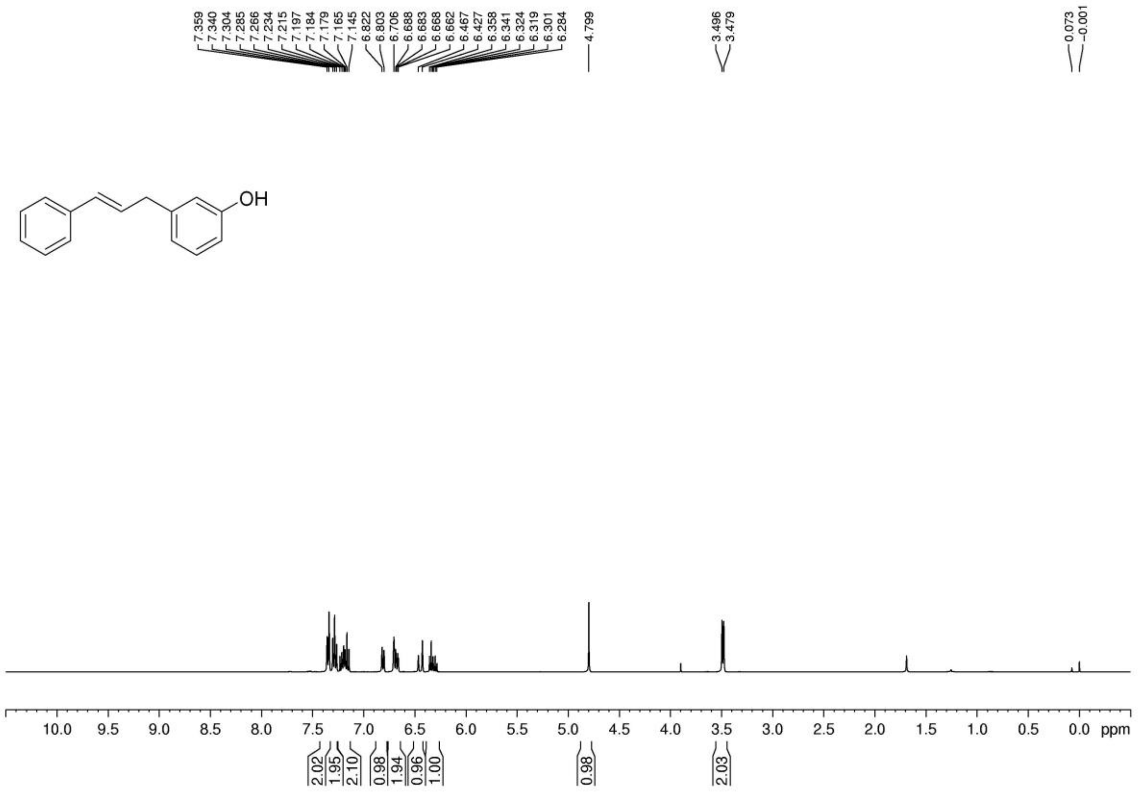
7.359
7.340
7.304
7.285
7.266
7.234
7.215
7.197
7.184
7.179
7.165
7.145
6.822
6.803
6.706
6.688
6.683
6.668
6.662
6.467
6.427
6.358
6.341
6.324
6.319
6.301
6.284
4.799
3.496
3.479
0.073
-0.001
OH
10.0 9.5 9.0 8.5 8.0 7.5 7.0 6.5 6.0 5.5 5.0 4.5 4.0 3.5 3.0 2.5 2.0 1.5 1.0 0.5 0.0 ppm
2.02
1.95
2.10
0.98
1.94
0.96
1.00
0.98
2.03

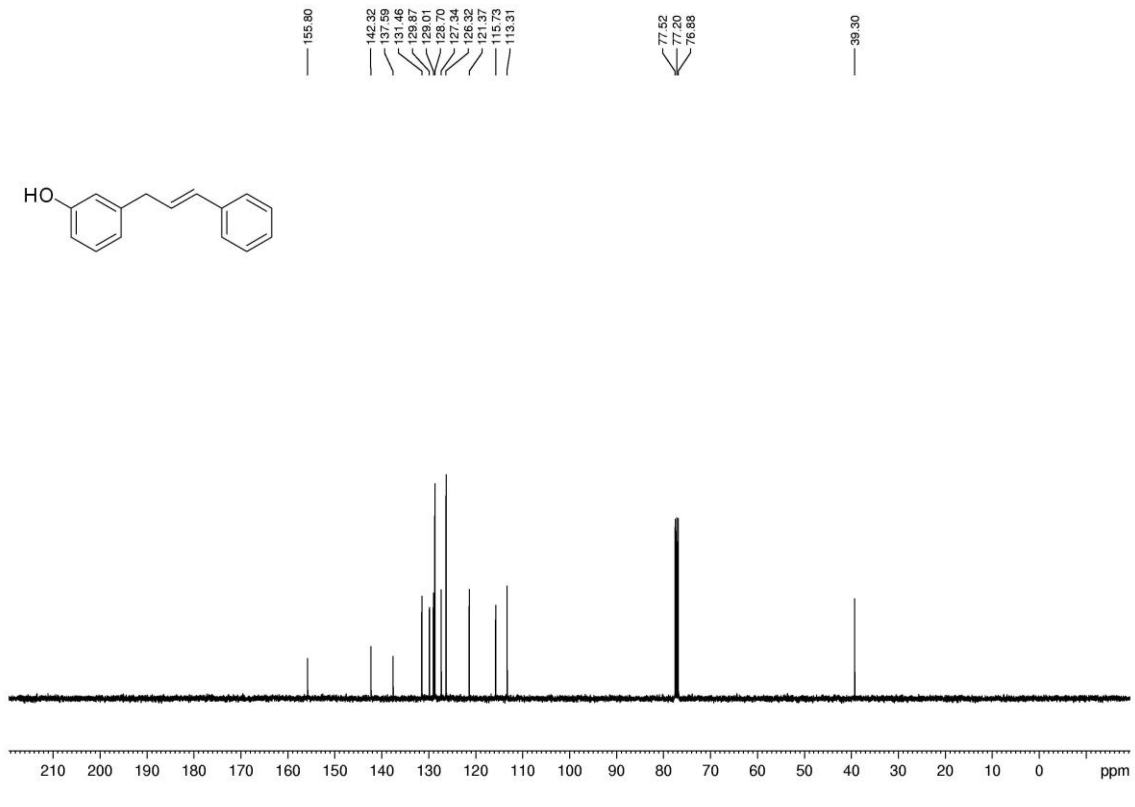
155.80
142.32
137.59
131.46
129.87
129.01
128.70
127.34
126.32
121.37
115.73
113.31
77.52
77.20
76.88
39.30
HO
210 200 190 180 170 160 150 140 130 120 110 100 90 80 70 60 50 40 30 20 10 0 ppm

27

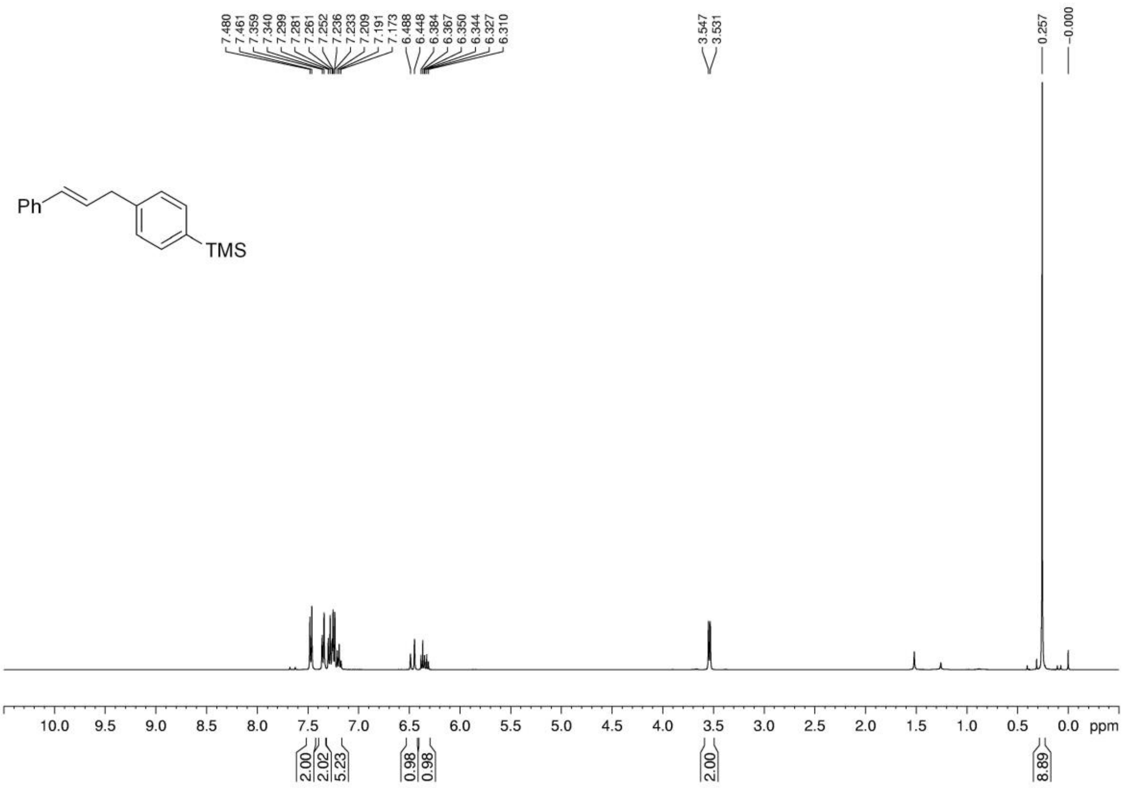

7.480 7.461 7.359 7.340 7.299 7.281 7.261 7.252 7.236 7.233 7.209 7.191 7.173 6.488 6.448 6.384 6.367 6.350 6.344 6.327 6.310
3.547 3.531
0.257
−0.000
Ph
TMS
10.0 9.5 9.0 8.5 8.0 7.5 7.0 6.5 6.0 5.5 5.0 4.5 4.0 3.5 3.0 2.5 2.0 1.5 1.0 0.5 0.0 ppm
2.00 2.02 5.23 0.98 0.98 2.00 8.89

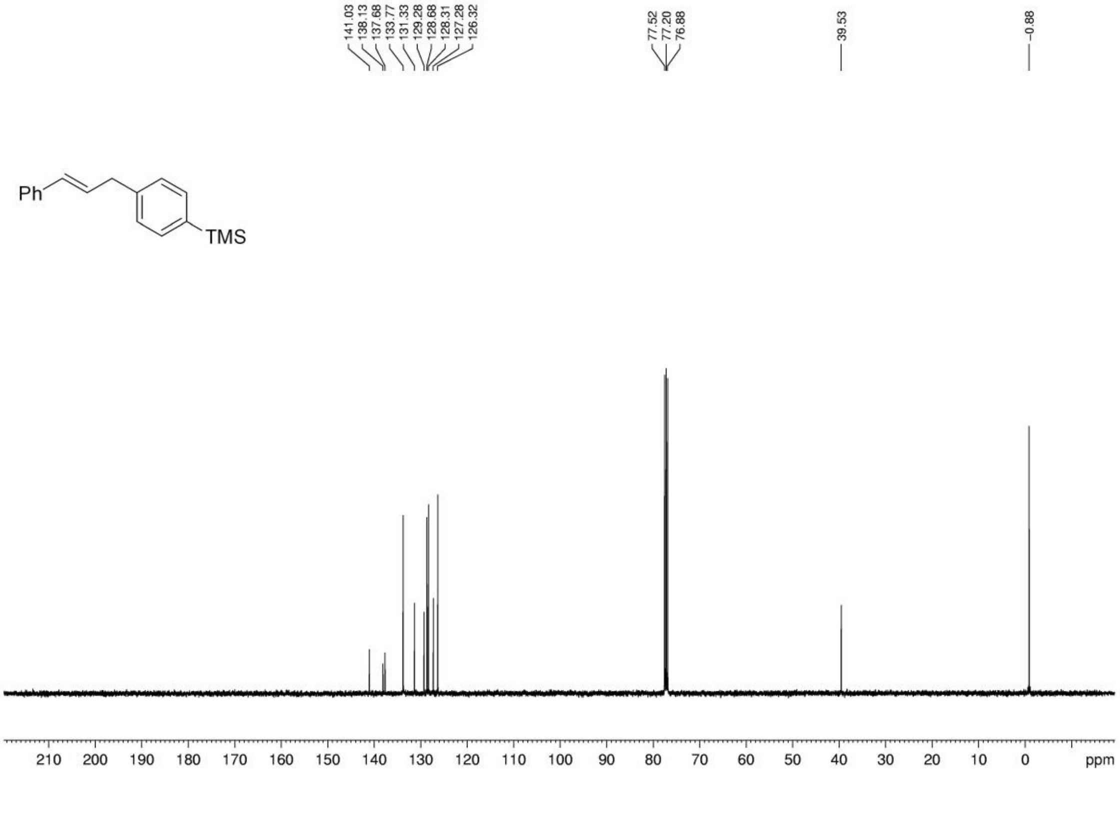

141.03 138.13 137.68 133.77 131.33 129.28 128.68 128.31 127.28 126.32
77.52 77.20 76.88
39.53
−0.88
Ph
TMS
210 200 190 180 170 160 150 140 130 120 110 100 90 80 70 60 50 40 30 20 10 0 ppm

28

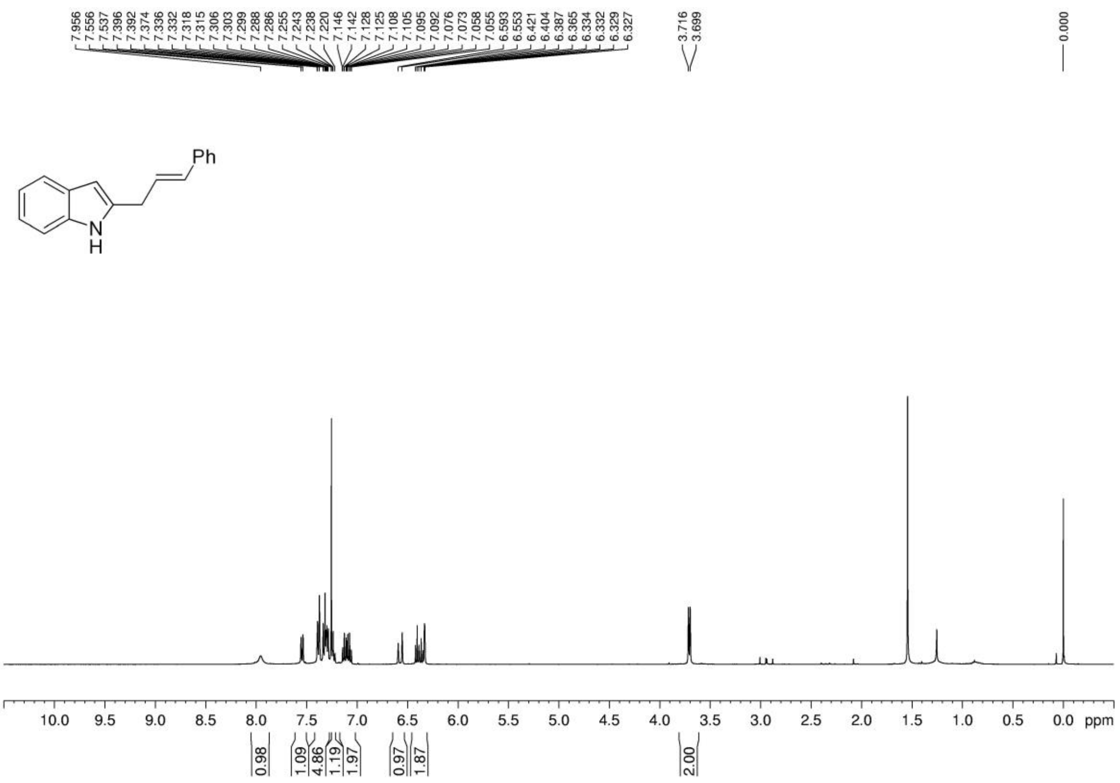
7.956
7.556
7.537
7.396
7.392
7.374
7.336
7.332
7.318
7.315
7.306
7.303
7.299
7.288
7.286
7.255
7.243
7.238
7.220
7.146
7.142
7.128
7.125
7.108
7.105
7.095
7.092
7.076
7.073
7.058
7.055
6.593
6.553
6.421
6.404
6.387
6.365
6.334
6.332
6.329
6.327
3.716
3.699
0.000
Ph
N
H
10.0 9.5 9.0 8.5 8.0 7.5 7.0 6.5 6.0 5.5 5.0 4.5 4.0 3.5 3.0 2.5 2.0 1.5 1.0 0.5 0.0 ppm
0.98
1.09
4.86
1.19
1.97
0.97
1.87
2.00

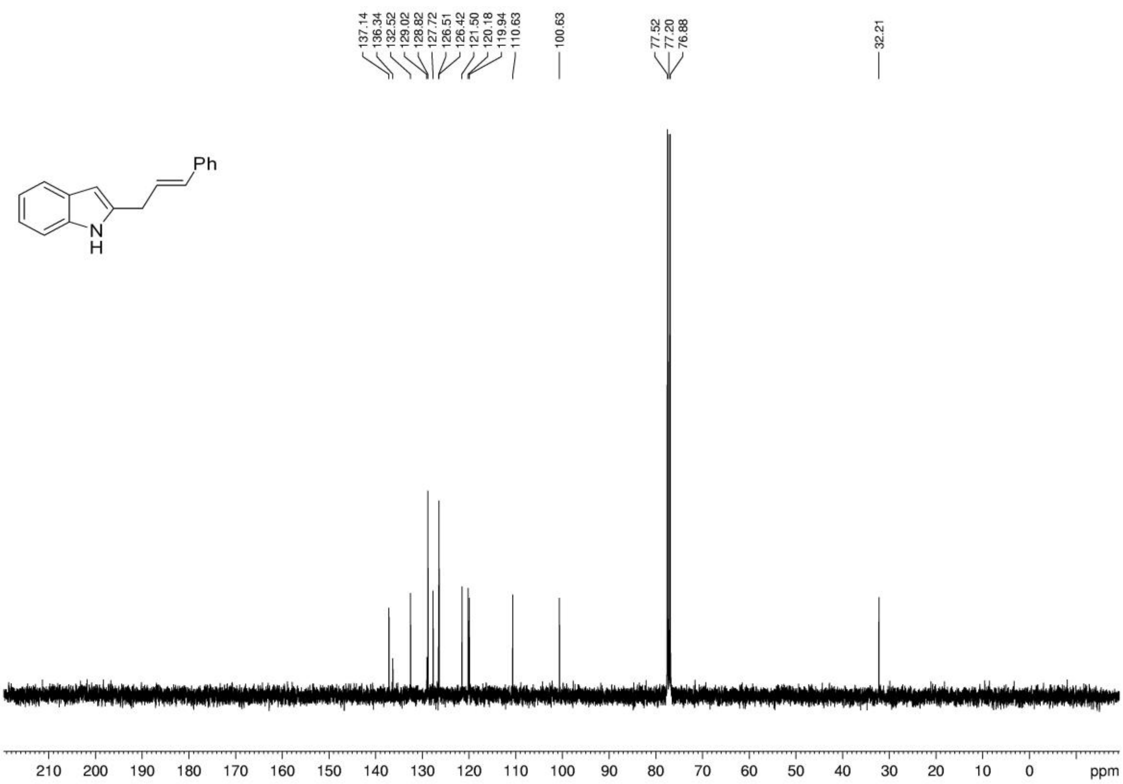
137.14
136.34
132.52
129.02
128.82
127.72
126.51
126.42
121.50
120.18
119.94
110.63
100.63
77.52
77.20
76.88
32.21
Ph
N
H
210 200 190 180 170 160 150 140 130 120 110 100 90 80 70 60 50 40 30 20 10 0 ppm

29

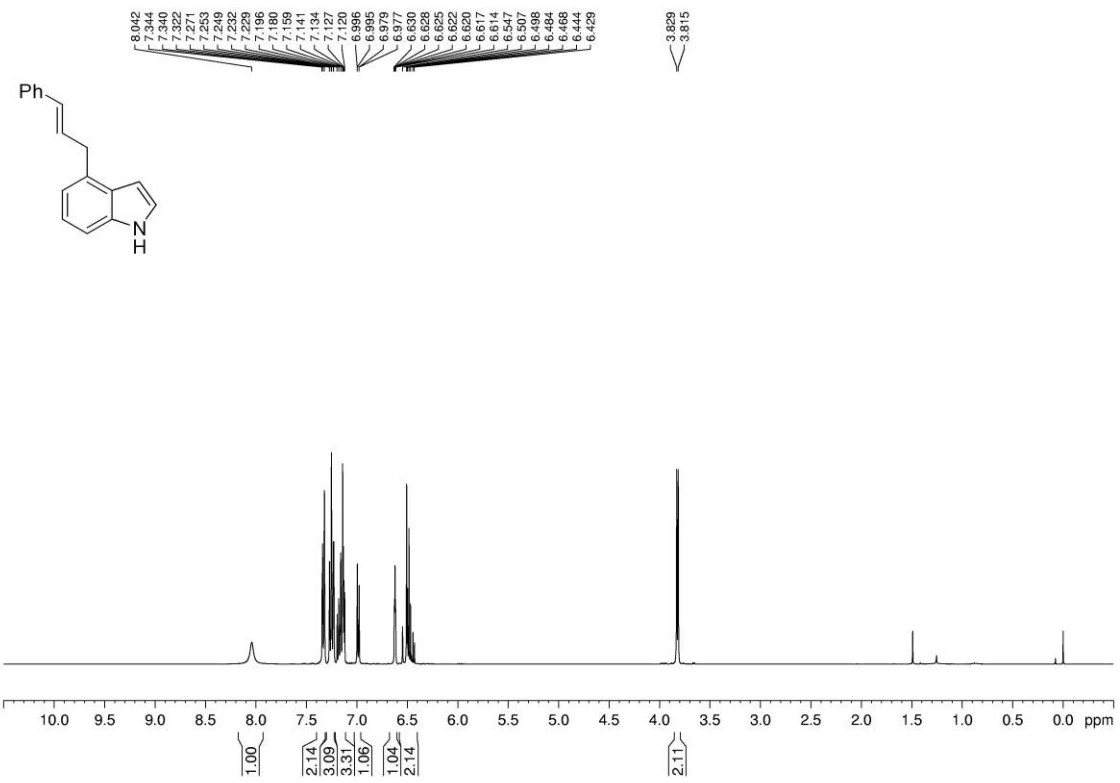
8.042
7.344
7.340
7.322
7.271
7.253
7.249
7.232
7.229
7.196
7.180
7.159
7.141
7.134
7.127
7.120
6.996
6.995
6.979
6.977
6.630
6.628
6.625
6.622
6.620
6.617
6.614
6.547
6.507
6.498
6.484
6.468
6.444
6.429
3.829
3.815
Ph
N
H
1.00
2.14
3.09
3.31
1.06
1.04
2.14
2.11
ppm

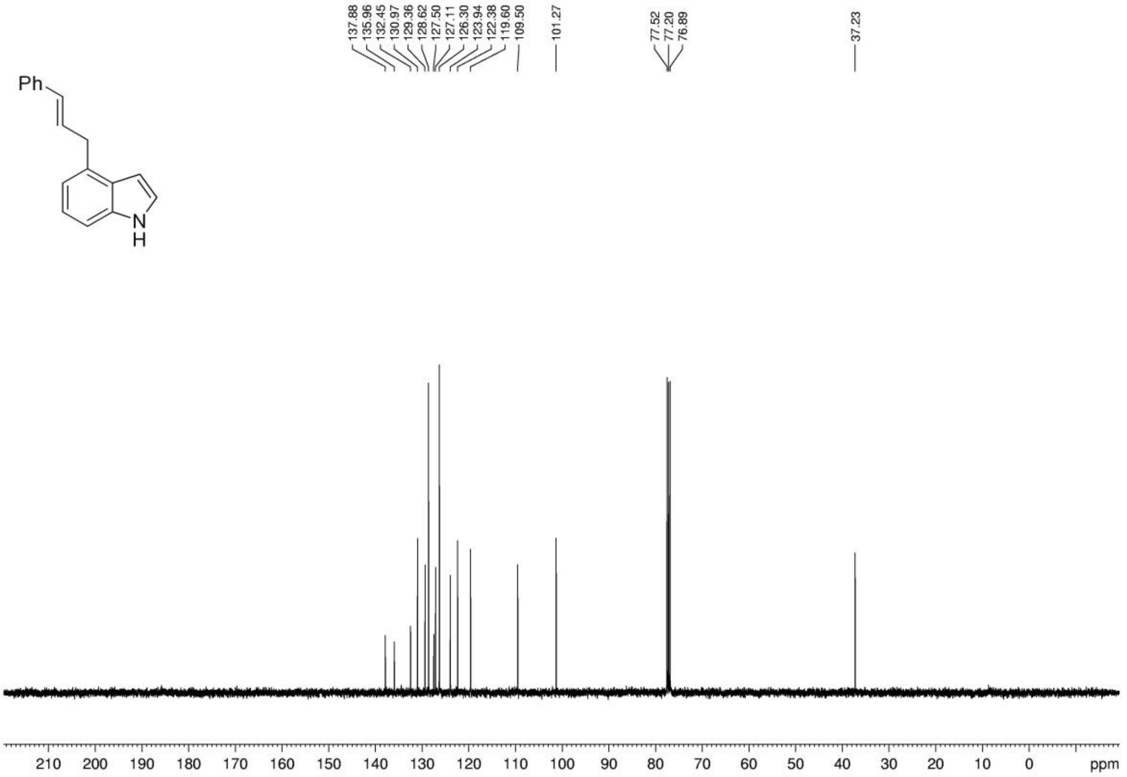
137.88
135.96
132.45
130.97
129.36
128.62
127.50
127.11
126.30
123.94
122.38
119.60
109.50
101.27
77.52
77.20
76.89
37.23
Ph
N
H
ppm

30

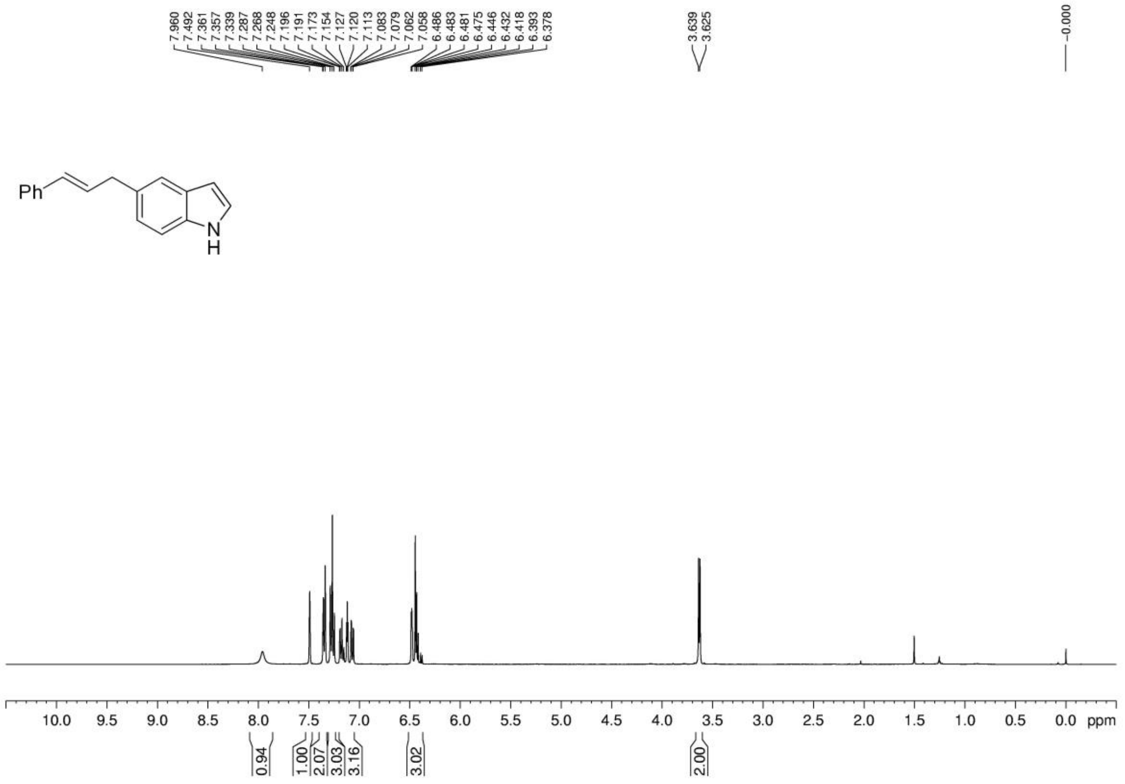
7.960
7.492
7.361
7.357
7.339
7.287
7.268
7.248
7.196
7.191
7.173
7.154
7.127
7.120
7.113
7.083
7.079
7.062
7.058
6.486
6.483
6.481
6.475
6.446
6.432
6.418
6.393
6.378
3.639
3.625
-0.000
Ph
N
H
10.0
9.5
9.0
8.5
8.0
7.5
7.0
6.5
6.0
5.5
5.0
4.5
4.0
3.5
3.0
2.5
2.0
1.5
1.0
0.5
0.0
ppm
0.94
1.00
2.07
3.03
3.16
3.02
2.00

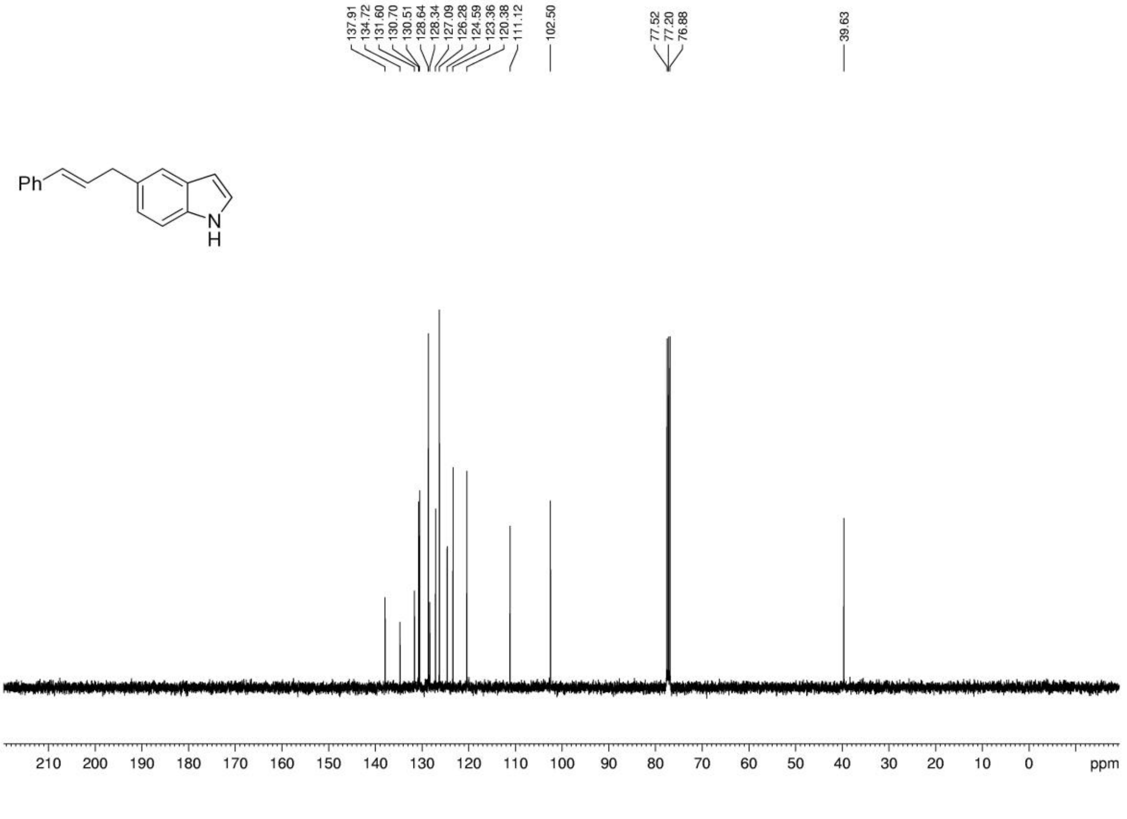
137.91
134.72
131.60
130.70
130.51
128.64
128.34
127.09
126.28
124.59
123.36
120.38
111.12
102.50
77.52
77.20
76.88
39.63
Ph
N
H
210
200
190
180
170
160
150
140
130
120
110
100
90
80
70
60
50
40
30
20
10
0
ppm

31

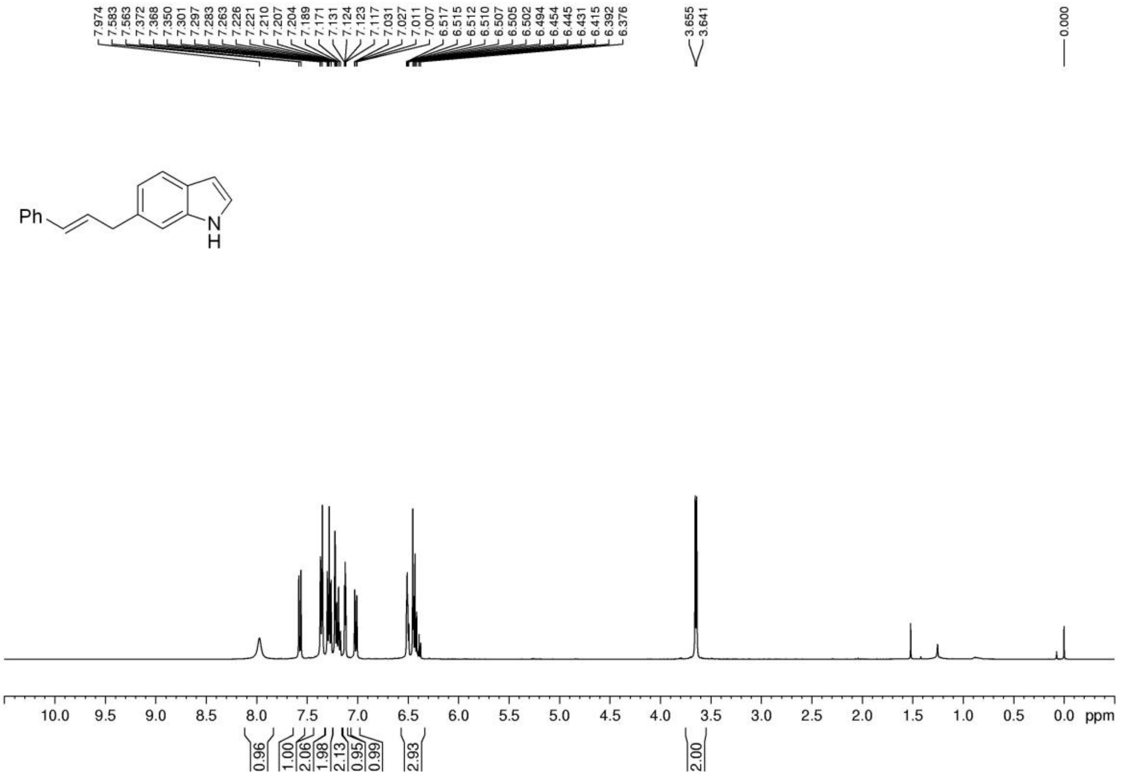
7.974
7.583
7.563
7.372
7.368
7.350
7.301
7.297
7.283
7.263
7.226
7.221
7.210
7.207
7.204
7.189
7.171
7.131
7.124
7.123
7.117
7.031
7.027
7.011
7.007
6.517
6.515
6.512
6.510
6.507
6.505
6.502
6.494
6.454
6.445
6.431
6.415
6.392
6.376
3.655
3.641
0.000
Ph
N
H
0.96
1.00
2.06
1.98
2.13
0.95
0.99
2.93
2.00
ppm

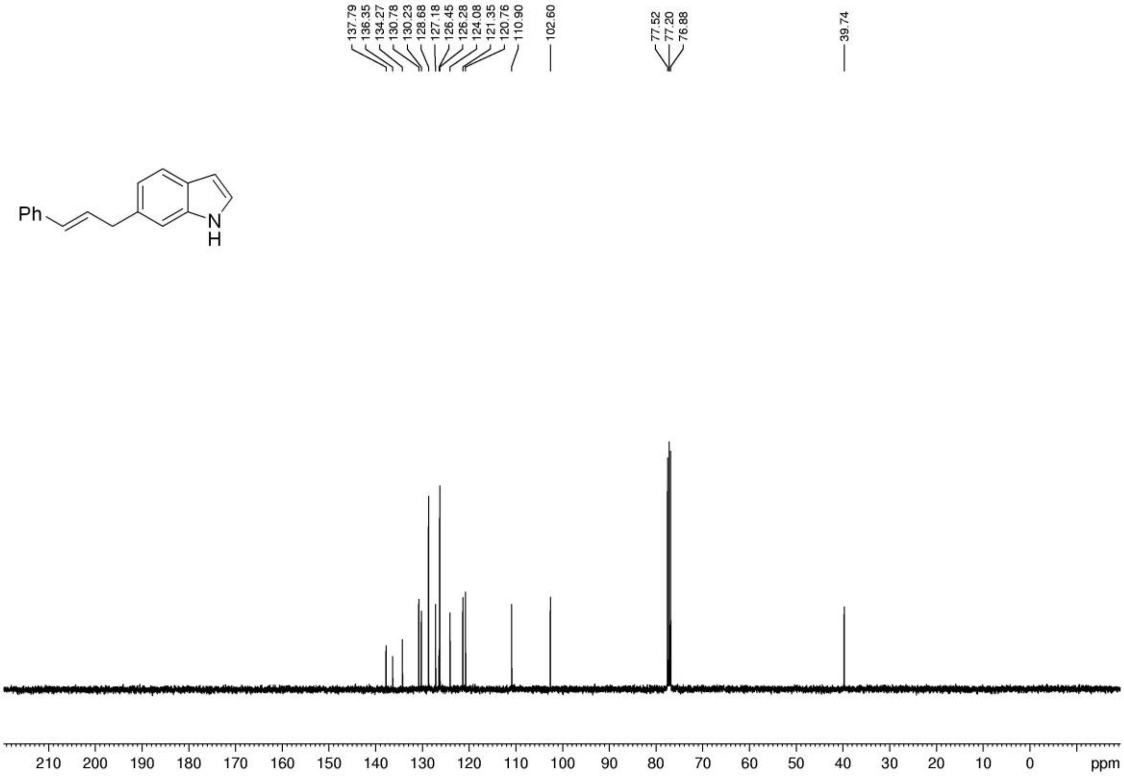
137.79
136.35
134.27
130.78
130.23
128.68
127.18
126.45
126.28
124.08
121.35
120.76
110.90
102.60
77.52
77.20
76.88
39.74
Ph
N
H
ppm

32

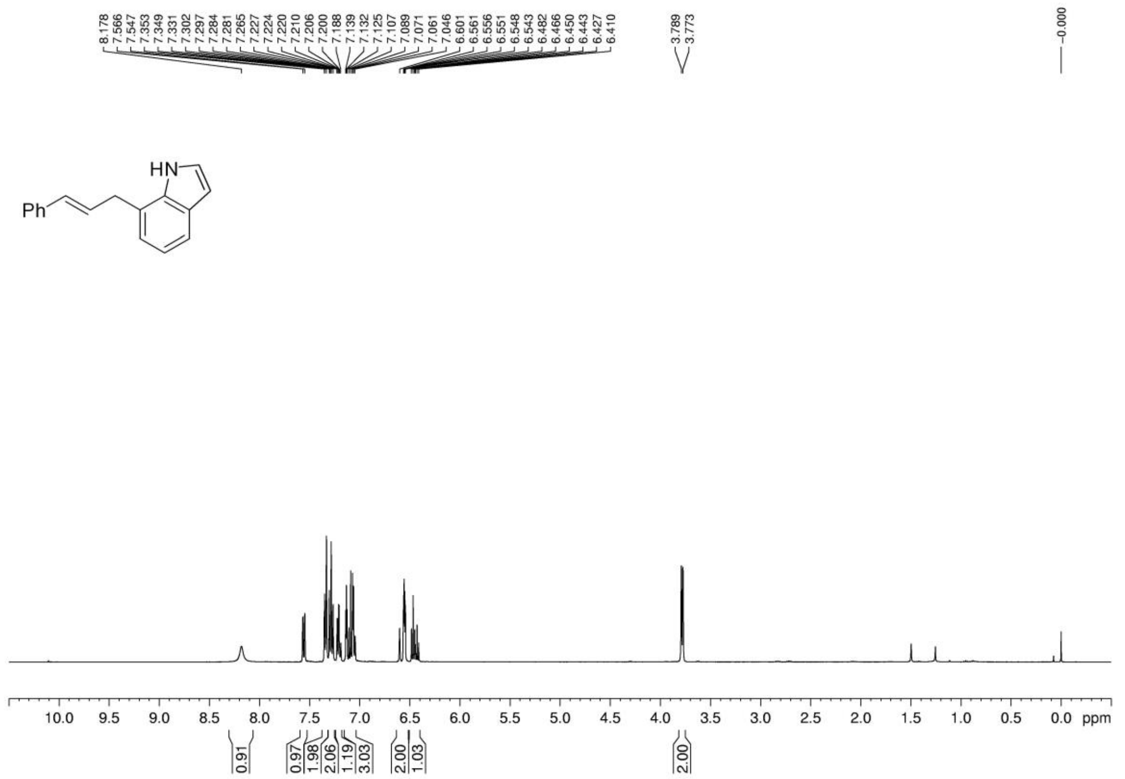

8.178
7.566
7.547
7.353
7.349
7.331
7.302
7.297
7.284
7.281
7.265
7.227
7.224
7.220
7.210
7.206
7.200
7.188
7.139
7.132
7.125
7.107
7.089
7.071
7.061
7.046
6.601
6.561
6.556
6.551
6.548
6.543
6.482
6.466
6.450
6.443
6.427
6.410
3.789
3.773
-0.000
HN
Ph
10.0 9.5 9.0 8.5 8.0 7.5 7.0 6.5 6.0 5.5 5.0 4.5 4.0 3.5 3.0 2.5 2.0 1.5 1.0 0.5 0.0 ppm
0.91
0.97
1.98
2.06
1.19
3.03
2.00
1.03
2.00

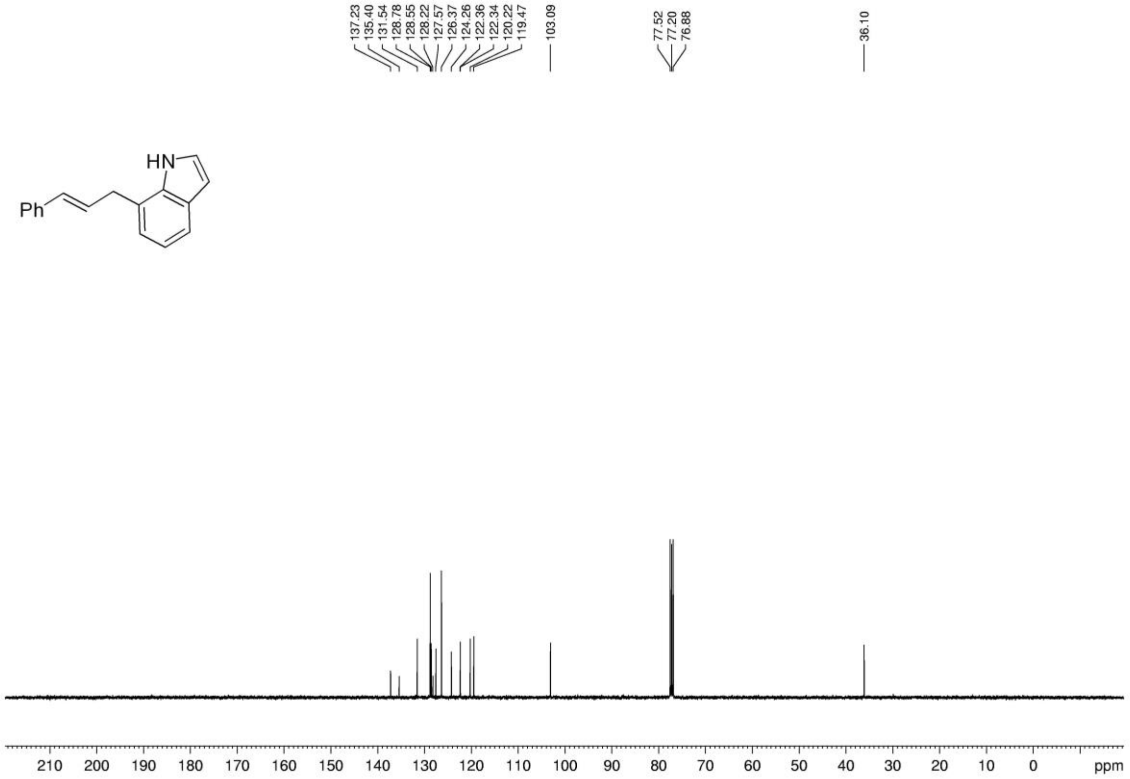

137.23
135.40
131.54
128.78
128.55
128.22
127.57
126.37
124.26
122.36
122.34
120.22
119.47
103.09
77.52
77.20
76.88
36.10
HN
Ph
210 200 190 180 170 160 150 140 130 120 110 100 90 80 70 60 50 40 30 20 10 0 ppm

33

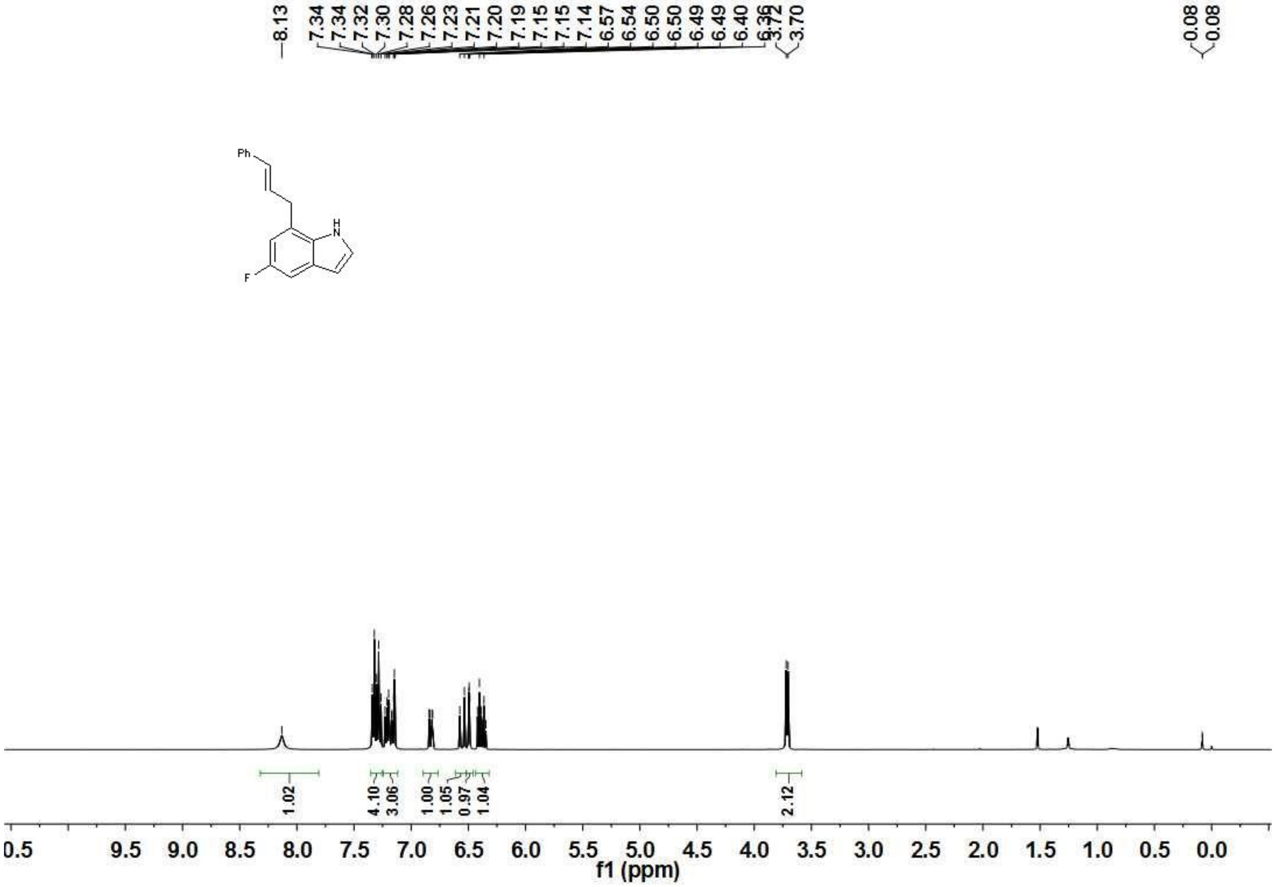
8.13
7.34
7.34
7.32
7.30
7.28
7.26
7.23
7.21
7.20
7.19
7.15
7.15
7.14
6.57
6.54
6.50
6.50
6.49
6.49
6.40
6.36
3.72
3.70
0.08
0.08
Ph
H
N
F
1.02
4.10
3.06
1.00
1.05
0.97
1.04
2.12
f1 (ppm)

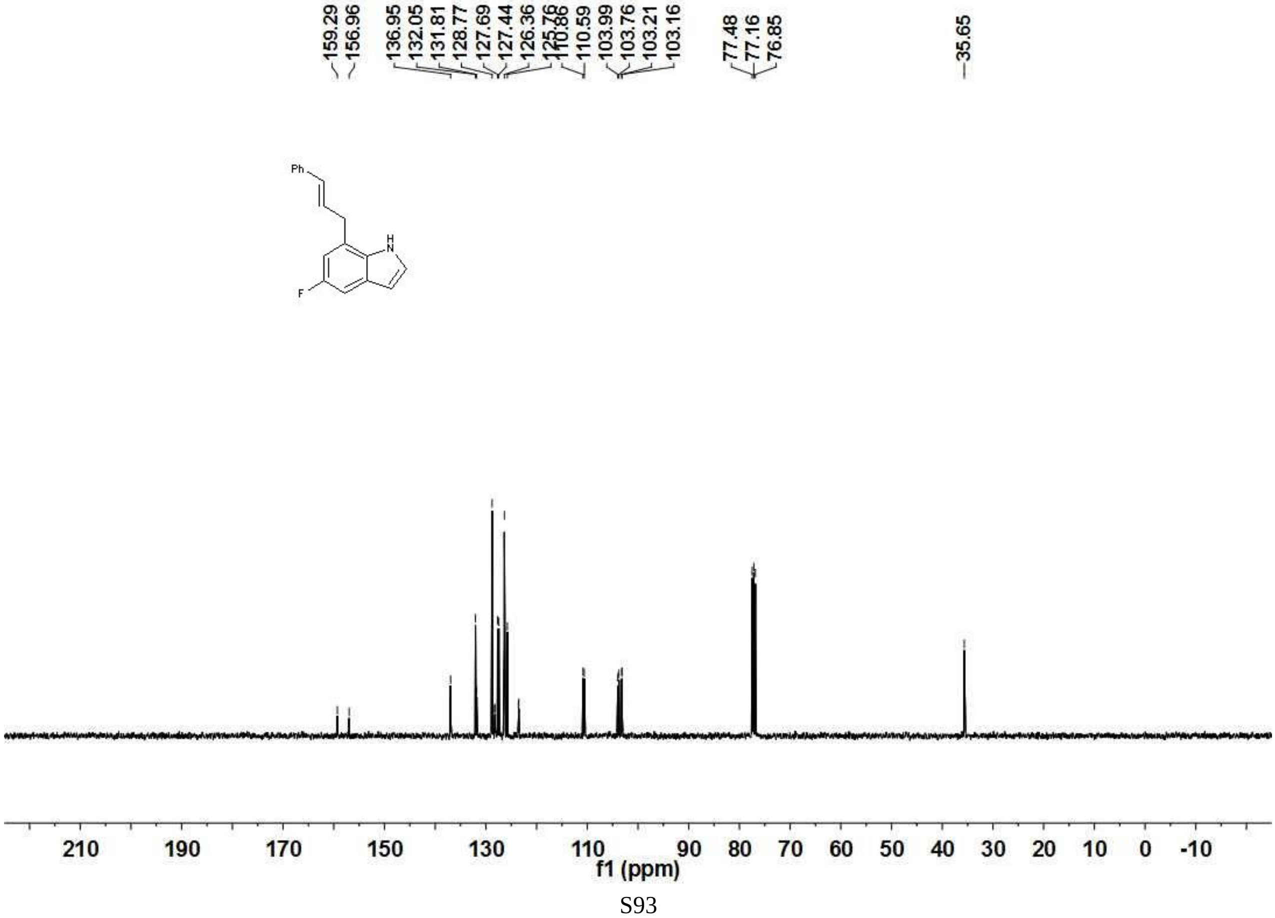
159.29
156.96
136.95
132.05
131.81
128.77
127.69
127.44
126.36
125.76
110.86
110.59
103.99
103.76
103.21
103.16
77.48
77.16
76.85
35.65
Ph
H
N
F
f1 (ppm)

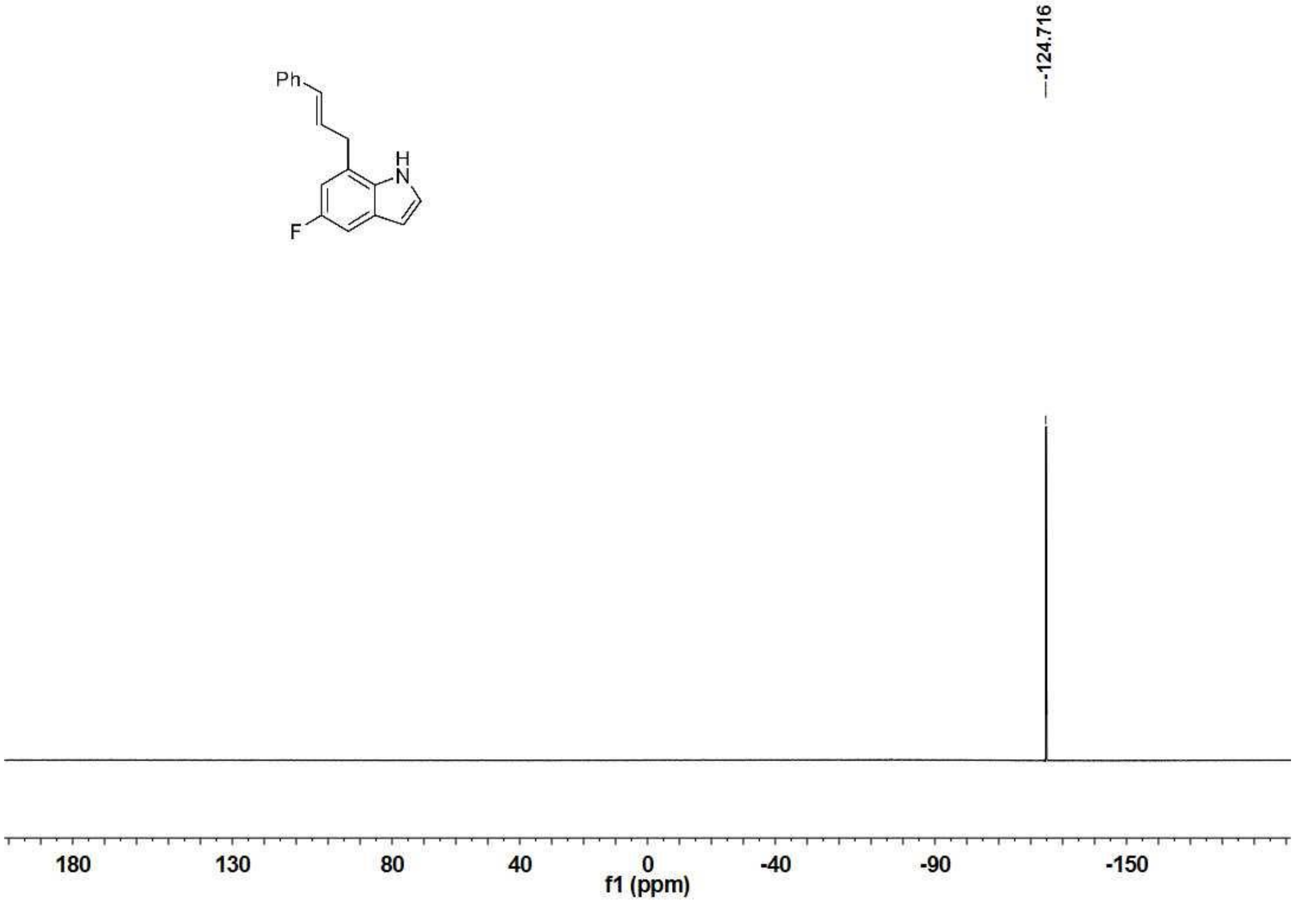

-124.716
Ph
H
N
F
180
130
80
40
0
-40
-90
-150
f1 (ppm)

34

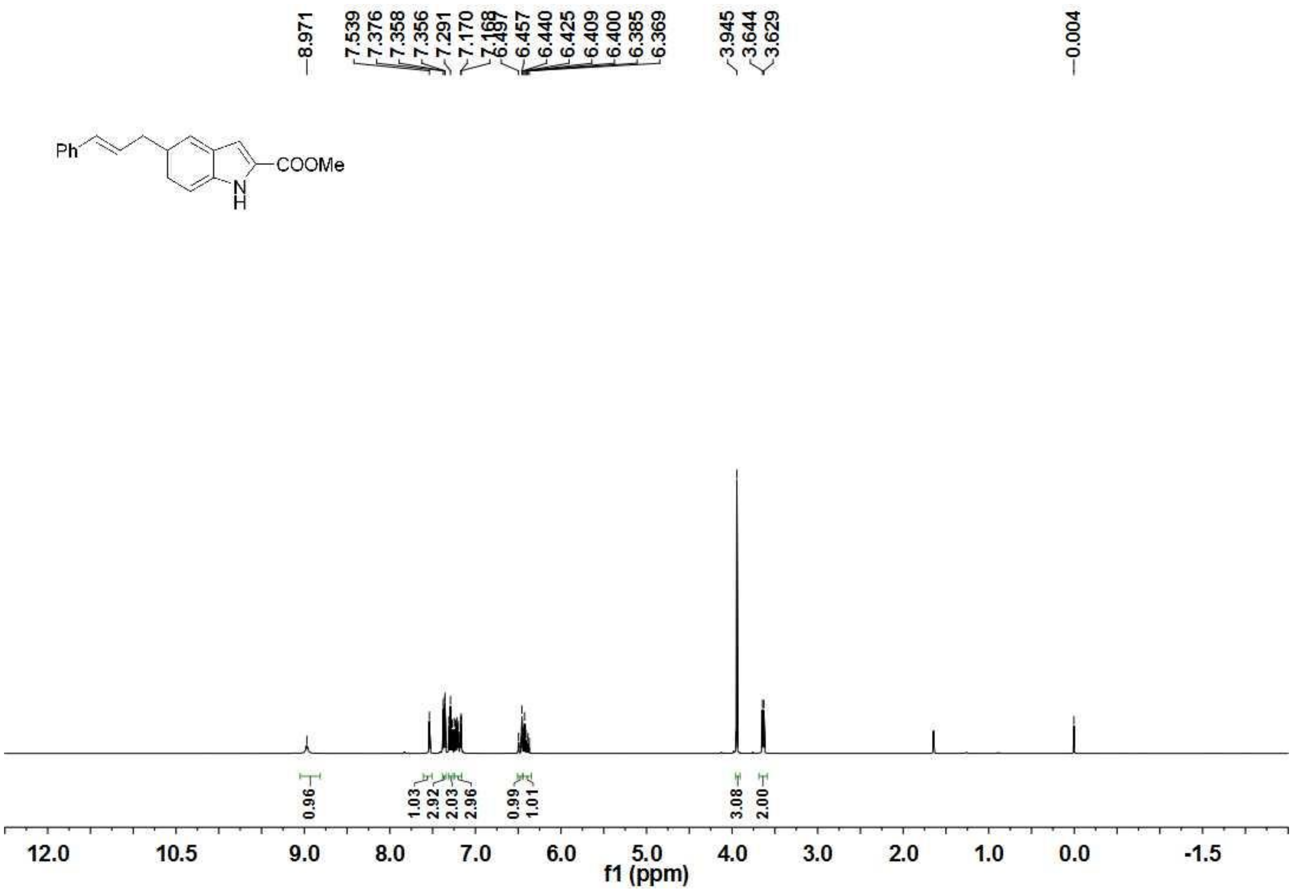

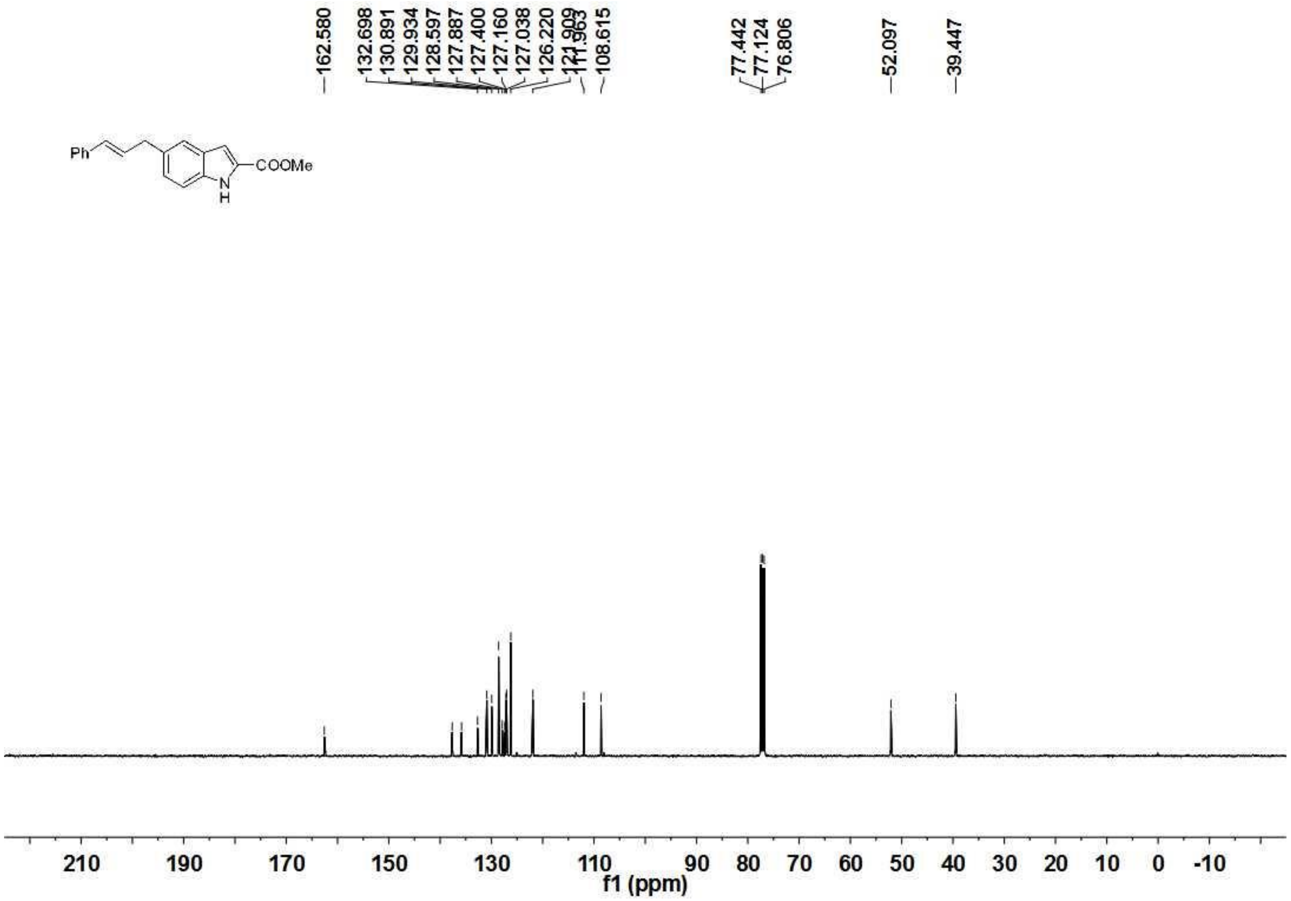

35

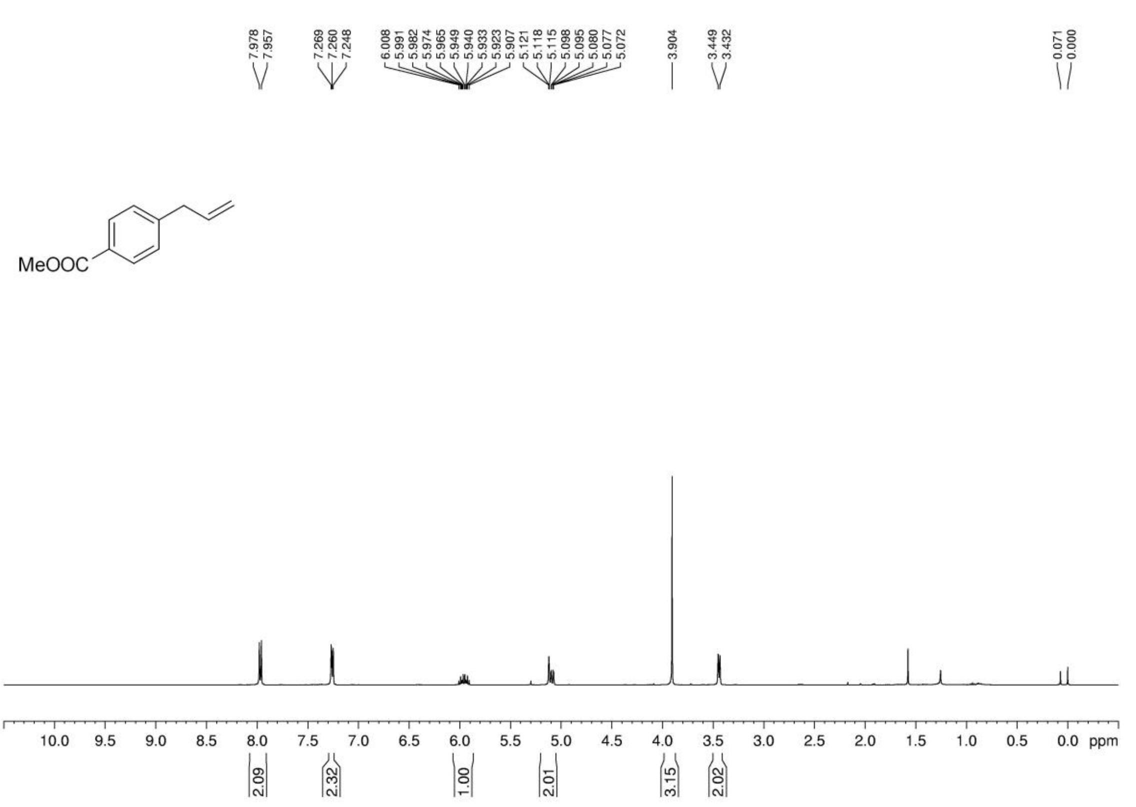

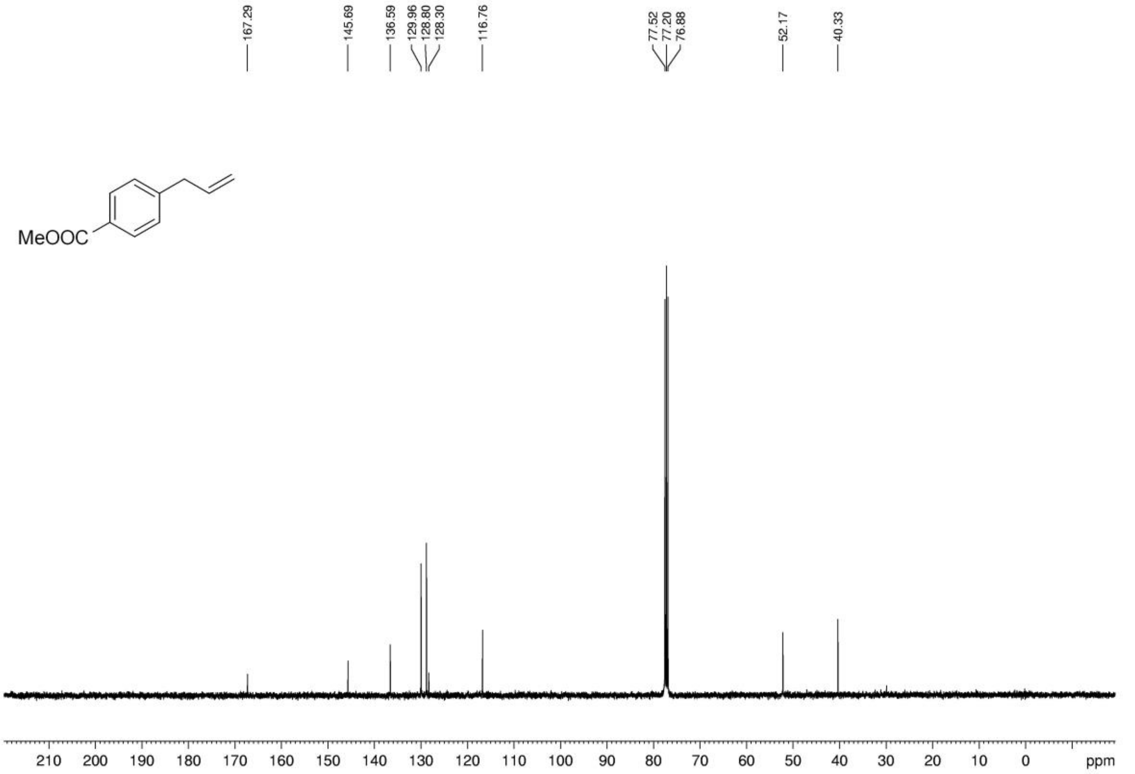

36

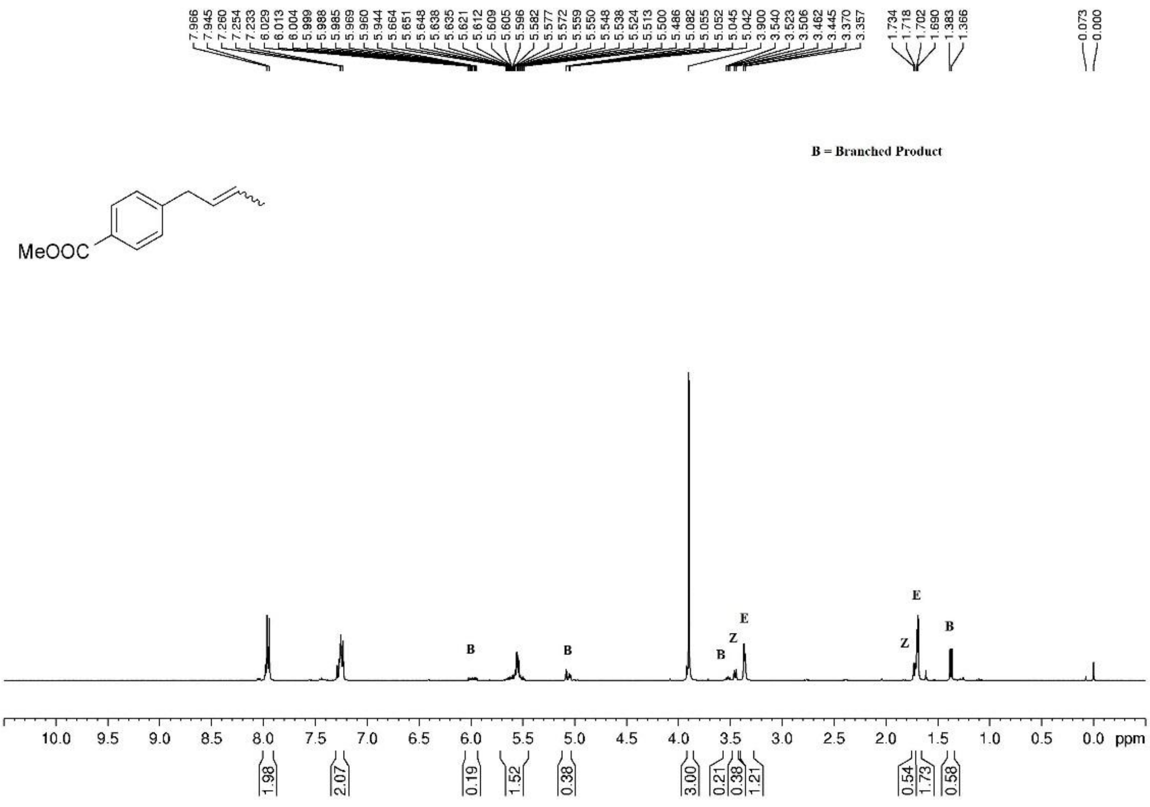

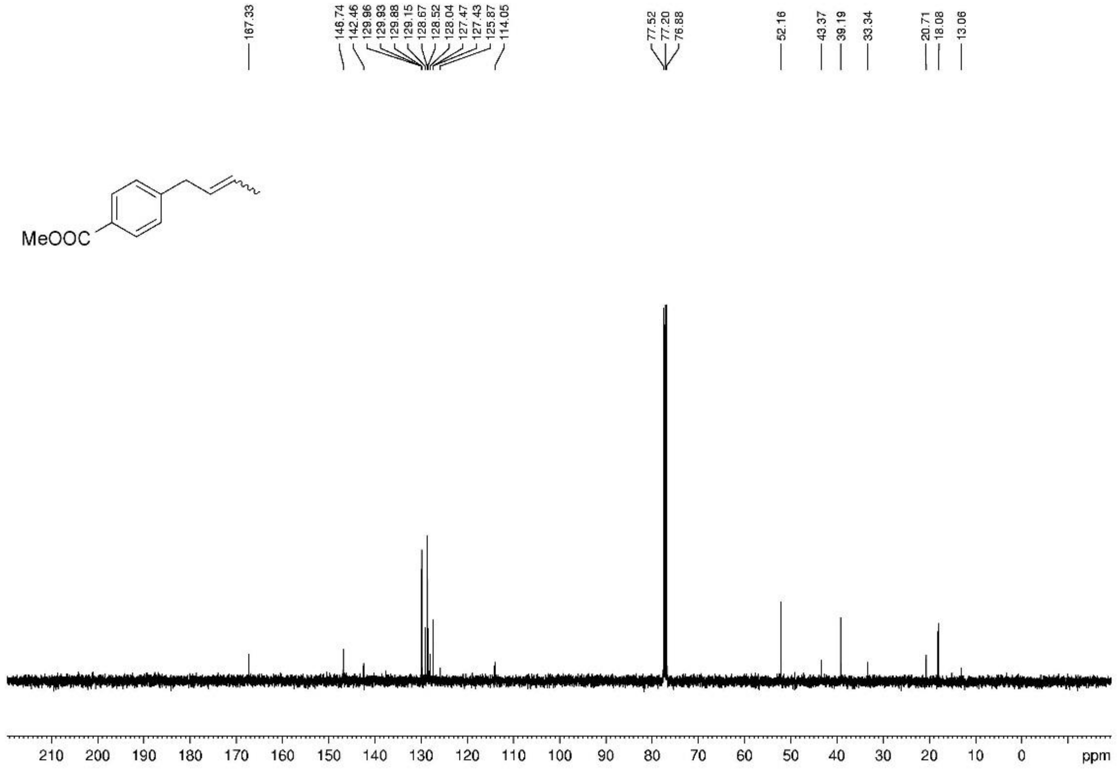

37

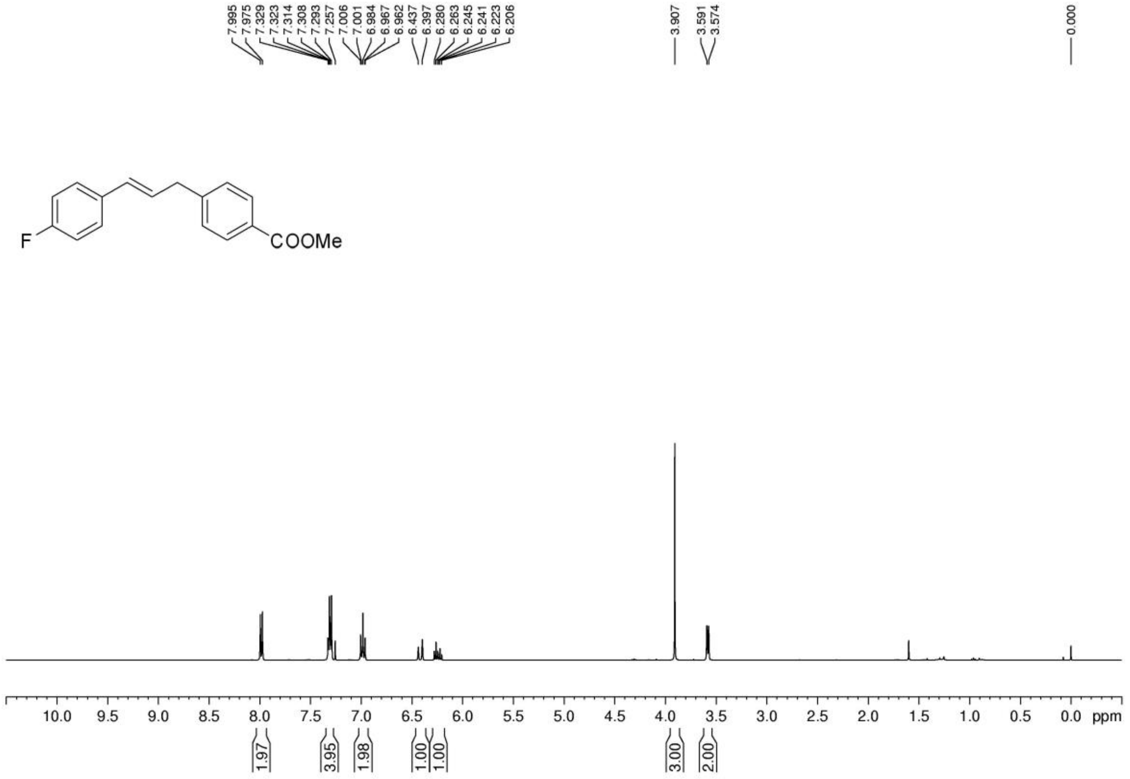

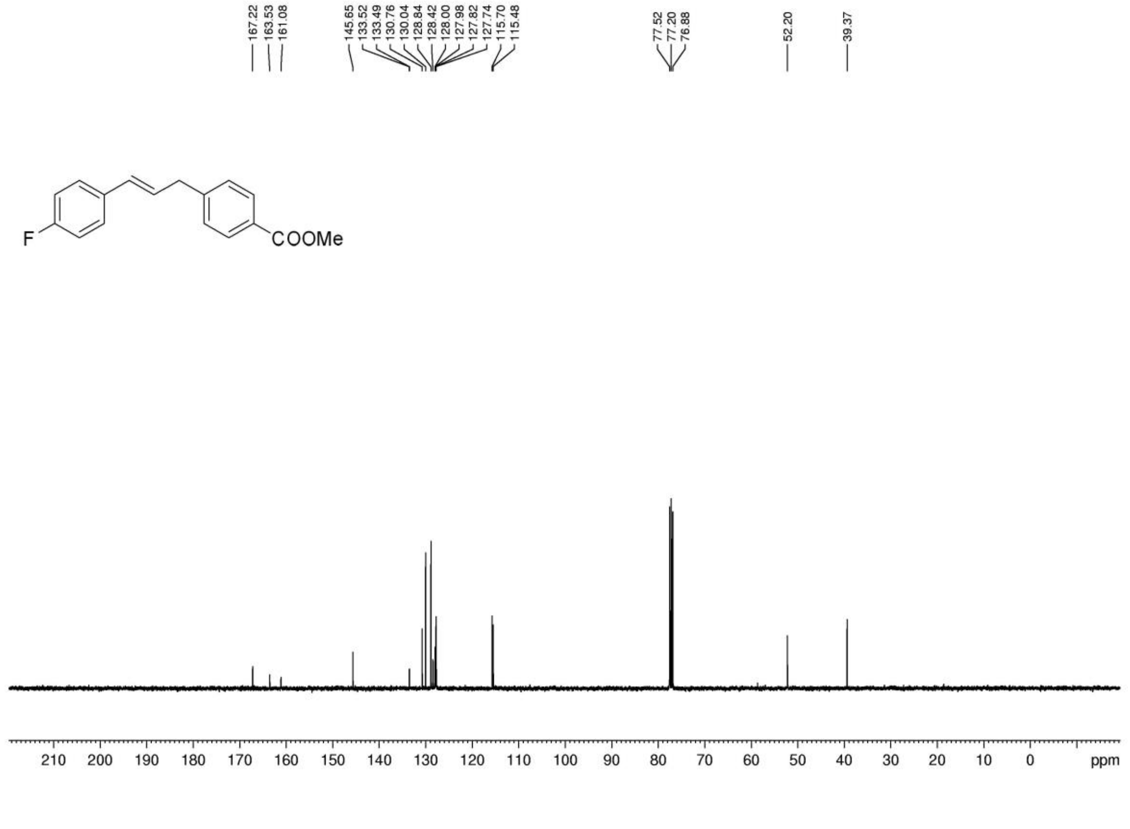

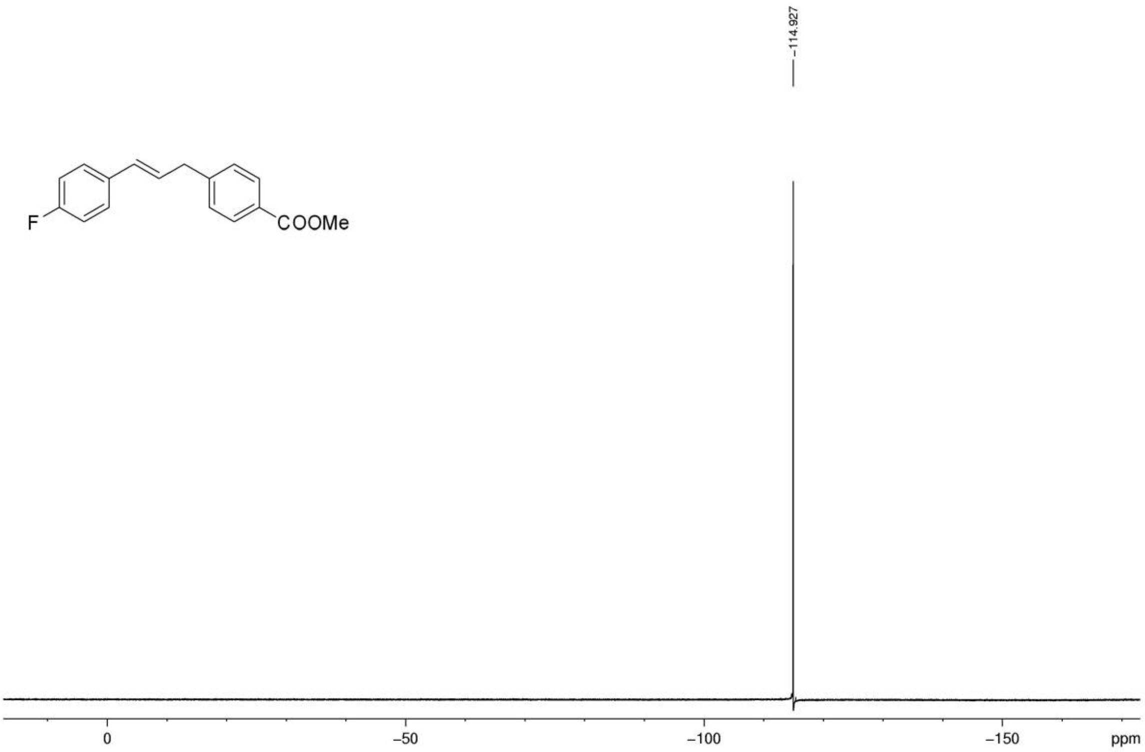

-114.927
F
COOMe
0
−50
−100
−150
ppm

38

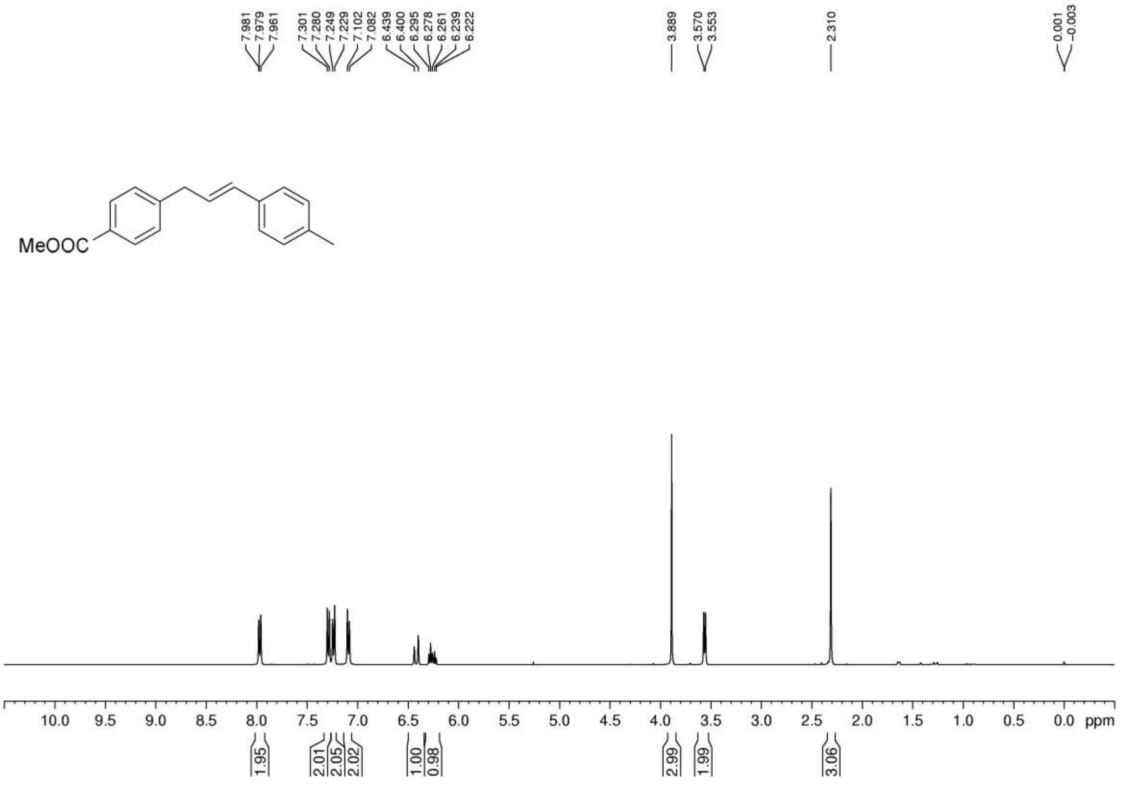
7.981
7.979
7.961
7.301
7.280
7.249
7.229
7.102
7.082
6.439
6.400
6.295
6.278
6.261
6.239
6.222
3.889
3.570
3.553
2.310
0.001
-0.003
MeOOC
10.0 9.5 9.0 8.5 8.0 7.5 7.0 6.5 6.0 5.5 5.0 4.5 4.0 3.5 3.0 2.5 2.0 1.5 1.0 0.5 0.0 ppm
1.95
2.01
2.05
2.02
1.00
0.98
2.99
1.99
3.06

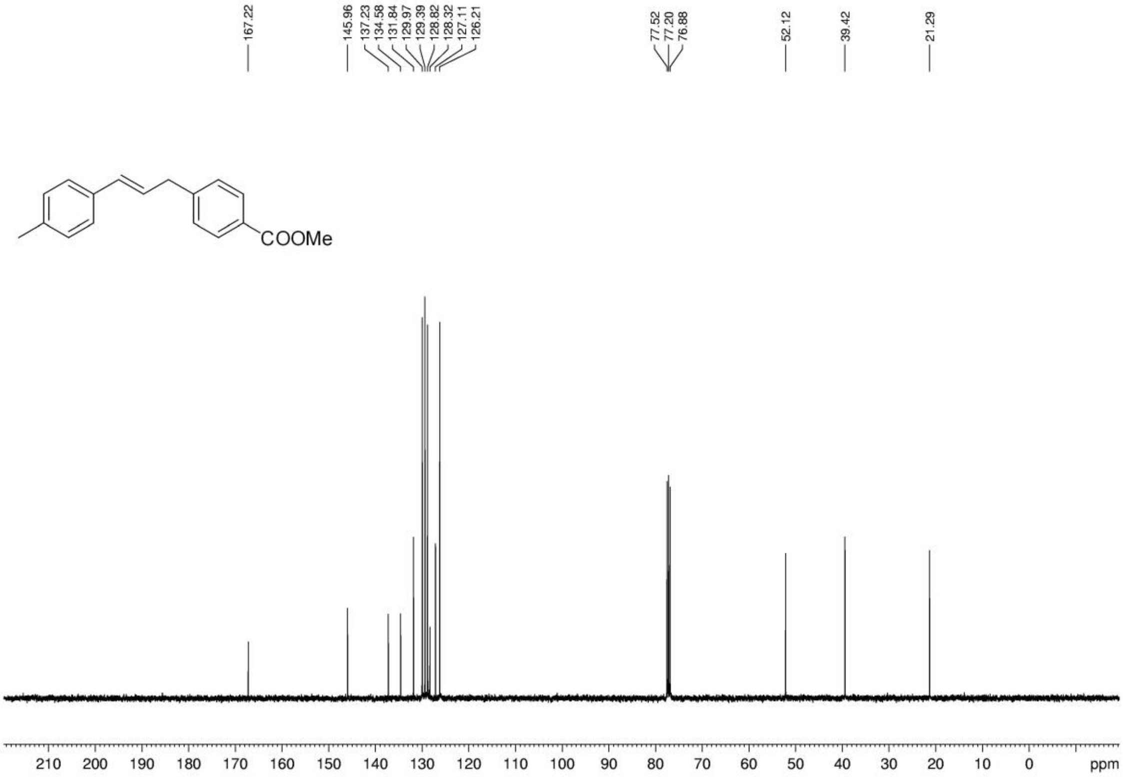
167.22
145.96
137.23
134.58
131.84
129.97
129.39
128.82
128.32
127.11
126.21
77.52
77.20
76.88
52.12
39.42
21.29
COOMe
210 200 190 180 170 160 150 140 130 120 110 100 90 80 70 60 50 40 30 20 10 0 ppm

39

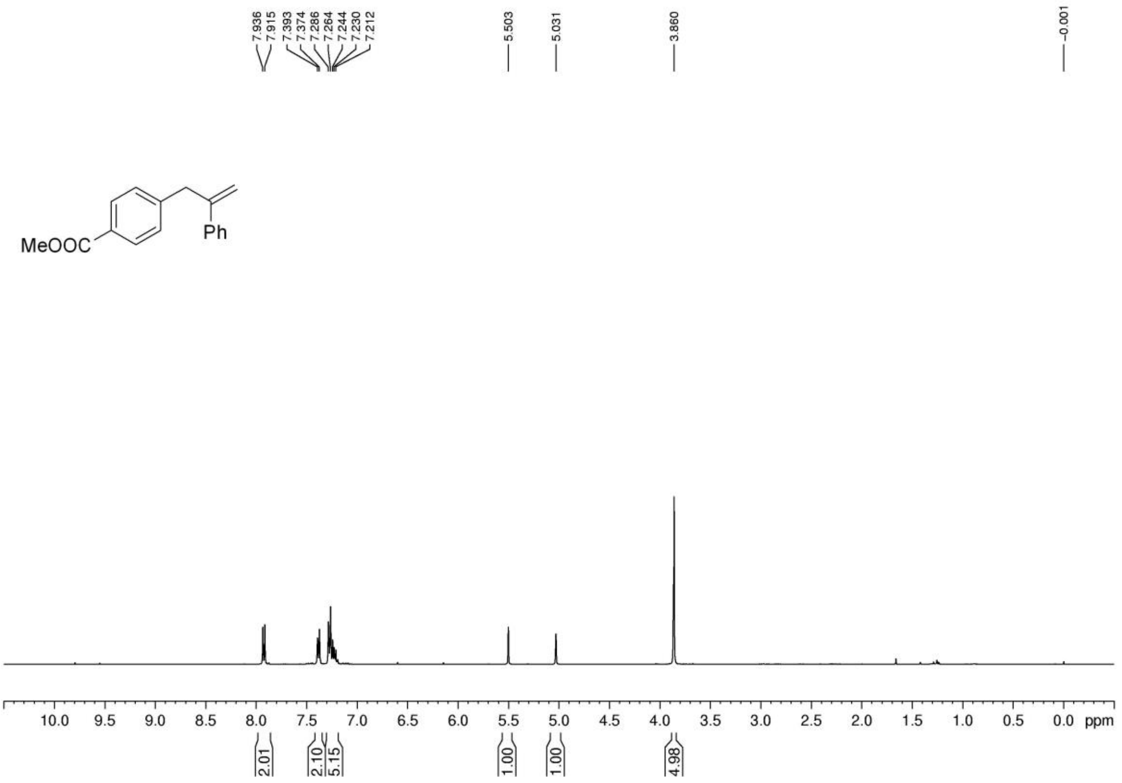

7.936
7.915
7.393
7.374
7.286
7.264
7.244
7.230
7.212
5.503
5.031
3.860
-0.001
MeOOC
Ph
10.0 9.5 9.0 8.5 8.0 7.5 7.0 6.5 6.0 5.5 5.0 4.5 4.0 3.5 3.0 2.5 2.0 1.5 1.0 0.5 0.0 ppm
2.01
2.10
5.15
1.00
1.00
4.98

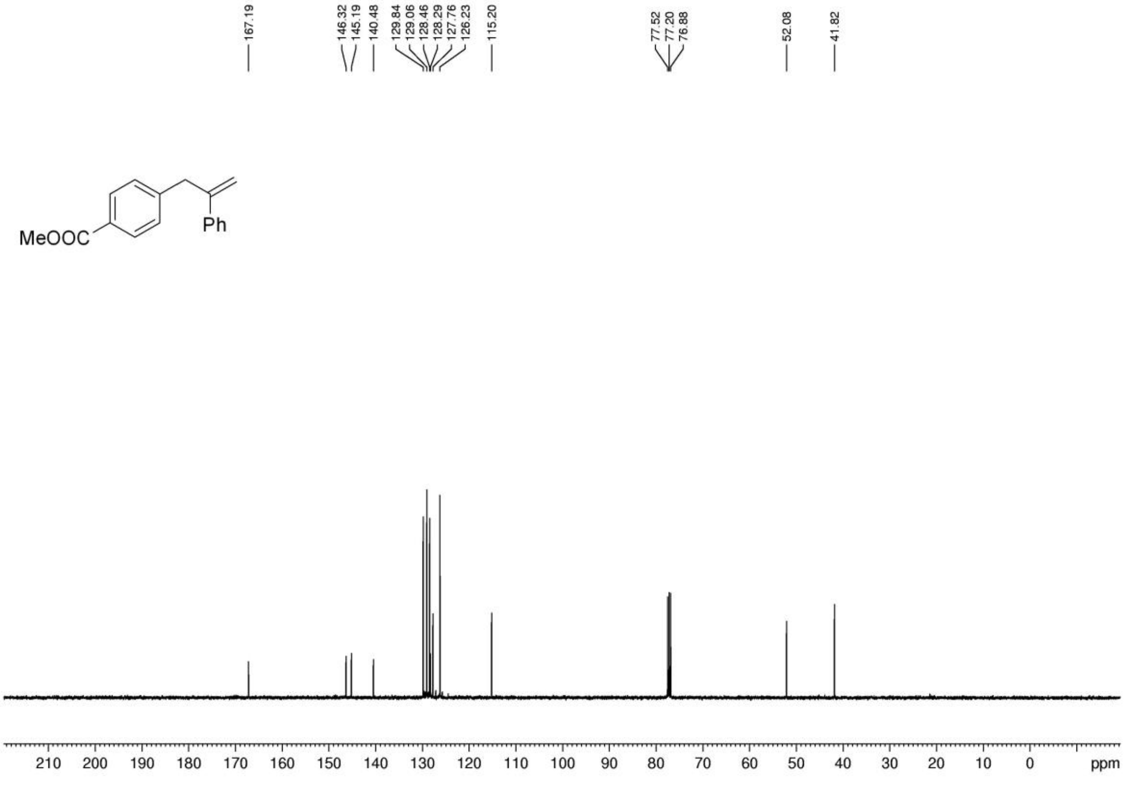

167.19
146.32
145.19
140.48
129.84
129.06
128.46
128.29
127.76
126.23
115.20
77.52
77.20
76.88
52.08
41.82
MeOOC
Ph
210 200 190 180 170 160 150 140 130 120 110 100 90 80 70 60 50 40 30 20 10 0 ppm

40

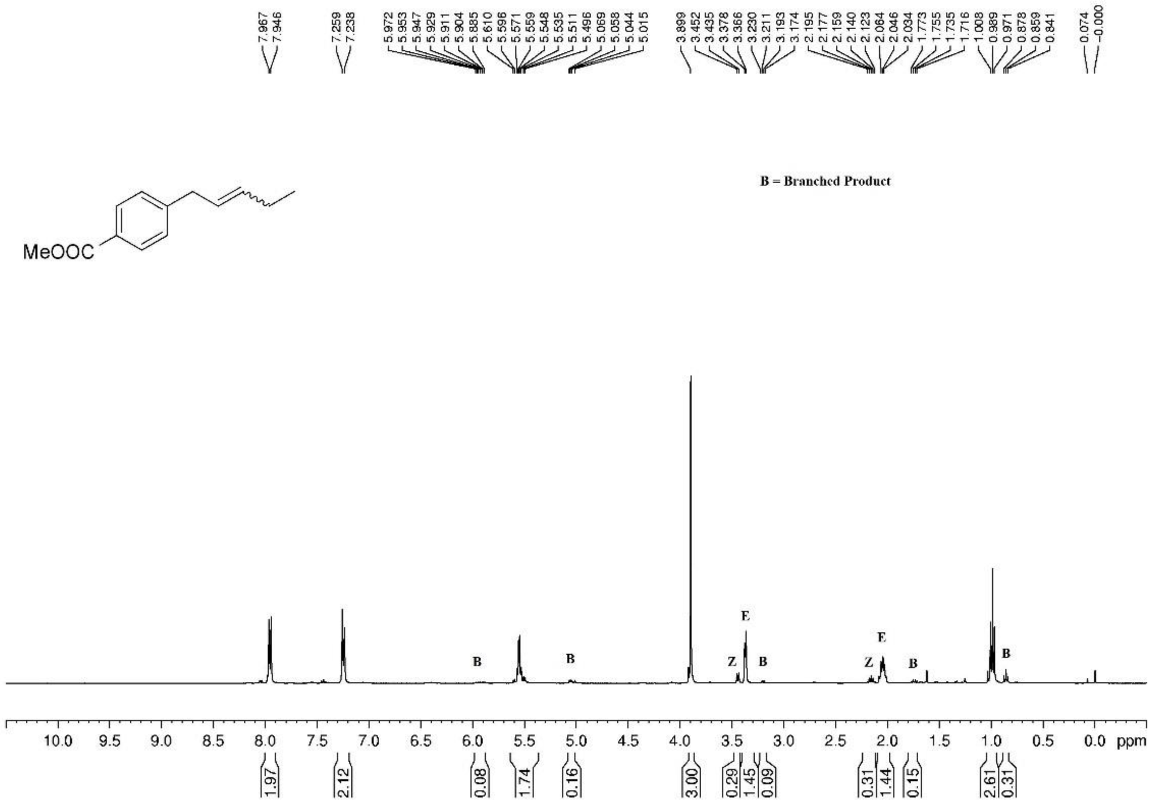
7.967
7.946
7.259
7.238
5.972
5.953
5.947
5.929
5.911
5.904
5.885
5.610
5.596
5.571
5.559
5.548
5.535
5.511
5.496
5.069
5.058
5.044
5.015
3.899
3.452
3.435
3.378
3.366
3.230
3.211
3.193
3.174
2.195
2.177
2.159
2.140
2.123
2.064
2.046
2.034
1.773
1.755
1.735
1.716
1.008
0.989
0.971
0.878
0.859
0.841
0.074
-0.000
MeOOC
B = Branched Product
B
B
E
Z
B
E
Z
B
B
10.0 9.5 9.0 8.5 8.0 7.5 7.0 6.5 6.0 5.5 5.0 4.5 4.0 3.5 3.0 2.5 2.0 1.5 1.0 0.5 0.0 ppm
1.97
2.12
0.08
1.74
0.16
3.00
0.29
1.45
0.09
0.31
1.44
0.15
2.61
0.31

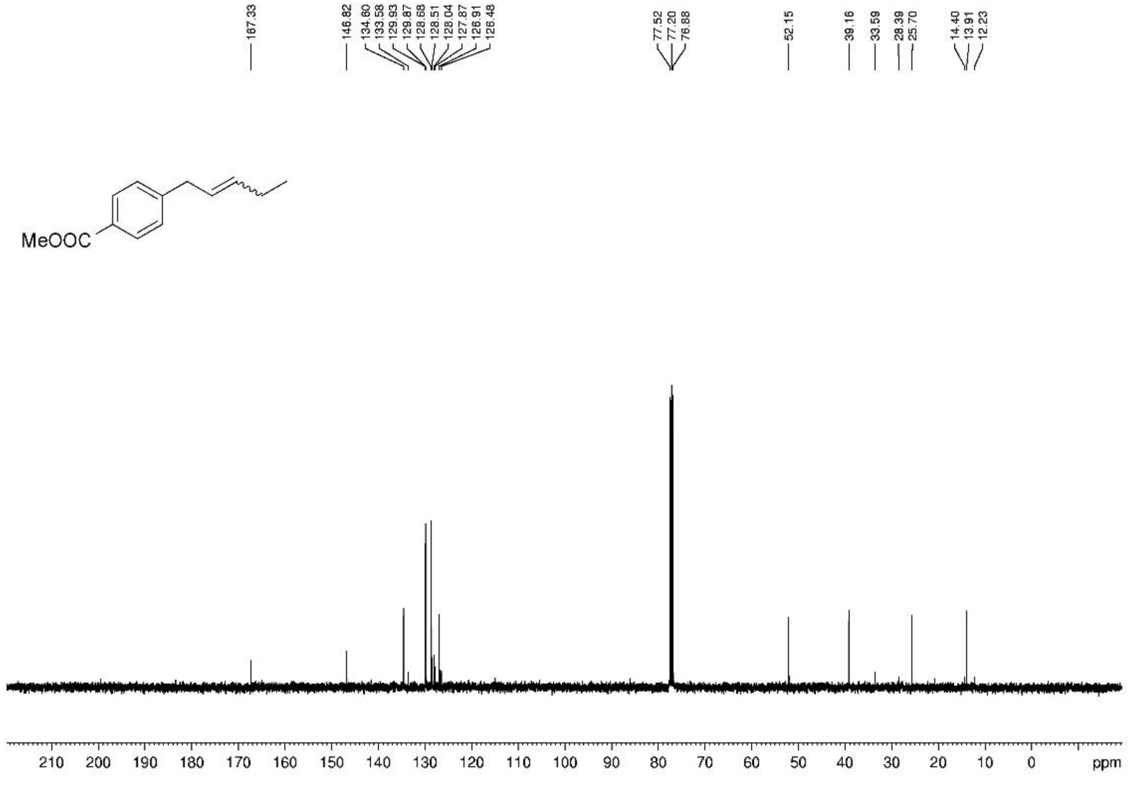
167.33
146.82
134.80
133.58
129.93
129.87
128.68
128.51
128.04
127.87
126.91
126.48
77.52
77.20
76.88
52.15
39.16
33.59
28.39
25.70
14.40
13.91
12.23
MeOOC
210 200 190 180 170 160 150 140 130 120 110 100 90 80 70 60 50 40 30 20 10 0 ppm

41

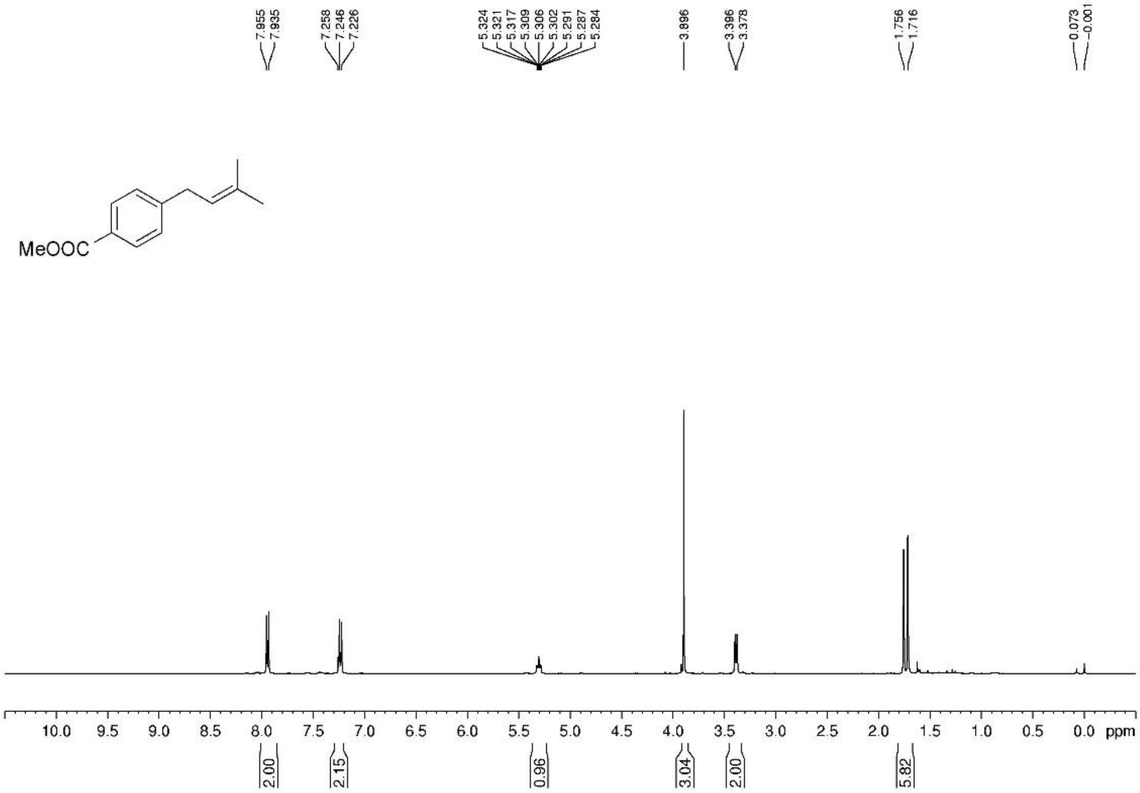

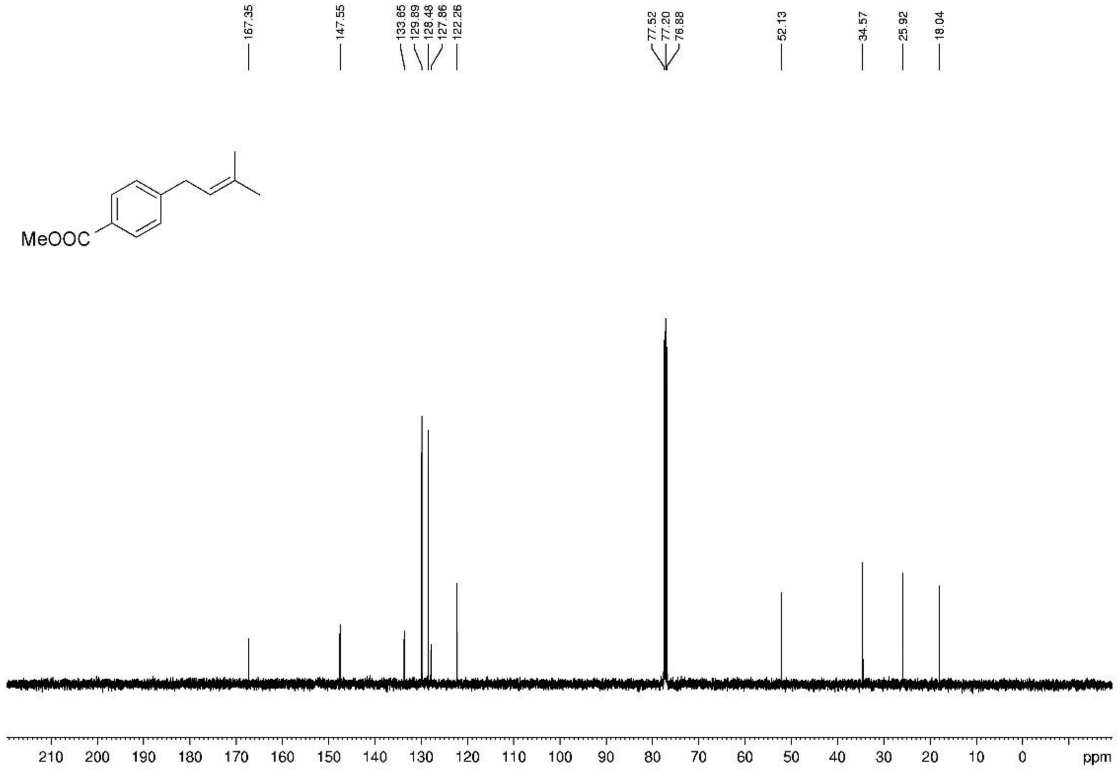

42

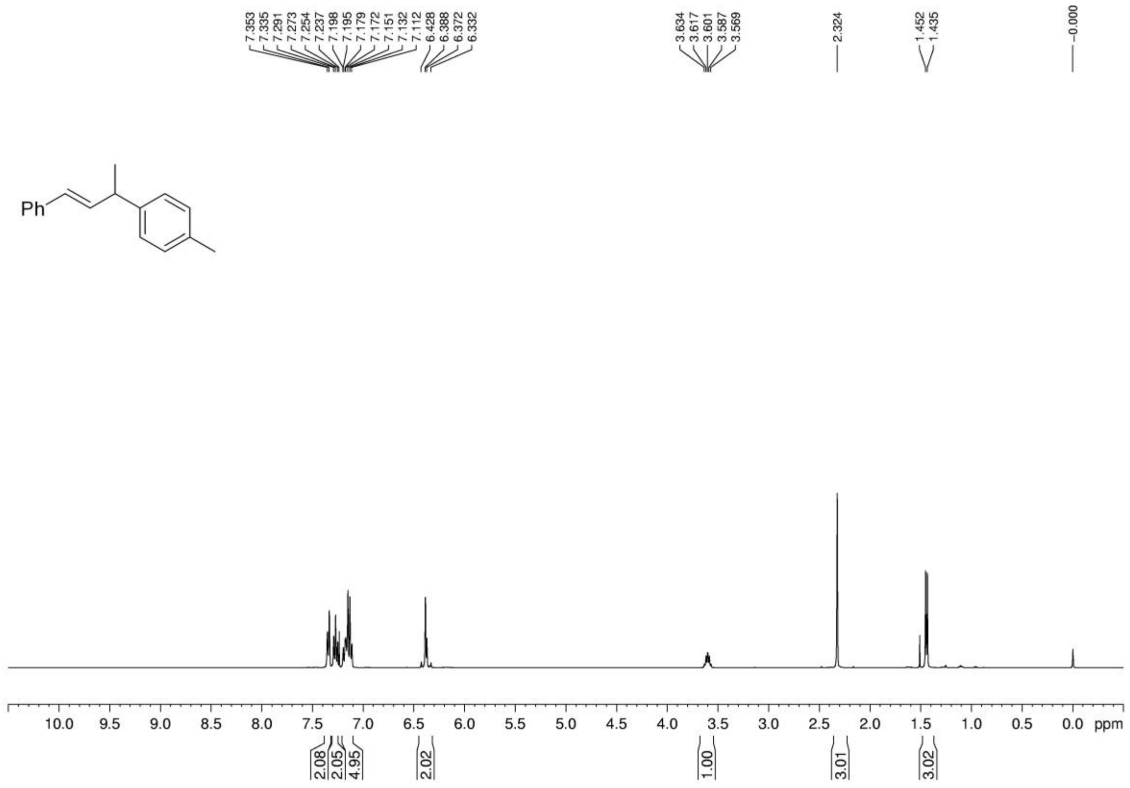

7.353
7.335
7.291
7.273
7.254
7.237
7.198
7.195
7.179
7.172
7.151
7.132
7.112
6.428
6.388
6.372
6.332
3.634
3.617
3.601
3.587
3.569
2.324
1.452
1.435
0.000
Ph
10.0 9.5 9.0 8.5 8.0 7.5 7.0 6.5 6.0 5.5 5.0 4.5 4.0 3.5 3.0 2.5 2.0 1.5 1.0 0.5 0.0 ppm
2.08
2.05
4.95
2.02
1.00
3.01
3.02

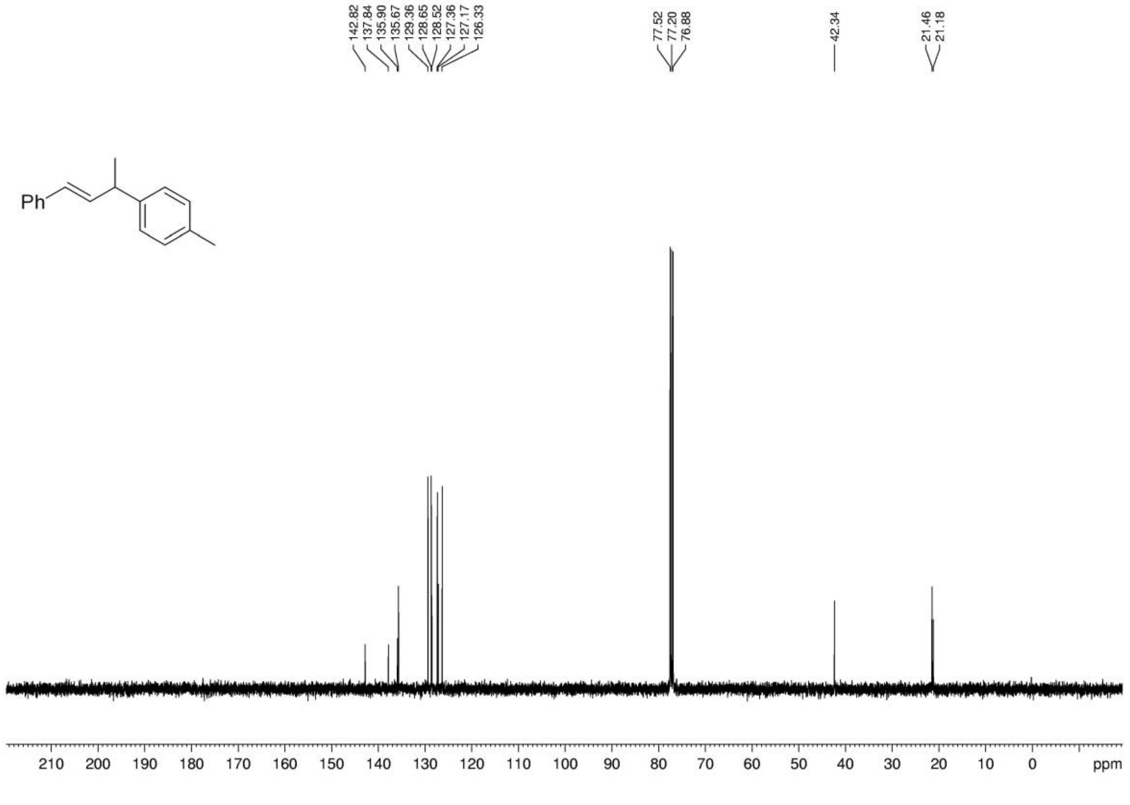

142.82
137.84
135.90
135.67
129.36
128.65
128.52
127.36
127.17
126.33
77.52
77.20
76.88
42.34
21.46
21.18
Ph
210 200 190 180 170 160 150 140 130 120 110 100 90 80 70 60 50 40 30 20 10 0 ppm

43

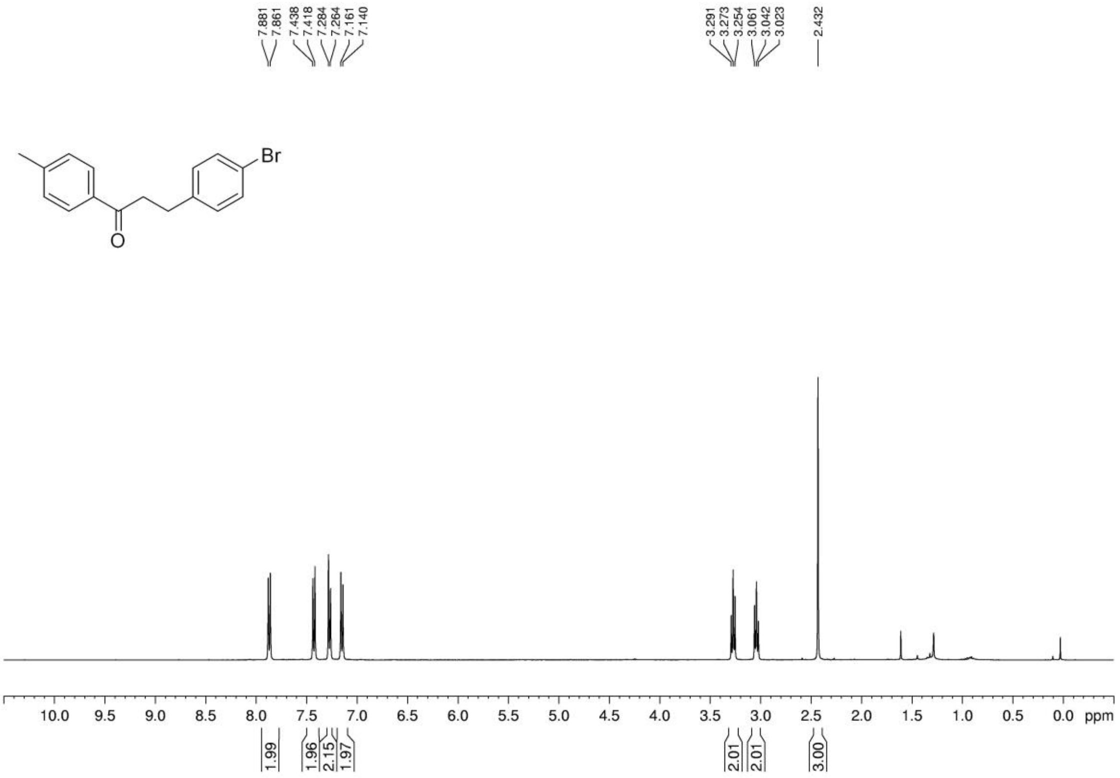
7.881
7.861
7.438
7.418
7.284
7.264
7.161
7.140
3.291
3.273
3.254
3.061
3.042
3.023
2.432
Br
O
10.0 9.5 9.0 8.5 8.0 7.5 7.0 6.5 6.0 5.5 5.0 4.5 4.0 3.5 3.0 2.5 2.0 1.5 1.0 0.5 0.0 ppm
1.99
1.96
2.15
1.97
2.01
2.01
3.00

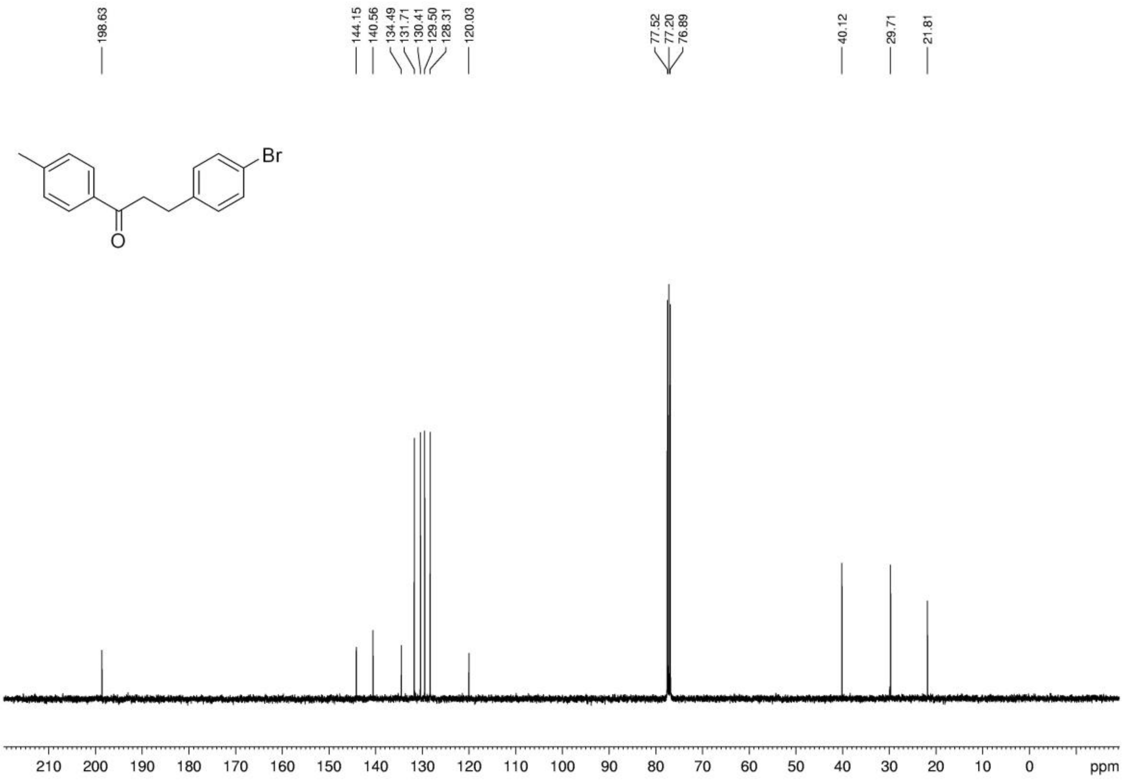
198.63
144.15
140.56
134.49
131.71
130.41
129.50
128.31
120.03
77.52
77.20
76.89
40.12
29.71
21.81
Br
O
210 200 190 180 170 160 150 140 130 120 110 100 90 80 70 60 50 40 30 20 10 0 ppm

44

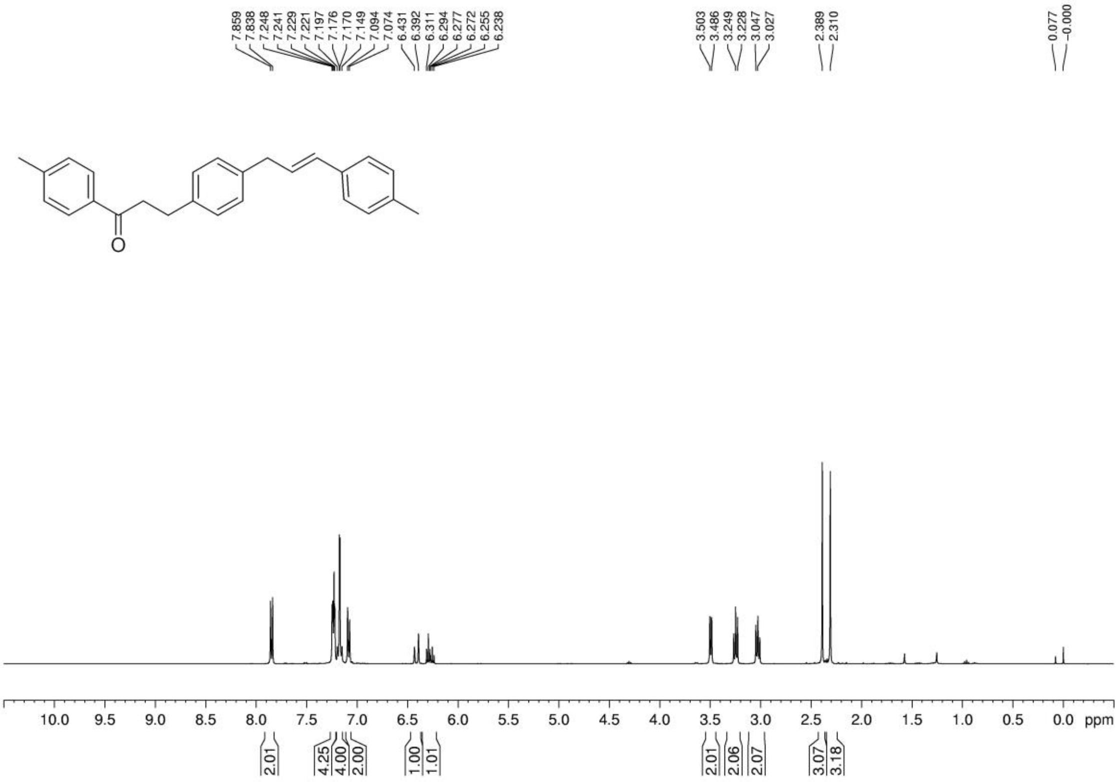
7.859
7.838
7.248
7.241
7.229
7.221
7.197
7.176
7.170
7.149
7.094
7.074
6.431
6.392
6.311
6.294
6.277
6.272
6.255
6.238
3.503
3.486
3.249
3.228
3.047
3.027
2.389
2.310
0.077
-0.000
O
10.0 9.5 9.0 8.5 8.0 7.5 7.0 6.5 6.0 5.5 5.0 4.5 4.0 3.5 3.0 2.5 2.0 1.5 1.0 0.5 0.0 ppm
2.01
4.25
4.00
2.00
1.00
1.01
2.01
2.06
2.07
3.07
3.18

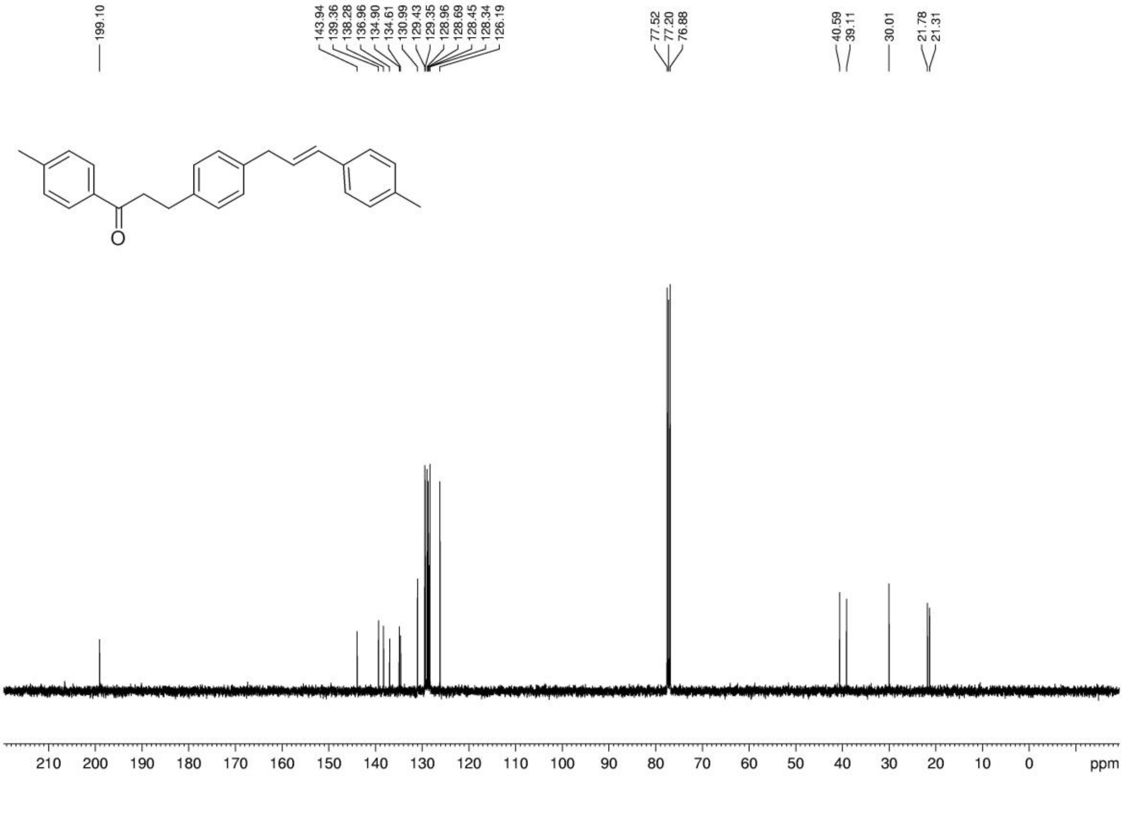
199.10
143.94
139.36
138.28
136.96
134.90
134.61
130.99
129.43
129.35
128.96
128.69
128.45
128.34
126.19
77.52
77.20
76.88
40.59
39.11
30.01
21.78
21.31
O
210 200 190 180 170 160 150 140 130 120 110 100 90 80 70 60 50 40 30 20 10 0 ppm

45

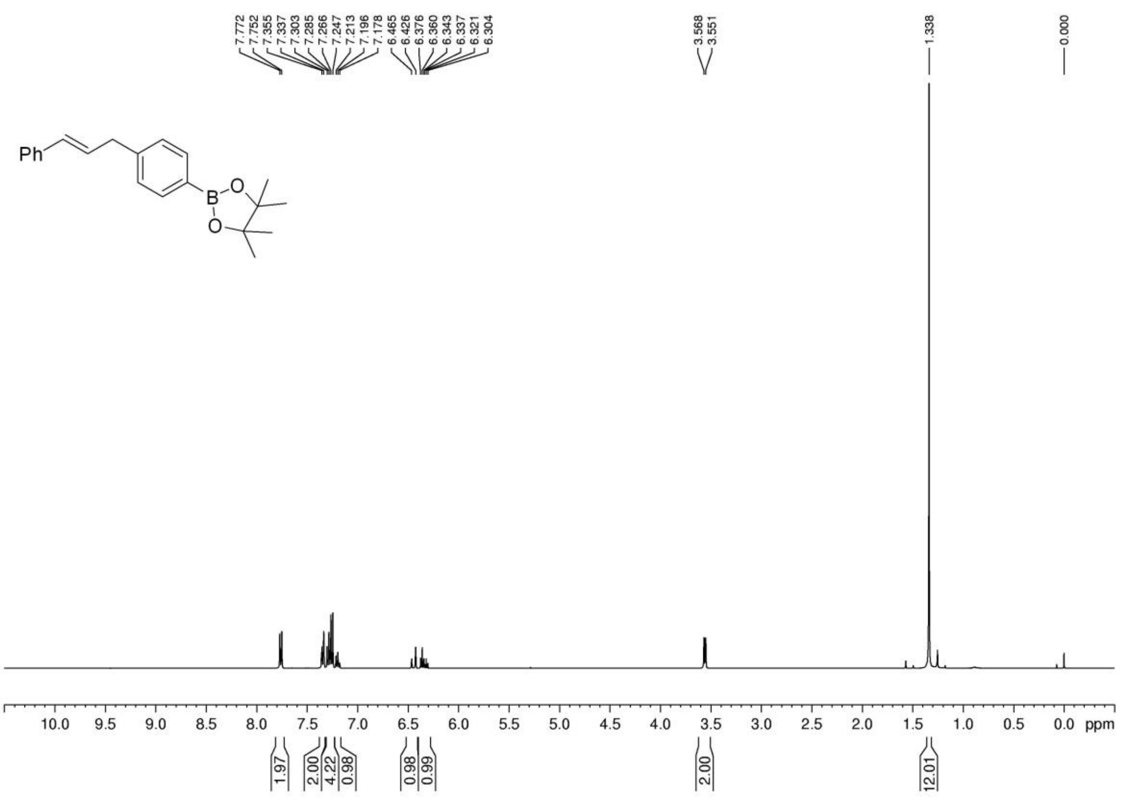

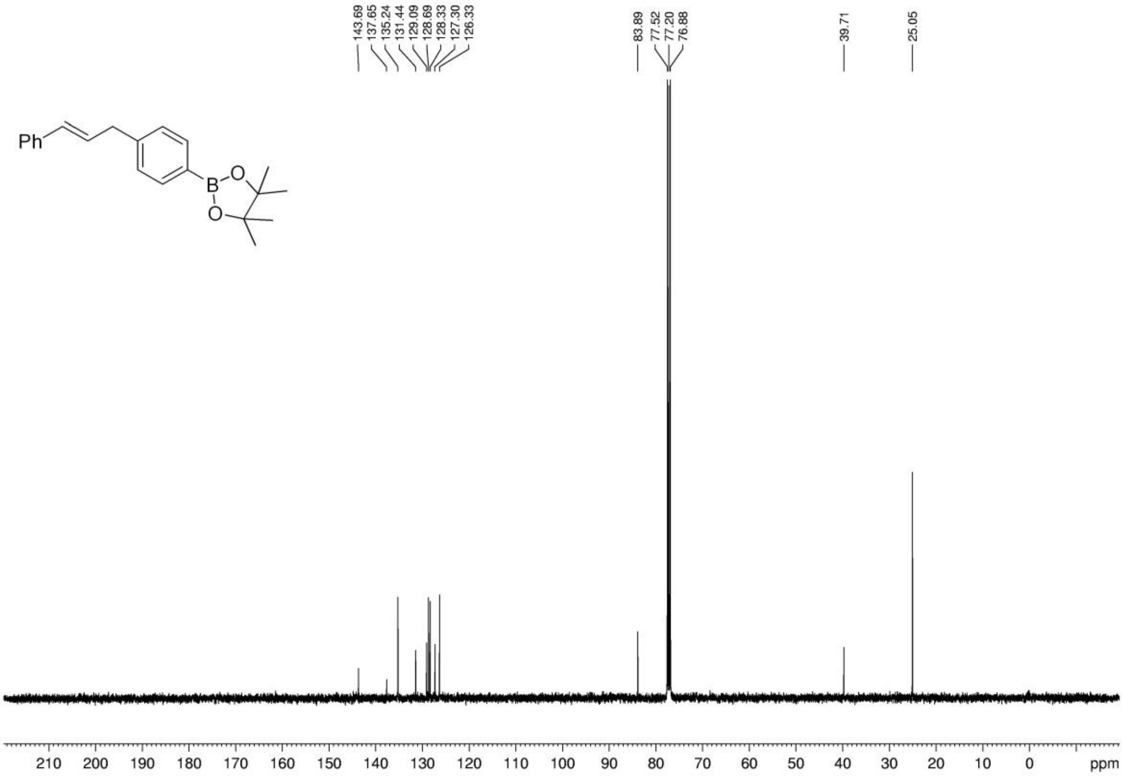

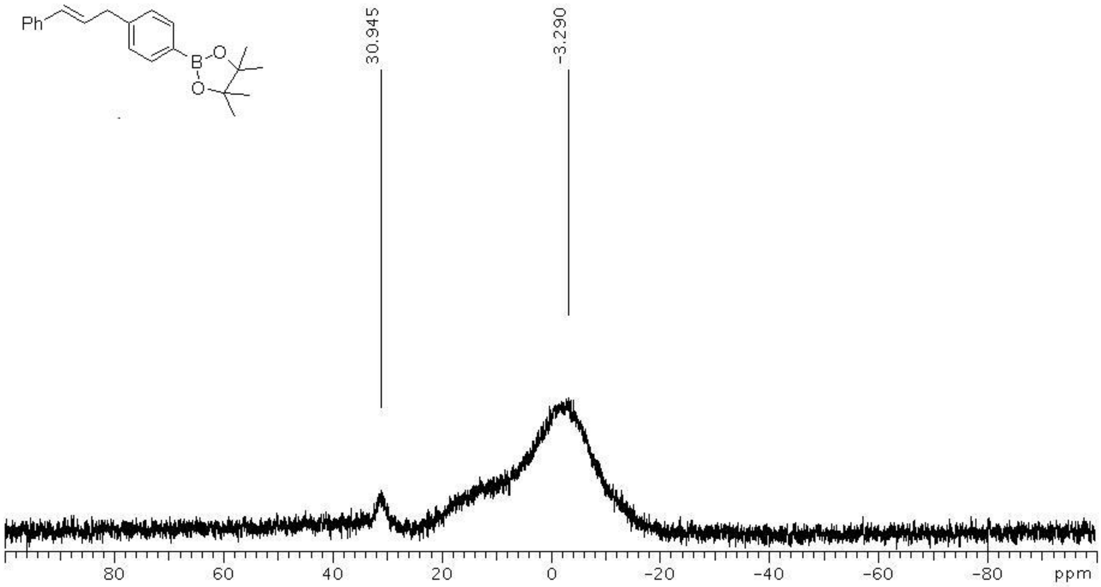

Ph
B
O
O
30.945
-3.290
80
60
40
20
0
-20
-40
-60
-80
ppm

46

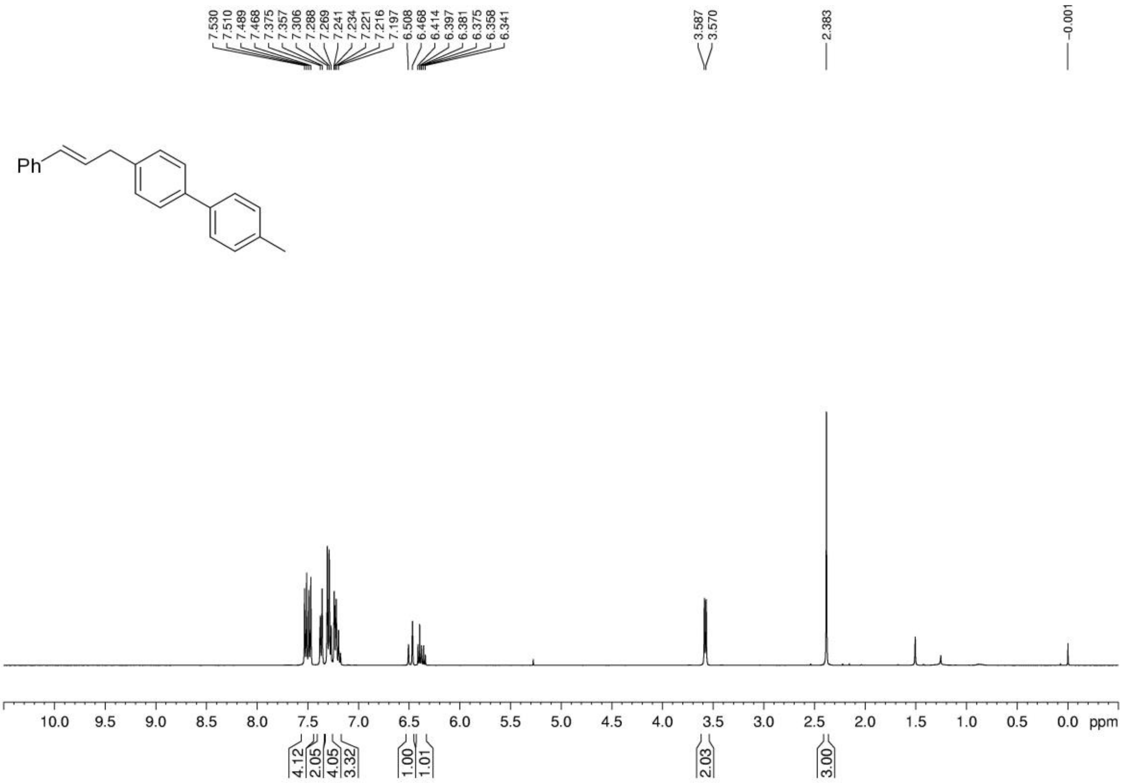

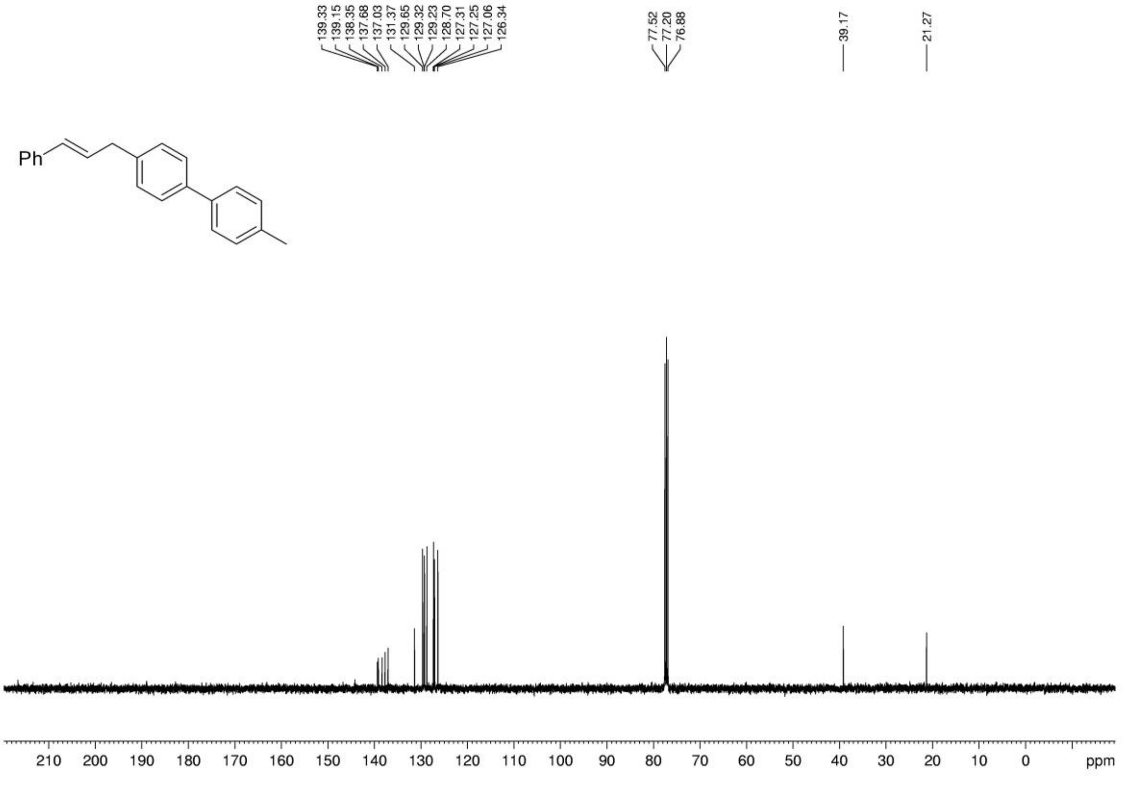

49

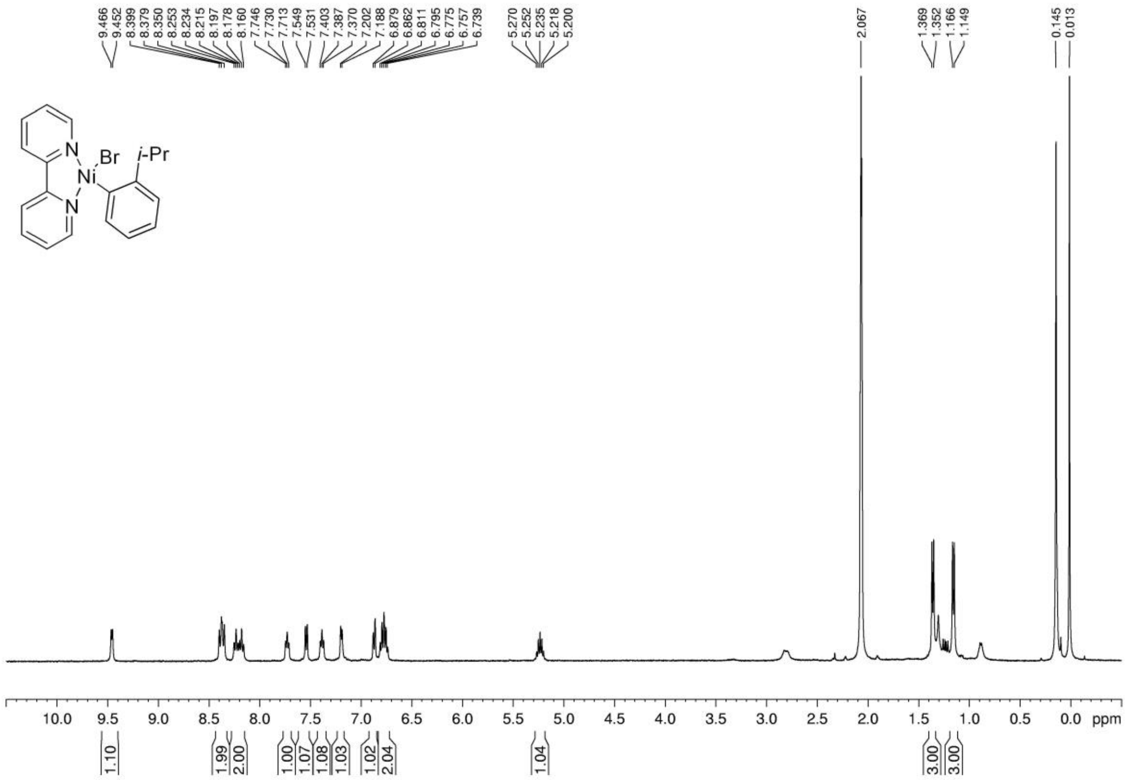

9.466
9.452
8.399
8.379
8.350
8.253
8.234
8.215
8.197
8.178
8.160
7.746
7.730
7.713
7.549
7.531
7.403
7.387
7.370
7.202
7.188
6.879
6.862
6.811
6.795
6.775
6.757
6.739
5.270
5.252
5.235
5.218
5.200
2.067
1.369
1.352
1.166
1.149
0.145
0.013
Br
i-Pr
Ni
N
N
10.0 9.5 9.0 8.5 8.0 7.5 7.0 6.5 6.0 5.5 5.0 4.5 4.0 3.5 3.0 2.5 2.0 1.5 1.0 0.5 0.0 ppm
1.10
1.99
2.00
1.00
1.07
1.08
1.03
1.02
2.04
1.04
3.00
3.00

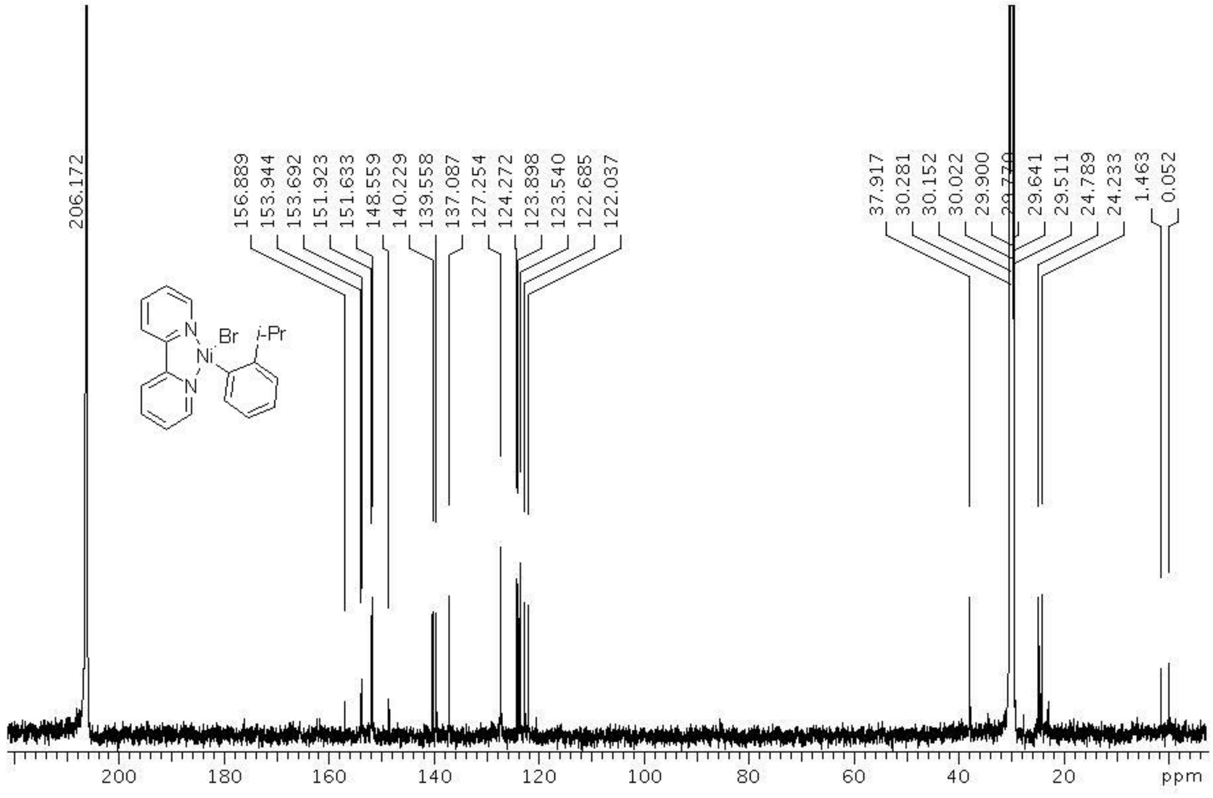

206.172
156.889
153.944
153.692
151.923
151.633
148.559
140.229
139.558
137.087
127.254
124.272
123.898
123.540
122.685
122.037
37.917
30.281
30.152
30.022
29.900
29.641
29.511
24.789
24.233
1.463
0.052
Br
i-Pr
Ni
N
N
200 180 160 140 120 100 80 60 40 20 ppm

50

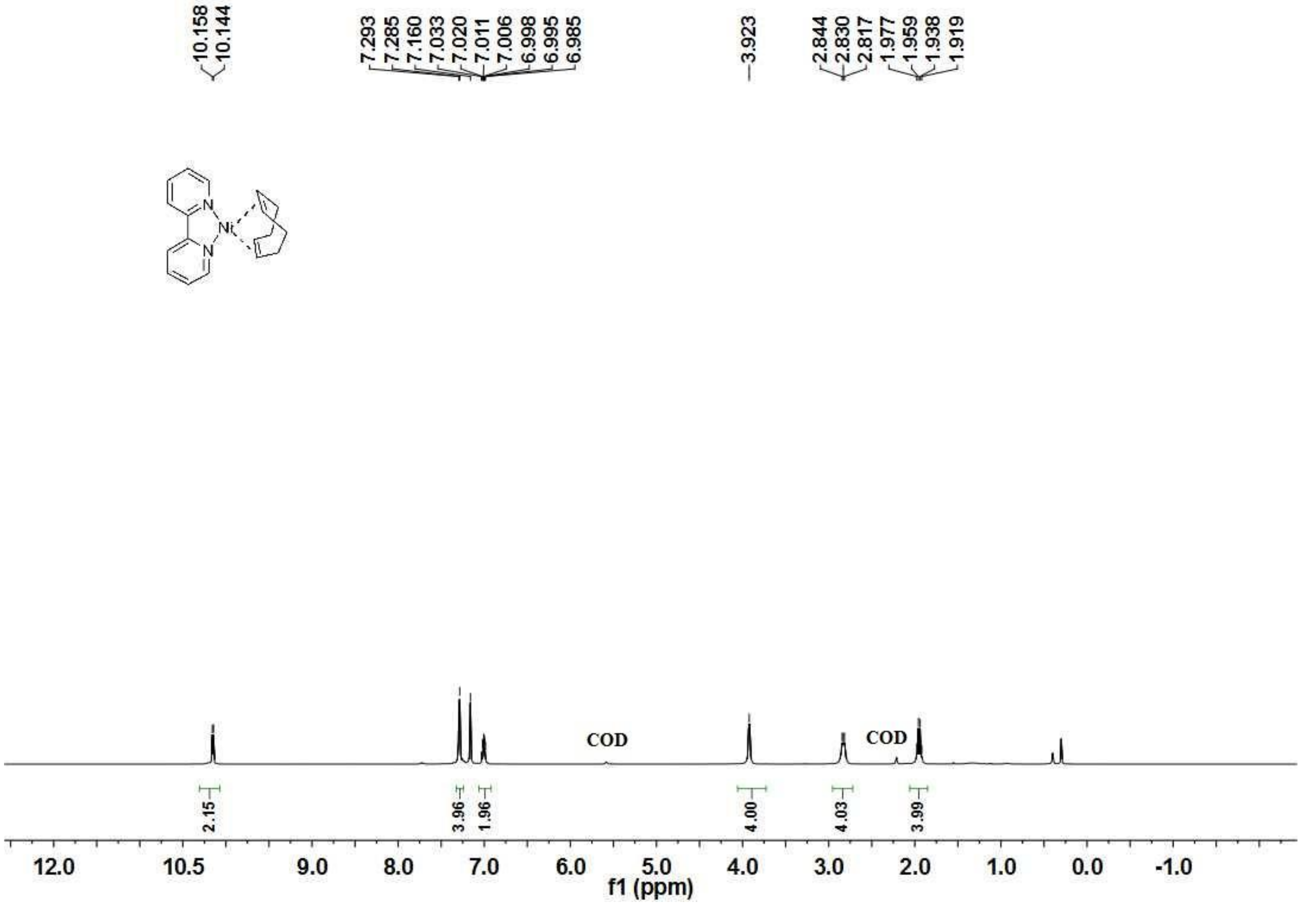
10.158
10.144
7.293
7.285
7.160
7.033
7.020
7.011
7.006
6.998
6.995
6.985
3.923
2.844
2.830
2.817
1.977
1.959
1.938
1.919
COD
COD
2.15
3.96
1.96
4.00
4.03
3.99
f1 (ppm)

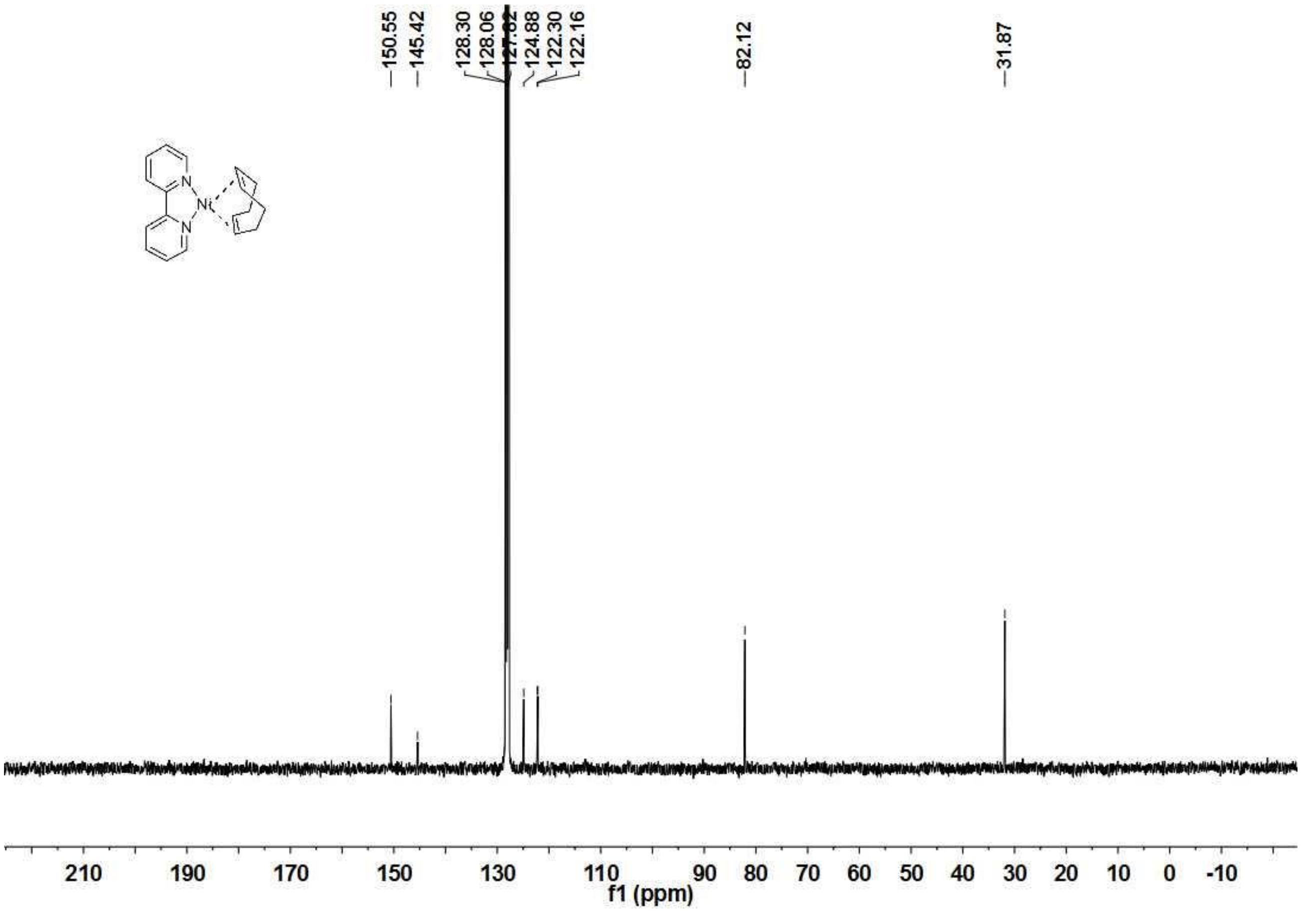
150.55
145.42
128.30
128.06
124.88
122.30
122.16
82.12
31.87
f1 (ppm)
